# New Pyranone Derivatives and Sesquiterpenoid Isolated from the Endophytic Fungus *Xylaria* sp. Z184

**DOI:** 10.3390/molecules29081728

**Published:** 2024-04-11

**Authors:** Yan Zhang, Yang Jin, Wensi Yan, Peishan Gu, Ziqian Zeng, Ziying Li, Guangtao Zhang, Mi Wei, Yongbo Xue

**Affiliations:** 1 School of Pharmaceutical Sciences (Shenzhen), Shenzhen Campus of Sun Yat-sen University, Shenzhen 518107, China; zhangy2328@mail2.sysu.edu.cn (Y.Z.); jiny67@mail2.sysu.edu.cn (Y.J.); yanws3@mail2.sysu.edu.cn (W.Y.); gupsh@mail2.sysu.edu.cn (P.G.); zengzq8@mail2.sysu.edu.cn (Z.Z.); 2 School of Pharmacy, Binzhou Medical University, Yantai 264003, China; 15853386606@163.com (Z.L.); greatzhangtao@hotmail.com (G.Z.); 3 School of Agriculture, Shenzhen Campus of Sun Yat-sen University, Shenzhen 518107, China; weim29@mail.sysu.edu.cn

**Keywords:** *Xylaria* sp., pyranone derivative, sesquiterpenoid, antimicrobial activity

## Abstract

The fungus *Xylaria* sp. Z184, harvested from the leaves of *Fallopia convolvulus* (L.) Á. Löve, has been isolated for the first time. Chemical investigation on the methanol extract of the culture broth of the titles strain led to the discovery of three new pyranone derivatives, called fallopiaxylaresters A–C (**1**–**3**), and a new bisabolane-type sesquiterpenoid, named fallopiaxylarol A (**4**), along with the first complete set of spectroscopic data for the previously reported pestalotiopyrone M (**5**). Known pyranone derivatives (**6**–**11**), sesquiterpenoids (**12**–**14**), isocoumarin derivatives (**15**–**17**), and an aromatic allenic ether (**18**) were also co-isolated in this study. All new structures were elucidated by the interpretation of HRESIMS, 1D, 2D NMR spectroscopy, and quantum chemical computation approach. The in vitro antimicrobial, anti-inflammatory, and α-glucosidase-inhibitory activities of the selected compounds and the crude extract were evaluated. The extract was shown to inhibit nitric oxide (NO) production induced by lipopolysaccharide (LPS) in murine RAW264.7 macrophage cells, with an inhibition rate of 77.28 ± 0.82% at a concentration of 50 μg/mL. The compounds **5**, **7**, and **8** displayed weak antibacterial activity against *Staphylococcus areus* subsp. *aureus* at a concentration of 100 μM.

## 1. Introduction

Natural products (NPs) have always been an indispensable source of new drugs [1]. As an important source of NPs with novel structures and high-value biological activities, plant endophytic fungi have always been attracting broad attention from natural product chemists and pharmacologists [2,3]. Xylariaceae is one of the largest, most commonly encountered, and highly diverse fungal families of the Ascomycota [4]. The genus *Xylaria*, belonging to the family Xylariaceae, is medicinal fungi commonly found in decaying plant tissues and is widely distributed in temperate, tropical, and subtropical regions [5,6]. So far, more than 200 bioactive compounds (>100 new ones) were isolated from *Xylaria*, including cytochalasins, α-pyrones, cyclopeptides, terpenoids, lactones, and succinic acid derivatives [7]. Furthermore, the secondary metabolites produced by species of *Xylaria* have been found to exert a wide range of biological activities, such as anti-inflammatory, antifungal, antibacterial, anti-tumor, and *α*-glucosidase-inhibitory activities [7,8].

**Figure 1:**
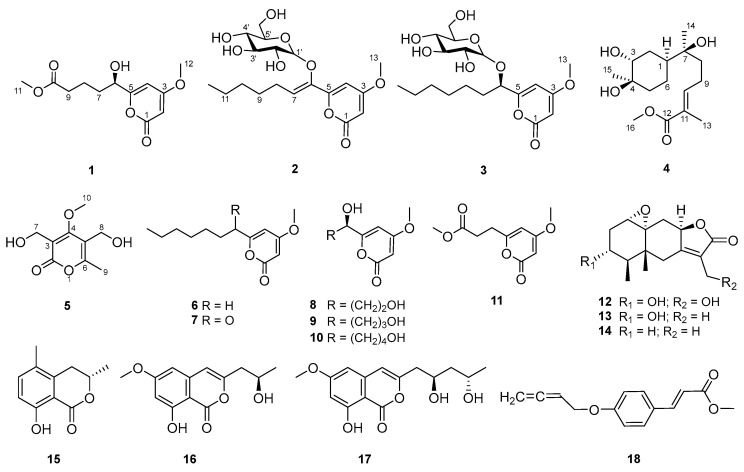
Chemical structures of compounds **1**–**18**.

As part of our group’s ongoing effort to identify bioactive natural products from medicinal plants and endophytic fungi [9,10,11], the fungus *Xylaria* sp. Z184, isolated from the leaves of *Fallopia convolvulus* (L.) Á. Löve for the first time, has attracted our attention for its impressive compound abundance in TLC and HPLC analyses (Figure S106). Our current investigation on this strain led to the isolation of three new pyranone derivatives, called fallopiaxylaresters A–C (**1**–**3**), a new bisabolane-type sesquiterpenoid fallopiaxylarol A (**4**) (Figure 1), and the first complete set of the spectroscopic data for the previously disclosed pestalotiopyrone M (**5**), as well as a suite of known compounds consisting of six pyranone derivatives (**6**–**11**), three sesquiterpenoids (**12**–**14**), three isocoumarin derivatives (**15**–**17**), and one aromatic allenic ether (**18**). Herein, the details of the isolation, structure elucidation of all new compounds and their anti-inflammatory, antimicrobial, and *α*-glucosidase-inhibitory activities were described.

## 2. Results and Discussion

**Figure 2:**
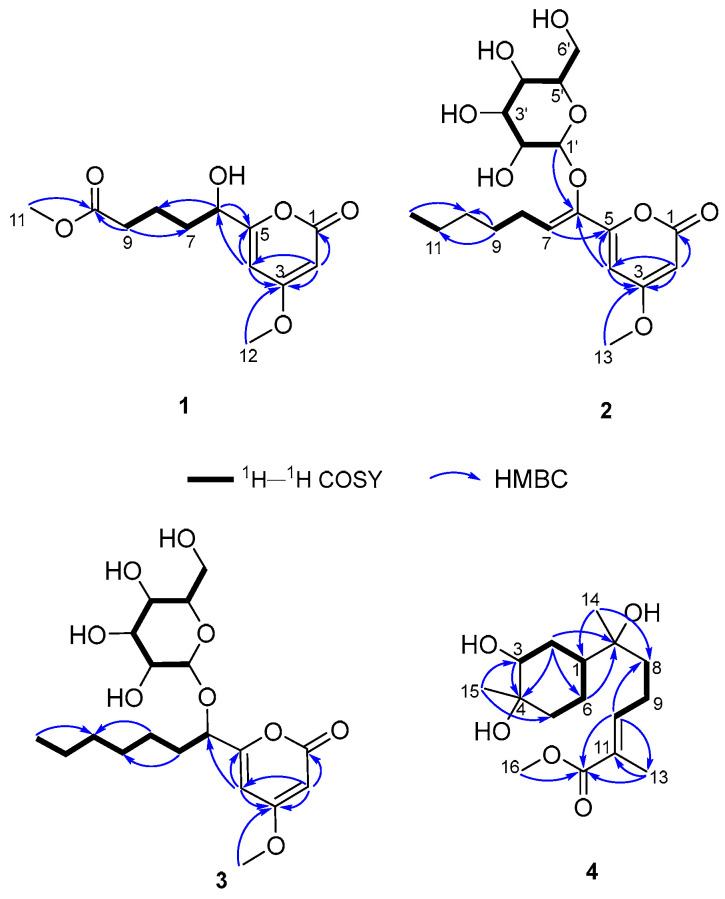
The key ^1^H–^1^H COSY and HMBC correlations of **1**–**4**.

**Figure 3:**
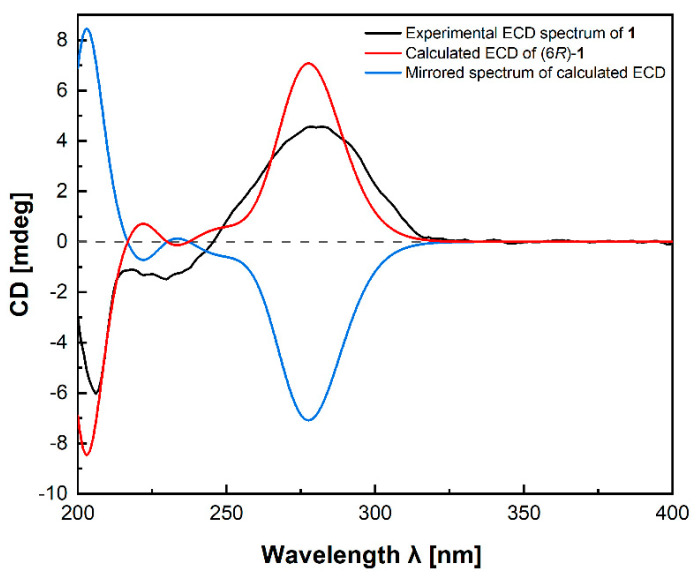
Experimental ECD spectrum of fallopiaxylarester A (**1**) (black); calculated ECD of (6*R*)–**1** (red); mirrored spectrum of calculated ECD (blue).

Compound **1**, named fallopiaxylarester A, was obtained as a white solid. Its molecular formula C_12_H_16_O_6_ was determined by the HRESIMS molecular ion peak at *m*/*z* 279.0837 [M + Na]^+^ (calcd for C_12_H_16_O_6_Na, 279.0839). The IR absorption bands showed the presence of hydroxyl (3426 cm^−1^) and carbonyl (1733 cm^−1^) functionalities. Detailed comparison of ^1^H and ^13^C NMR spectra of **1** and **10** revealed that the structure of **1** is almost identical with that of **10**, which was supported by the further analysis of 2D NMR spectra (Table 1). The HMBC correlations of **1** from H-2 (*δ*_H_ 5.56) to C-1/C-3/C-4, from H_3_-12 (*δ*_H_ 3.87) to C-3, from H-4 (*δ*_H_ 6.22) to C-3/C-5/C-6, and from H-6 (*δ*_H_ 4.35) to C-5 and C-8, showed the same settlement as with compound **10** (Figure 2). The main difference between **1** and **10** was found at C-10 of the side chain of the pyranone core, replaced by the fragment of methyl valerate group. This deduction was further identified by the HMBC correlations from H_3_-11 (*δ*_H_ 3.66) and H_2_-9 (*δ*_H_ 2.38) to C-10. Thus, the planar structure of **1** was established. Since there was only one chiral center in the molecule, the relative configuration was arbitrarily assigned as 6R*. The absolute configuration of C-6 was subsequently assigned to be *R* by comparing the optical rotational value [*α***${]}_{D}^{25}$** +56.2 (*c* 0.11, MeOH) with [*α***${]}_{D}^{25}$** +96.0 (*c* 0.10, MeOH) of compound **10** [12]. Furthermore, the deduction was also confirmed by a time-dependent density functional theory–electronic circular dichroism (TDDFT-ECD) approach. As shown in Figure 3, the Boltzmann-averaged ECD spectrum of (6*R*)-**1** displayed a similar curve compared to the experimental one. Thus, the absolute configuration at C-6 in **1** was unambiguously assigned as 6*R* (Figure 1).

Compound **2**, a white solid, was determined to possess the molecular formula of C_19_H_28_O_9_ with six degrees of unsaturation by using HRESIMS {*m*/*z* 423.1626 [M + Na]^+^, (calcd for C_19_H_28_O_9_Na, 423.1626)}. The spectra of **2** showed similar absorption bands, indicating the same presence of hydroxyl (3359 cm^−1^) and carbonyl (1696 cm^–1^) functionalities. The ^1^H NMR spectra data showed signals of a typical sugar moiety at *δ*_H_ 5.12 (d, *J* = 3.7 Hz), 3.53 (dd, *J* = 10.0, 3.7 Hz), 3.78 (t, *J* = 9.3 Hz), 3.45 (t, *J* = 9.3 Hz), 3.90 (m), and 3.76 (m) (Table 2). Analysis of the ^13^C NMR and DEPT spectra of **2** indicated the presence of 19 carbon signals, assignable to two methyl carbons (one methoxyl, *δ*_C_ 57.0 and 14.4), five methylene carbons (*δ*_C_ 27.1, 30.0, 32.8, 23.6 and 62.1), eight methine carbons (three olefinic, *δ*_C_ 89.4, 100.2, 125.0, 102.8, 73.4, 74.4, 71.0, and 75.5), one ester carbonyl carbon (*δ*_C_ 166.7), and three olefinic quaternary carbons (*δ*_C_ 174.0, 157.9 and 145.8). These substructures accounted for four out of five degrees of unsaturation, indicating one cyclic system in **2**. The ^1^H–^1^H COSY spectrum revealed three spin systems: (a) H-7/H-8/H-9; (b) H-11/H-12; and (c) H-1′/H-2′/H-3′/H-4′/H-5′/H-6′ (Figure 2). And those spin systems were connected by the key HMBC correlations from H-2 (*δ*_H_ 5.59) to C-1/C-3/C-4, from H_3_-13 (*δ*_H_ 3.87) to C-3, from H-4 (*δ*_H_ 6.95) to C-3/C-5/C-6, from H-7 (*δ*_H_ 6.07) to C-5, and from H_2_-9 (*δ*_H_ 1.48) to C-10/C-11.

**Figure 4:**
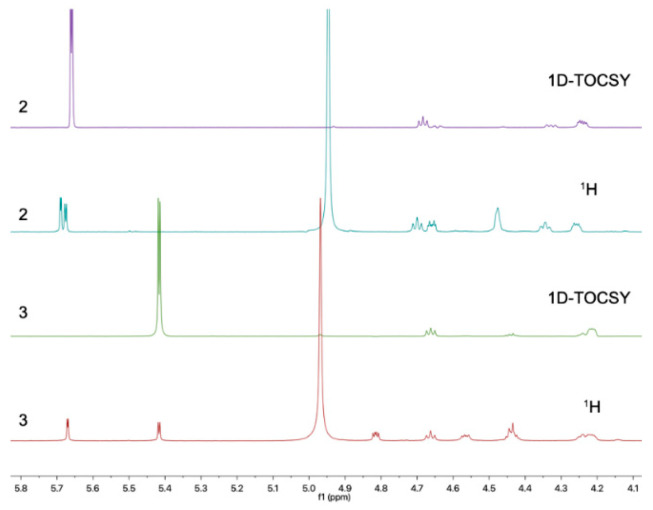
1D-TOCSY spectra of compounds **2** (purple) and **3** (green).

**Figure 5:**
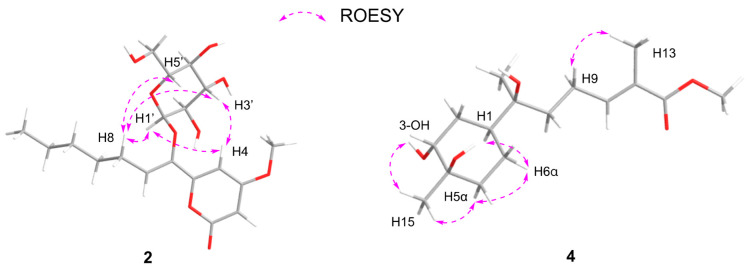
Energy-minimized structures of **2** and **4** with the key ROESY correlations.

Subsequently, the long-range HMBC correlation from H-1′ (*δ*_H_ 5.12) to the anomeric carbon C-6 suggested the sugar was attached at the C-6 of the side chain. Acid hydrolysis of **2** followed by HPLC analysis of the sugar derivative was applied to determine the type of sugar moiety, but without success. Then, a different deuterated solvent, pyridine-*d*_5_, was used to analyze the proton signals on the sugar. In addition, the isolated anomeric proton signal was probed by a selective 1D-TOCSY experiment. In the 1D-TOCSY experiment, irradiation of the signal at *δ*_H_ 5.68 (1H, d, *J* = 3.7 Hz) enabled the identification of H-2′ (*δ*_H_ 4.27, dd, *J* = 10.5, 4.1 Hz), H-3′ (*δ*_H_ 4.71, t, *J* = 9.3 Hz), H-4′ (*δ*_H_ 4.35, t, *J* = 10.0 Hz), H-5′ (*δ*_H_ 4.66, dt, *J* = 10.0, 3.5 Hz), and H_2_-6′ (*δ*_H_ 4.48, br s) in the same conjugated system (Figure 4, Table 2). The *J* values of *J*_H-5′/H-4′_ (10.0 Hz) and *J*_H-5′/H-6′_ (3.5 Hz) in ^1^H NMR in pyridine-*d*_5_ of **2** combined with the 1D-TOCSY experiment suggested an α-D-glucose. Furthermore, those chemical shift values of anomeric carbon at *δ*_C_ 102.8 (C-1′), four tertiary carbons at *δ*_C_ 73.4 (C-2′), 74.4 (C-3′), 71.0 (C-4′), 75.5 (C-5′), and a methylene oxide carbon at *δ*_C_ 62.1 (C-6′) in methanol-*d*_4_ were highly similar to 6-Buty-AA-2G along with other derivatives of AA-2G in the literature [13], which also confirmed the inference that the sugar unit was α-D-glucose. In addition, the geometry of the double bond between C-6 and C-7 was inferred by the ROESY spectrum in DMSO-*d_6_*. The ROESY correlations between H_2_-8 (*δ*_H_ 2.33) and H-1′ (*δ*_H_ 4.96)/H-3′ (*δ*_H_ 3.70)/H-5′ (*δ*_H_ 3.54), and between H-4 (*δ*_H_ 6.98) and H-1′/H-3′, demonstrated the *Z* geometry of Δ^6,7^ (Figure 5 and Figure S15). Thus, this undescribed **2** was established as shown in Figure 1 and named fallopiaxylarester B.

Compound **3**, named fallopiaxylarester C, was isolated as a white solid. Its molecular formula of C_19_H_30_O_9_ with five degrees of unsaturation was based on HRESIMS analysis {*m*/*z* 425.1780 [M + Na]^+^, (calcd for C_19_H_30_O_9_Na, 425.1782)}. Like **2**, the presence of *α*, *β*-unsaturated *γ*-lactone and hydroxyl groups in **3** was obvious by its IR absorption bands at *v*_max_ 3380 and 1700 cm^–1^. Furthermore, with the analysis of 1D NMR spectra, a sugar moiety in **3** was also quickly recognized. In addition, the HMBC correlation from H-6 (*δ*_H_ 4.52) to C-1′ suggested the same set as with **2** (Table 3). The main difference between these two compounds was the hydrogenation of the trisubstituted olefinic group at C-6/C-7 in **2**, with a 2 mass unit difference between **2** and **3**. Subsequently, an acid hydrolysis of **3** afforded the products, including a pyrone aglycone **3a** and a sugar moiety **3b**. The absolute configuration of C-6 in **3a** was assigned to be *R* form by comparing its optical value [*α***${]}_{D}^{25}$** +52.5 (*c* 0.11, MeOH) with +67.6 (*c* 0.25, MeOH) of nodulisporipyrones A [14]. According to the detailed analysis of ^1^H NMR and 1D-TOCSY experiment of **3**, H-1′ (*δ*_H_ 5.42, d, *J* = 3.8Hz), H-2′ (*δ*_H_ 4.22, dd, *J* = 9.6, 3.8 Hz), H-3′ (*δ*_H_ 4.67, t, *J* = 9.6 Hz), H-4′ (*δ*_H_ 4.24, t, *J* = 9.6 Hz), H-5′ (*δ*_H_ 4.44, t, *J* = 9.6 Hz), H_2_-6′ (*δ*_H_ 4.57, dd, *J* = 9.6, 5.6 Hz; *δ*_H_ 4.43, m), the sugar moiety was indicated as α-D-glucose (Figure 4, Table 3). Moreover, large similarities were observed by comparison of NMR data in DMSO-*d*_6_ of **3** with 5-(*α*-D-glucopyranosyloxymethyl)-2-furancarboxylic acid and other analogs in the literature [15]. Thus, the structure of **3** was established as shown (Figure 1).

Compound **4** was isolated as a colorless oil. Its molecular formula of C_16_H_28_O_5_ with three degrees of unsaturation was also based on HRESIMS analysis {*m*/*z* 323.1829 [M + Na]^+^, (calcd for C_16_H_28_O_5_Na, 323.1829)}. The IR spectrum of **4** demonstrated characteristic absorption bands for hydroxyl (3425 cm^−1^) and carbonyl (1687 cm^−1^) groups. The 1D NMR and HSQC spectra of **4** revealed 16 carbon signals, including four methyl groups, five sp^3^ methylene groups, one sp^2^ methine, two sp^3^ methine groups, and four quaternary carbons (three oxygenated carbons) (Table 4). The above information accounted for two degrees of unsaturation, indicating one cyclic system in compound**4**. The ^1^H–^1^H COSY spectrum revealed two spin systems: (a) H-5/H-6/H-1/H-2/H-3, and (b) H-8/H-9/H-10 (Figure 2). Furthermore, the HMBC correlations from H_3_-15 (*δ*_H_ 1.04) to C-3/C-5, from H_2_-2 (*δ*_H_ 1.59, 1.53)/H-3 (*δ*_H_ 3.36) to C-4, from H_2_-2 (*δ*_H_ 1.59, 1.53)/H_2_-6 (*δ*_H_ 1.29) to C-7, from H_3_-14 (*δ*_H_ 0.96) to C-1/C-8, from H-10 (*δ*_H_ 6.71) to C-8/C-12/C-13, from H_3_-13 (*δ*_H_ 1.77) to C-11/C-12, and from H_3_-16 (*δ*_H_ 3.64) to C-12 made those two spin systems connected. Thus, the planar structure of **4** was established as shown (Figure 1) and named fallopiaxylarol A.

Initially, the ROESY correlation between H_3_-13 and H_2_-9 and the lack of correlation of H_3_-13/H-10 assigned the *E*-geometry of Δ^10, 11^, which was also supported by the *J* value of H-10 (7.5) (Figure 5). In addition, the ROESY correlations of H-1/3-OH/H_3_-15/H-5α/H-6α suggested the *cis* orientation of the H-1, 3-OH, and H_3_-15. Furthermore, the literature survey revealed that the NMR data of the six-membered ring and the optical rotation values of **4** were almost identical to those of (1*S*,3*R*,4*R*,7*S*)-3,4-dihydroxy-*α*-bisabolol [16]. Thus, the absolute configuration of **4** was tentatively determined as shown in Figure 1.

After the literature survey, as for the secondary metabolites produced by the genus *Xylaria*, the main structural differences between the co-isolated new pyranone derivatives in this case and the other analogues of the genus are the variation of substituents in the side chain attached pyranone core [7,12,16]. Although compound **5** has previously been reported as a natural product from fermentation extracts of endophytic fungi [17], this is the first report of its existence to be accompanied by a full suite of supporting spectroscopic data. The 14 known compounds, pestalotiopyrone M (**5**), 4-methoxy-6-nonyl-2-pyrone (**6**) [18], xylariaopyrone A (**7**) [19], xylariaopyrone H (**8**) [12], xylariaopyrone I (**9**) [12], xylapyrone D (**10**) [20], scirpyrone H (**11**) [21], 1*α*,10*α*-epoxy-3*α*,13-dihydroxyeremophil-7(11)-en-12,8*β*-olide (**12**) [4], 3*α*-hydroxymairetolide A (**13**) [4], mairetolide A (**14**) [22], (−)-5-methylmellein (**15**) [23], diaporthin (**16**) [24], mucorisocoumarin B (**17**) [25], and eucalyptene (**18**) [26], were also isolated from *Xylaria* sp. Z184. The structures of these compounds (**5**–**18**) were identified by comparing the spectral data to those reported in the respective references (Figures S80–S105).

The secondary metabolites generated by stains of *Xylaria* usually show obvious anti-inflammatory and antifungal activities [7,8]. In this case, compounds **2**–**10** and **15**–**18** and the crude extract were selected to evaluate the antimicrobial, anti-inflammatory and α-glucosidase-inhibition activities due to the limitation of samples. In antimicrobial assay, compounds **5**, **7**, and **8** displayed weak activity against *Staphylococcus areus* subsp. *aureus* with inhibition ratios of 25.9%, 31.5%, and 25.3% at a concentration of 100 μM. Unfortunately, in anti-inflammatory and α-glucosidase assay, only the crude extract potently inhibited LPS-induced NO production in RAW264.7 mouse macrophages, with an inhibition rate of 77.28 ± 0.82% at a concentration of 50 μg/mL. Although it was cytotoxic at this concentration, reducing the concentration to 6.25 μg/mL abrogated the cytotoxicity (Table 5).

## 3. Materials and Methods

### 3.1. General Experimental Procedures

Optical rotations were determined with a PerkinElmer 341 polarimeter (PerkinElmer, Waltham, MA, USA). UV absorptions were obtained by using a Waters UV-2401A spectrophotometer equipped with a DAD and a 1 cm path length cell. Methanolic samples were scanned from 190 to 400 nm in 1 nm steps. Measurements of IR spectra were performed using a Bruker Vertex 70 FT-IR spectrometer (Bruker, Karlsruhe, Germany). NMR spectra were recorded on Bruker AM-400 and AM-600 NMR spectrometers (Bruker, Karlsruhe, Germany) with TMS as internal standard, and NMR data were referenced to selected chemical shifts of methanol-*d*_4_ (^1^H: 3.31 ppm, ^13^C: 49.0 ppm), chloroform-*d* (^1^H: 7.26 ppm, ^13^C: 77.0 ppm), and dimethyl sulfoxide-*d*_6_ (^1^H: 2.50 ppm, ^13^C: 39.5 ppm), respectively. HRESIMS data were acquired on a Thermo Fisher LTQ XL LC/MS (Thermo Fisher, Palo Alto, CA, USA). Semi-preparative HPLC was performed on an Agilent 1220 instrument equipped with a UV detector with a semi-preparative column (RP-C_18_, 5 μm, 250 × 10 mm, Welch Materials, Inc., Shanghai, China). Column chromatography was performed using Sephadex^TM^ LH-20 gel (40–70 μm; Merck KGaA, Darmstadt, Germany), and precoated silica gel plates (GF254, Qingdao Marine Chemical Co., Ltd., Qingdao, China) were used for TLC analyses. Spots were visualized by heating silica gel plates sprayed with 10% H_2_SO_4_ in EtOH. All HPLC solvents were purchased from Guangdong Guanghua Sci-Tech Co., Ltd. (Guangzhou, China). All solvents were of analytical grade (Guangzhou Chemical Regents Company, Ltd., Guangzhou, China).

### 3.2. Fungal Material

**Figure 6:**
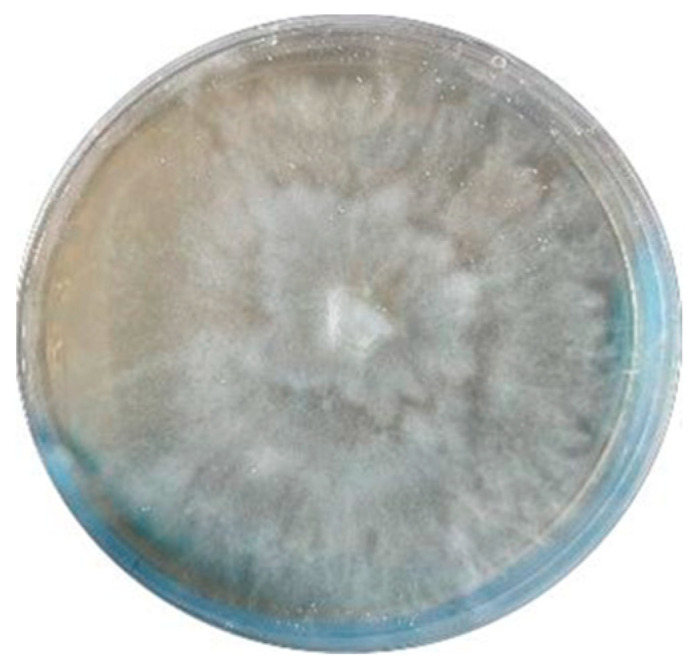
Photo of the fungus *Xylaria* sp. Z184.

The fungus *Xylaria* sp. Z184 was isolated from the leaves of *Fallopia convolvulus* (L.) Á. Löve collected in Zhuyang Town, Henan province, P. R. China (N 34°14′12″ W 110°47′09″) in June 2022. Leaves of *F*. *convolvulus* (L.) Á. Löve were processed within 24 h and rinsed with sterile water. On a sterile workbench, after 30 min of ultraviolet light exposure, the leaves underwent sequential treatment with a 5% sodium hypochlorite solution, sterile water, and 75% ethanol, either soaked or rinsed, followed by drying with sterile filter paper. Leaves were trimmed into small squares with sterile scissors and placed into previously prepared PDA monoclonal agar plates, inoculating three petri plates in parallel. These plates were incubated at 30 °C for 3–7 days, until mycelial growth was observed extending from the inside of the tissue block to its surroundings. Distinct morphological colonies were subsequently transferred to new media for continued cultivation. This procedure was repeated until the fungal strains showed uniform growth, leading to the isolation of purified strains (Figure 6).

To identify the strains, the standardized operating procedure was performed, which included genomic DNA extraction, 16*S*/18*S* amplification, PCR product detection and purification, and comparison of sequencing results with the NCBI-BLAST database (https://www.ncbi.nlm.nih.gov/) accessed on 10 December 2022, using ITS1 and ITS4 primers for both amplification and sequencing. The sequence data for this strain was submitted to the GenBank under accession No. KU645984. The fungal strain was deposited on 20% aqueous glycerol stock in a −80 °C freezer at the School of Pharmaceutical Sciences (Shenzhen), Shenzhen Campus of Sun Yat-sen University, Shenzhen, China.

### 3.3. Fermentation, Extraction, and Isolation

*Xylaria* sp. Z184 was cultured on potato dextrose agar for 5 days at 28 °C to prepare the seed culture. The cultured agar plates were cut into small pieces, which were then inoculated into 30 previously autoclaved Erlenmeyer flasks (350 mL), each containing 50 g of rice and 45 mL of distilled water. All flasks were incubated at 28 °C for 40 days. Cultural media was extracted with methanol four times, and the solvent was evaporated under reduced pressure at 45 °C. Then the extract was suspended in water and extracted four times with ethyl acetate. The combined ethyl acetate layers were concentrated under reduced pressure to yield a brown extract (7.5 g).

The crude extract was chromatographed on Sephadex LH-20 (MeOH) to give eight fractions (Fr.1–Fr.8). Fr. 3 (2.8 g) was separated with silica gel column chromatography (CC) with petroleum ether (PE)/EtOAc (20:1–0:1, *v*/*v*) to give seven subfractions (Fr. 3.1–Fr. 3.7). Fr. 3.3 (306.2 mg) was purified with silica gel CC using PE/EtOAc (15:1–1:1, *v*/*v*) to yield six further subfractions (Fr. 3.3.1–Fr. 3.3.6). Fr. 3.3.4 (25.8 mg) was purified by semi-preparative HPLC (MeOH/H_2_O, 48:52, *v*/*v*, 3.0 mL/min) to yield compound **7** (4.3 mg, *t*_R_ 22.0 min). Fr. 4 (1.7 g) was separated by silica gel CC with CH_2_Cl_2_/MeOH (80:1–0:1, *v*/*v*) to obtain seven subfractions (Fr. 4.1–Fr. 4.7). Fr. 4.3 (277.5 mg) was purified by semi-preparative HPLC (MeCN/H_2_O, 25:75, 0–14 min, then MeCN/H_2_O, 50:50, 14.01–33 min, *v*/*v*, 3.0 mL/min) to yield compounds **13** (4.6 mg, *t*_R_ 12.1 min), **14** (1.6 mg, *t*_R_ 27.6 min), and **6** (4.1 mg, *t*_R_ 31.5 min). Fr. 4.5 (239.9 mg) was purified by semi-preparative HPLC (MeCN/H_2_O, 20:80, 0–10 min, then MeCN/H_2_O, 40:60, 10.01–21 min, *v*/*v*, 3.0 mL/min) to yield compounds **10** (8.7 mg, *t*_R_ 13.2 min) and **4** (6.0 mg, *t*_R_ 19.5 min). Fr. 4.6 (225.9 mg) was purified by semi-preparative HPLC (MeOH/H_2_O, 40:60, *v*/*v*, 3.0 mL/min) to yield compounds **2** (3.9 mg, *t*_R_ 30.1 min) and **3** (12.1 mg, *t*_R_ 35.5 min). Fr. 5 (1.3 g) was separated with silica gel CC with CH_2_Cl_2_/MeOH (25:1–0:1, *v*/*v*) to give six subfractions (Fr. 5.1—Fr. 5.6). Fr. 5.1 (44.0 mg) was further purified by semi-preparative HPLC (MeOH/H_2_O, 55:45, 0–23 min, then MeOH/H_2_O, 65:35, 23.01–47 min, *v*/*v*, 3.0 mL/min) to yield compounds **15** (3.1 mg, *t*_R_ 21.8 min) and **18** (1.4 mg, *t*_R_ 45.5 min). Fr. 5.3 (399.7 mg) was purified by semi-preparative HPLC (MeOH/H_2_O, 35:65, 0–19 min, then MeOH/H_2_O, 54:46, 19.01–40 min, *v*/*v*, 3.0 mL/min) to yield compounds **1** (1.2 mg, *t*_R_ 6.0 min), **17** (8.3 mg, *t*_R_ 18.1 min), **16** (2.7 mg, *t*_R_ 31.9 min), and **11** (2.4 mg, *t*_R_ 38.2 min). Similarly, Fr. 5.4 (355.8 mg) was purified by semi-preparative HPLC (MeCN/H_2_O, 10:90, *v*/*v*, 3.0 mL/min) to yield compounds **5** (3.5 mg, *t*_R_ 6.5 min), **8** (1.3 mg, *t*_R_ 8.1 min), **9** (8.3 mg, *t*_R_ 10.9 min), and **12** (2.1 mg, *t*_R_ 15.5 min).

### 3.4. Spectral and Physical Data of Compounds ***1***–***5***

Fallopiaxylarester A (**1**): White solid; [*α***${]}_{D}^{25}$** +56.2 (*c* 0.11, MeOH); UV (MeOH) *λ*_max_ (log *ε*): 279 (0.19), 204 (0.72) nm; IR (KBr) *v*_max_: 3426, 2922, 1733, 1648, 1569, 1457, 1412, 1384, 1247, 1032, 832 cm^–1^; ECD (MeOH) *λ*_max_ (Δε): 279 (+3.0), 206 (–4.0) nm. ^1^H and ^13^C NMR data, see Table 1; HRESIMS (*m*/*z*): 279.0837 [M + Na]^+^ (calcd for C_12_H_16_O_6_Na, 279.0839).

Fallopiaxylarester B (**2**): White solid; [*α***${]}_{D}^{25}$** +102.9 (*c* 0.38, MeOH); UV (MeOH) *λ*_max_ (log *ε*): 310 (0.16), 260 (0.06), 219 (0.41) nm; IR (KBr) *v*_max_: 3359, 2928, 2858, 1696, 1623, 1560, 1456, 1409, 1260, 1230, 1080, 1018, 817, 539 cm^–1^; ECD (MeOH) *λ*_max_ (Δε): 310 (+3.4), 231 (–4.1), 205 (–3.3) nm. ^1^H and ^13^C NMR data, see Table 2; HRESIMS (*m*/*z*): 423.1626 [M + Na]^+^ (calcd for C_19_H_28_O_9_Na, 423.1626).

Fallopiaxylarester C (**3**): White solid; [*α***${]}_{D}^{25}$** +124.2 (*c* 0.39, MeOH); UV (MeOH) *λ*_max_ (log *ε*): 281 (0.11), 204 (0.45) nm; IR (KBr) *v*_max_: 3380, 2927, 2857, 1700, 1649, 1569, 1458, 1414, 1384, 1250, 1025, 836, 700 cm^–1^; ECD (MeOH) *λ*_max_ (Δε): 280 (+4.5), 232 (+0.9), 205 (+3.0) nm; ^1^H and ^13^C NMR data, see Table 3; HRESIMS (*m*/*z*): 425.1780 [M + Na]^+^ (calcd for C_19_H_30_O_9_Na, 425.1782).

Fallopiaxylarol A (**4**): Colorless oil; [*α***${]}_{D}^{25}$** –31.8 (*c* 0.10, MeOH); UV (MeOH) *λ*_max_ (log *ε*): 205 (0.36), 218 (0.48) nm; IR (KBr) *v*_max_: 3546, 3426, 2945, 2930, 1688, 1287, 1150, 1036 cm^−1^; ^1^H and ^13^C NMR data, see Table 4; HRESIMS (*m*/*z*): 323.1829 [M + Na]^+^ (calcd for C_16_H_28_O_5_Na, 323.1829).

Pestalotiopyrone M (**5**): White solid; UV (MeOH) *λ*_max_ nm (log *ε*) 206 (0.45), 293 (0.16); IR (KBr) *ν*_max_ 3311, 2961, 2928, 1711, 1565, 1365, 1014, 989 cm^–1^; ^1^H NMR (methanol-*d*_4_, 600 MHz) *δ*_H_: 4.54 (2H, s, H-7), 4.41 (2H, s, H-8), 4.18 (3H, s, H-10), 2.35 (3H, s, H-9); ^13^C NMR (methanol-*d*_4,_ 150 MHz) *δ*_C_: 171.6 (C, C-4), 167.4 (C, C-2), 163.0 (C, C-6), 115.2 (C, C-5), 110.1 (C, C-3), 63.1 (CH_3_, C-10), 55.5 (CH_2_, C-8), 55.1 (CH_2_, C-7), 17.3 (CH_3_, C-9); HRESIMS (*m*/*z*): 223.0578 [M + Na]^+^ (calcd for C_9_H_12_O_5_Na, 223.0577).

### 3.5. Computational Details (TDDFT-ECD) of ***1***

The conformational search of (6*R*)-**1** was performed by using torsional sampling (MCMM) conformational searches with an OPLS_2005 force field within an energy window of 21 kJ/mol. Conformers above 1% Boltzmann populations were re-optimized at the B3LYP/6-31G(d) level with the IEFPCM solvent model for methanol. The following TDDFT calculations of the re-optimized geometries were all performed at the B3LYP/6-311G(d,p) level with the IEFPCM solvent model for methanol. Frequency analysis was performed as well to confirm that the re-optimized geometries were at the energy minima. Finally, the SpecDis 1.62 [27] software was used to obtain the Boltzmann-averaged ECD spectra of **1** and visualize the result.

### 3.6. Biological Assays

#### 3.6.1. Antimicrobial Activity

Compounds **2**–**10** and **15**–**18**, and the crude extract were evaluated for antimicrobial activities against *Staphylococcus aureus* subsp. *aureus* and fluconazole-resistant *Candida albicans*. The antimicrobial assay was conducted according to a previously described method [28]. Samples were added into a 96-well culture plate with a maximum test compound concentration of 100 μM. Bacterial liquid was added to each well until the final concentration was 5 × 10^5^ CFU/mL. The plate was then incubated at 37 °C for 24 h, and the OD values at 595 nm were measured using a microplate reader. Blank bacterial medium served as control.

#### 3.6.2. Anti-Inflammatory Activity

The RAW 264.7 cells (2 × 10^5^ cells/well) were incubated in 96-well culture plates with or without 1 µg/mL lipopolysaccharide (LPS, Sigma Chemical Co., St. Louis, MO, USA) for 24 h in the presence or absence of the test compounds. Supernatant aliquots (50 µL) were then treated with 100 µL Griess reagent (Sigma Chemical Co., St. Louis, MO, USA). The absorbance was measured at 570 nm by using a Synergy TMHT microplate reader (BioTek Instruments Inc., Winooski, VT, USA). In this study, N^G^-methyl-L-arginine acetate (L-NMMA, Sigma Chemical Co., USA) was used as a positive control. In the remaining medium, an MTT assay was carried out to determine whether the suppressive effect was related to cell viability. The inhibitory rate of nitric oxide (NO) production = (NO level of blank control − NO level of test samples)/NO level of blank control. The percentage of NO production was evaluated by measuring the amount of nitrite concentration in the supernatants with Griess reagent, as described previously [29].

#### 3.6.3. Alpha-Glucosidase-Inhibition Activity

The α-glucosidase inhibition was assessed according to the slightly modified method of Ma et al. [30]. All samples were dissolved in DMSO at a concentration of 50 μM. The α-glucosidase (Sigma Chemical Co., St. Louis, MO, USA) and substrate (4–Nitrophenyl α-D-glucopyranoside, PNPG, Sigma Chemical Co., St. Louis, MO, USA) were dissolved in potassium phosphate buffer (0.1 M, pH 6.7). The samples were preincubated with α-glucosidase at 37 °C for 10 min. Then, PNPG was quickly added to the 96-well enzyme label plate to start the reaction, and the plate was incubated at 37 °C for 50 min. At the same time, a blank control without samples and a positive control of quercetin (10 mM) was set up. All samples were thoroughly mixed and analyzed in triplicate. The OD value was measured at 405 nm using a microplate reader. The inhibition percentage (%) was calculated by the following equation: Inhibition (%) = (1 − OD _sample_)/OD _control blank_ × 100.

## 4. Conclusions

In this paper, three new pyranone derivatives (**1**–**3**) and a new bisabolane-type sesquiterpenoid (**4**) were discovered from the fungus *Xylaria* sp. Z184. Moreover, we co-isolated 14 previously reported compounds (**5**–**18**), and reported the first complete set of spectroscopic data for pyranone **5**. In vitro bioassays were performed on a number of the isolated compounds and crude fungal extract. Compounds **5**, **7**, and **8** were demonstrated to be weak growth inhibitors of *Staphylococcus aureus* subsp. *Aureus*, and the extract was shown to be a potent inhibitor of NO production in LPS-stimulated RAW 264.7 mouse macrophages, with an inhibition rate of 77.28 ± 0.82% at 50 μg/mL. Although the crude fungal extract showed certain inhibitory activity of NO production in LPS-stimulated RAW 264.7 mouse macrophages, unfortunately, in the subsequent isolated compounds, no such convenient activity was found. This suggests that there may still be other structural types of compounds in the extract that exhibit anti-inflammatory activity. Thus, in addition to revealing four novel compounds, this work enhances understanding of the structural diversity within the *Xylaria* metabolomes.

## Figures and Tables

**Table 1:** ^1^H NMR (*δ*_H_, 600 MHz) and ^13^C NMR (*δ*_C_, 150 MHz) data for **1** in methanol-*d*_4_.

No.	*δ*_H_, Mult (*J* Hz)	*δ*_C_, Type
1		167.0, C
2	5.56, d (2.1)	88.6, CH
3		173.8, C
4	6.22, d (2.1)	99.9, CH
5		168.7, C
6	4.35, dd (7.2, 4.8)	70.7, CH
7	1.83, m	35.2, CH_2_
	1.68, m, overlap	
8	1.76, m	21.7, CH_2_
	1.69, m, overlap	
9	2.38, t (6.7)	34.4, CH_2_
10		175.6, C
11	3.66, s	52.0, CH_3_
12	3.87, s	57.0, CH_3_

**Table 2:** ^1^H NMR (*δ*_H_, 600 MHz) and ^13^C NMR (*δ*_C_, 150 MHz) data for **2**.

No.	*δ*_H_, Mult (*J* Hz) ^a^	*δ*_C_, Type ^a^	*δ*_H_, Mult (*J* Hz) ^b^	*δ*_C_, Type ^b^	*δ*_H_, Mult (*J* Hz) ^c^	*δ*_C_, Type ^c^
1		166.7, C		164.1, C		162.7, C
2	5.59, d (2.2)	89.4, CH	5.70, d (2.2)	89.7, CH	5.61, d (2.2)	88.6, CH
3		174.0, C		172.3, C		171.2, C
4	6.95, d (2.2)	100.2, CH	7.64, d (2.2)	99.7, CH	6.98, d (2.2)	98.3, CH
5		157.9, C		157.5 C		155.8, C
6		145.8, C		146.1, CH		144.3, C
7	6.07, t (7.5)	125.0, CH	6.24, t (7.5)	123.6, CH	5.91, t (7.5)	122.8, CH
8	2.42, m	27.1, CH_2_	2.61, q (7.5)	26.9, CH_2_	2.33, dd (15.0, 7.6)	25.3, CH_2_
9	1.48, m	30.0, CH_2_	1.33, m	29.7, CH_2_	1.39, m	28.4, CH_2_
10	1.36, m, overlap	32.8, CH_2_	1.19, m, overlap	32.3, CH_2_	1.28, m, overlap	31.1, CH_2_
11	1.37, m, overlap	23.6, CH_2_	1.18, m, overlap	23.2, CH_2_	1.29, m, overlap	22.0, CH_2_
12	0.92, t (6.9)	14.4, CH_3_	0.75, t (7.0)	14.6, CH_3_	0.87, t (6.9)	13.9, CH_3_
13	3.87, s	57.0, CH_3_	3.60, s	56.5, CH_3_	3.81, s	56.4, CH_3_
1′	5.12, d (3.7)	102.8, CH	5.68, d (3.7)	103.4, CH	4.96, d (3.7)	101.3, CH
2′	3.53, dd (10.0, 3.7)	73.4, CH	4.27, dd (10.5, 4.1)	74.0, CH	3.32, m, overlap	71.7, CH
3′	3.78, t (9.3)	74.4, CH	4.71, t (9.3)	75.2, CH	3.70, m	72.5, CH
4′	3.45, t (9.3)	71.0, CH	4.35, t (10.0)	71.9, CH	3.22, m	69.4, CH
5′	3.90, m	75.5, CH	4.66, dt (10.0, 3.5)	76.8, CH	3.54, m, overlap	74.7, CH
6′	3.76, m	62.1, CH_2_	4.48, br s	62.9, CH_2_	3.55, m, overlap	60.3, CH_2_
2′-OH					5.52, d (4.9)	
3′-OH					5.10, m	
4′-OH					5.10, m	
6′-OH					4.53, t (5.8)	

^a^ Measured in methanol-*d*_4_, ^b^ measured in pyridine-*d*_5_, ^c^ measured in DMSO-*d*_6_.

**Table 3:** ^1^H NMR (*δ*_H_, 600 MHz) and ^13^C NMR (*δ*_C_, 150 MHz) data for **3**.

No.	*δ*_H_, Mult (*J* Hz) ^a^	*δ*_C_, Type ^a^	*δ*_H_, Mult (*J* Hz) ^b^	*δ*_C_, Type ^b^	*δ*_H_, Mult (*J* Hz) ^c^	*δ*_C_, Type ^c^
1		167.1, C		164.5, C		164.2, C
2	5.57, d (2.2)	89.1, CH	5.68, d (2.3)	89.1, CH	5.57, d (2.2)	88.0, CH
3		173.6, C		171.9, C		170.9, C
4	6.55, d (2.2)	101.9, CH	6.99, d (2.3)	100.5, CH	6.50, d (2.2)	99.4, CH
5		165.5, C		165.8, C		163.3, C
6	4.52, t (6.2)	75.3, CH	4.82, dd (7.8, 4.4)	74.8, CH	4.38, dd (6.8, 4.8)	72.9, CH
7	1.85, dd (14.0, 7.6)	35.1, CH_2_	1.89, m	35.2, CH_2_	1.70, m	33.5, CH_2_
			1.84, m			
8	1.43, m	26.2, CH_2_	1.51, m	26.2, CH_2_	1.32, m	24.4, CH_2_
9	1.35, m	30.1, CH_2_	1.18, m, overlap	29.7, CH_2_	1.27, m, overlap	28.4, CH_2_
	1.33, m		1.10, m, overlap			
10	1.31, m, overlap	32.8, CH_2_	1.08, m, overlap	32.2, CH_2_	1.23, m, overlap	31.1, CH_2_
11	1.32, m, overlap	23.7, CH_2_	1.16, m, overlap	23.3, CH_2_	1.25, m, overlap	22.0, CH_2_
12	0.90, t (6.8)	14.4, CH_3_	0.78, t (7.3)	14.7, CH_3_	0.85, t (6.8)	14.0, CH_3_
13	3.87, s	57.0, CH_3_	3.63, s	56.4, CH_3_	3.81, s	56.4, CH_3_
1′	4.80, d (3.8)	98.5, CH	5.42, d (3.8)	99.3, CH	4.66, d (3.8)	97.2, CH
2′	3.41, dd (9.8, 3.8)	73.2, CH	4.22, dd (9.6, 3.8)	74.0, CH	3.22, m	71.5, CH
3′	3.68, m, overlap	74.8, CH	4.67, t (9.6)	75.6, CH	3.45, m, overlap	73.7, CH
4′	3.29, m	71.7, CH	4.24, t (9.6)	72.6, CH	3.07, m	70.1, CH
5′	3.68, m, overlap	74.6, CH	4.44, t (9.6)	75.8, CH	3.45, m, overlap	73.0, CH
6′	3.68, m, overlap	62.7, CH_2_	4.57, dd (9.6, 5.6)	63.3, CH_2_	3.61, m	60.9, CH_2_
	3.83, m		4.43, m		3.45, m, overlap	
2′-OH					5.04, d (5.9)	
3′-OH					4.92, d (4.1)	
4′-OH					4.99, d (5.2)	
6′-OH					4.52, t (5.4)	

^a^ Measured in methanol-*d*_4_, ^b^ measured in pyridine-*d*_5_, ^c^ measured in DMSO-*d*_6_.

**Table 4:** ^1^H NMR (*δ*_H_, 600 MHz) and ^13^C NMR (*δ*_C_, 150 MHz) data for **4**.

No.	*δ*_H_, Mult (*J* Hz) ^a^	*δ*_C_, Type ^a^	*δ*_H_, Mult (*J* Hz) ^b^	*δ*_C_, Type ^b^
1	1.72, m	39.1, CH	1.59, m, overlap	38.8, CH
2	1.80, m	29.3, CH_2_	1.59, m, overlap	29.2, CH_2_
			1.53 m	
3	3.66, br s	74.0, CH	3.36, m, overlap	72.6, CH
4		70.9, C		69.5, C
5	1.74, m	33.6, CH_2_	1.49, m	33.5, CH_2_
	1.55, m		1.29, m, overlap	
6	1.49, m	22.1, CH_2_	1.29, m, overlap	21.7, CH_2_
	1.40, m			
7		74.0, C		72.0, C
8	1.60, m	38.8, CH_2_	1.41, m	38.1, CH_2_
9	2.26, m	22.9, CH_2_	2.18, q (8.0)	22.7, CH_2_
10	6.77, td (7.5, 1.2)	142.6, CH	6.71, td (7.5, 0.9)	143.5, CH
11		127.6, C		126.4, C
12		168.8, C	0.90, t (6.8)	167.7, C
13	1.84, s	12.4, CH_3_	1.77, s	12.2, CH_3_
14	1.14, s	23.7, CH_3_	0.96, s	23.8, CH_3_
15	1.26, s	27.6, CH_3_	1.04, s	27.9, CH_3_
16	3.73, s	51.8, CH_3_	3.64, s	51.6, CH_3_
3-OH			4.36, d (4.0)	
4-OH			3.96, s	
7-OH			3.88, s	

^a^ Measured in chloroform-*d*, ^b^ measured in DMSO-*d*_6_.

**Table 5:** Inhibitory activities of compounds selected and crude extract on LPS-stimulated NO production.

Compounds	Concentration	NO Production Inhibition (%) ^a^
**2**	50 μM	4.51 ± 0.35
**3**	50 μM	–3.93 ± 2.43
**4**	50 μM	1.85 ± 3.18
**5**	50 μM	–1.61 ± 0.53
**12**	50 μM	0.92 ± 2.97
**13**	50 μM	4.67 ± 2.36
**14**	50 μM	3.54 ± 1.26
**18**	50 μM	–0.92 ± 2.21
**Crude extract**	50 μg/mL	77.28 ± 0.82
	6.25 μg/mL	7.78 ± 3.29
**L-NMMA ^b^**	50 μM	53.75 ± 1.28

^a^ All compounds examined in a set of triplicated experiment. ^b^ Positive control.

## Data Availability

The authors declare that all relevant data supporting the results of this study are available within in the article and its Supplementary Materials file.

## Supplementary Materials

The following supporting information can be downloaded at: https://www.mdpi.com/article/10.3390/molecules29081728/s1, Figures S1–S79: 1D, 2D NMR spectra, and HRESIMS of compounds **1**–**5**; Figures S80-S105: ^1^H and ^13^C NMR spectra of compounds **6**–**18**; Figure S106: The TLC and HPLC profiles of the crude extract of fungus *Xylaria* sp. Z184.
