# Supplementary material for: New Pyranone Derivatives and Sesquiterpenoid Isolated from the Endophytic Fungus Xylaria sp. Z184
Source: Molecules. 2024 Apr 11;29(8):1728. doi: 10.3390/molecules29081728 (PMC11051921; doi:10.3390/molecules29081728)
Supplement: Supplementary file 1 [file molecules-29-01728-s001.zip › molecules-2945204-supplementary.pdf]

Supplementary Material

New pyranone derivatives and sesquiterpenoid isolated from the endophytic fungus *Xylaria* sp. Z184

Yan Zhang[a, †], Yang Jin[a, †], Wensi Yan[a], Peishan Gu[a], Ziqian Zeng[a], Ziying Li[b], Guangtao Zhang[b], Mi Wei[c], Yongbo Xue[a,*]

Affiliations:

[a]*School of Pharmaceutical Sciences (Shenzhen), Shenzhen Campus of Sun Yat-sen University, Shenzhen, Guangdong 518107, P. R. China*

[b]*School of Pharmacy, Binzhou Medical University*

[c]*School of Agriculture, Shenzhen Campus of Sun Yat-sen University, Shenzhen, Guangdong 518107, P. R. China*

[†]Yan Zhang and Yang Jin contributed equally to this work.

E-mail: Yongbo Xue*- xueyb@mail.sysu.edu.cn

Telephone number: 13660160614

ORCID: http://orcid.org/0000-0001-9133-6439

* Corresponding author

Contents

Fig. S1 ^{1}H NMR spectrum of **1** in methanol-d_4 (600 MHz). 6
Fig. S2 ^{13}C NMR spectrum of **1** in methanol-d_4 (150 MHz). 7
Fig. S3 HSQC spectrum of **1** in methanol-d_4 (600 MHz). 8
Fig. S4 HMBC spectrum of **1** in methanol-d_4 (600 MHz). 9
Fig. S5 ^{1}H–^{1}H COSY spectrum of **1** in methanol-d_4 (600 MHz). 10
Fig. S6 ROESY spectrum of **1** in methanol-d_4 (600 MHz). 11
Fig. S7 HRESIMS spectrum of **1**. 12
Fig. S8 IR spectrum of **1**. 13
Fig. S9 UV spectrum of **1**. 14
Fig. S10 ^{1}H NMR spectrum of **2** in DMSO-d_6 (600 MHz). 15
Fig. S11 ^{13}C NMR spectrum of **2** in DMSO-d_6 (150 MHz). 16
Fig. S12 HSQC spectrum of **2** in DMSO-d_6 (600 MHz). 17
Fig. S13 HMBC spectrum of **2** in DMSO-d_6 (600 MHz). 18
Fig. S14 ^{1}H-^{1}H COSY spectrum of **2** in DMSO-d_6 (600 MHz). 19
Fig. S15 ROESY spectrum of **2** in DMSO-d_6 (600 MHz). 20
Fig. S16 ^{1}H NMR spectrum of **2** in methanol-d_4 (600 MHz). 21
Fig. S17 ^{13}C NMR spectrum of **2** in methanol-d_4 (150 MHz). 22
Fig. S18 HSQC spectrum of **2** in methanol-d_4 (600 MHz). 23
Fig. S19 HMBC spectrum of **2** in methanol-d_4 (600 MHz). 24
Fig. S20 ^{1}H–^{1}H COSY spectrum of **2** in methanol-d_4 (600 MHz). 25
Fig. S21 ROESY spectrum of **2** in methanol-d_4 (600 MHz). 26
Fig. S22 ^{1}H NMR spectrum of **2** in pyridine-d_5 (600 MHz). 27
Fig. S23 1D-TOCSY spectrum of **2** in pyridine-d_5 (800 MHz). 28
Fig. S24 ^{13}C NMR spectrum of **2** in pyridine-d_5 (150 MHz). 29
Fig. S25 HSQC spectrum of **2** in pyridine-d_5 (600 MHz). 30
Fig. S26 HMBC spectrum of **2** in pyridine-d_5 (600 MHz). 31
Fig. S27 ^{1}H–^{1}H COSY spectrum of **2** in pyridine-d_5 (600 MHz). 32
Fig. S28 HRESIMS spectrum of **2**. 33
Fig. S29 IR spectrum of **2**. 34
Fig. S30 UV spectrum of **2**. 35

Fig. S31 ^{1}H NMR spectrum of **3** in DMSO-d_6 (600 MHz).........36
Fig. S32 ^{13}C NMR spectrum of **3** in DMSO-d_6 (600 MHz).........37
Fig. S33 HSQC spectrum of **3** in DMSO-d_6 (600 MHz).........38
Fig. S34 HMBC spectrum of **3** in DMSO-d_6 (600 MHz).........39
Fig. S35 ^{1}H-^{1}H COSY spectrum of **3** in DMSO-d_6 (600 MHz).........40
Fig. S36 ROESY spectrum of **3** in DMSO-d_6 (600 MHz).........41
Fig. S37 ^{1}H NMR Spectrum of **3** in methanol-d_4 (600 MHz).........42
Fig. S38 ^{13}C NMR spectrum of **3** in methanol-d_4 (600 MHz).........43
Fig. S39 HSQC spectrum of **3** in methanol-d_4 (600 MHz).........44
Fig. S40 HMBC spectrum of **3** in methanol-d_4 (600 MHz).........45
Fig. S41 ^{1}H–^{1}H COSY spectrum of **3** in methanol-d_4 (600 MHz).........46
Fig. S42 ROESY spectrum of **3** in methanol-d_4 (600 MHz).........47
Fig. S43 ^{1}H NMR spectrum of **3** in pyridine-d_5 (600 MHz).........48
Fig. S44 1D-TOCSY spectrum of **3** in pyridine-d_5 (800 MHz).........49
Fig. S45 ^{13}C NMR spectrum of **3** in pyridine-d_5 (150 MHz).........50
Fig. S46 HSQC spectrum of **3** in pyridine-d_5 (600 MHz).........51
Fig. S47 HMBC spectrum of **3** in pyridine-d_5 (600 MHz).........52
Fig. S48 ^{1}H–^{1}H COSY spectrum of **2** in pyridine-d_5 (600 MHz).........53
Fig. S49 HRESIMS spectrum of **3**.........54
Fig. S50 IR spectrum of **3**.........55
Fig. S51 UV spectrum of **3**.........56
Fig. S52 ^{1}H NMR Spectrum of **4** in chloroform-d (600 MHz).........57
Fig. S53 ^{1}H NMR Spectrum of **4** in chloroform-d (600 MHz) (expanded).........58
Fig. S54 ^{13}C NMR Spectrum of **4** in chloroform-d (150 MHz).........59
Fig. S56 HMBC Spectrum of **4** in chloroform-d (600 MHz).........61
Fig. S57 ^{1}H–^{1}H COSY Spectrum of **4** in chloroform-d (600 MHz).........62
Fig. S58 ROESY Spectrum of **4** in chloroform-d (600 MHz).........63
Fig. S59 ROESY Spectrum of **4** in chloroform-d (600 MHz) (expanded).........64
Fig. S60 ROESY Spectrum of **4** in chloroform-d (600 MHz) (expanded).........65
Fig. S61 ^{1}H NMR Spectrum of **4** in DMSO-d_6 (600 MHz).........66
Fig. S62 ^{13}C NMR Spectrum of **4** in DMSO-d_6 (150 MHz).........67
Fig. S63 HSQC Spectrum of **4** in DMSO-d_6 (600 MHz).........68

Fig. S64 HMBC Spectrum of **4** in DMSO-d_6 (600 MHz)........69
Fig. S65 ^{1}H–^{1}H COSY Spectrum of **4** in DMSO-d_6 (600 MHz)........70
Fig. S66 ROESY Spectrum of **4** in DMSO-d_6 (600 MHz)........71
Fig. S67 ROESY Spectrum of **4** in DMSO-d_6 (600 MHz) (expanded)........72
Fig. S68 HRESIMS Spectrum of **4**. 73
Fig. S69 IR Spectrum of **4**........74
Fig. S70 UV Spectrum of **4**. 75
Fig. S71 ^{1}H NMR Spectrum of **5** in methanol-d_4 (600 MHz). 76
Fig. S72 ^{13}C NMR Spectrum of **5** in methanol-d_4 (150 MHz). 77
Fig. S73 The DEPT Spectrum of **5** in methanol-d_4 (150 MHz). 78
Fig. S74 HSQC Spectrum of **5** in methanol-d_4 (600 MHz). 79
Fig. S75 HMBC Spectrum of **5** in methanol-d_4 (600 MHz). 80
Fig. S76 ^{1}H–^{1}H COSY Spectrum of **5** in methanol-d_4 (600 MHz)........81
Fig. S77 HRESIMS Spectrum of **5**. 82
Fig. S78 IR Spectrum of **5**........83
Fig. S79 UV Spectrum of **5**. 84
Fig. S80 ^{1}H NMR spectrum of **6** in DMSO-d_6 (600 MHz). 85
Fig. S81 ^{13}C NMR spectrum of **6** in DMSO-d_6 (150 MHz). 85
Fig. S82 ^{1}H NMR spectrum of **7** in chloroform-*d* (600 MHz). 86
Fig. S83 ^{13}C NMR spectrum of **7** in chloroform-*d* (150 MHz). 86
Fig. S84 ^{1}H NMR spectrum of **8** in methanol-d_4 (600 MHz). 87
Fig. S85 ^{13}C NMR spectrum of **8** in methanol-d_4 (150 MHz). 87
Fig. S86 ^{1}H NMR spectrum of **9** in methanol-d_4 (600 MHz). 88
Fig. S87 ^{13}C NMR spectrum of **9** in methanol-d_4 (150 MHz). 88
Fig. S88 ^{1}H NMR spectrum of **10** in methanol-d_4 (400 MHz). 89
Fig. S89 ^{13}C NMR spectrum of **10** in methanol-d_4 (100 MHz). 89
Fig. S90 ^{1}H NMR spectrum of **11** in methanol-d_4 (600 MHz). 90
Fig. S91 ^{13}C NMR spectrum of **11** in methanol-d_4 (150 MHz). 90
Fig. S92 ^{1}H NMR spectrum of **12** in methanol-d_4 (600 MHz). 91
Fig. S93 ^{13}C NMR spectrum of **12** in methanol-d_4 (150 MHz). 91
Fig. S94 ^{1}H NMR spectrum of **13** in methanol-d_4 (600 MHz). 92
Fig. S95 ^{13}C NMR spectrum of **13** in methanol-d_4 (150 MHz). 92

Fig. S96 ^{1}H NMR spectrum of **14** in chloroform-*d* (600 MHz)..93
Fig. S97 ^{13}C NMR spectrum of **14** in chloroform-*d* (150 MHz)..93
Fig. S98 ^{1}H NMR spectrum of **15** in chloroform-*d* (600 MHz)..94
Fig. S99 ^{13}C NMR spectrum of **15** in chloroform-*d* (150 MHz)..94
Fig. S100 ^{1}H NMR spectrum of **16** in methanol-d_4 (600 MHz)..95
Fig. S101 ^{13}C NMR spectrum of **16** in methanol-d_4 (150 MHz). 95
Fig. S102 ^{1}H NMR spectrum of **17** in methanol-d_4 (600 MHz)..96
Fig. S103 ^{13}C NMR spectrum of **17** in methanol-d_4 (150 MHz). 96
Fig. S104 ^{1}H NMR spectrum of **18** in chloroform-*d* (600 MHz)..97
Fig. S105 ^{13}C NMR spectrum of **18** in chloroform-*d* (150 MHz)..97
Fig. S106 The TLC and HPLC profiles of the crude extract of fungus *Xylaria* sp. Z184. 98

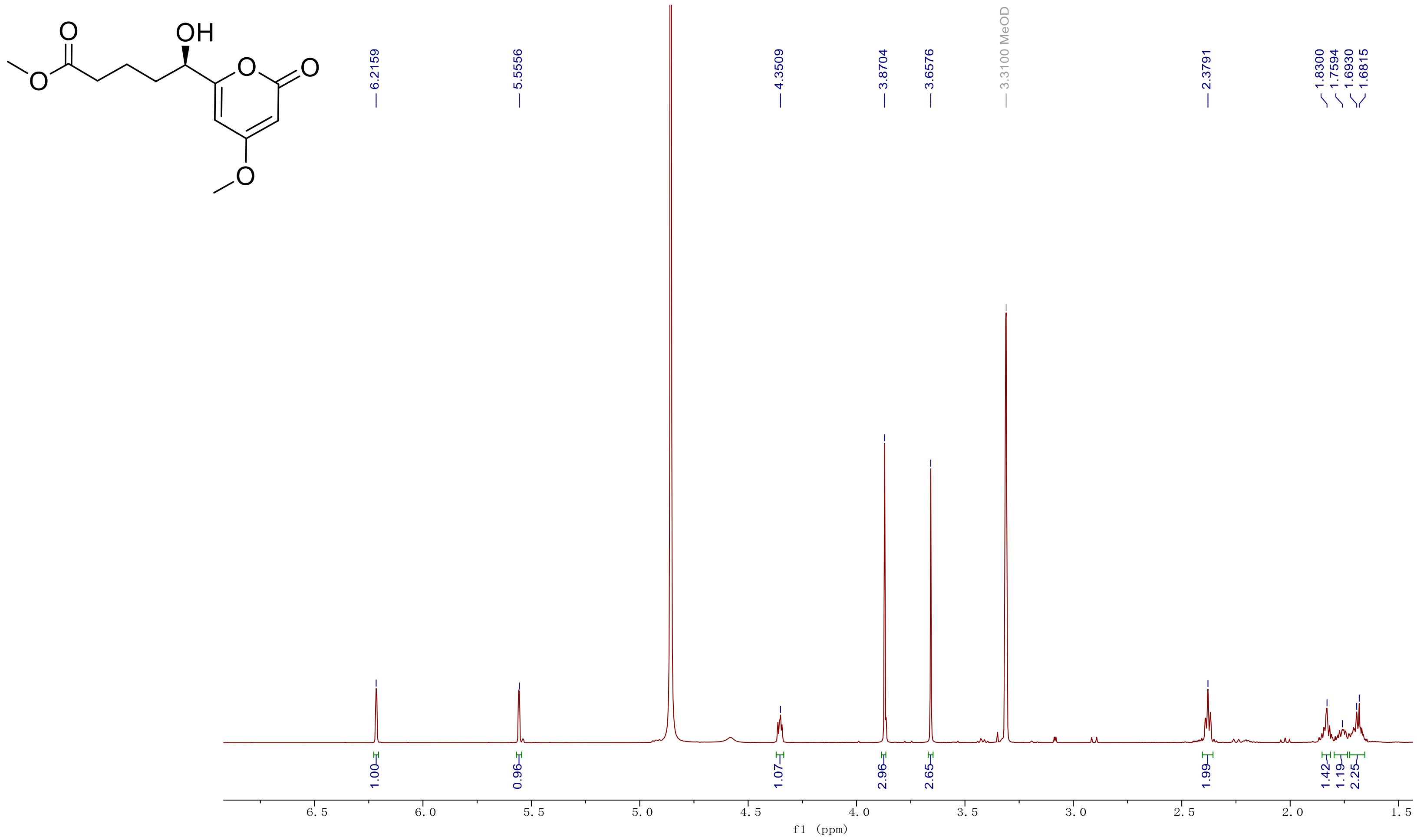

Fig. S1 ^{1}H NMR spectrum of **1** in methanol-d_4 (600 MHz).

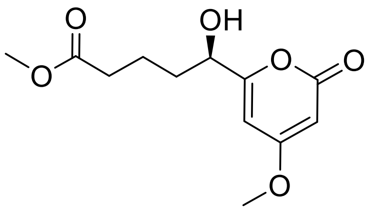

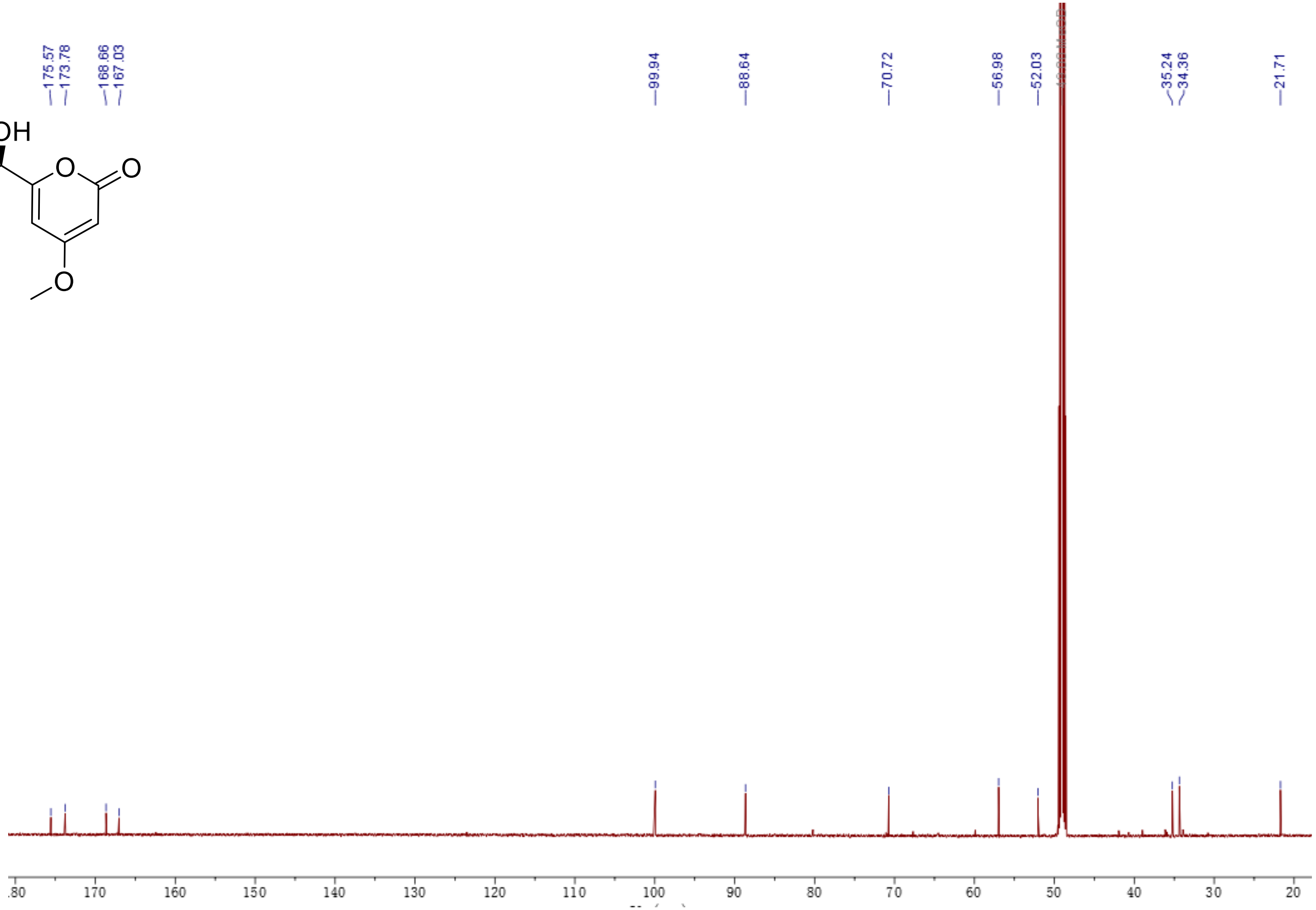

Fig. S2 ^{13}C NMR spectrum of **1** in methanol-d_4 (150 MHz).

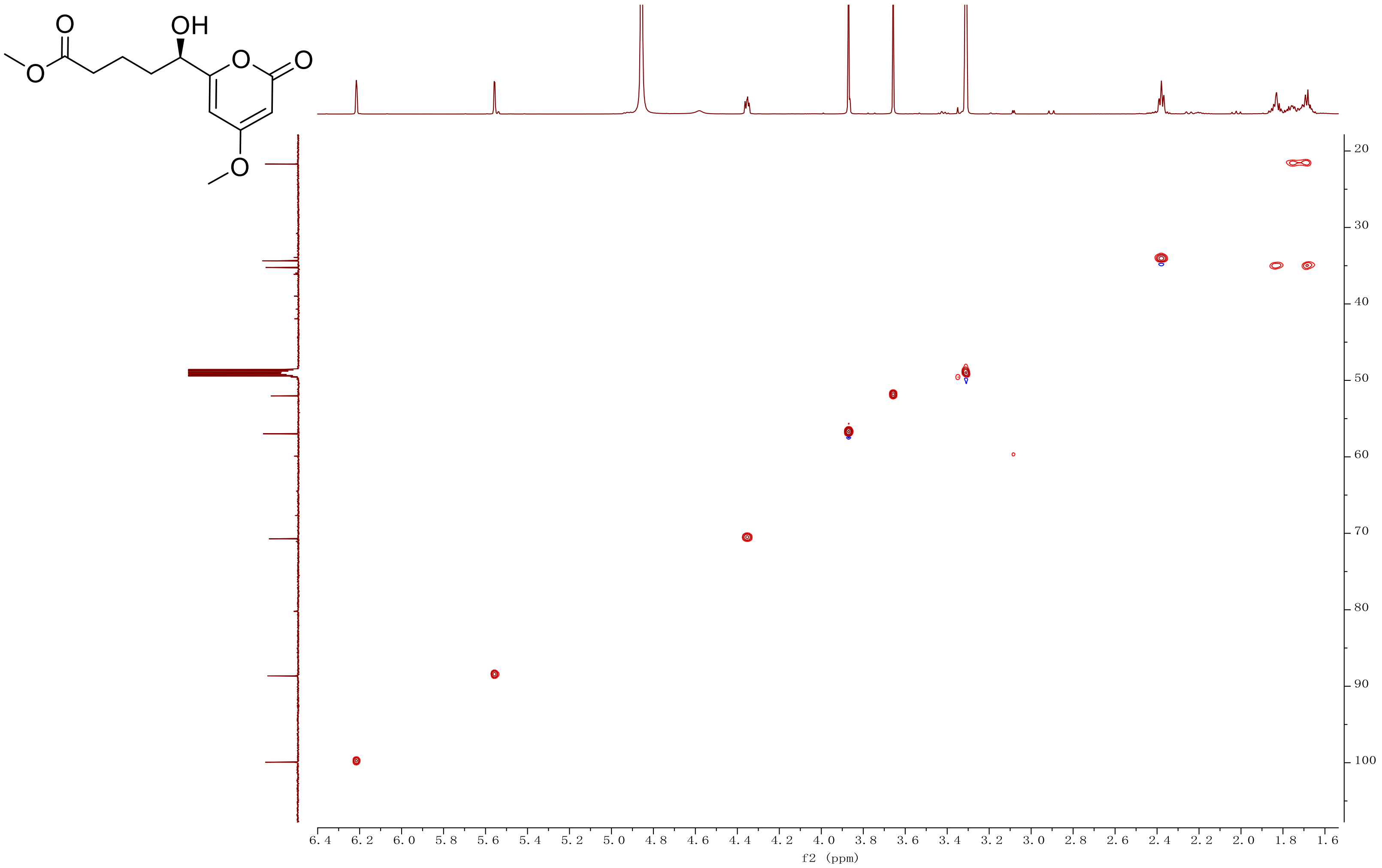

Fig. S3 HSQC spectrum of **1** in methanol-d_4 (600 MHz).

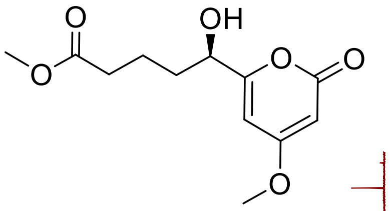

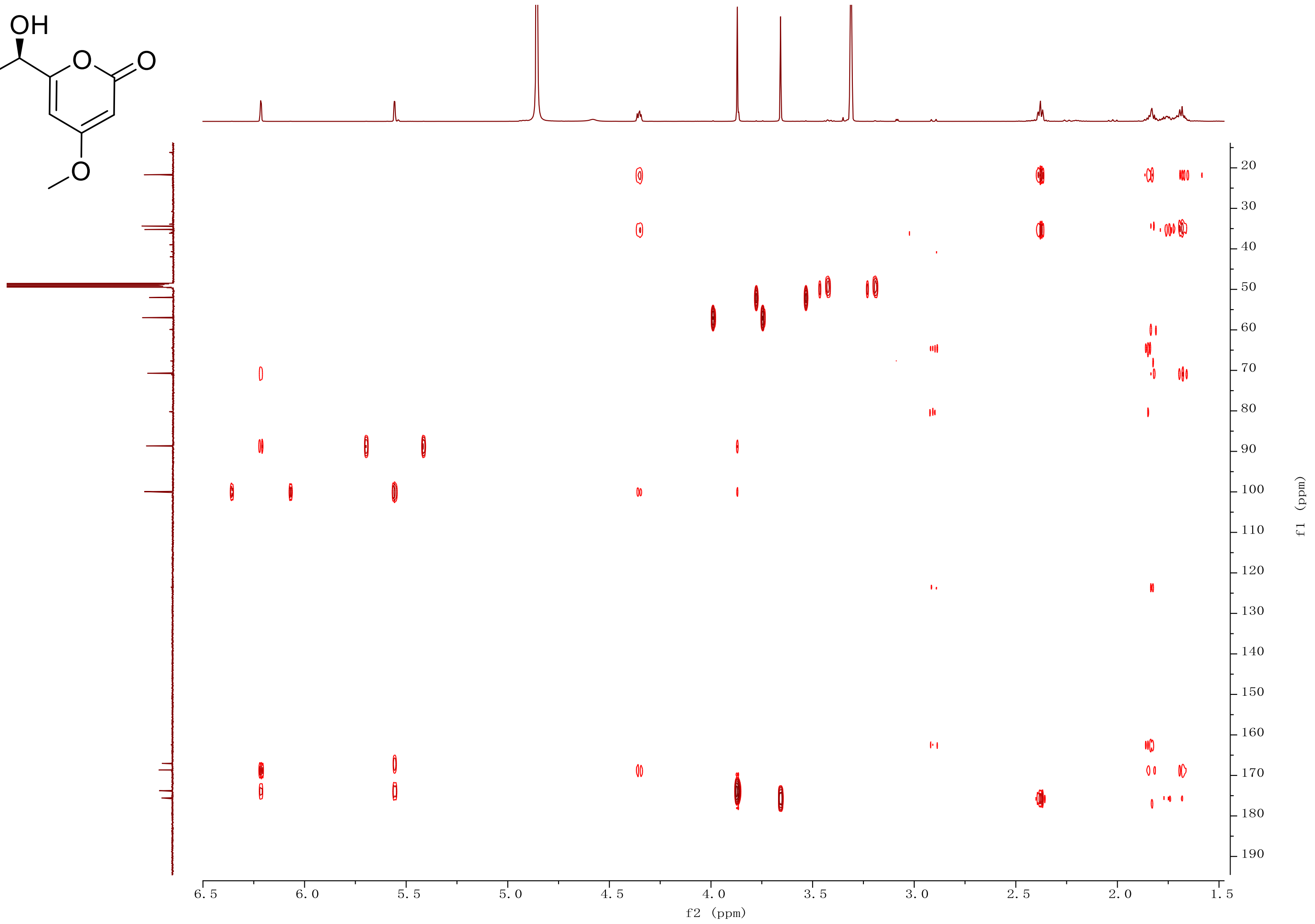

Fig. S4 HMBC spectrum of **1** in methanol-d_4 (600 MHz).

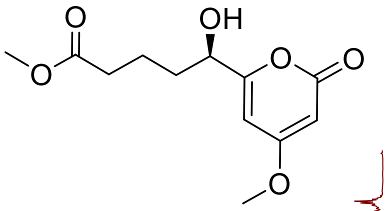

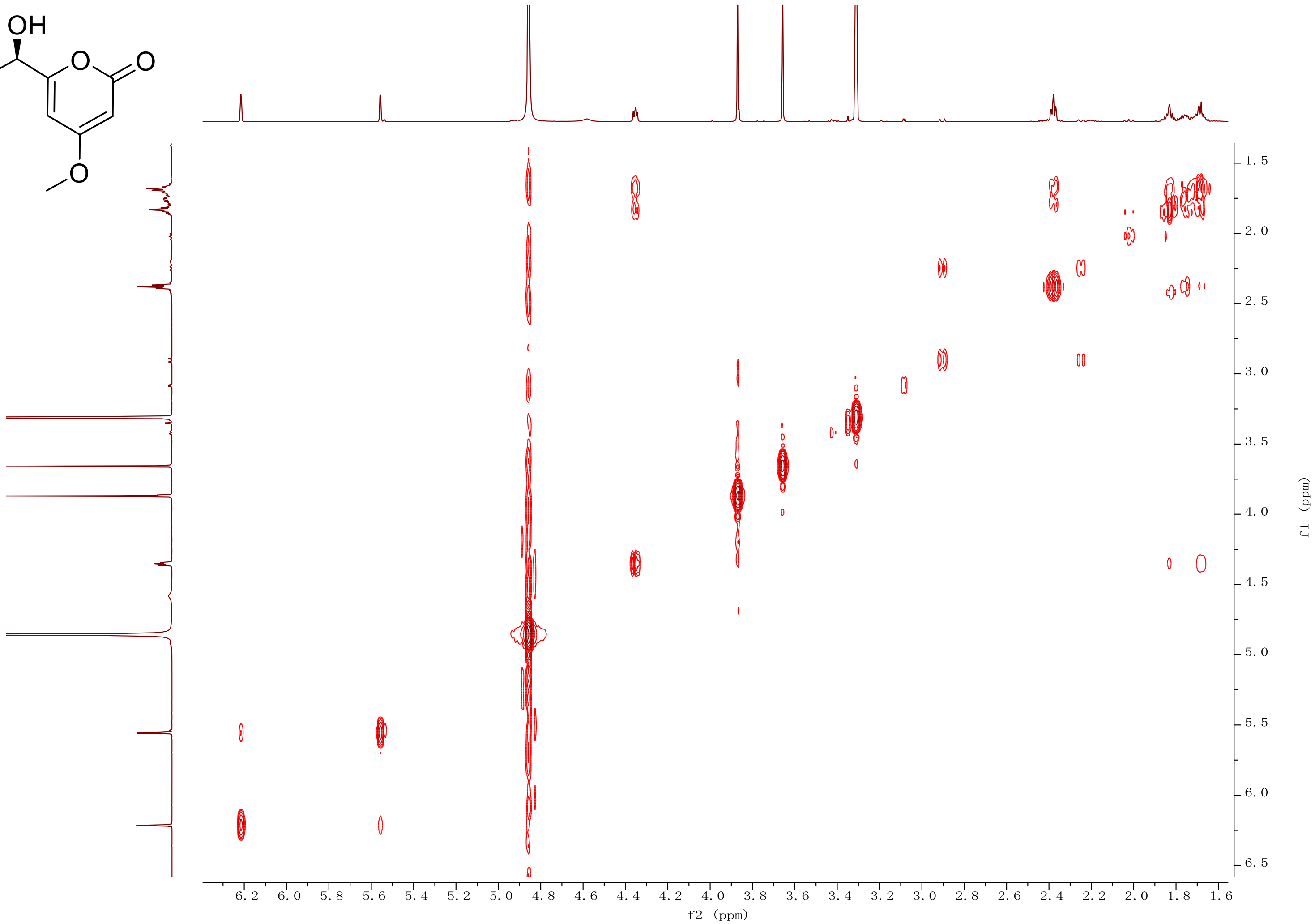

Fig. S5 ^{1}H–^{1}H COSY spectrum of **1** in methanol-d_4 (600 MHz).

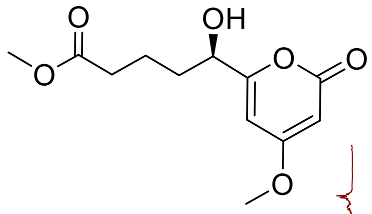

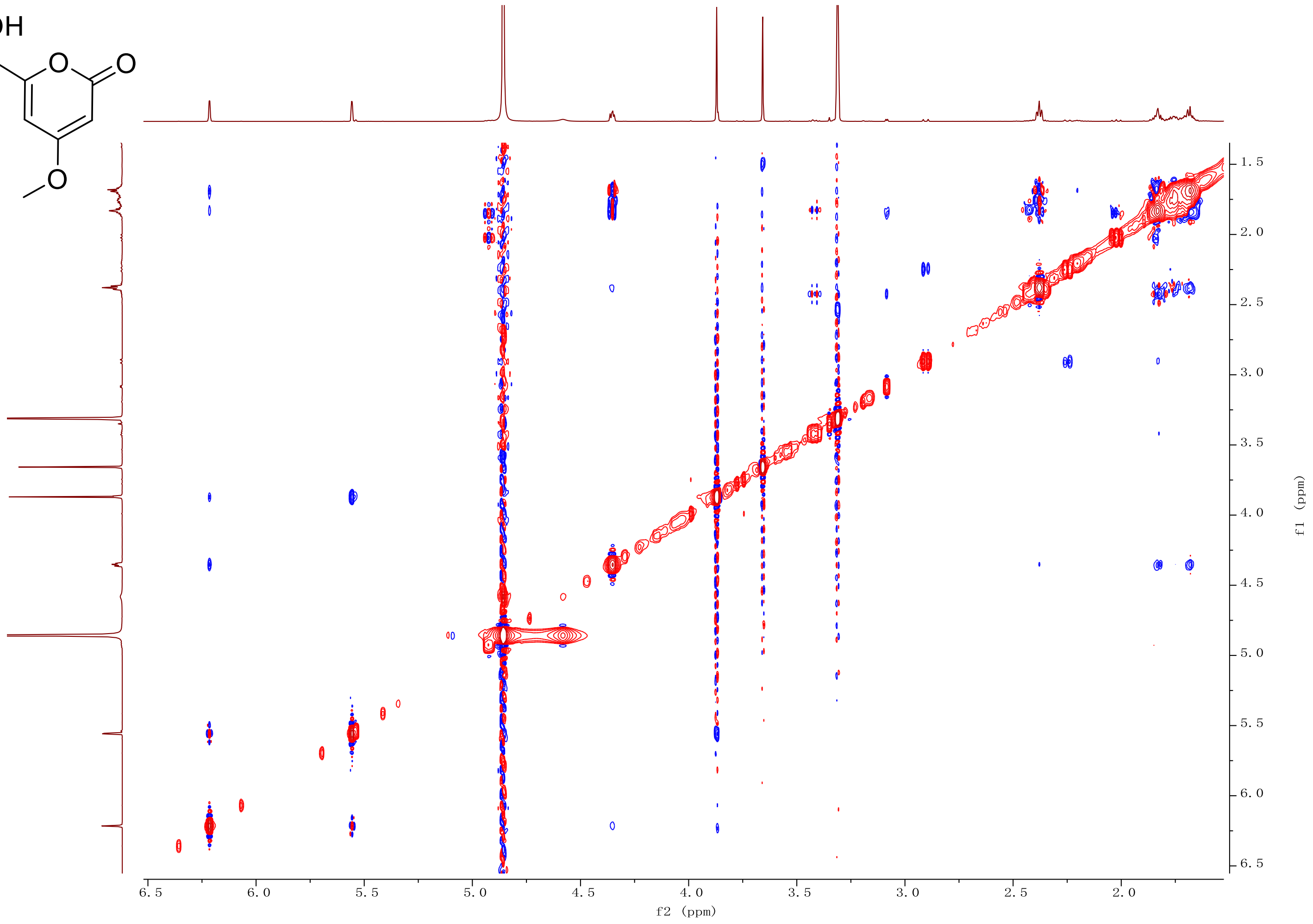

Fig. S6 ROESY spectrum of **1** in methanol-d_4 (600 MHz).

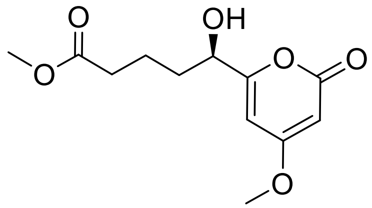

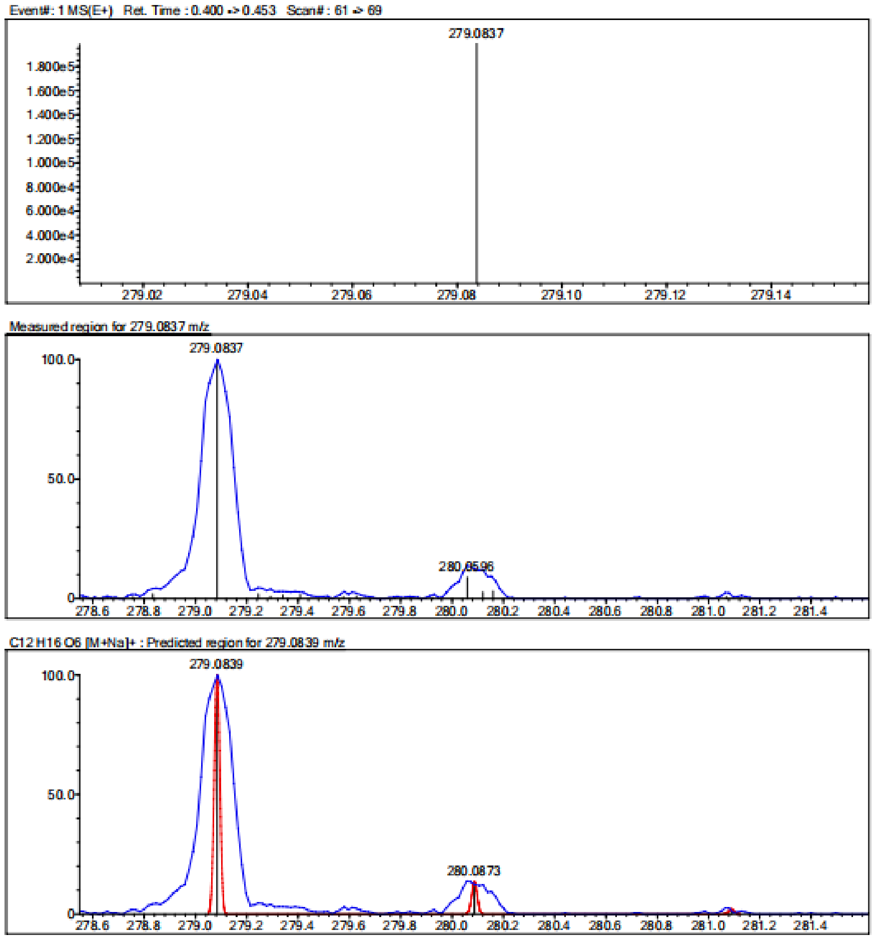

Formula (M)	Ion	Meas. m/z	Pred. m/z	Df. (mDa)	Df. (ppm)	DBE
C12 H16 O6	[M+Na]+	279.0837	279.0839	-0.2	-0.72	5.0

Fig. S7 HRESIMS spectrum of **1**.

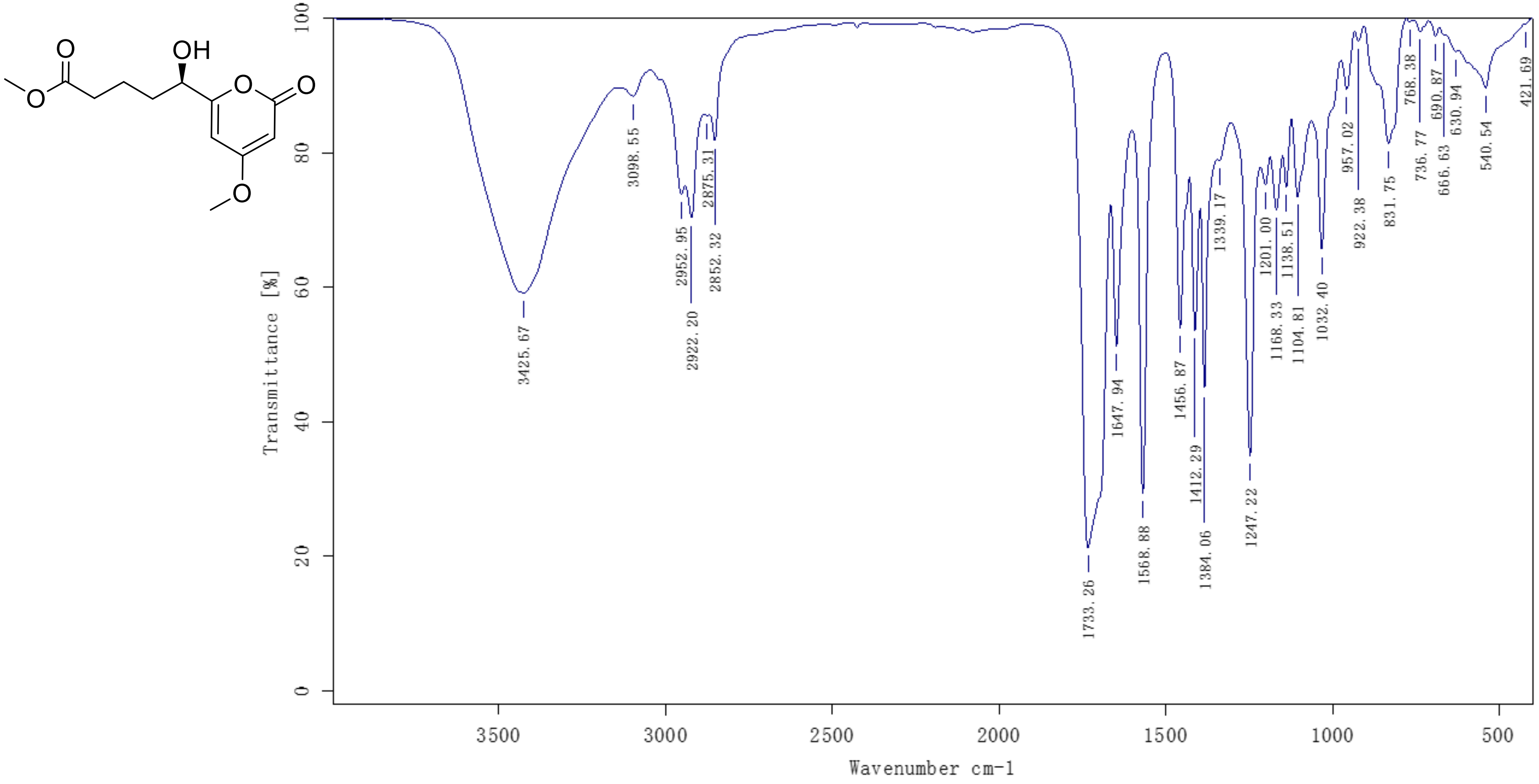

Fig. S8 IR spectrum of **1**.

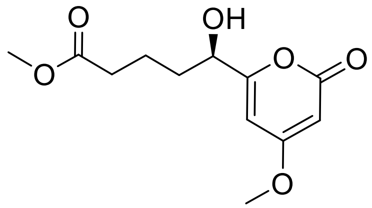

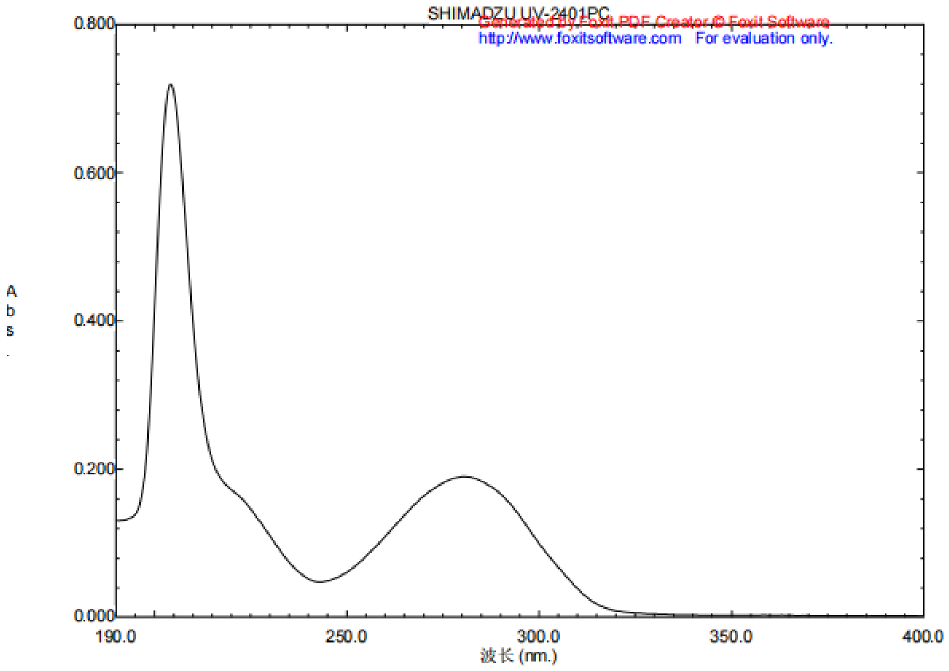

Fig. S9 UV spectrum of **1**.

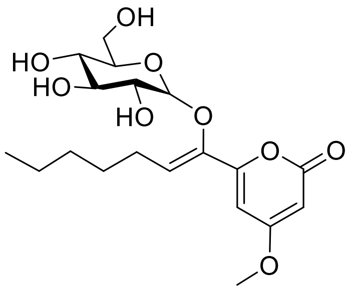

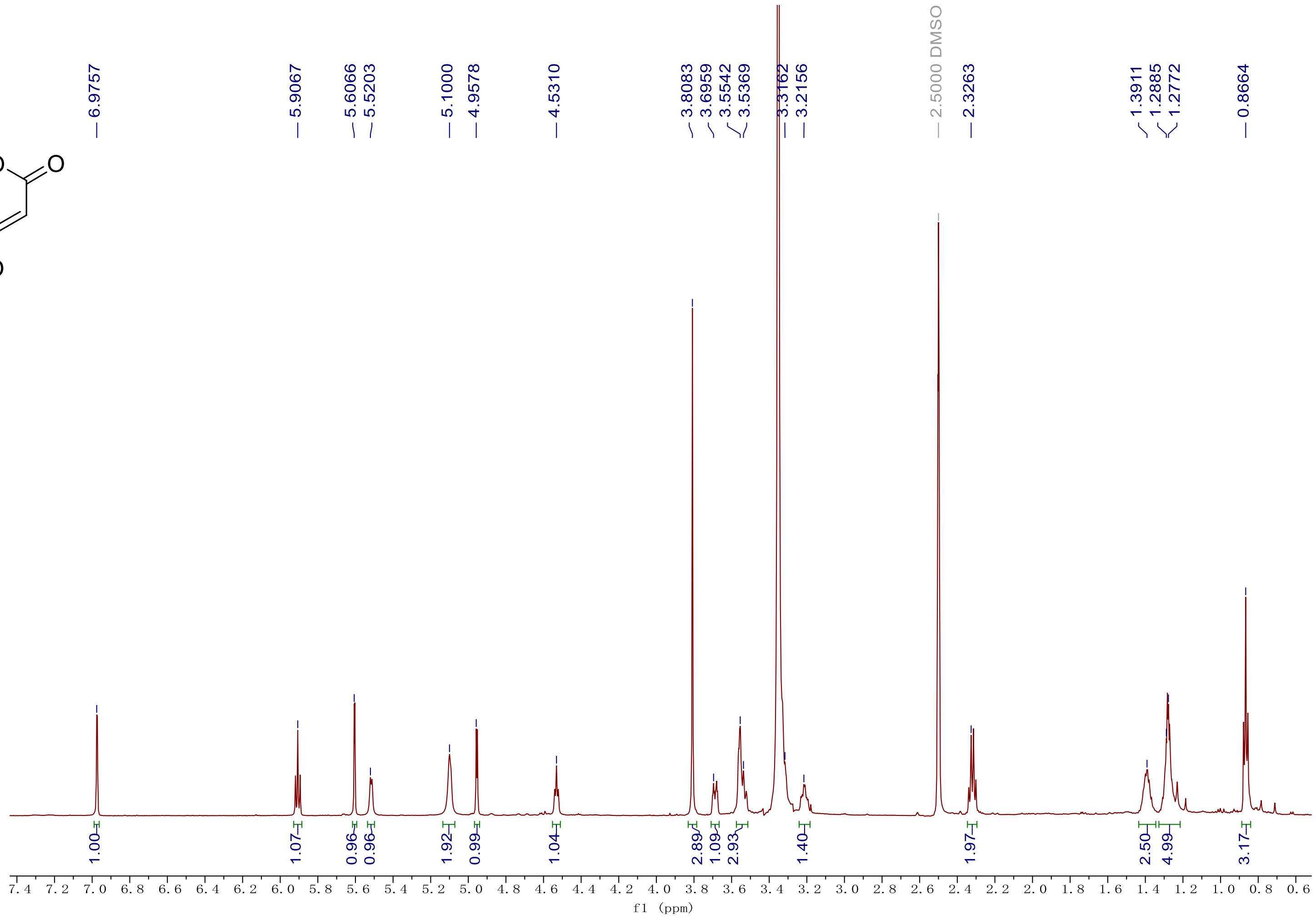

Fig. S10 ¹H NMR spectrum of **2** in DMSO-d_6 (600 MHz).

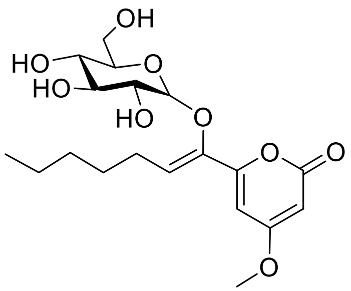

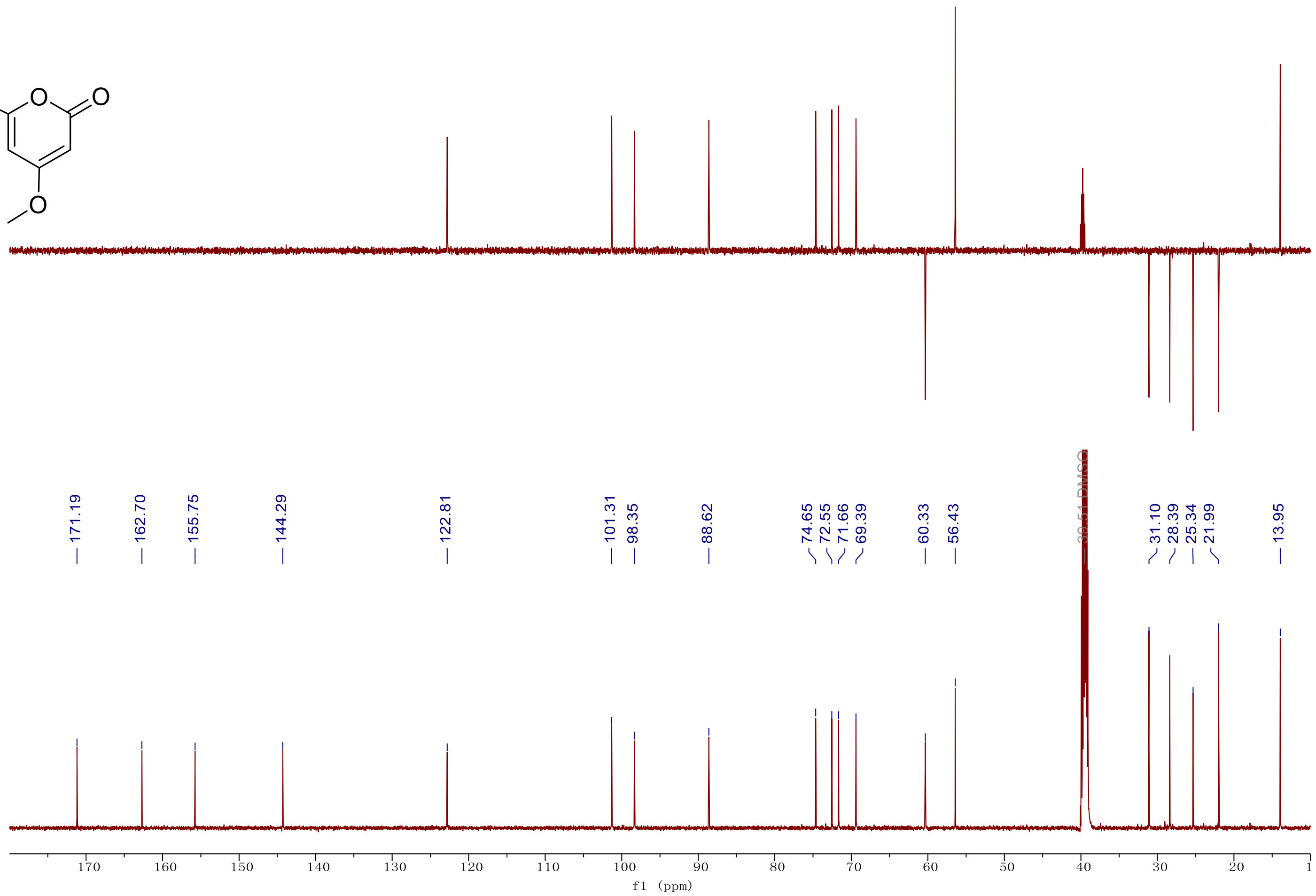

Fig. S11 ^{13}C NMR spectrum of **2** in DMSO-d_6 (150 MHz).

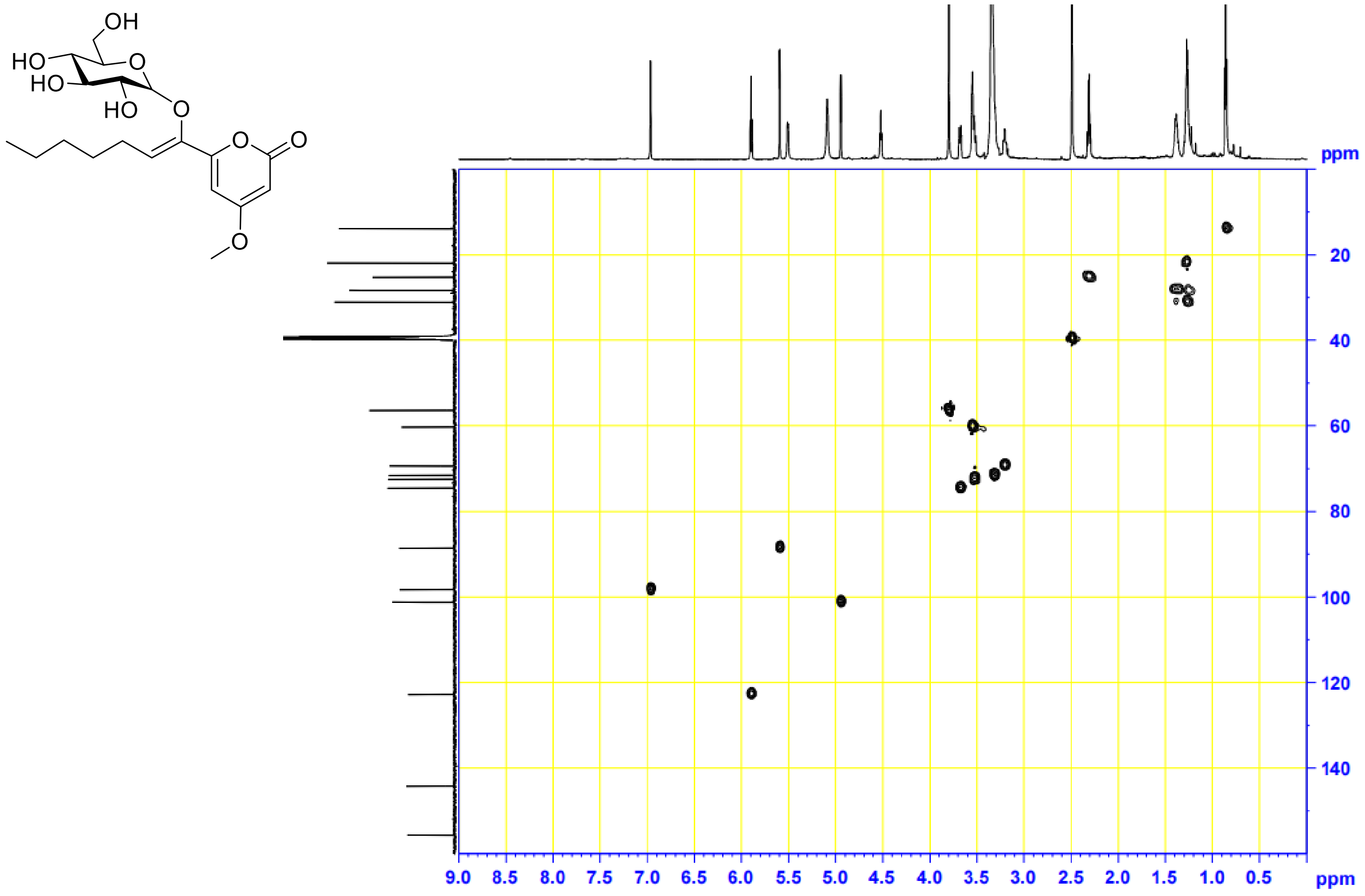

Fig. S12 HSQC spectrum of **2** in DMSO-d_6 (600 MHz).

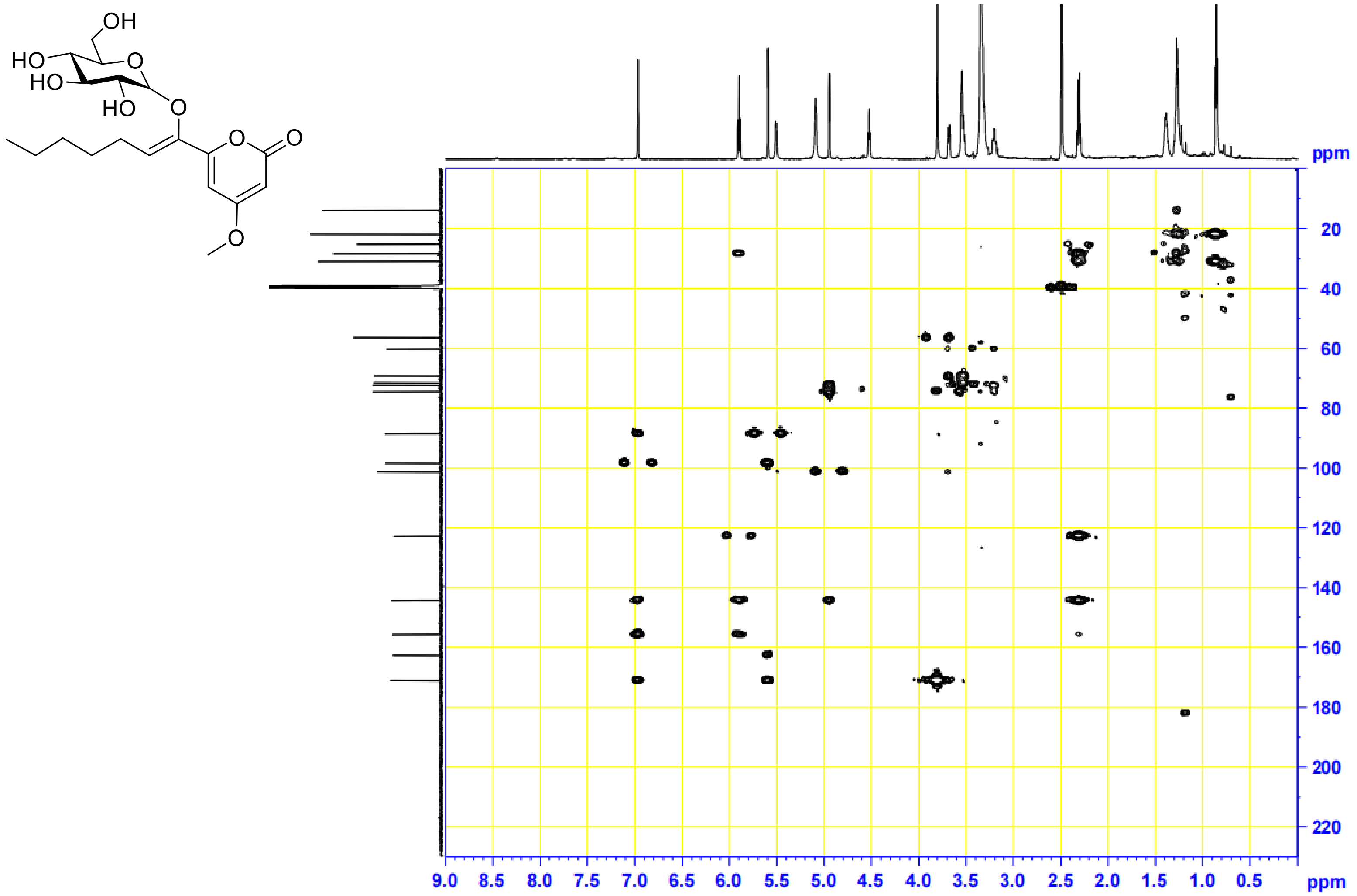

Fig. S13 HMBC spectrum of **2** in DMSO-d_6 (600 MHz).

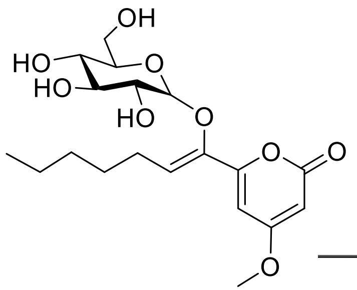

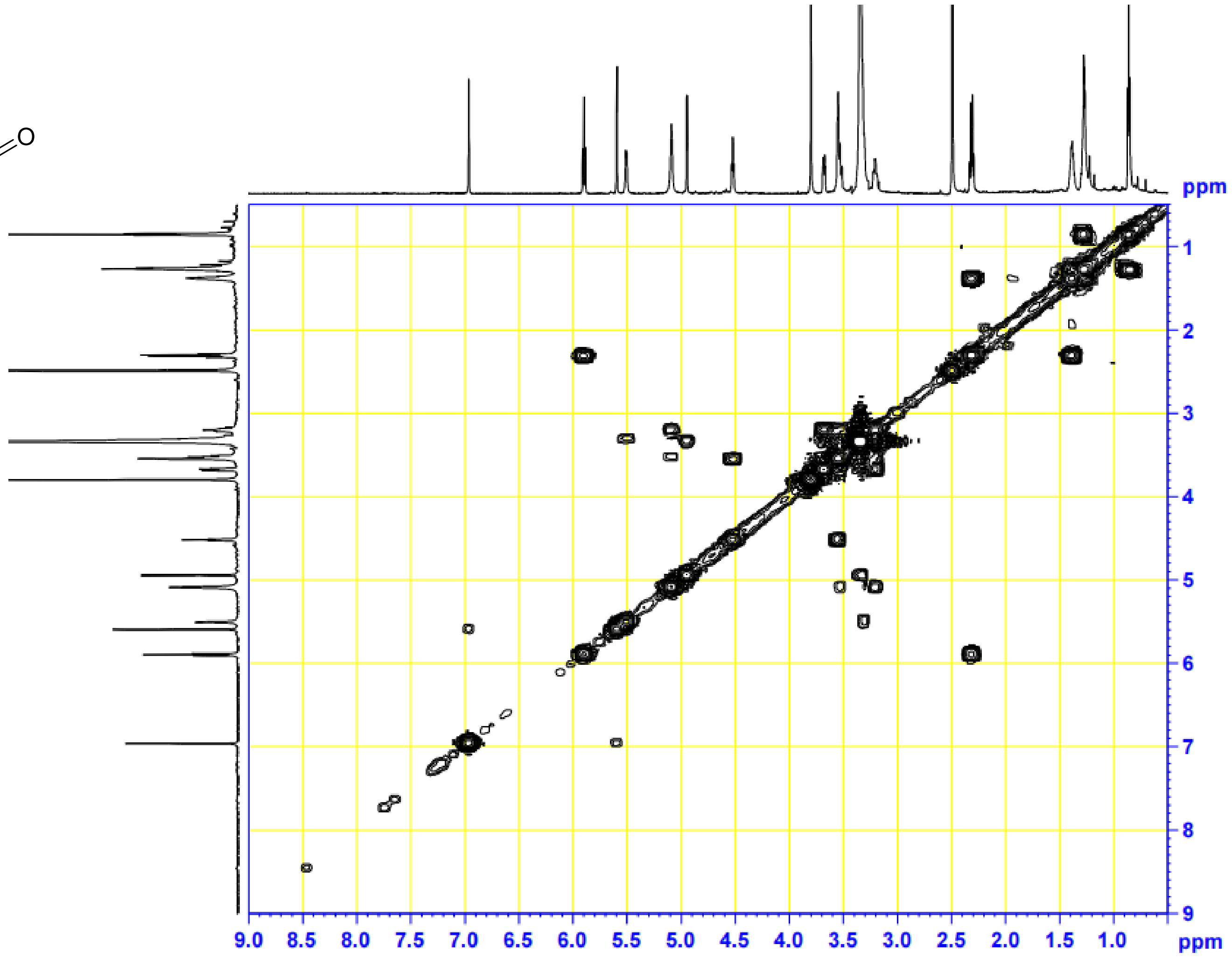

Fig. S14 ^{1}H-^{1}H COSY spectrum of **2** in DMSO-d_6 (600 MHz).

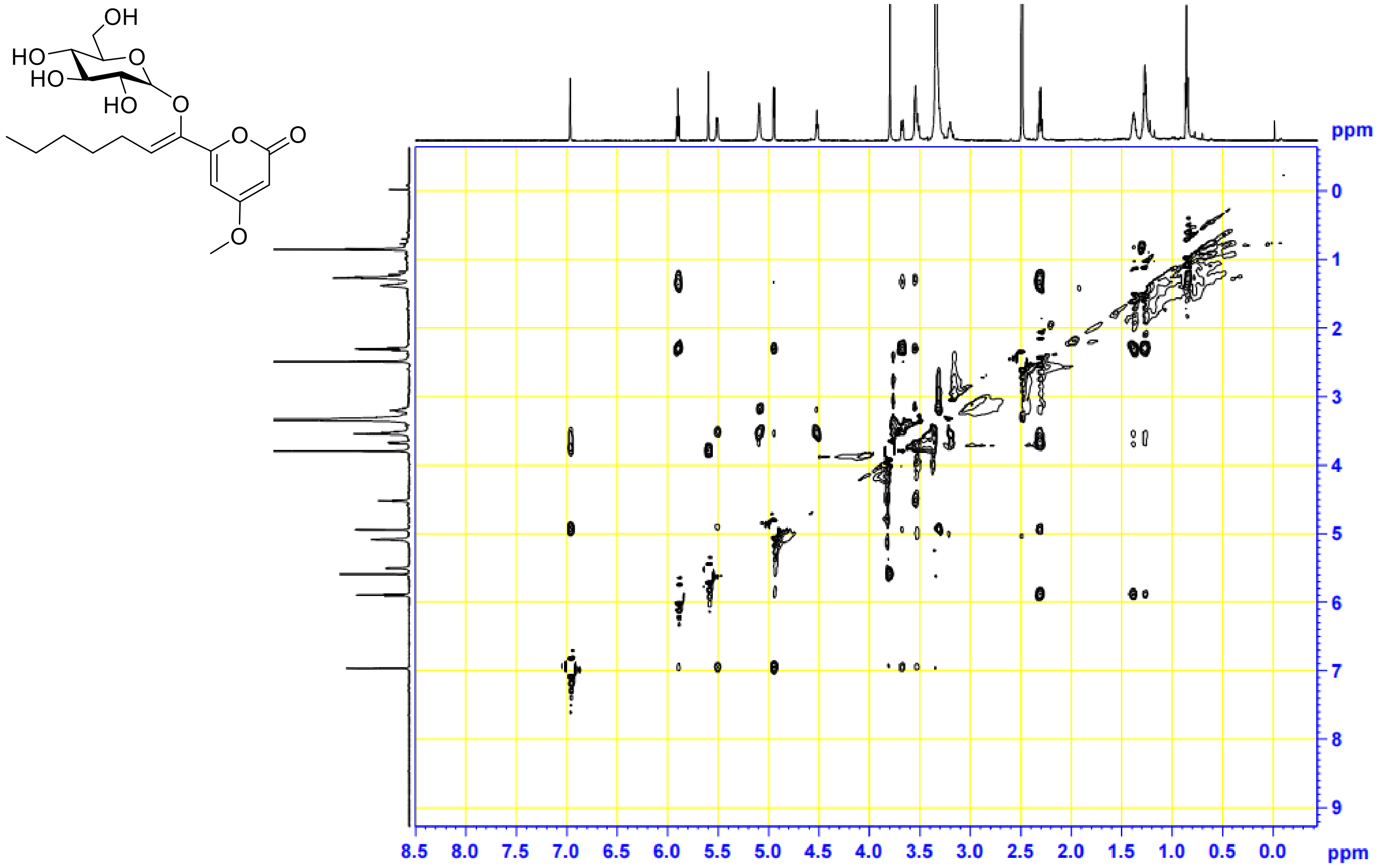

Fig. S15 ROESY spectrum of **2** in DMSO-d_6 (600 MHz).

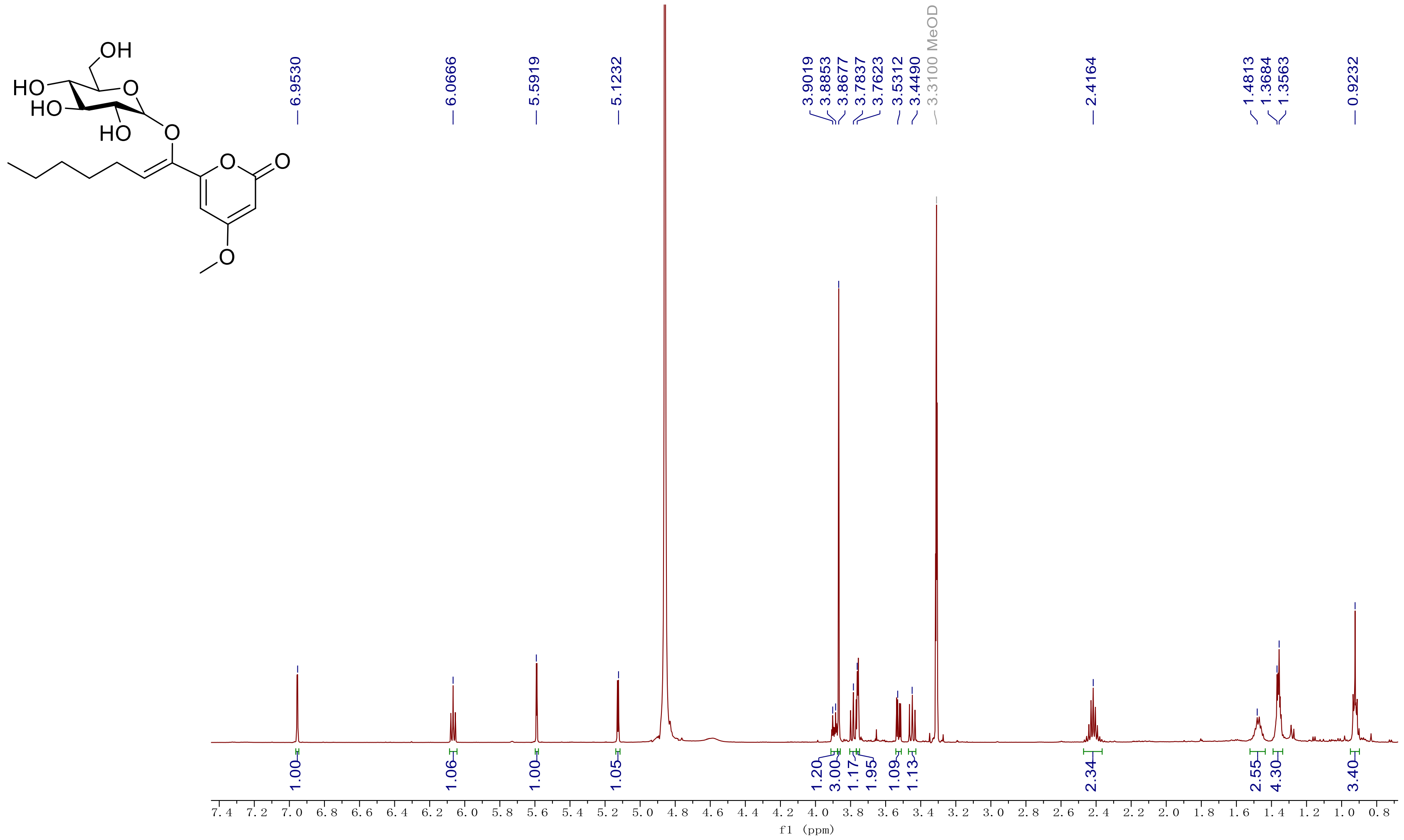

Fig. S16 ^{1}H NMR spectrum of **2** in methanol-d_4 (600 MHz).

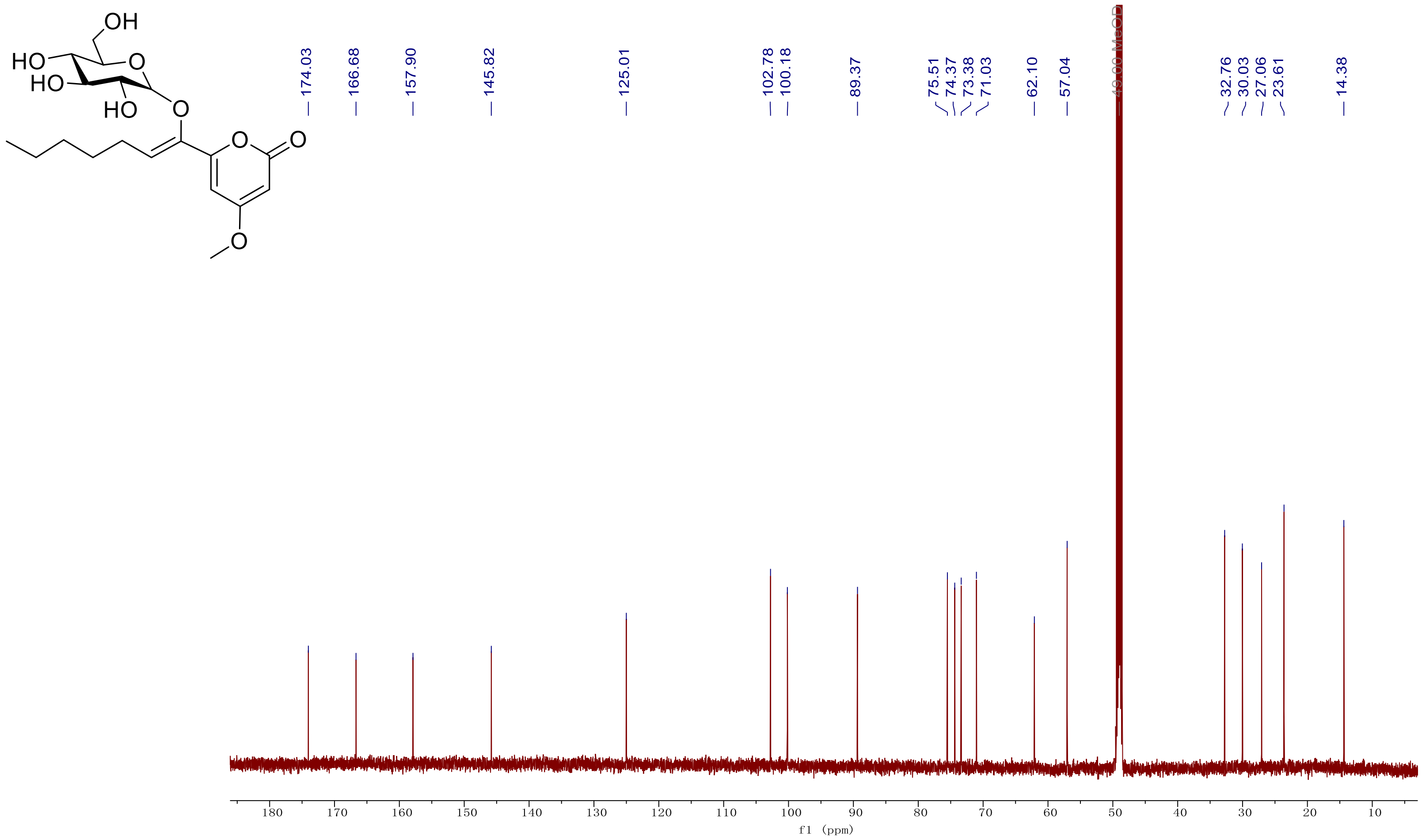

Fig. S17 ^{13}C NMR spectrum of **2** in methanol-d_4 (150 MHz).

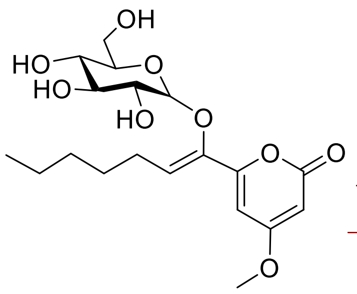

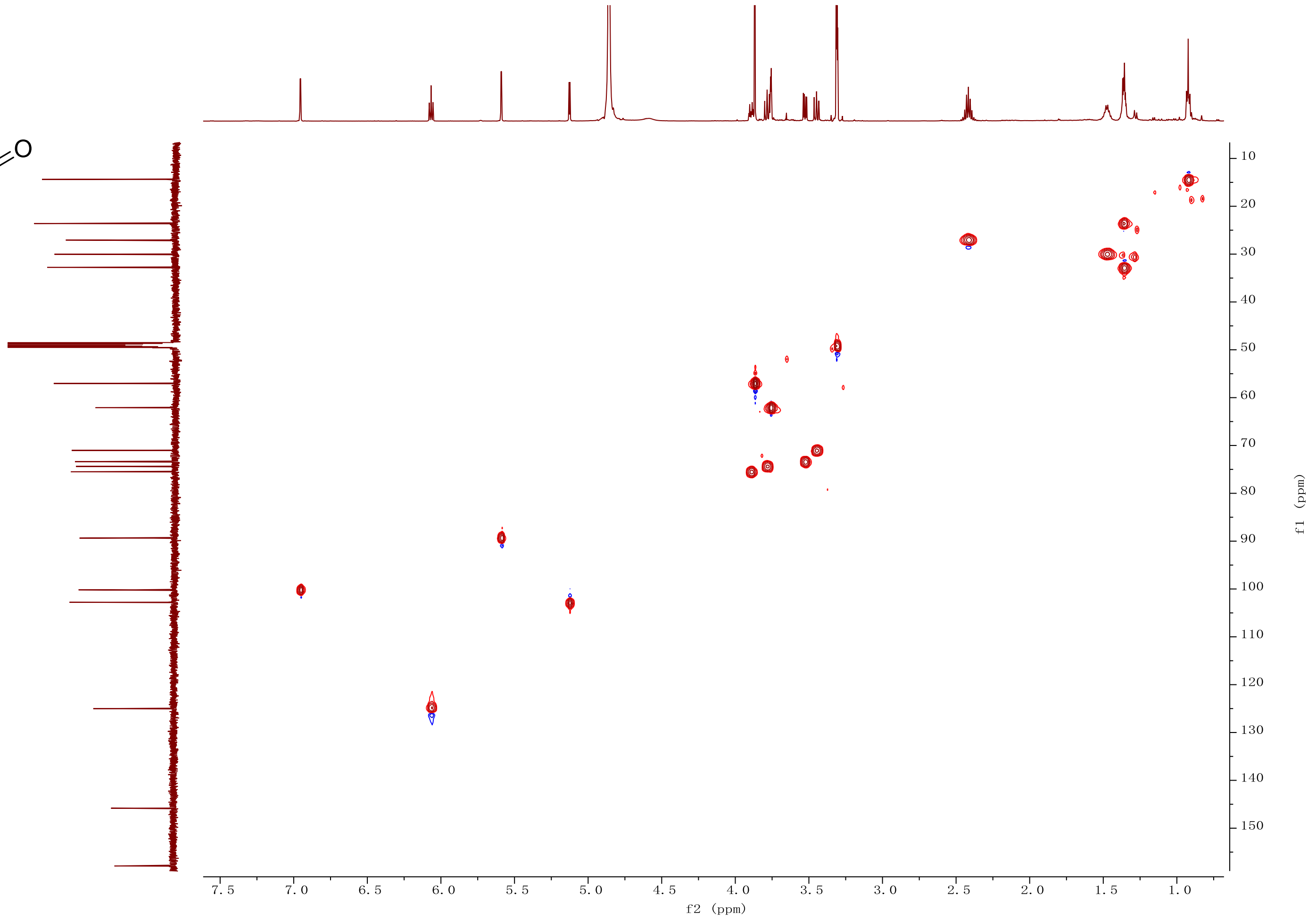

Fig. S18 HSQC spectrum of **2** in methanol-d_4 (600 MHz).

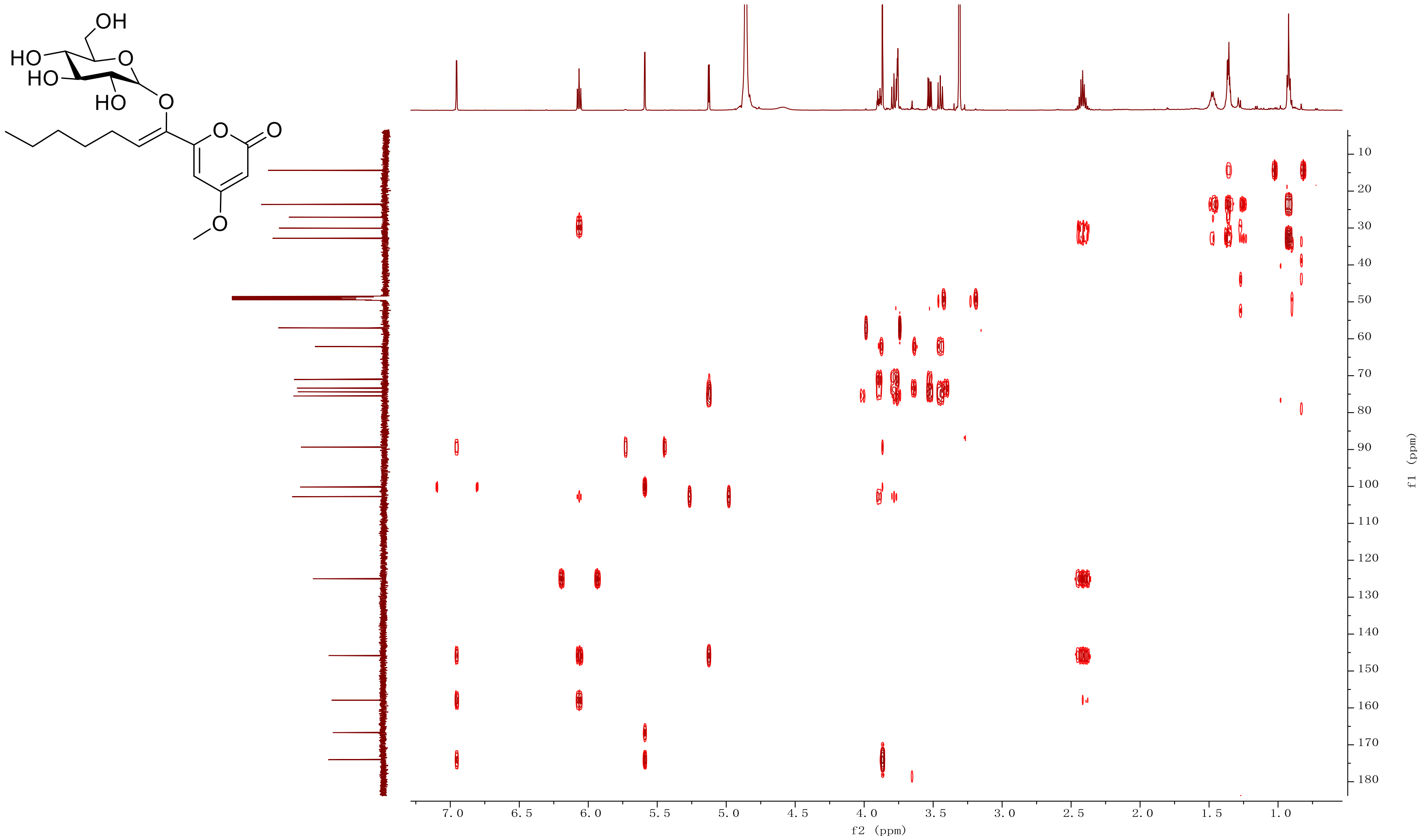

Fig. S19 HMBC spectrum of **2** in methanol-d_4 (600 MHz).

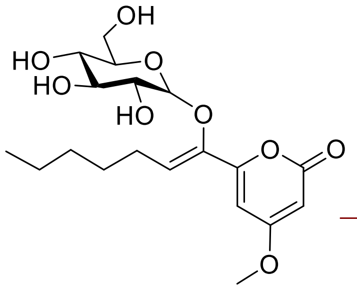

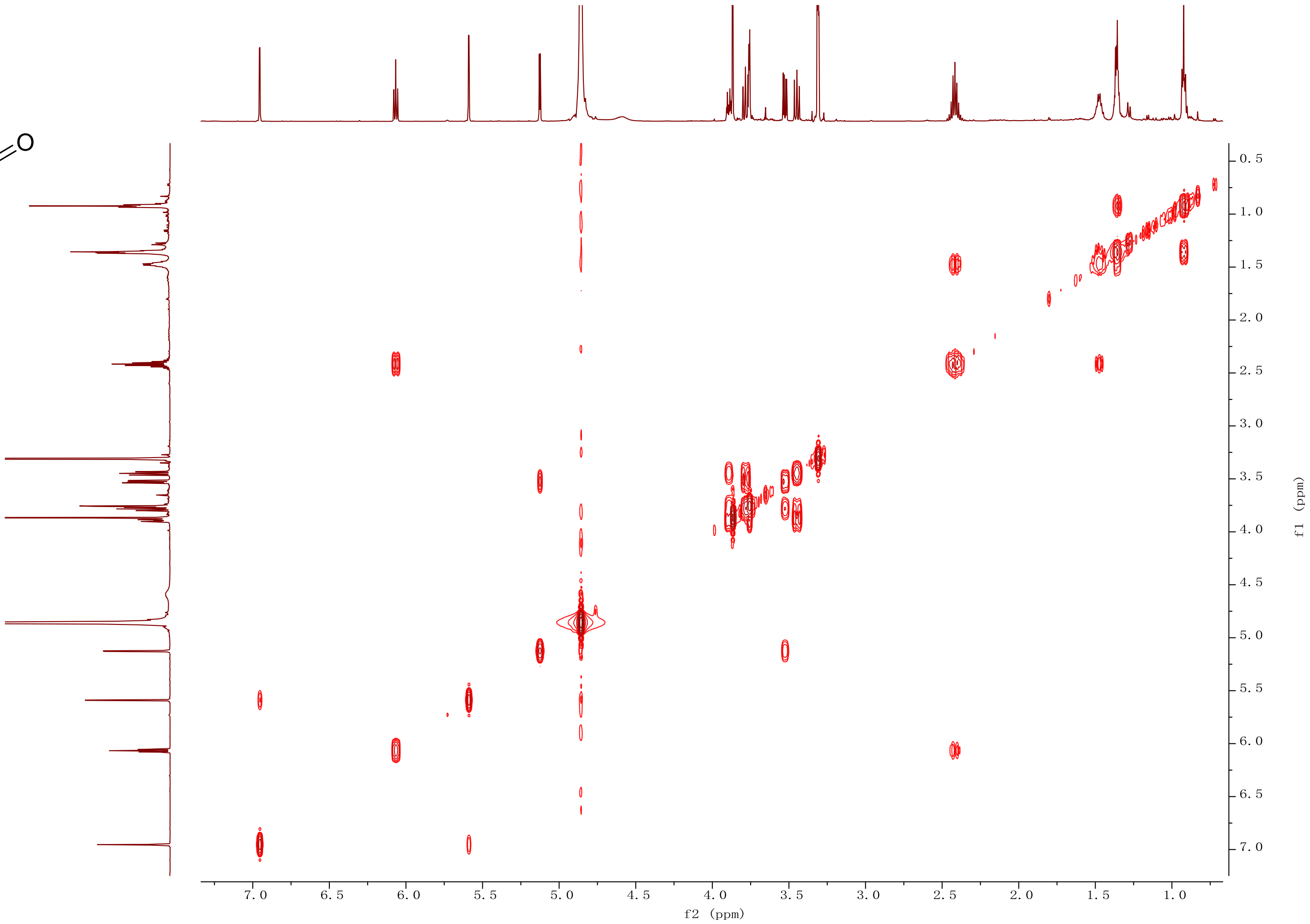

Fig. S20 ^{1}H–^{1}H COSY spectrum of **2** in methanol-d_4 (600 MHz).

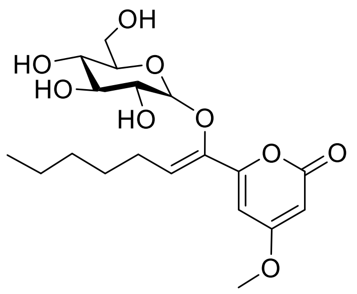

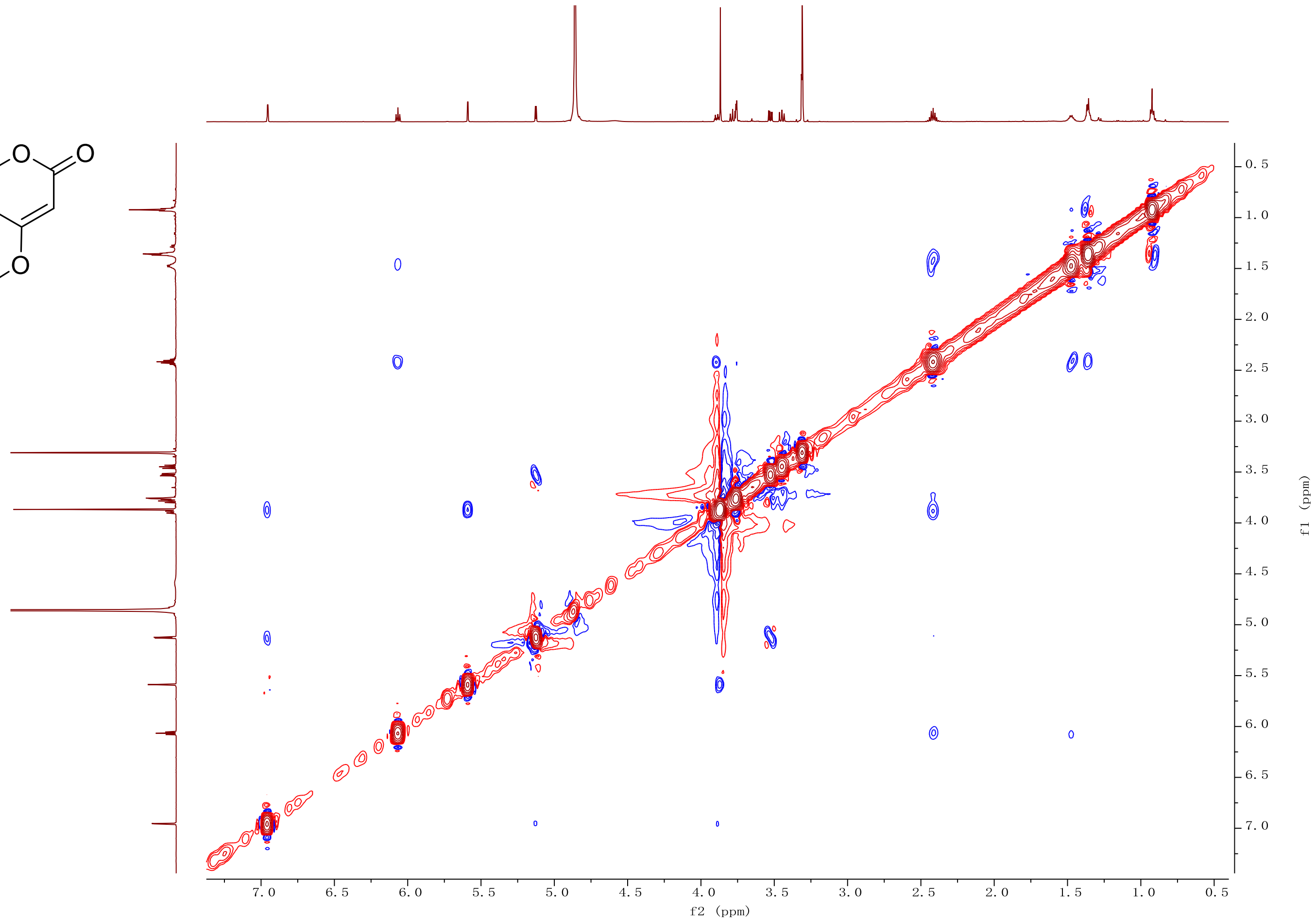

Fig. S21 ROESY spectrum of **2** in methanol-d_4 (600 MHz).

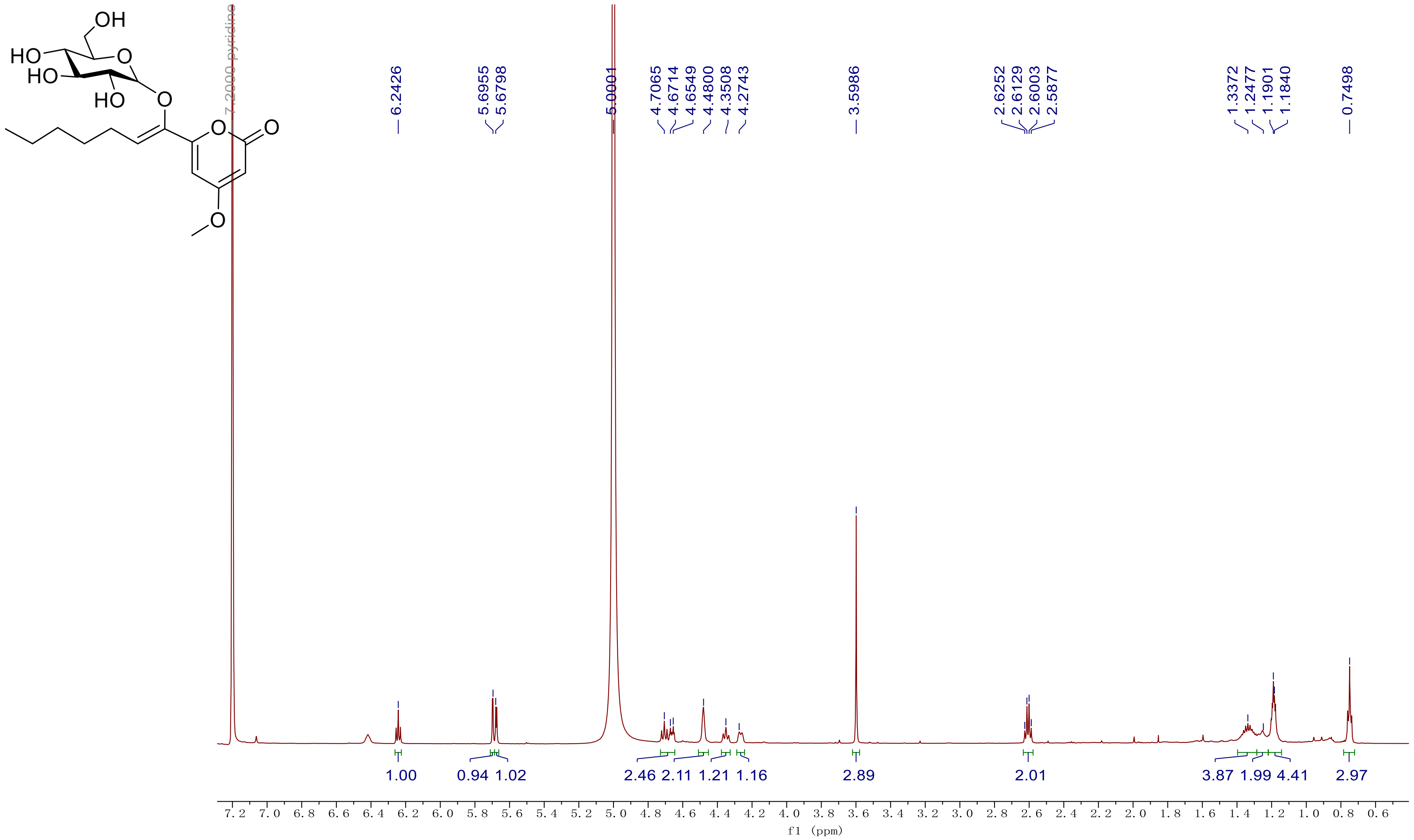

Fig. S22 ^{1}H NMR spectrum of **2** in pyridine-d_5 (600 MHz).

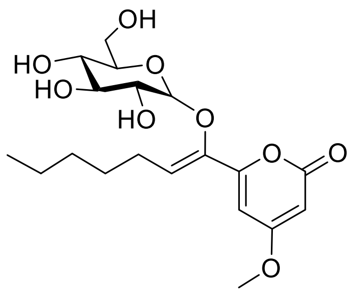

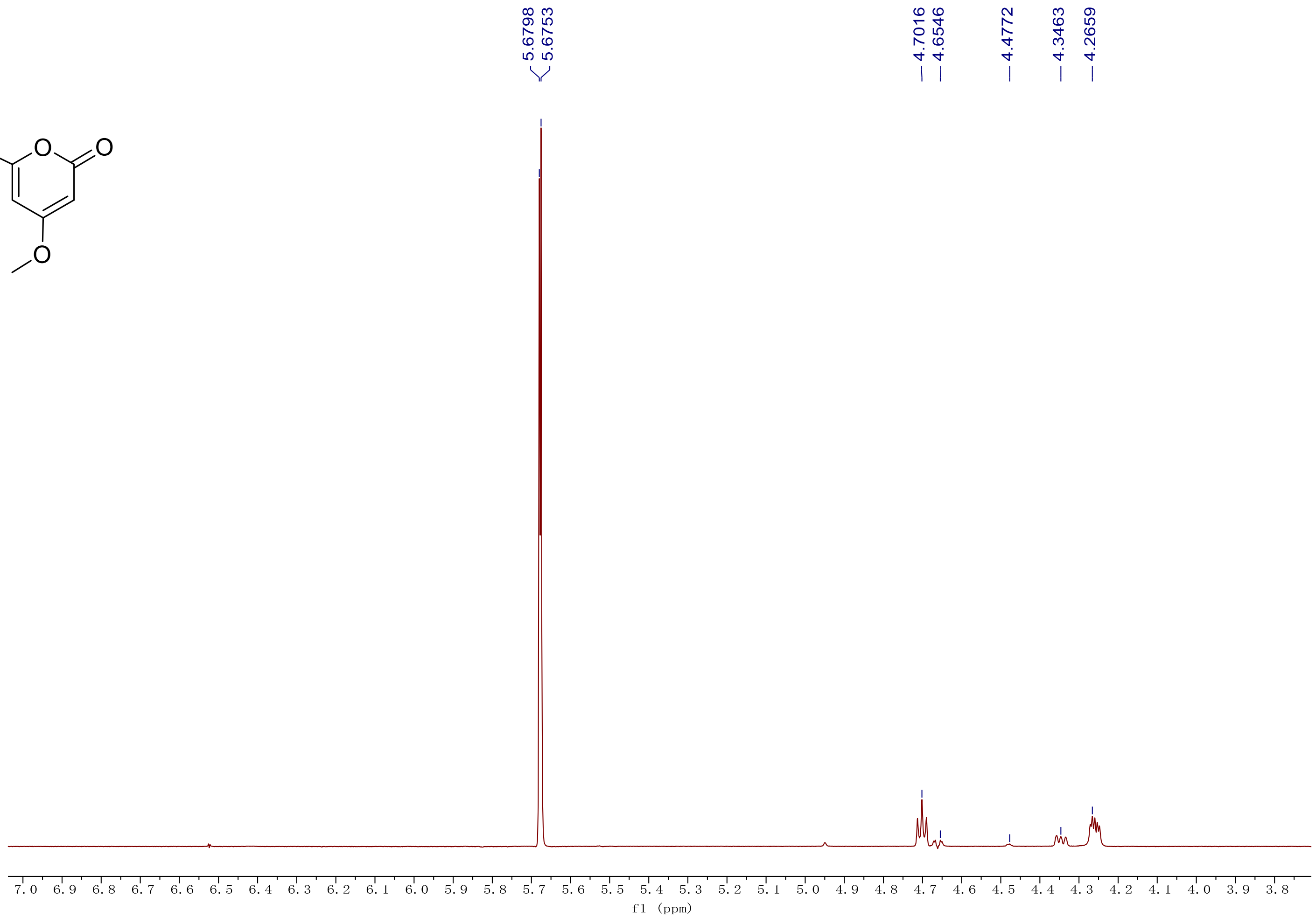

Fig. S23 1D-TOCSY spectrum of **2** in pyridine-d_5 (800 MHz).

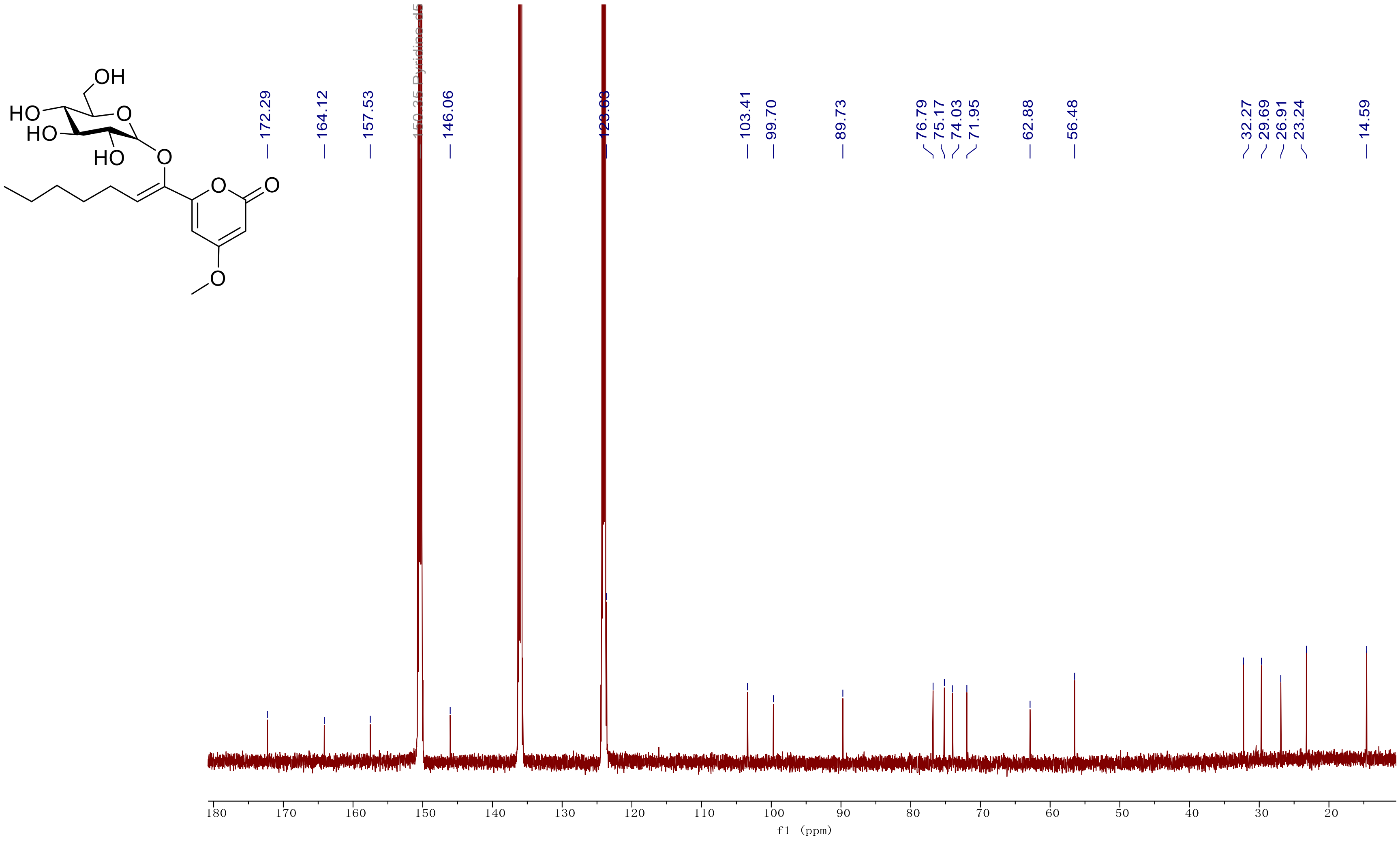

Fig. S24 ^{13}C NMR spectrum of **2** in pyridine-d_5 (150 MHz).

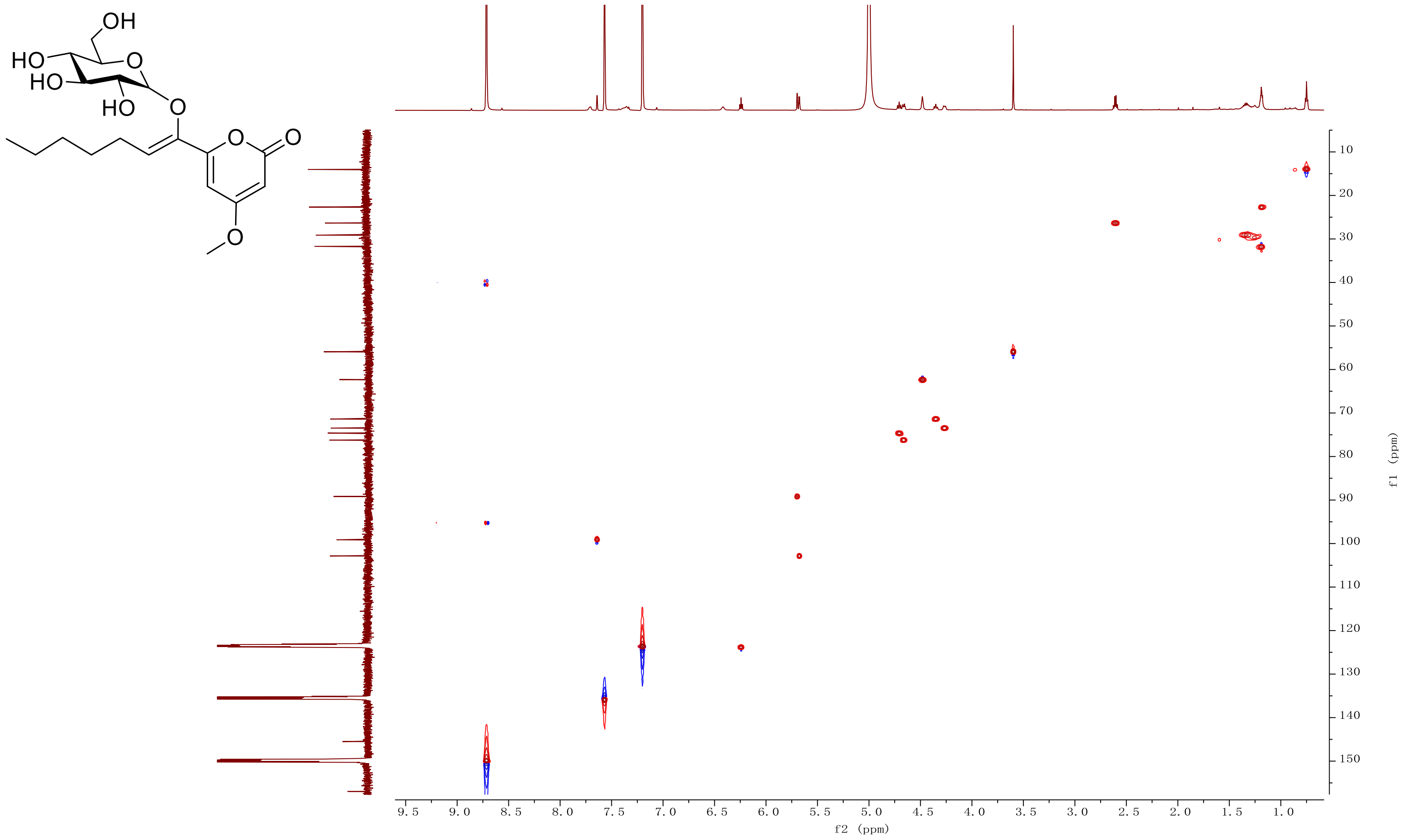

Fig. S25 HSQC spectrum of **2** in pyridine-d_5 (600 MHz).

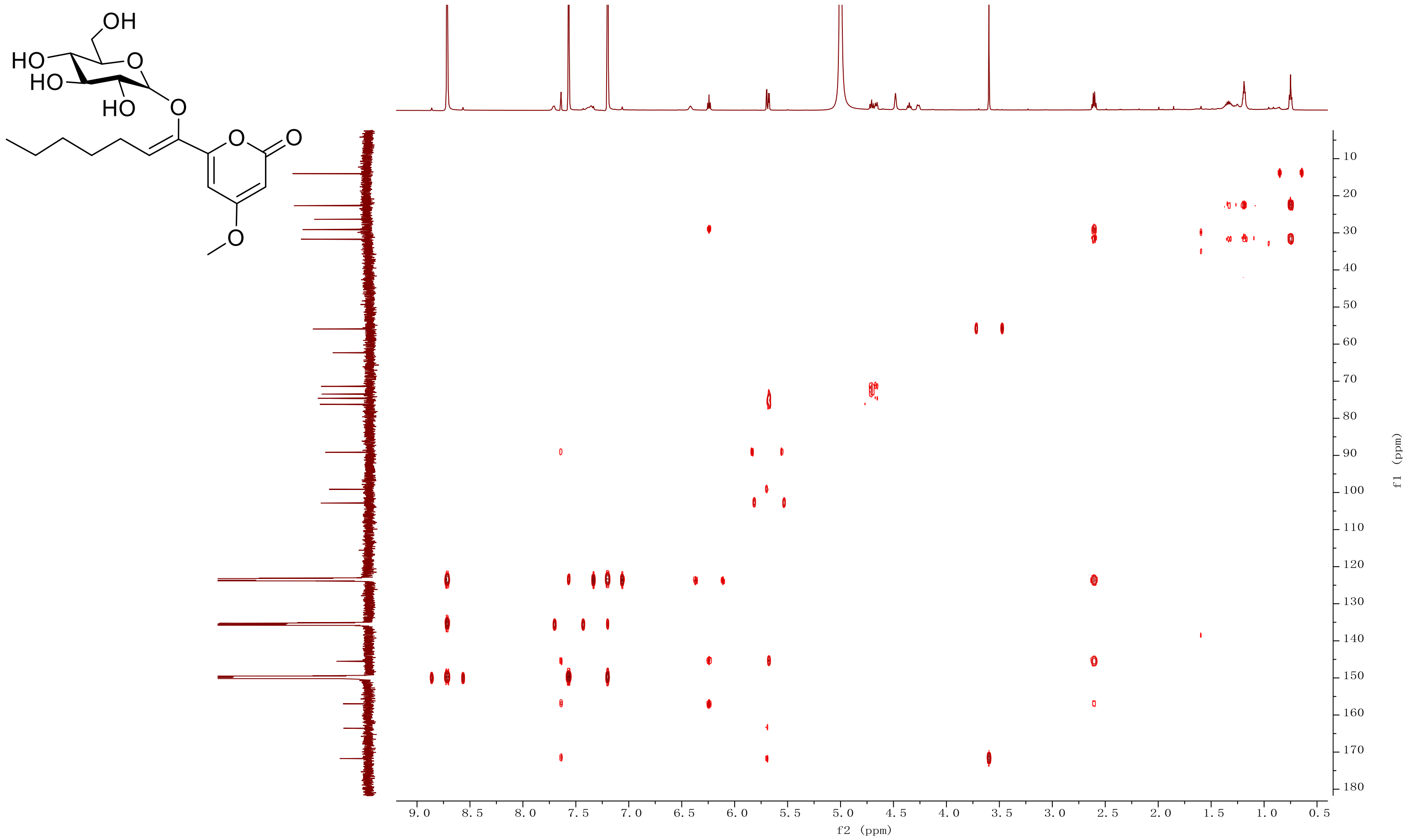

Fig. S26 HMBC spectrum of **2** in pyridine-d_5 (600 MHz).

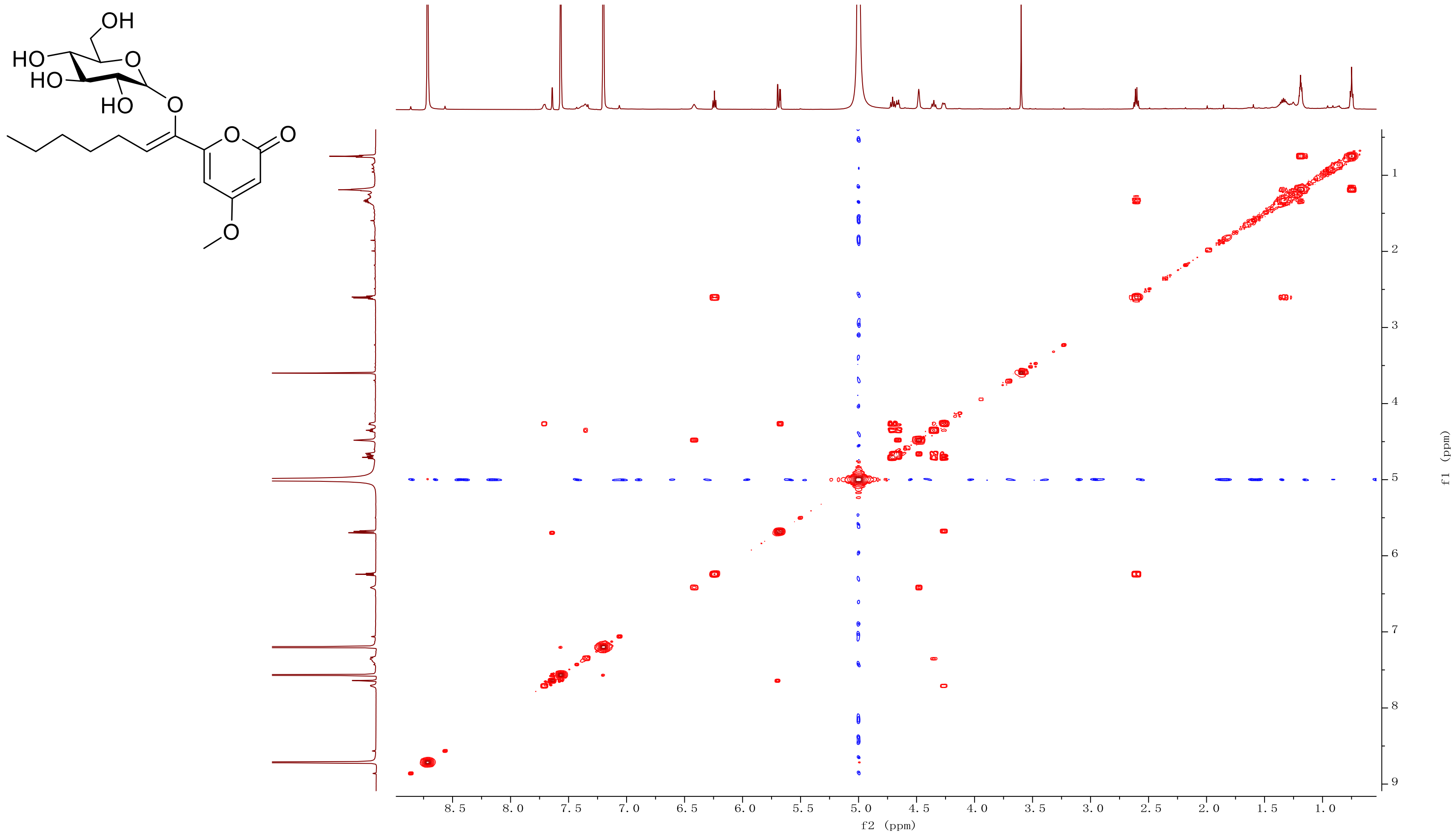

Fig. S27 ^{1}H–^{1}H COSY spectrum of **2** in pyridine-d_5 (600 MHz).

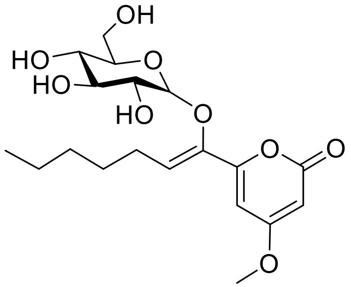

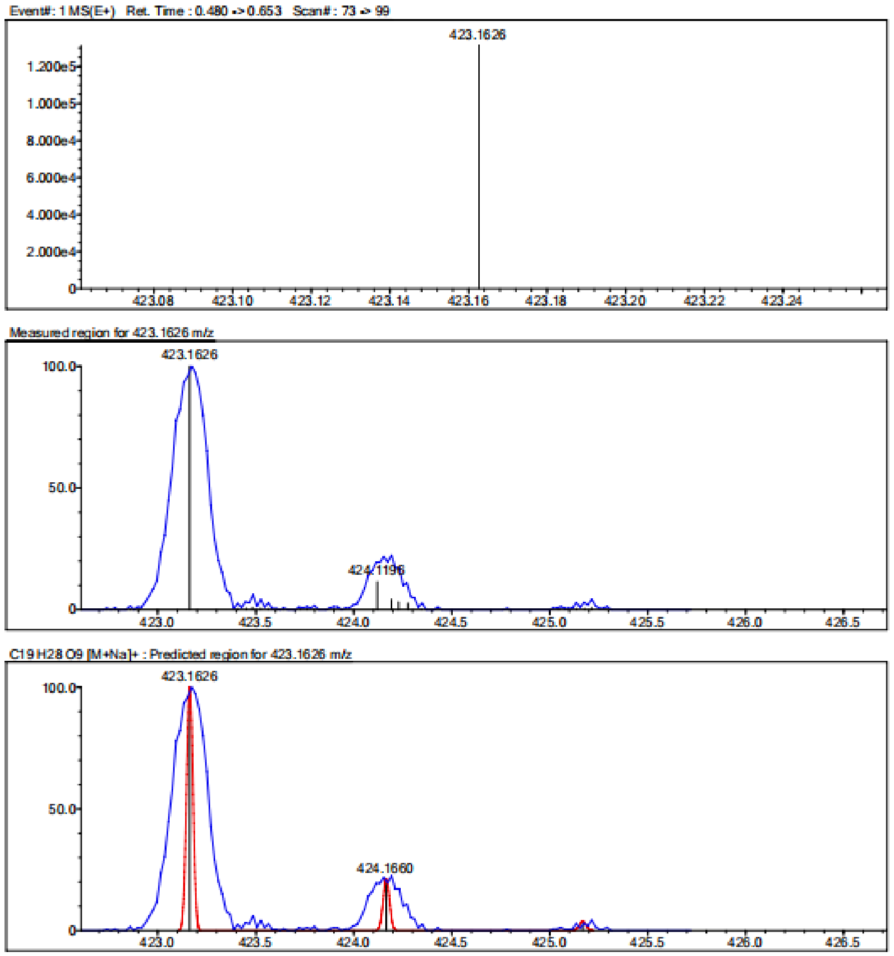

Formula (M)	Ion	Meas. m/z	Pred. m/z	Df. (mDa)	Df. (ppm)	DBE
C19 H28 O9	[M+Na]+	423.1626	423.1626	0.0	0.00	6.0

Fig. S28 HRESIMS spectrum of **2**.

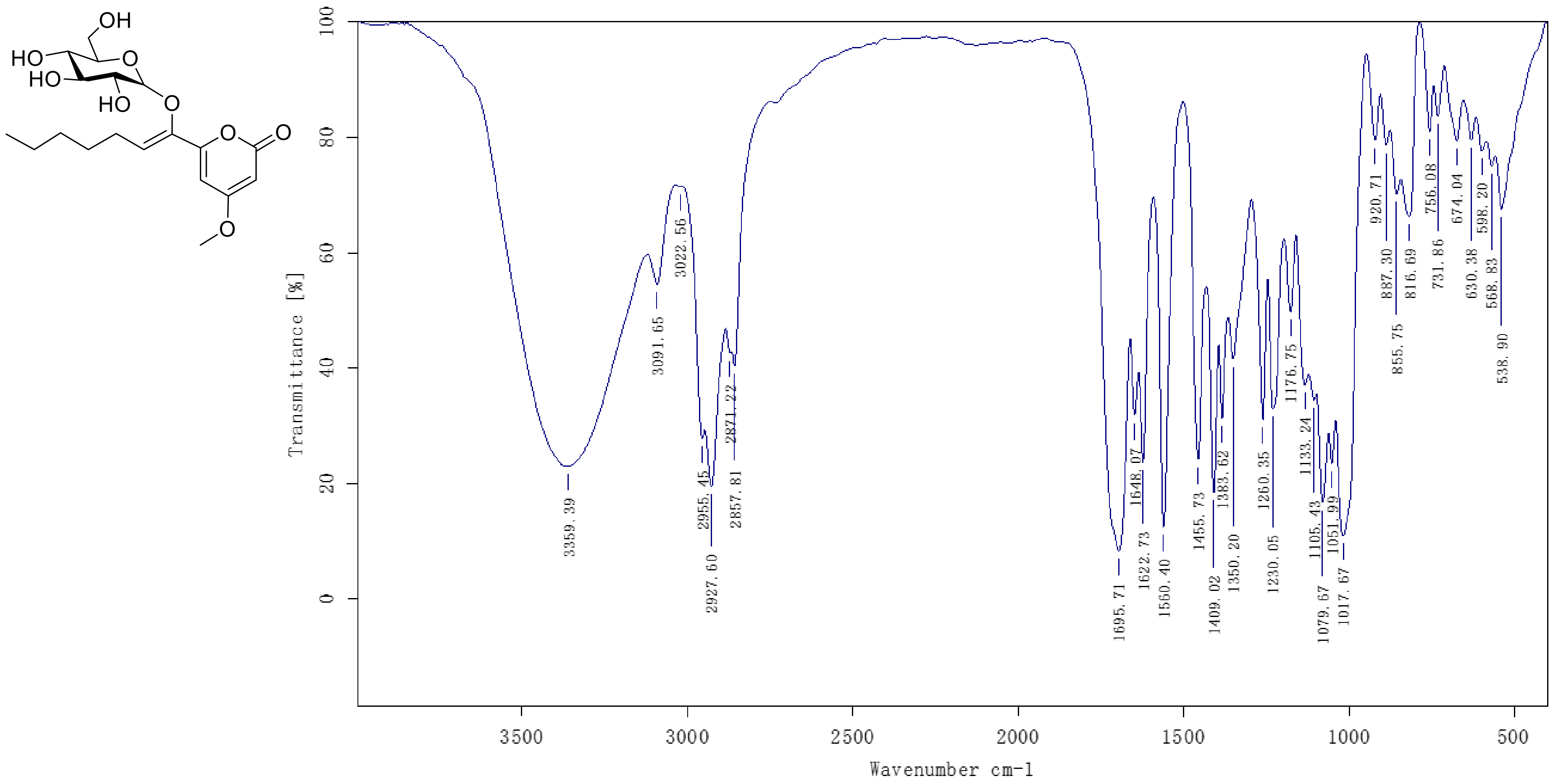

Fig. S29 IR spectrum of **2**.

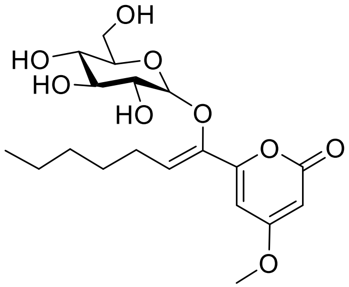

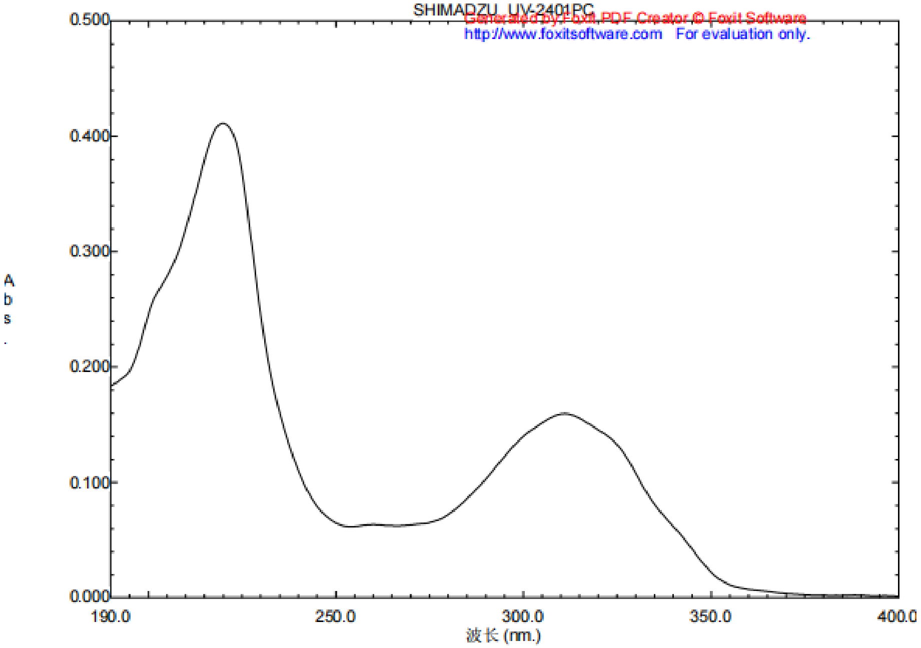

Fig. S30 UV spectrum of **2**.

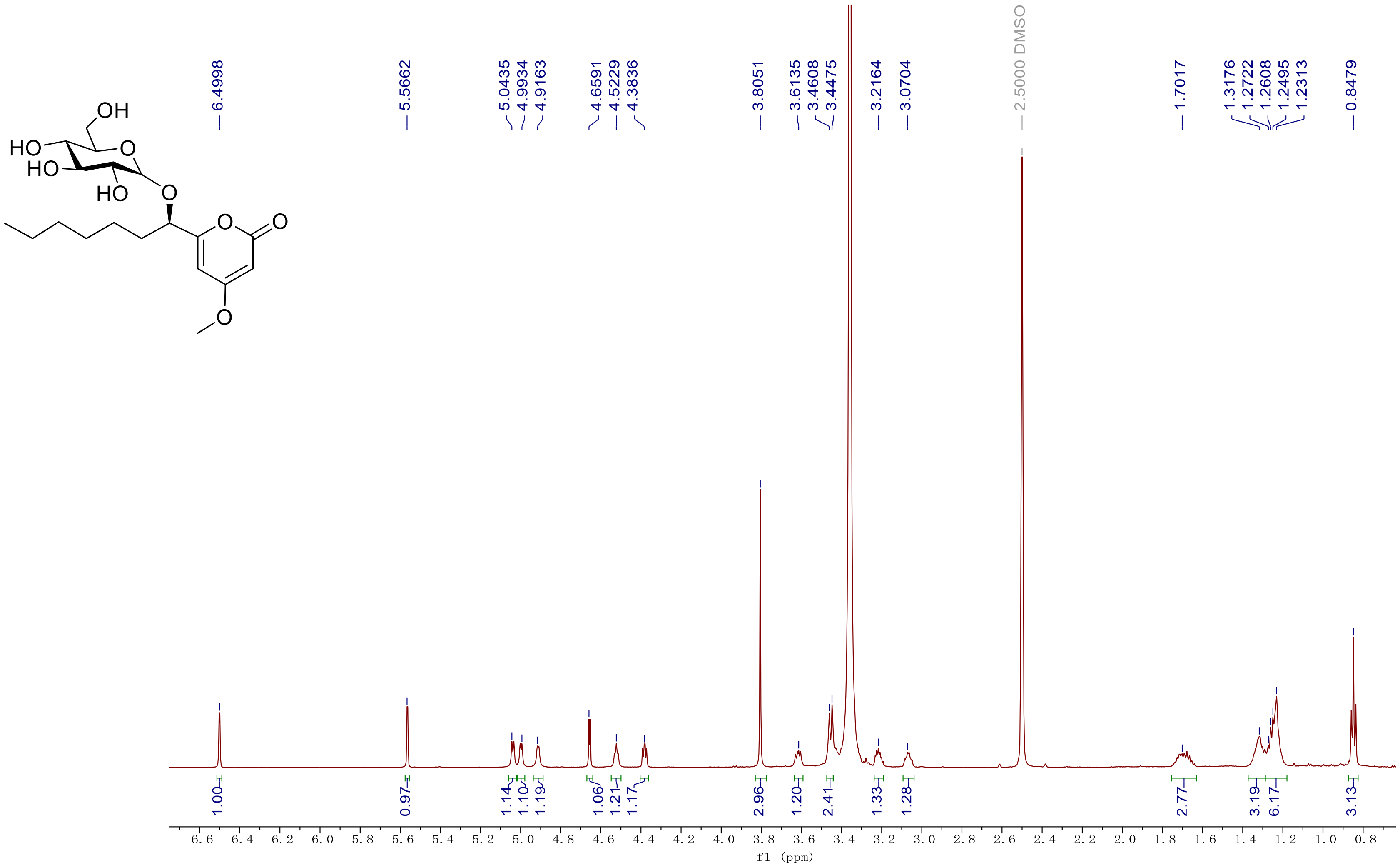

Fig. S31 ^{1}H NMR spectrum of **3** in DMSO-d_6 (600 MHz).

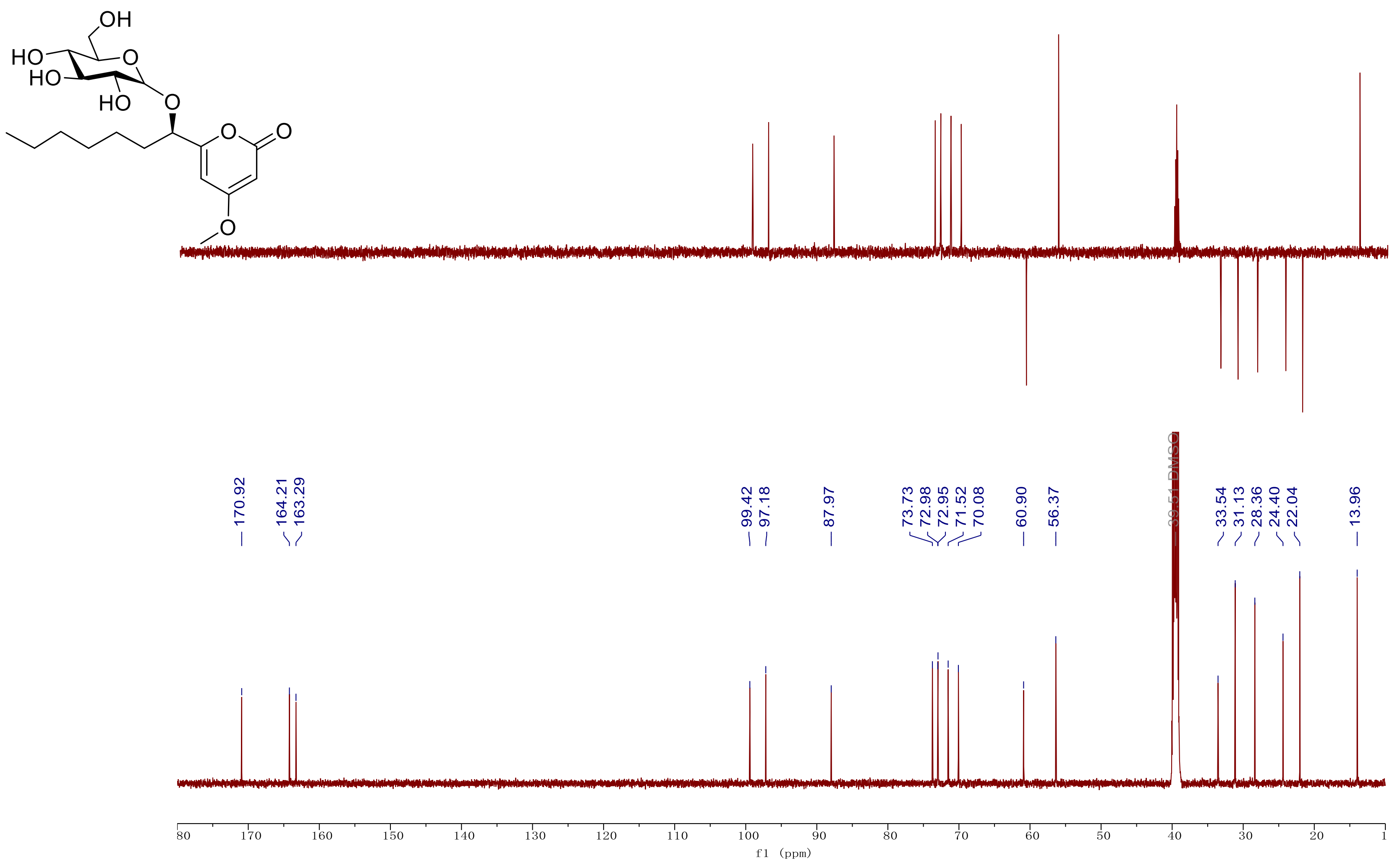

Fig. S32 ^{13}C NMR spectrum of **3** in DMSO-d_6 (600 MHz).

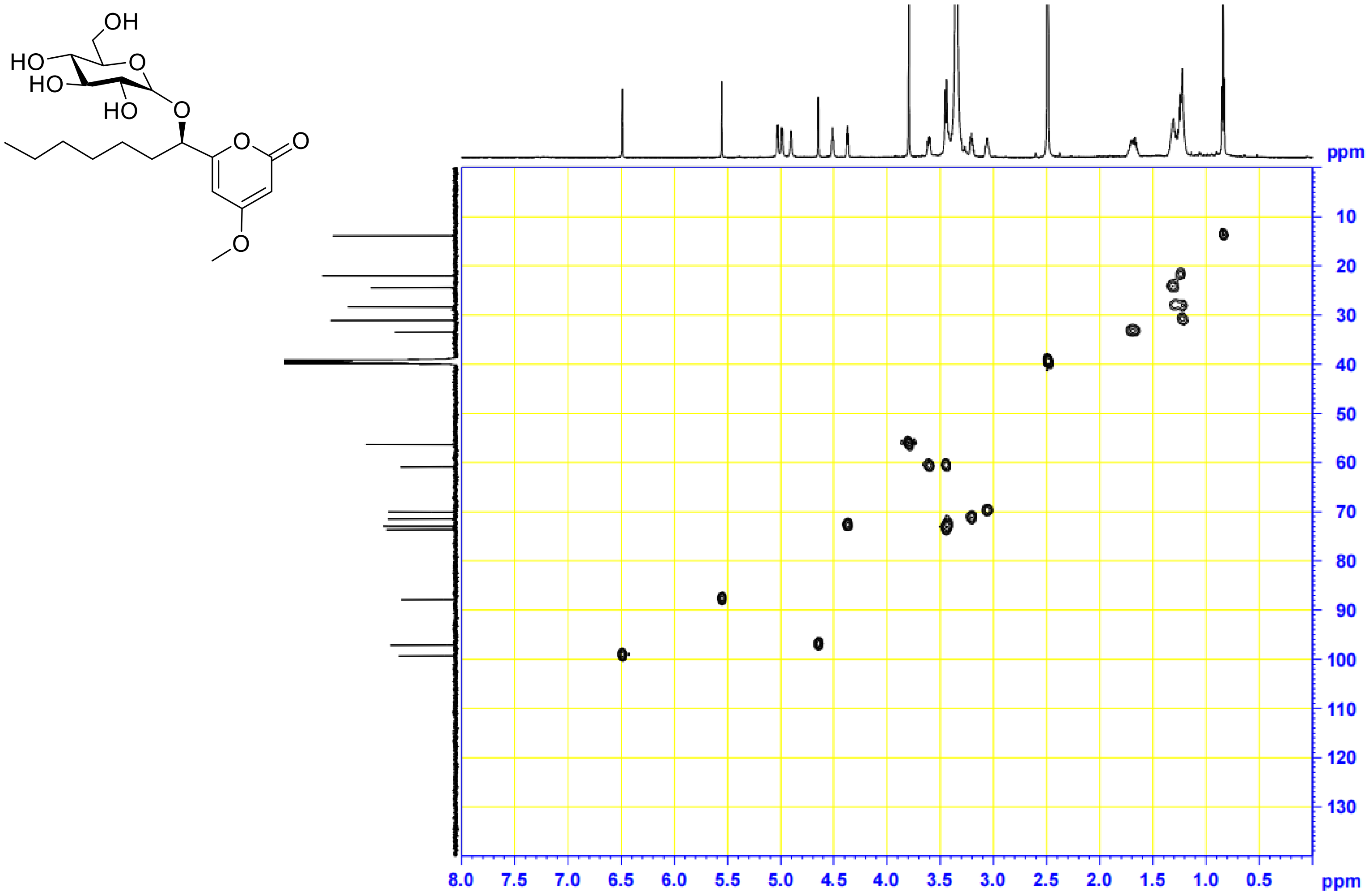

Fig. S33 HSQC spectrum of **3** in DMSO-d_6 (600 MHz).

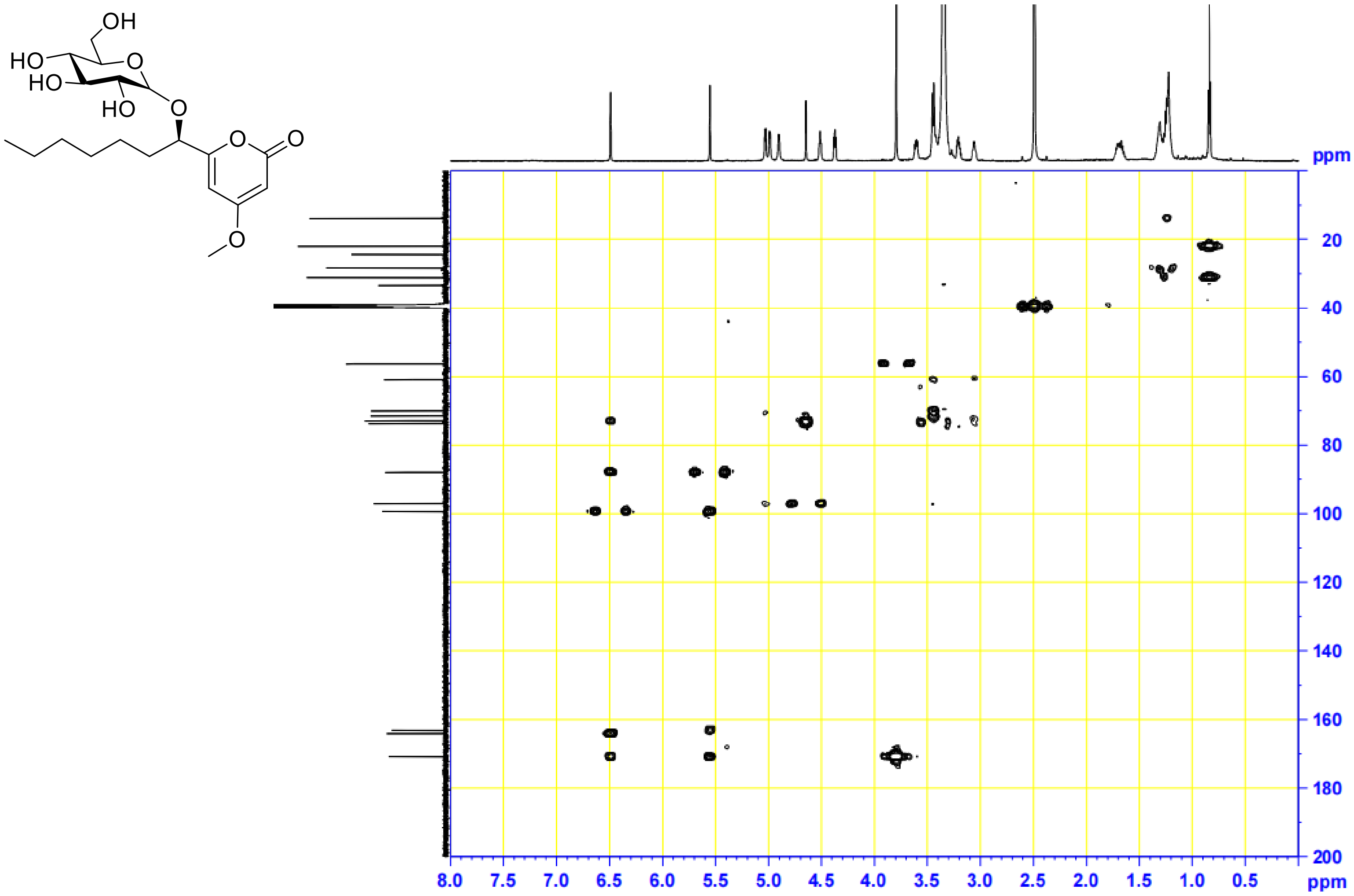

Fig. S34 HMBC spectrum of **3** in DMSO-d_6 (600 MHz).

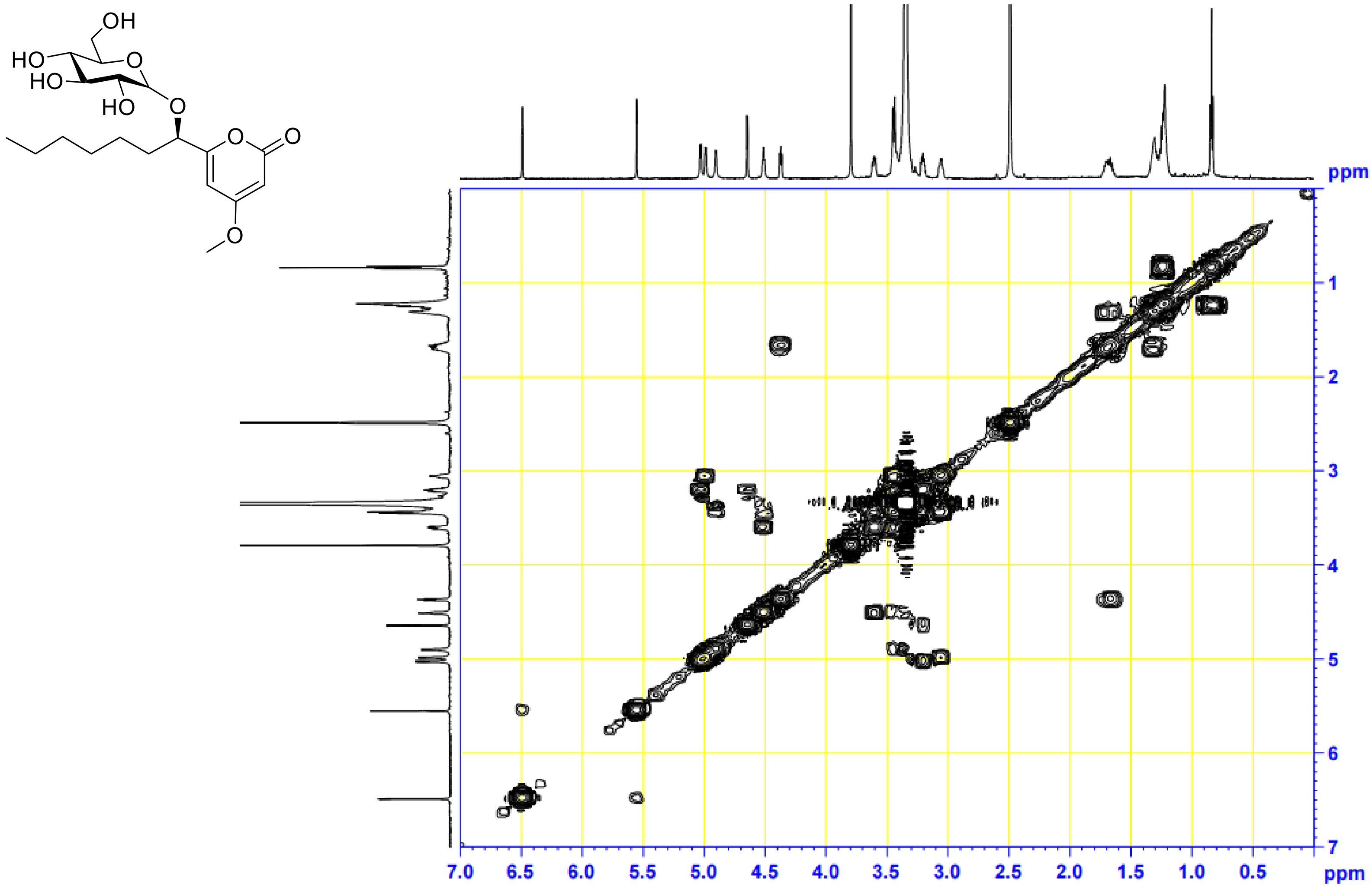

Fig. S35 ^{1}H-^{1}H COSY spectrum of **3** in DMSO-d_6 (600 MHz).

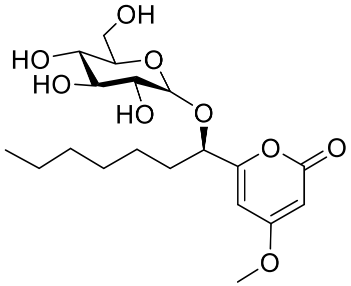

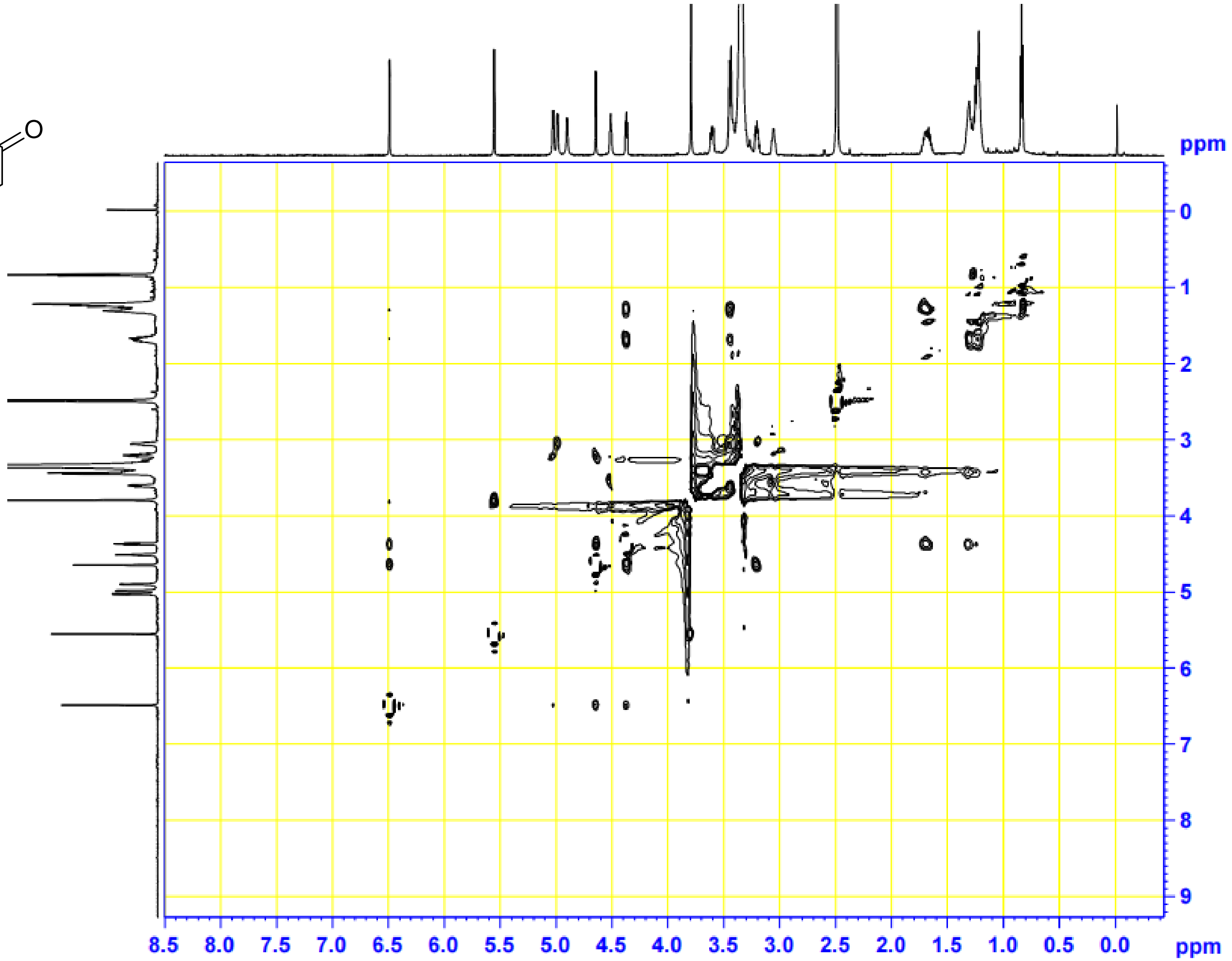

Fig. S36 ROESY spectrum of **3** in DMSO-d_6 (600 MHz).

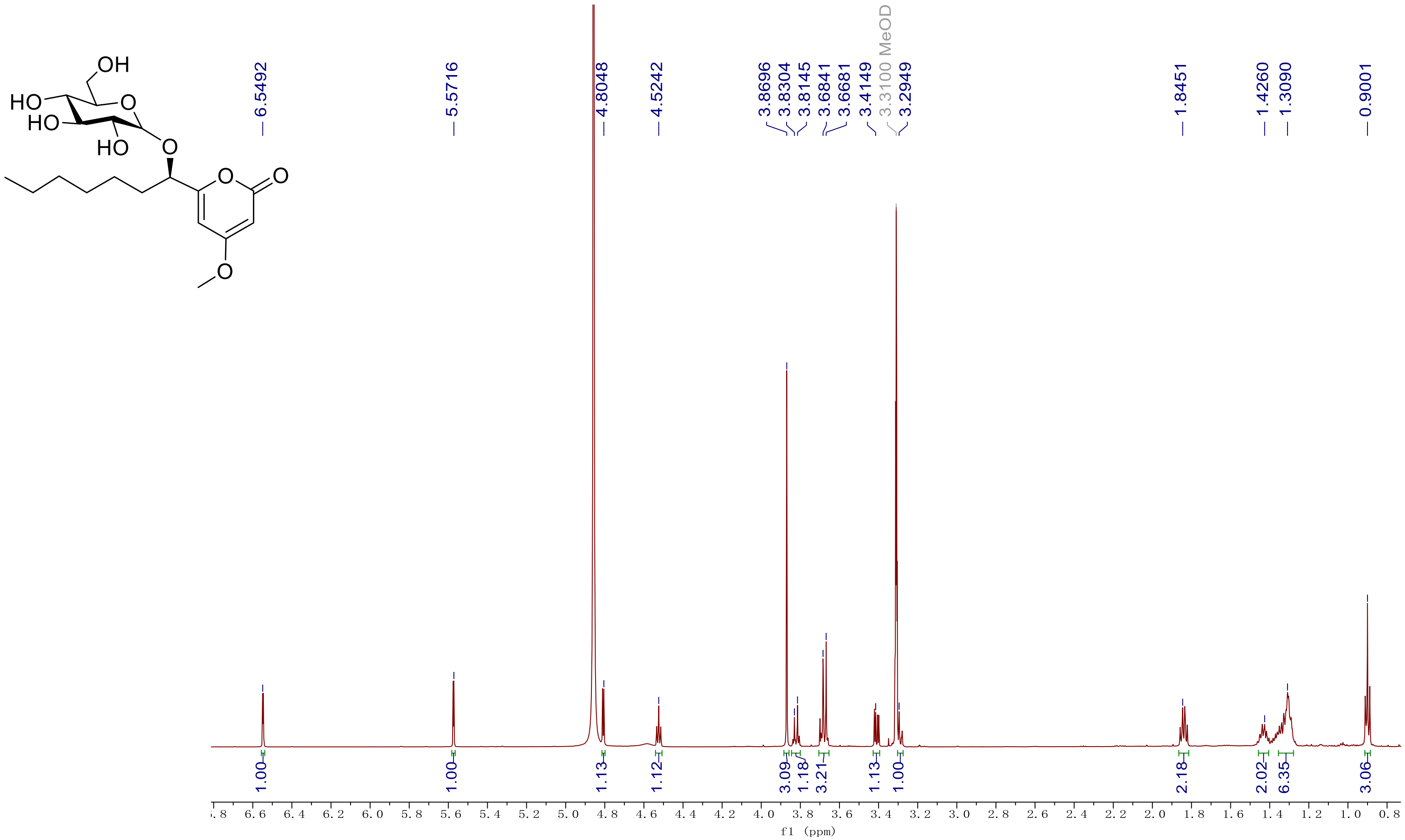

Fig. S37 ^{1}H NMR Spectrum of **3** in methanol-d_4 (600 MHz)

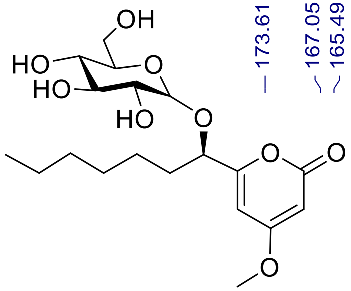

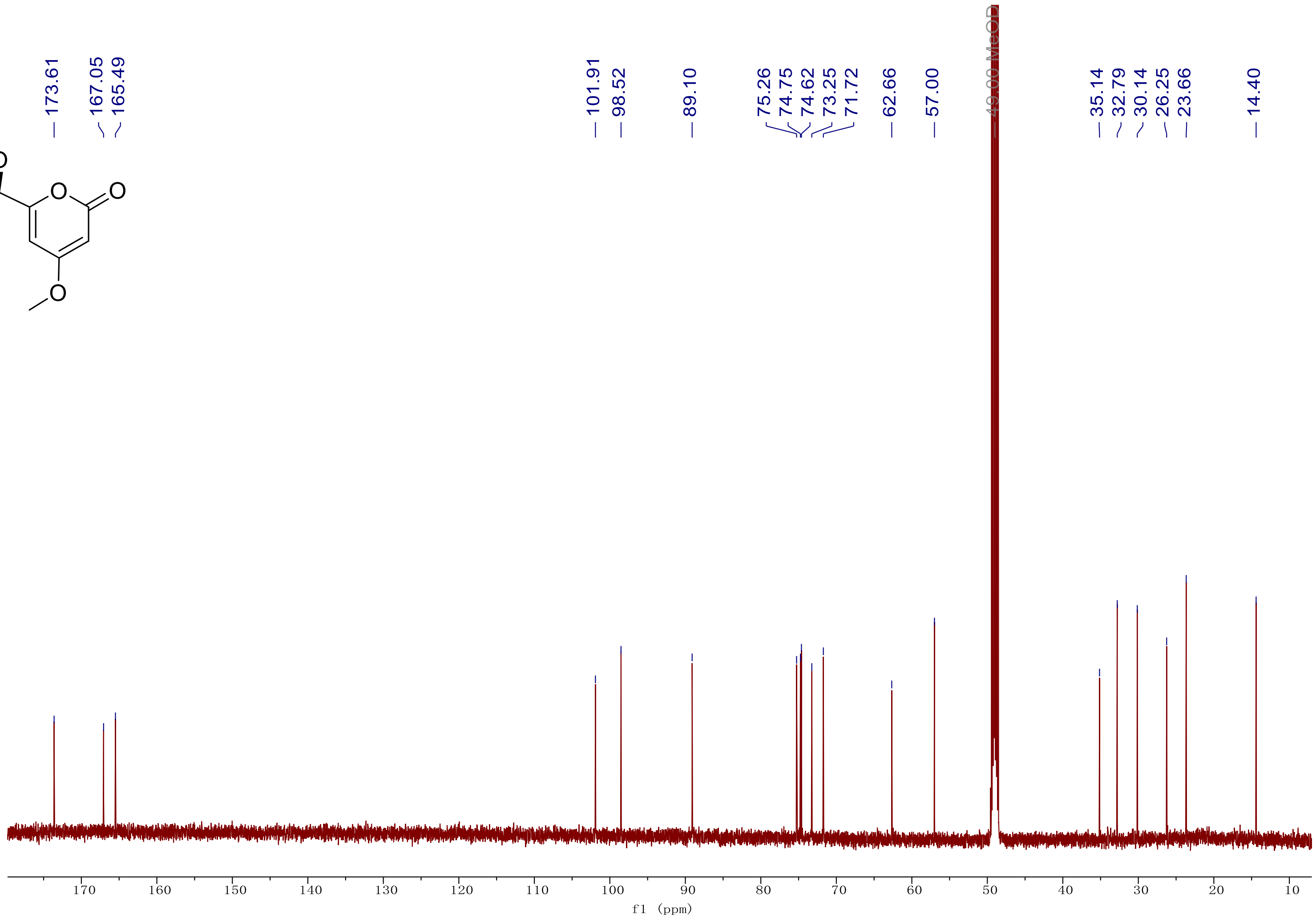

Fig. S38 ^{13}C NMR spectrum of **3** in methanol-d_4 (600 MHz).

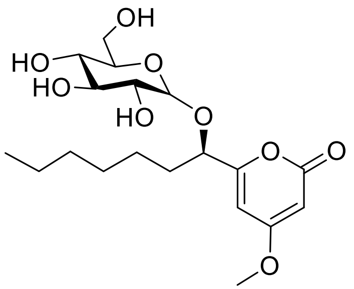

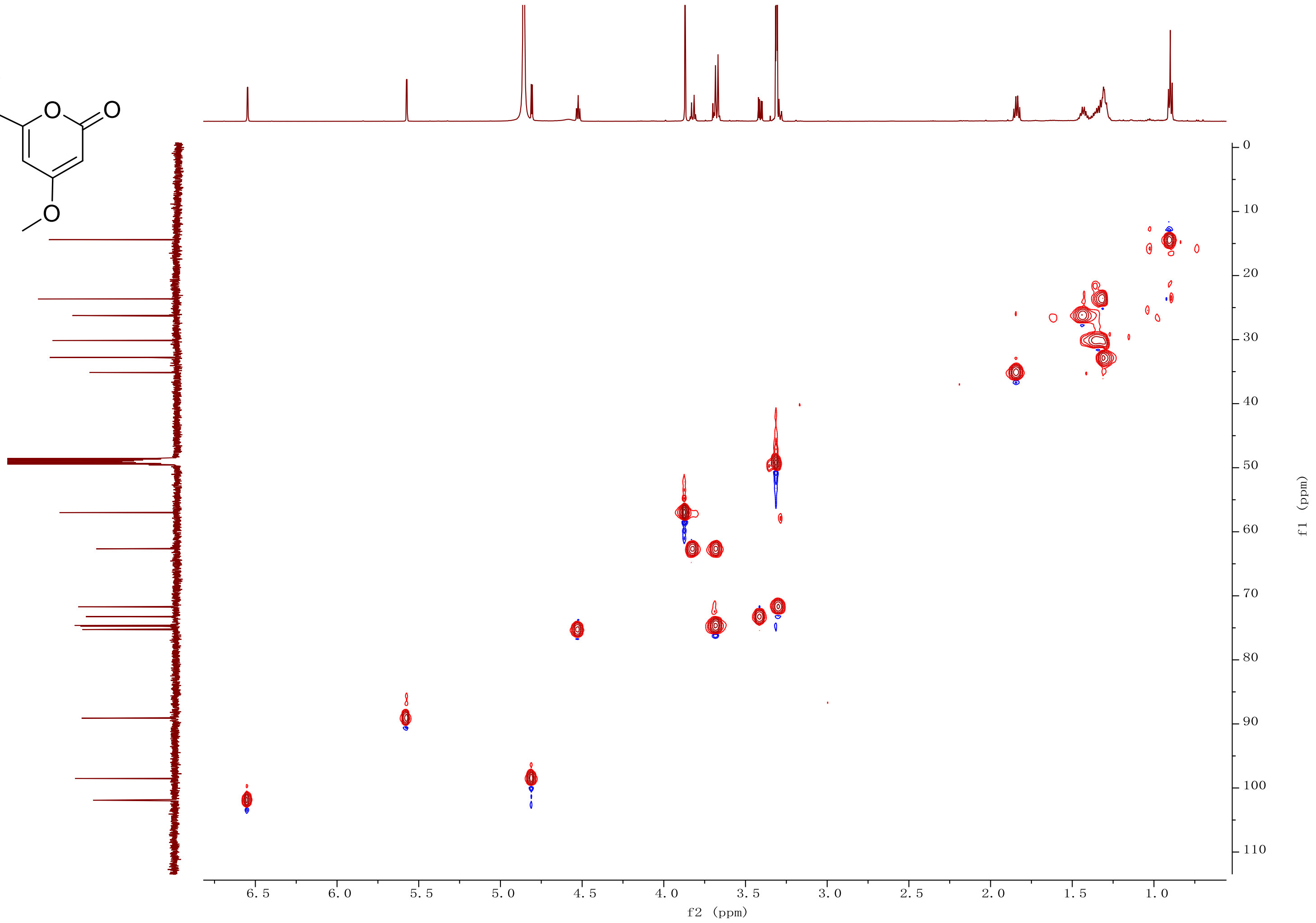

Fig. S39 HSQC spectrum of **3** in methanol-d_4 (600 MHz).

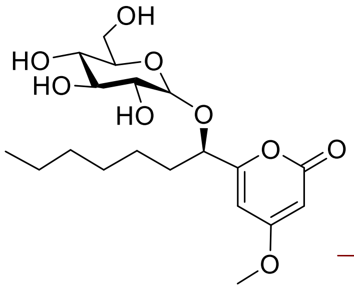

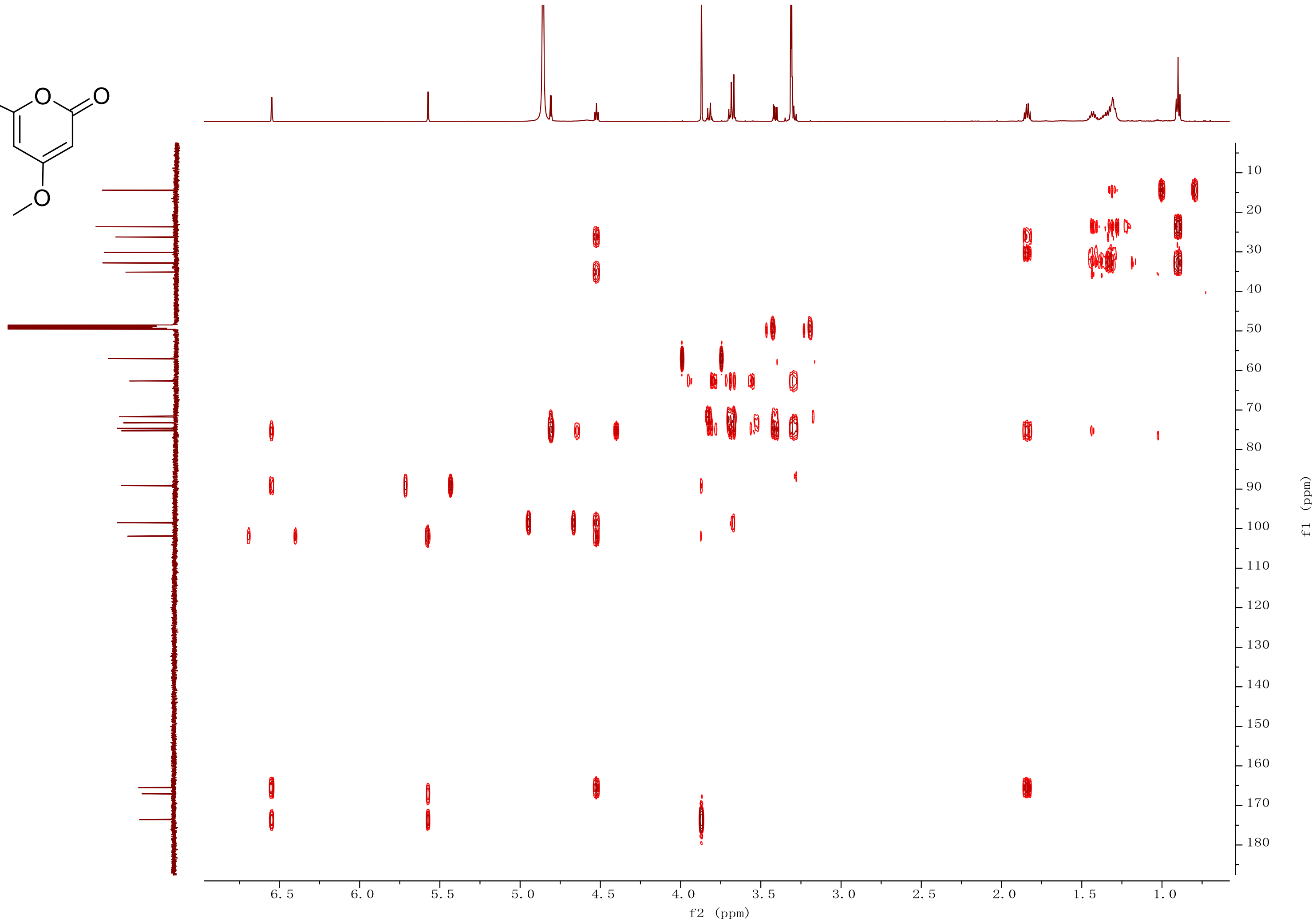

Fig. S40 HMBC spectrum of **3** in methanol-d_4 (600 MHz).

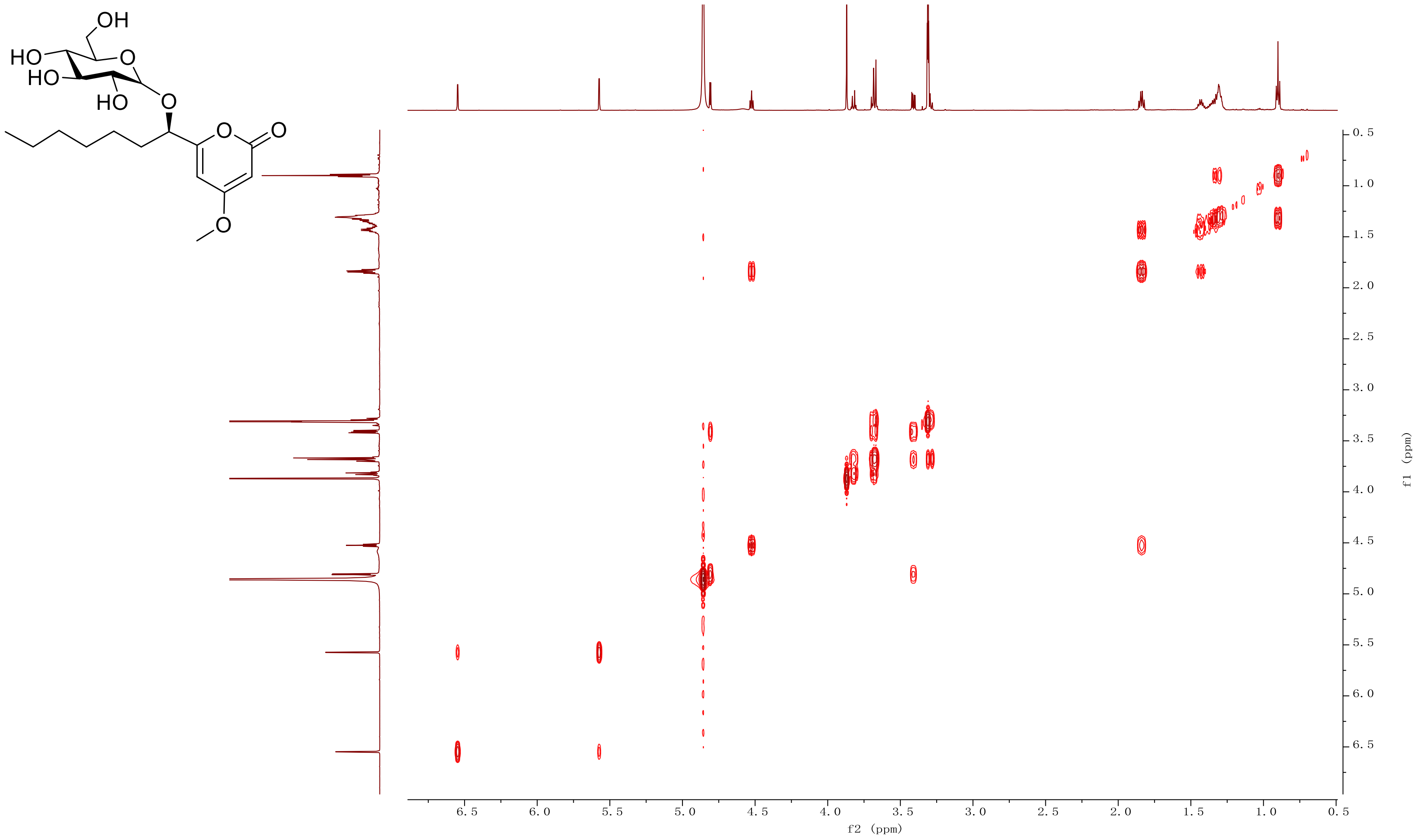

Fig. S41 ^{1}H–^{1}H COSY spectrum of **3** in methanol-d_4 (600 MHz).

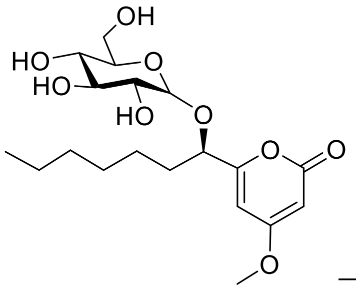

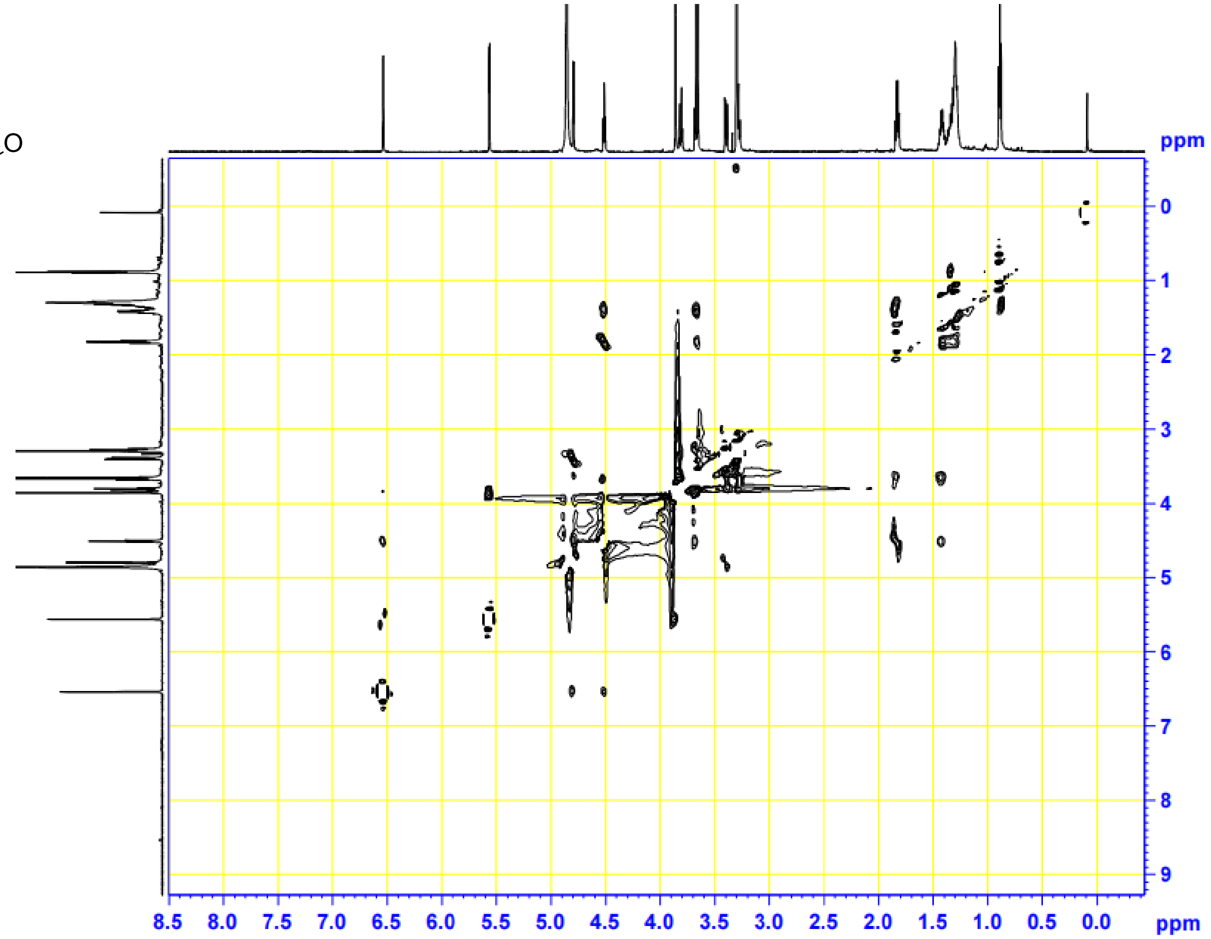

Fig. S42 ROESY spectrum of **3** in methanol-d_4 (600 MHz).

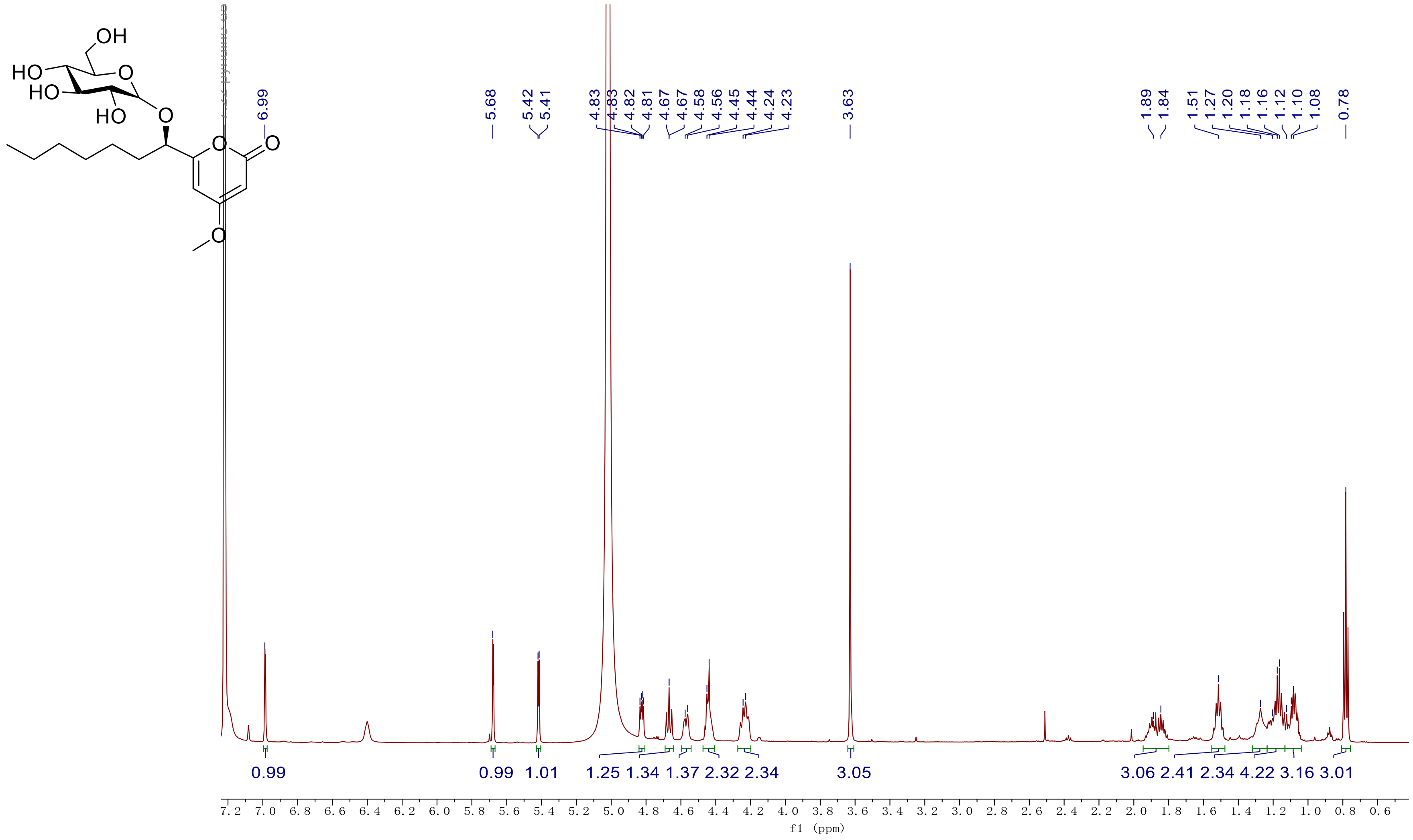

Fig. S43 ^{1}H NMR spectrum of **3** in pyridine-d_5 (600 MHz).

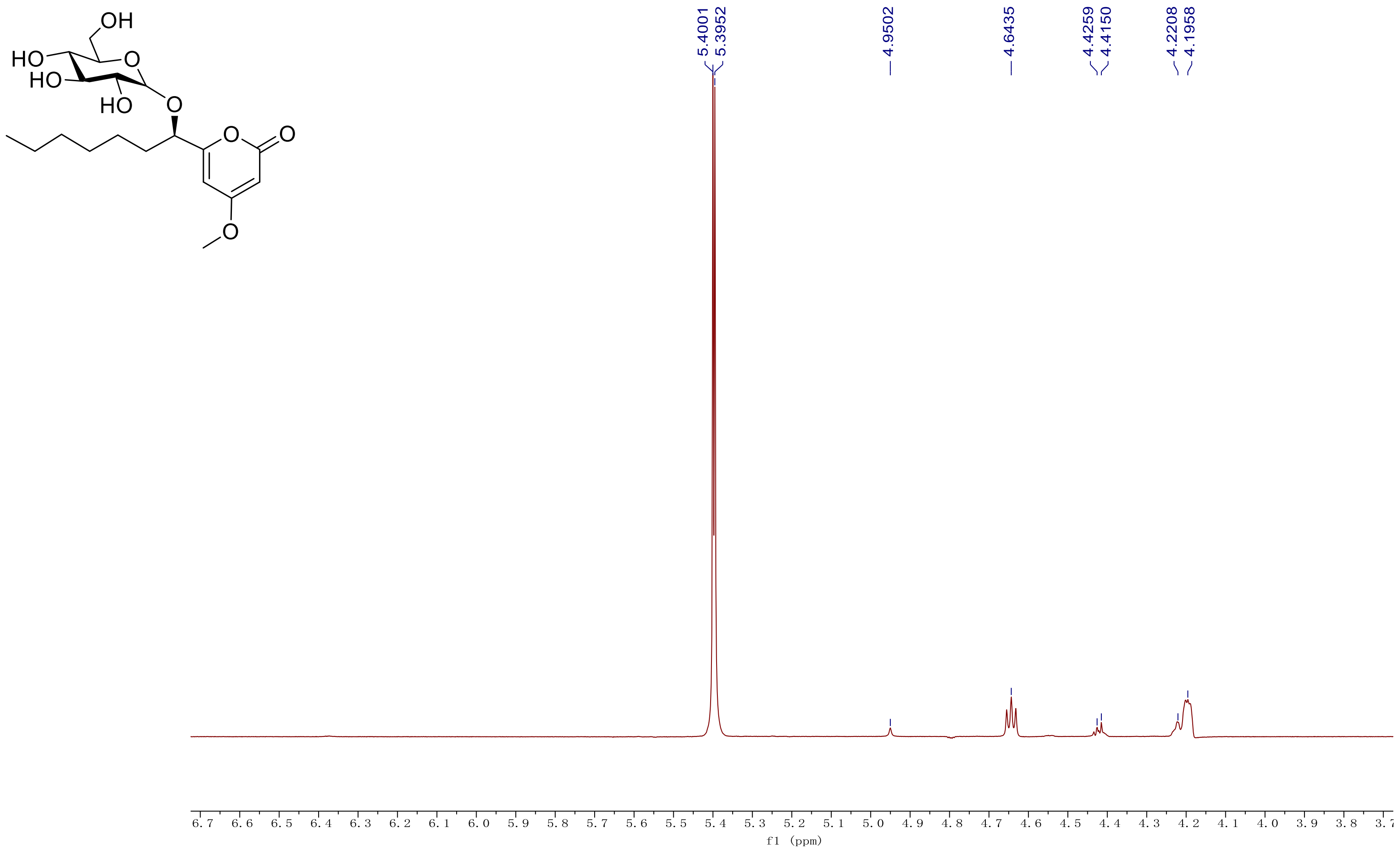

Fig. S44 1D-TOCSY spectrum of **3** in pyridine-d_5 (800 MHz).

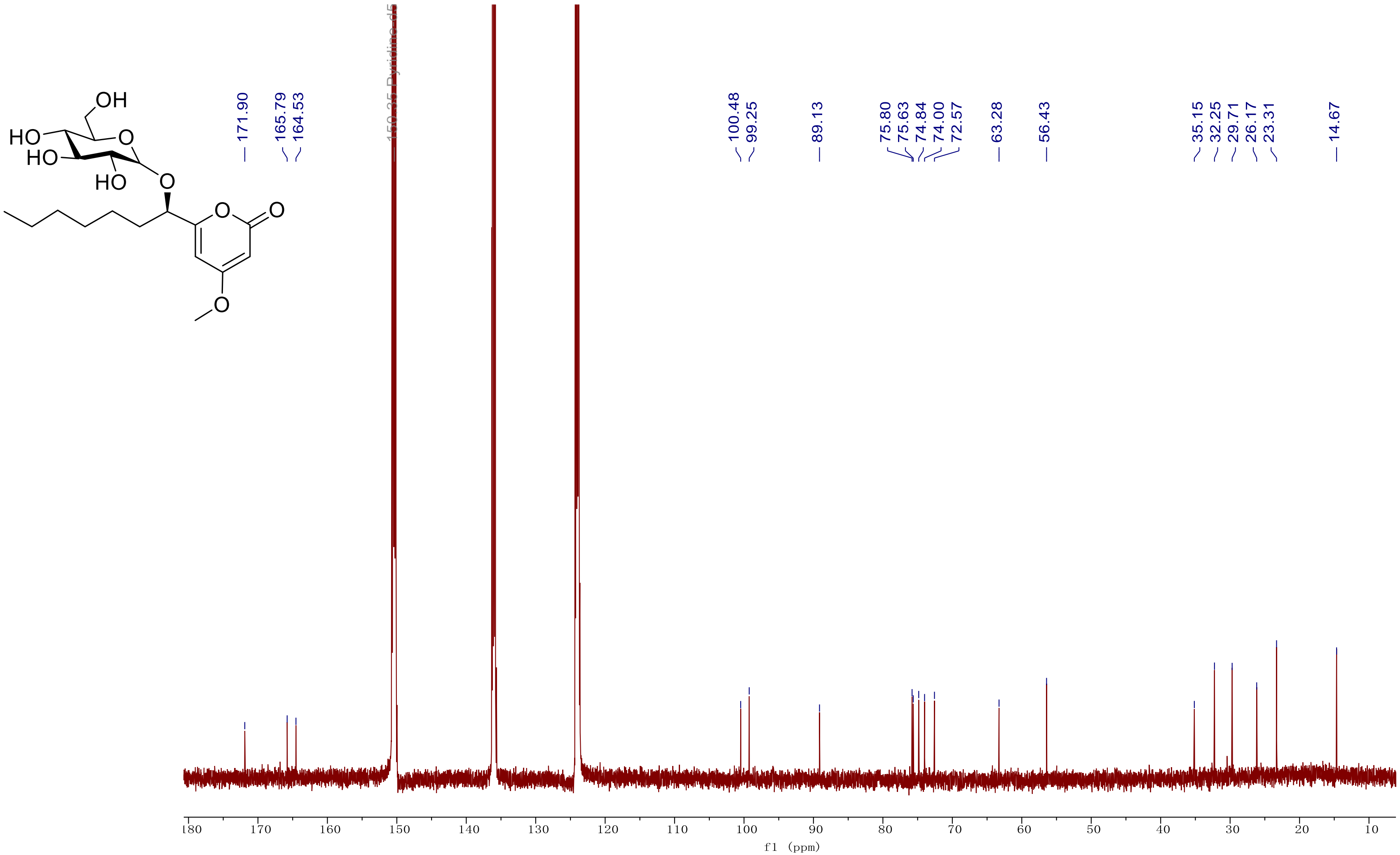

Fig. S45 ^{13}C NMR spectrum of **3** in pyridine-d_5 (150 MHz).

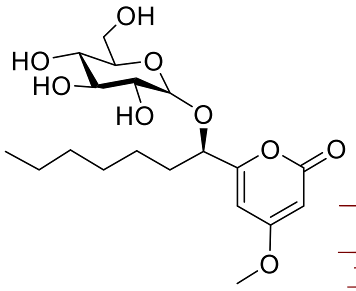

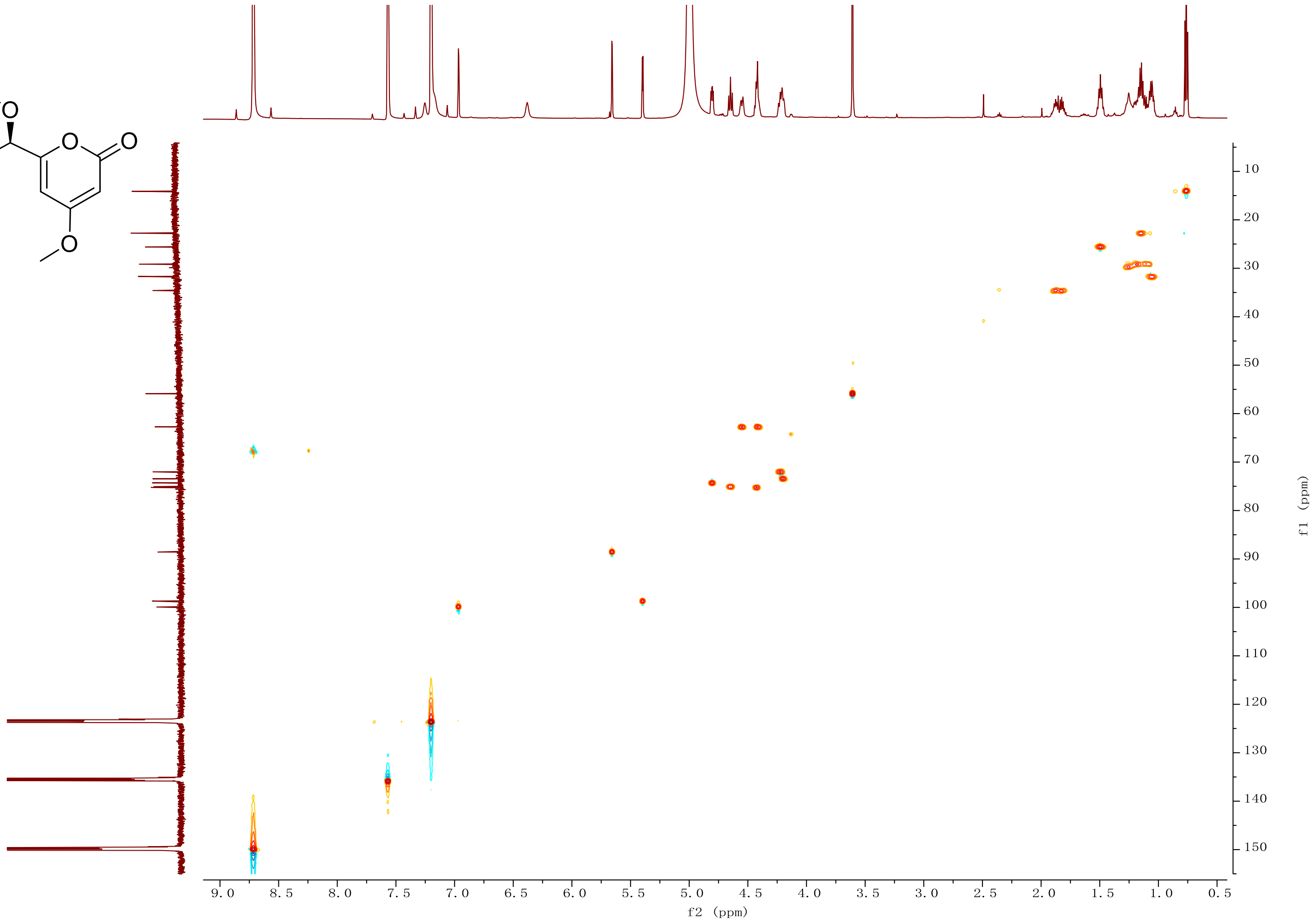

Fig. S46 HSQC spectrum of **3** in pyridine-d_5 (600 MHz).

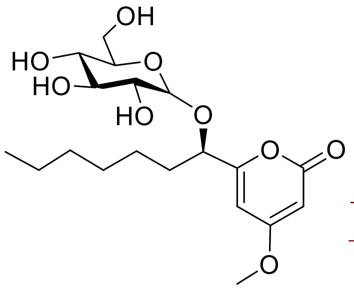

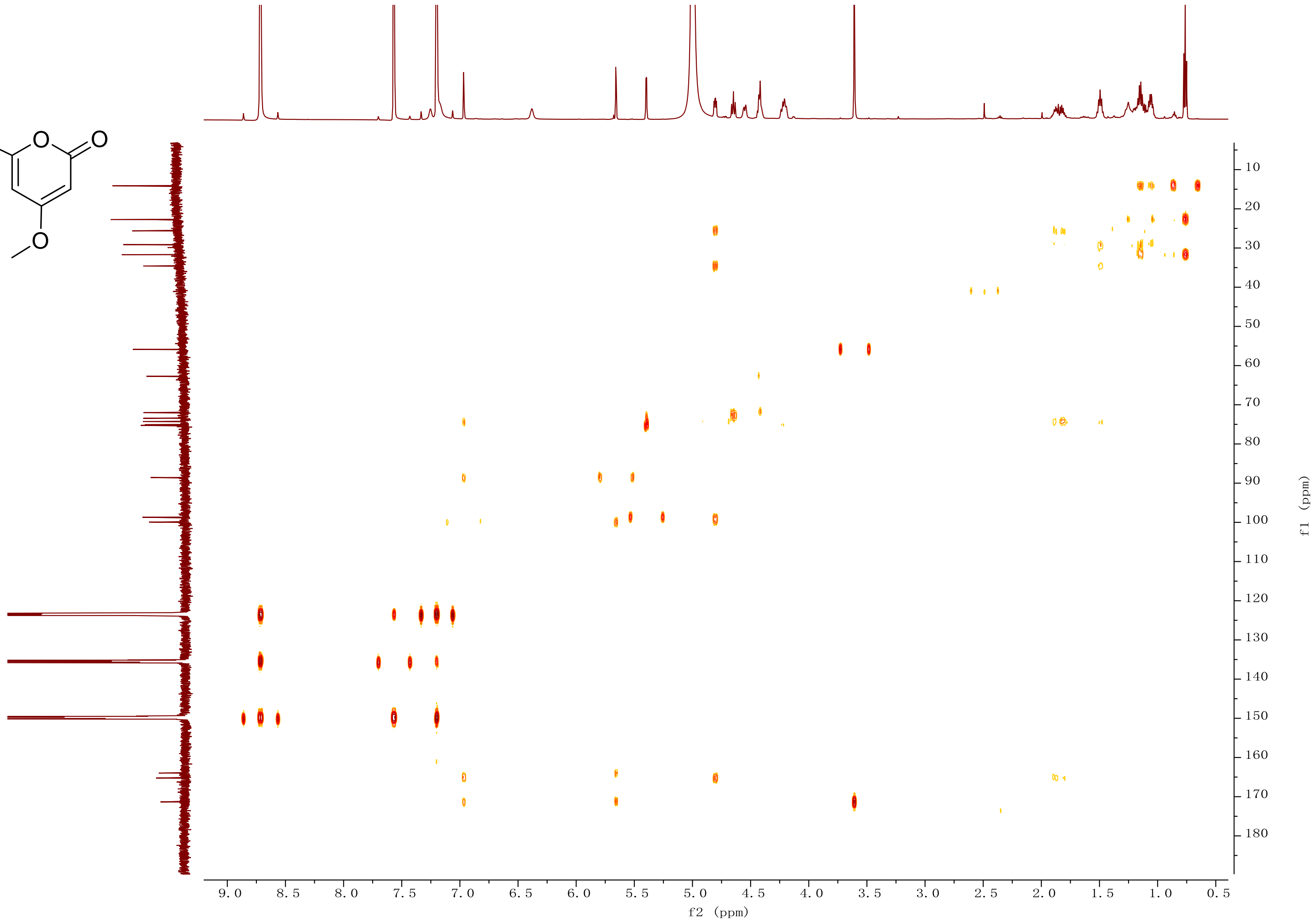

Fig. S47 HMBC spectrum of **3** in pyridine-*d*$_5$ (600 MHz).

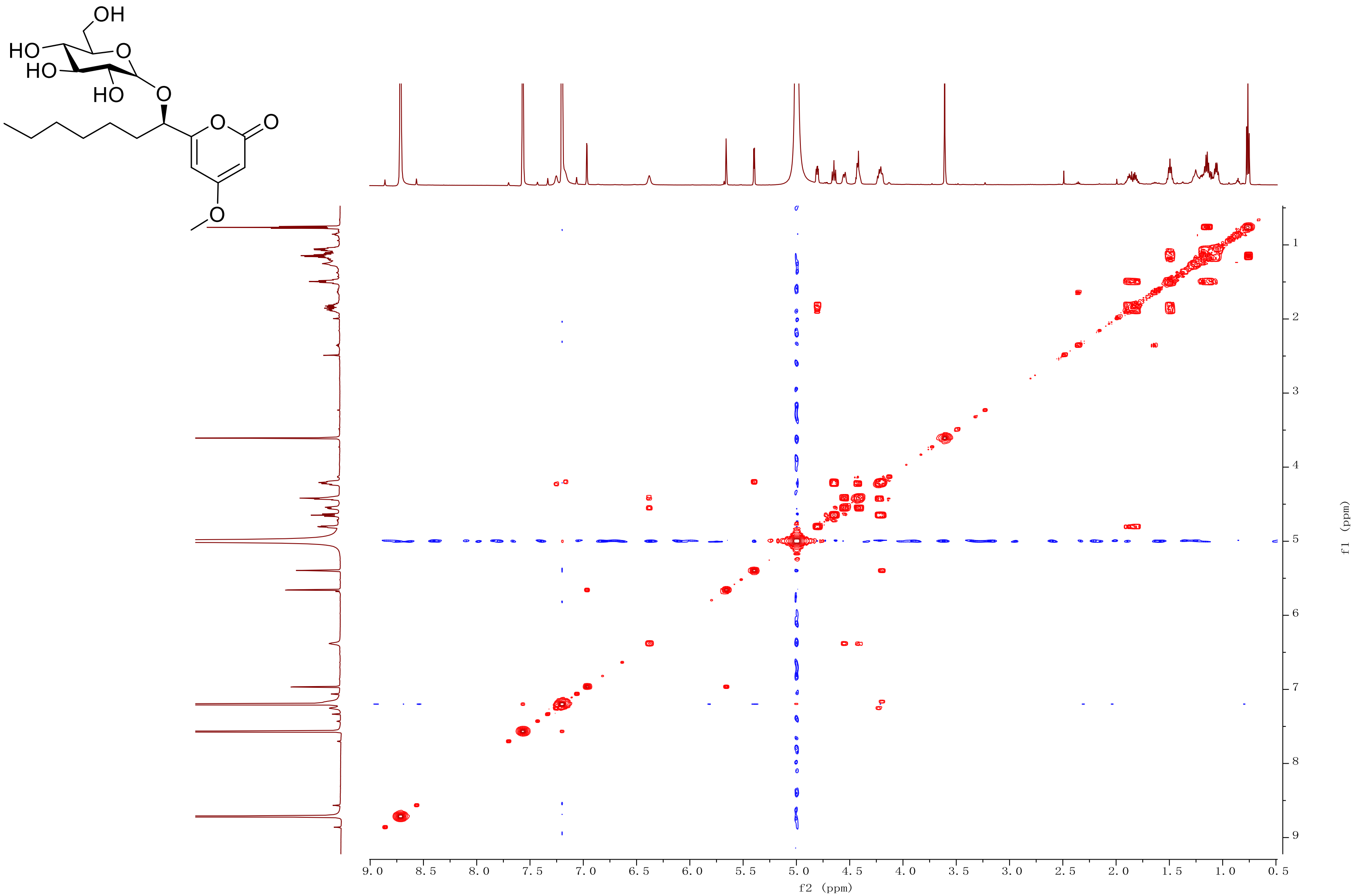

Fig. S48 ^{1}H–^{1}H COSY spectrum of **2** in pyridine-d_5 (600 MHz).

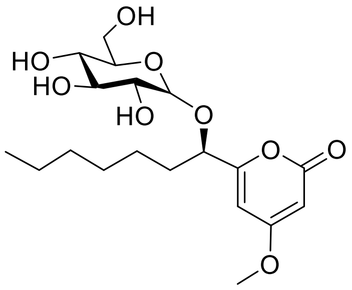

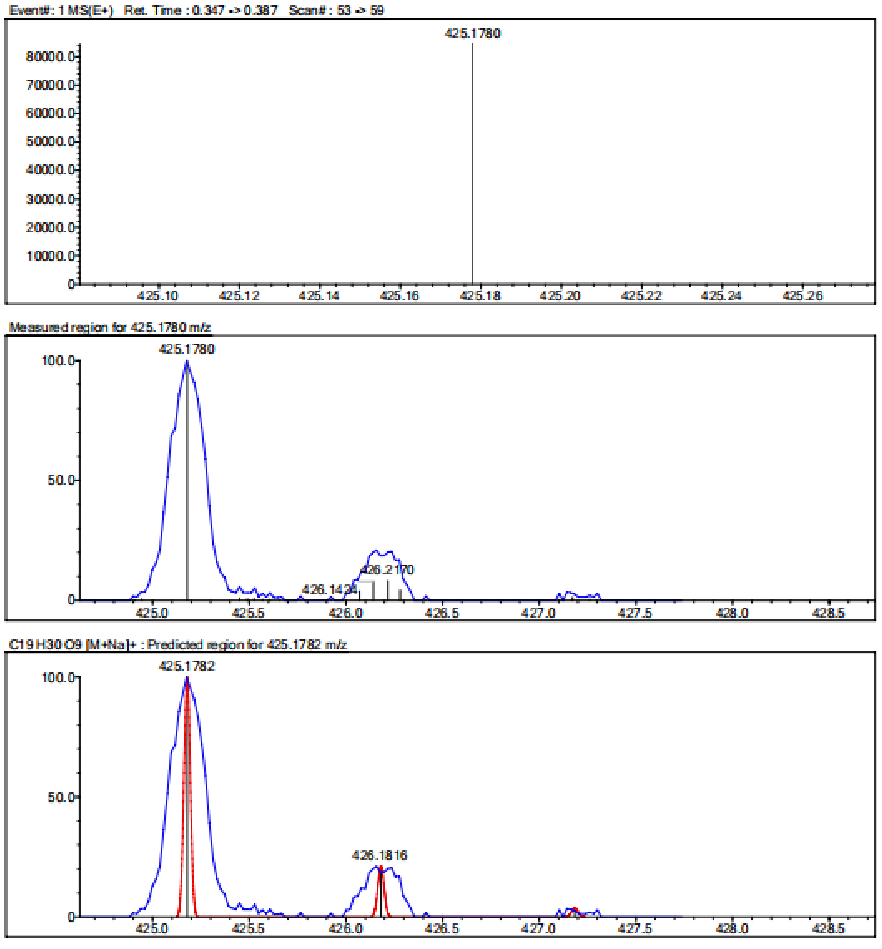

Formula (M)	Ion	Meas. m/z	Pred. m/z	Df. (mDa)	Df. (ppm)	DBE
C19 H30 O9	[M+Na]+	425.1780	425.1782	-0.2	-0.47	5.0

Fig. S49 HRESIMS spectrum of **3**.

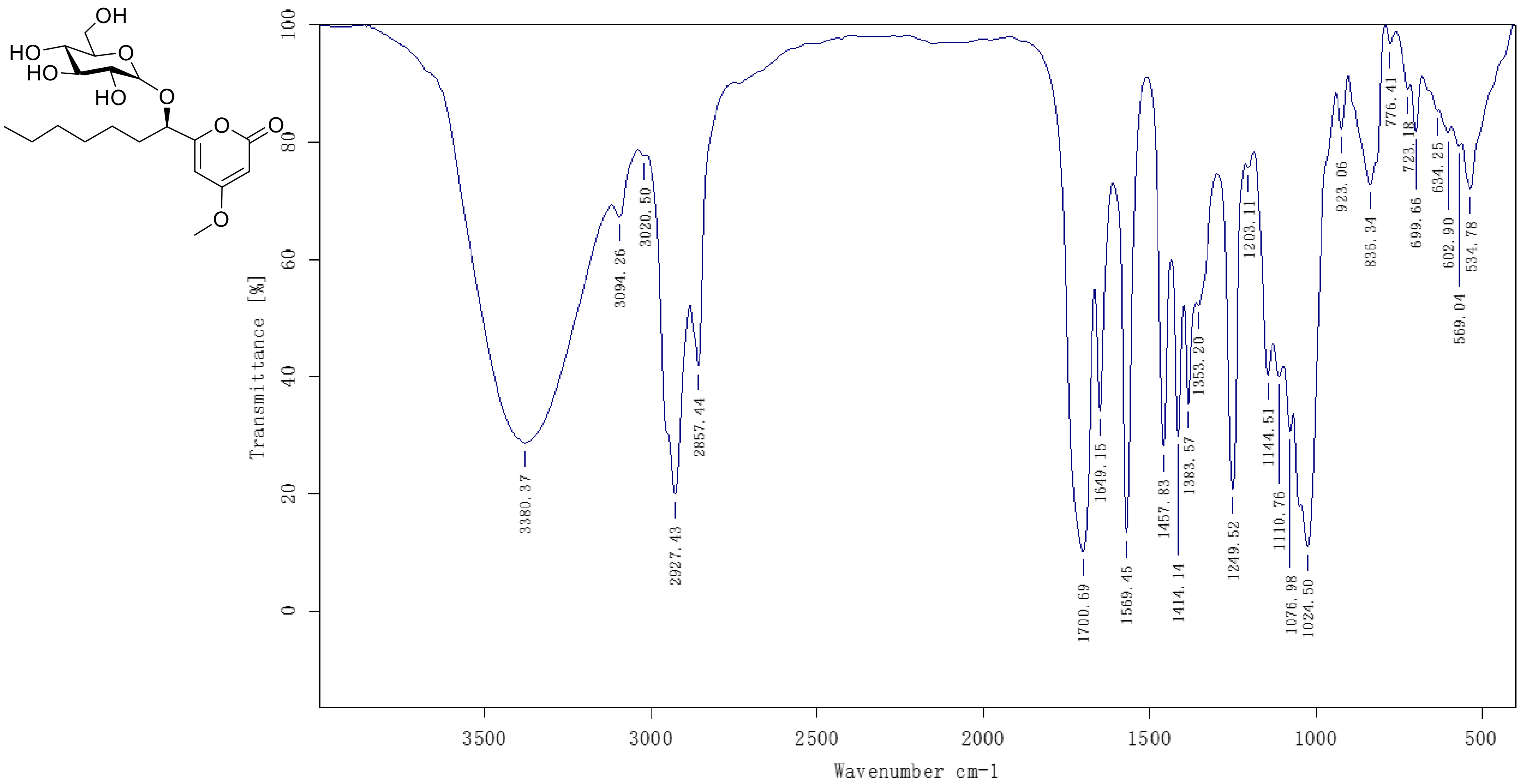

Fig. S50 IR spectrum of **3**.

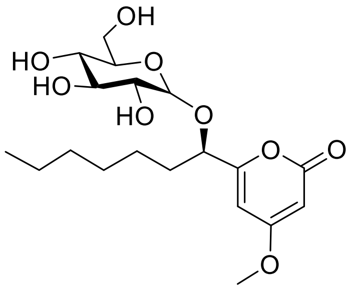

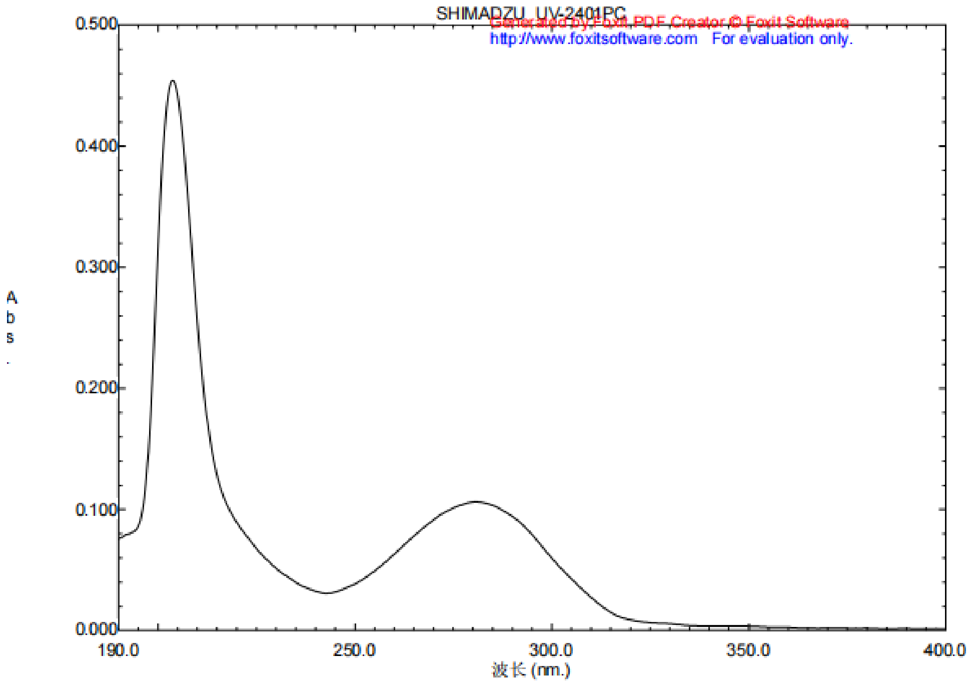

Fig. S51 UV spectrum of **3**.

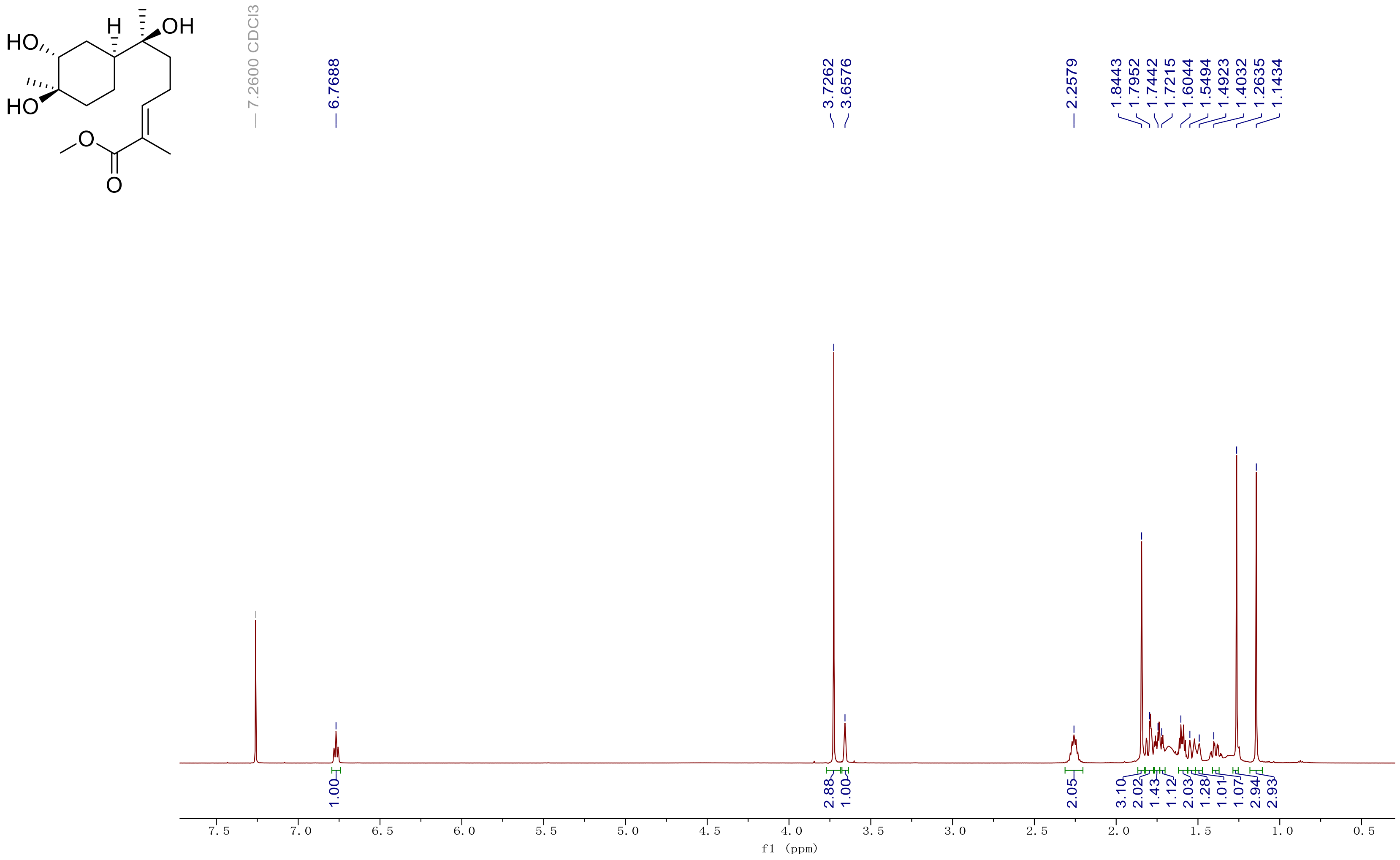

Fig. S52 ^{1}H NMR Spectrum of **4** in chloroform-*d* (600 MHz).

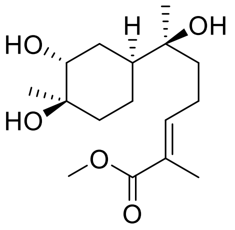

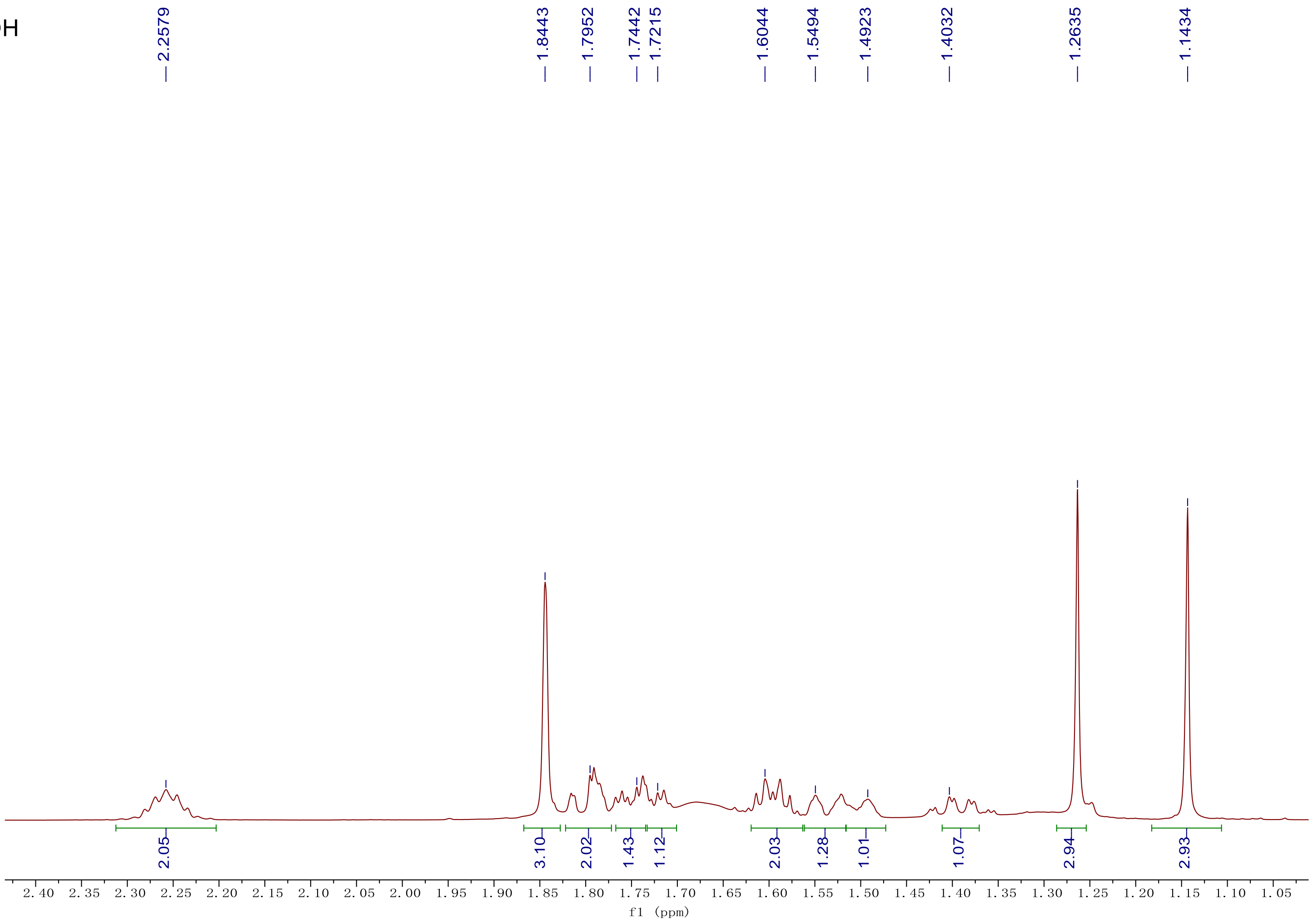

Fig. S53 ^{1}H NMR Spectrum of **4** in chloroform-*d* (600 MHz) (expanded).

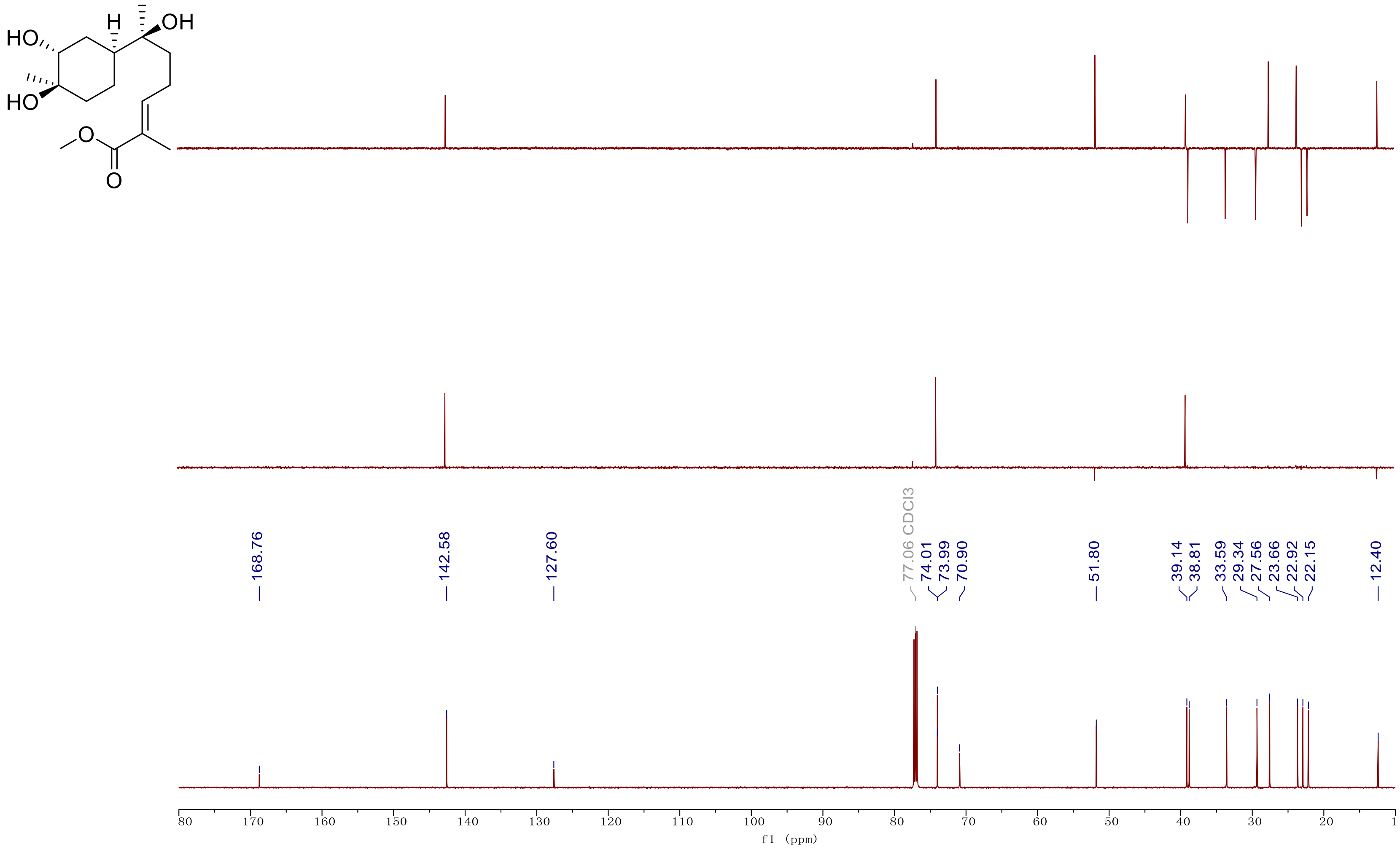

Fig. S54 ^{13}C NMR Spectrum of **4** in chloroform-*d* (150 MHz).

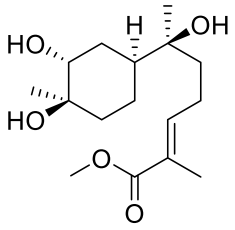

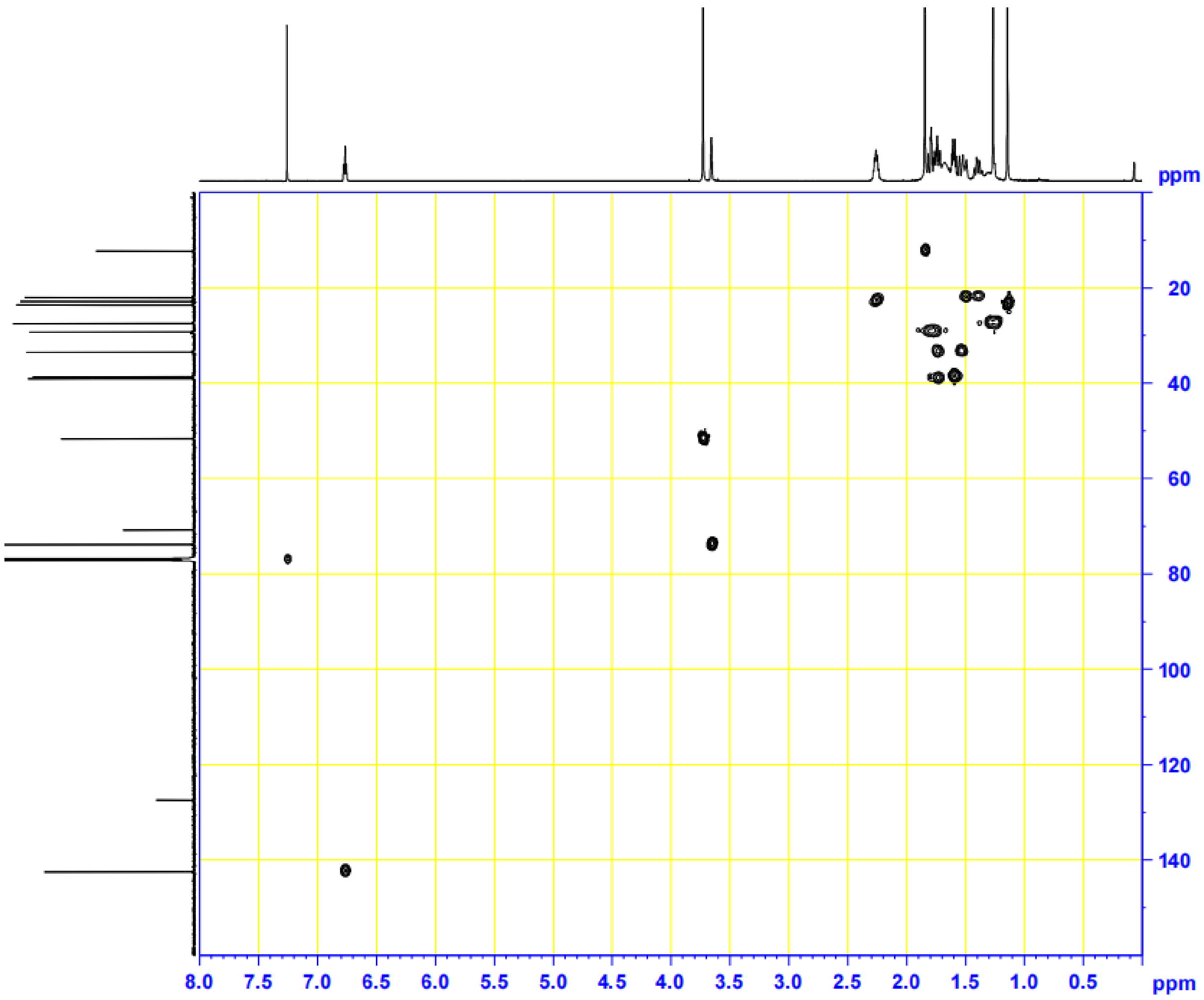

Fig. S55 HSQC Spectrum of **4** in chloroform-*d* (600 MHz).

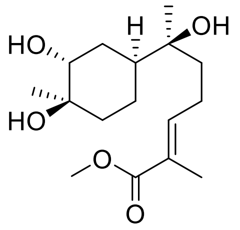

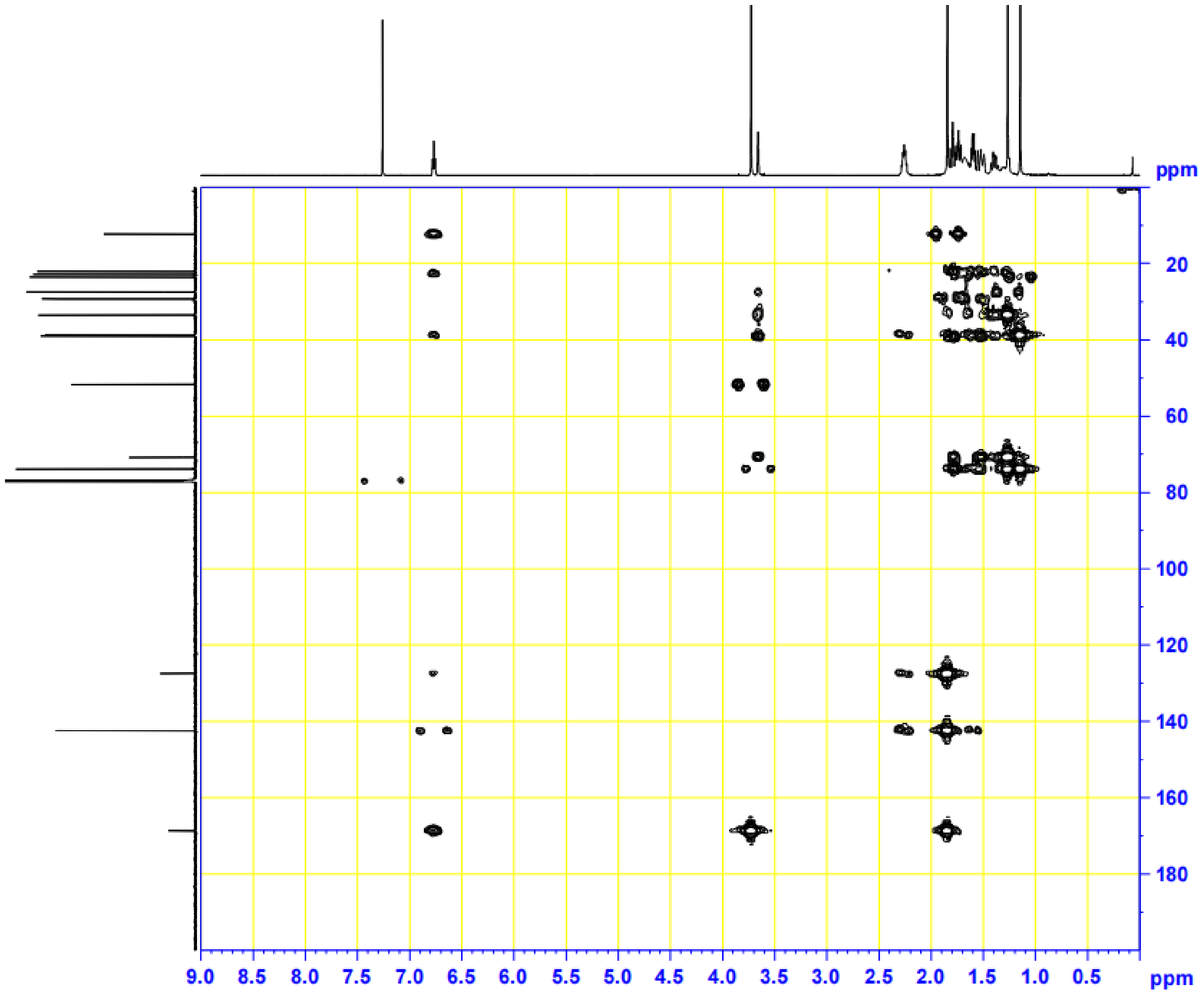

Fig. S56 HMBC Spectrum of **4** in chloroform-*d* (600 MHz).

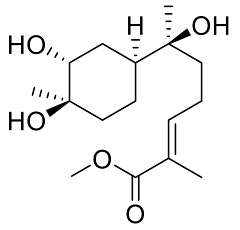

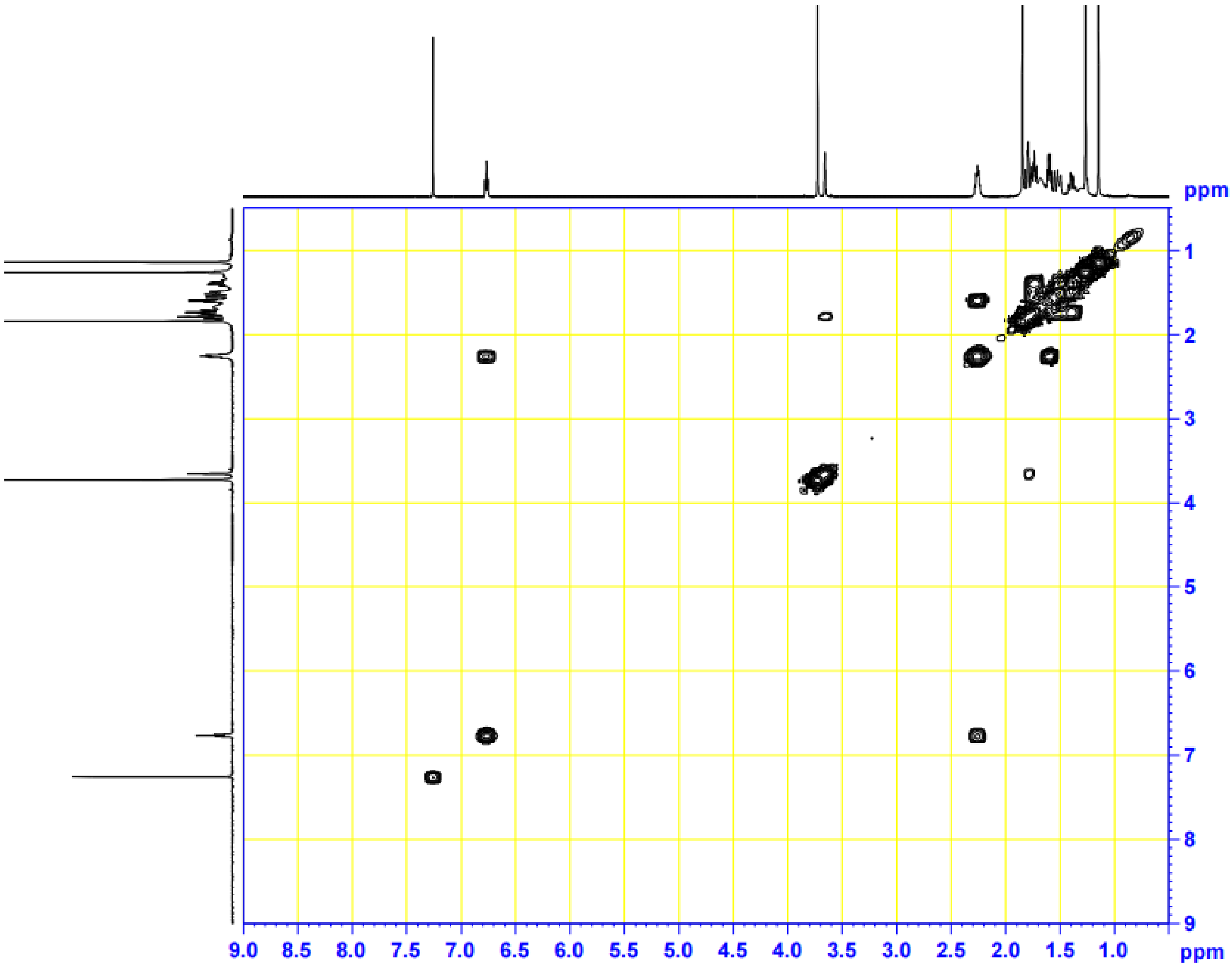

Fig. S57 ^{1}H–^{1}H COSY Spectrum of **4** in chloroform-*d* (600 MHz).

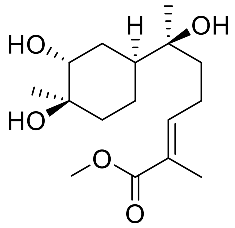

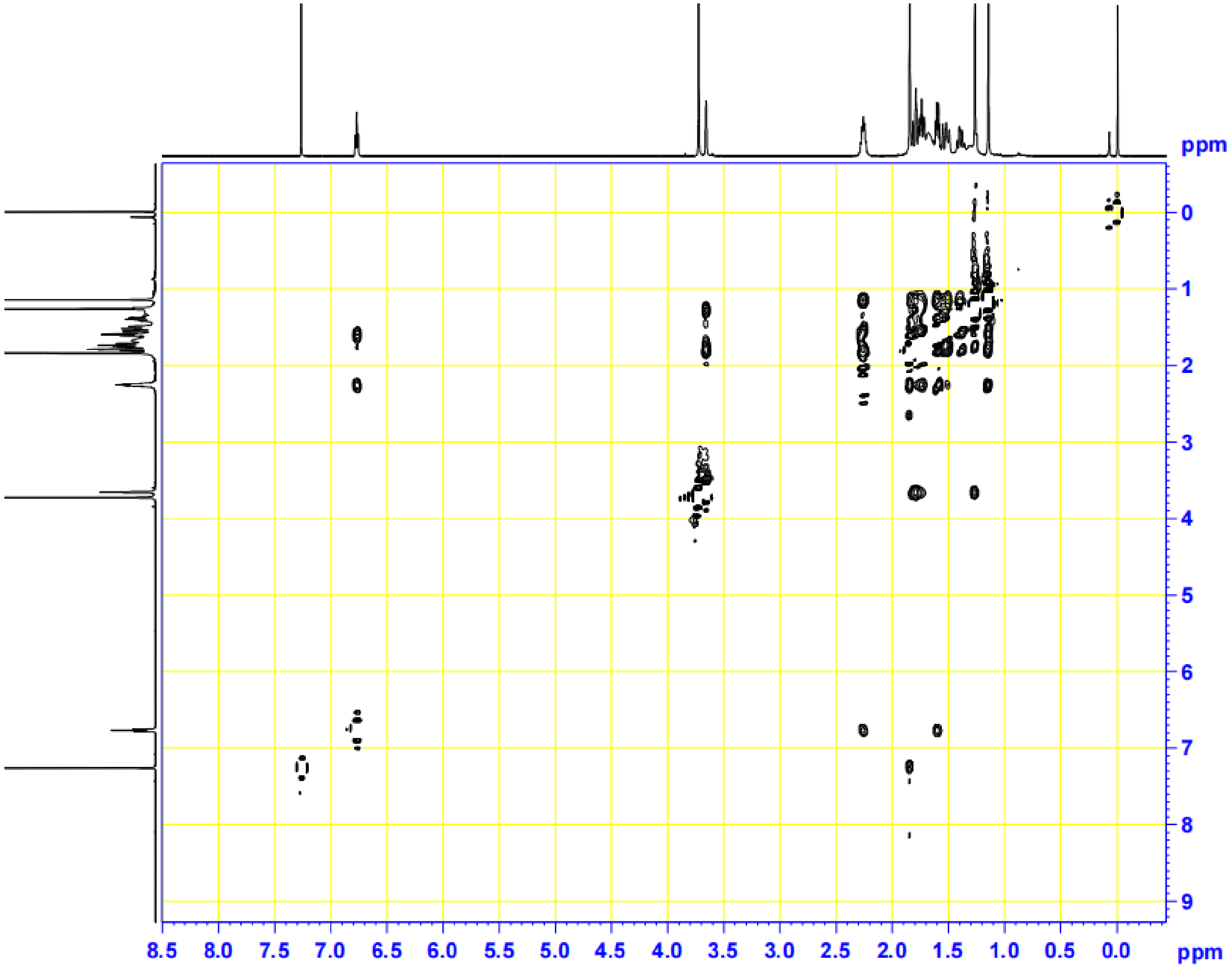

Fig. S58 ROESY Spectrum of **4** in chloroform-*d* (600 MHz).

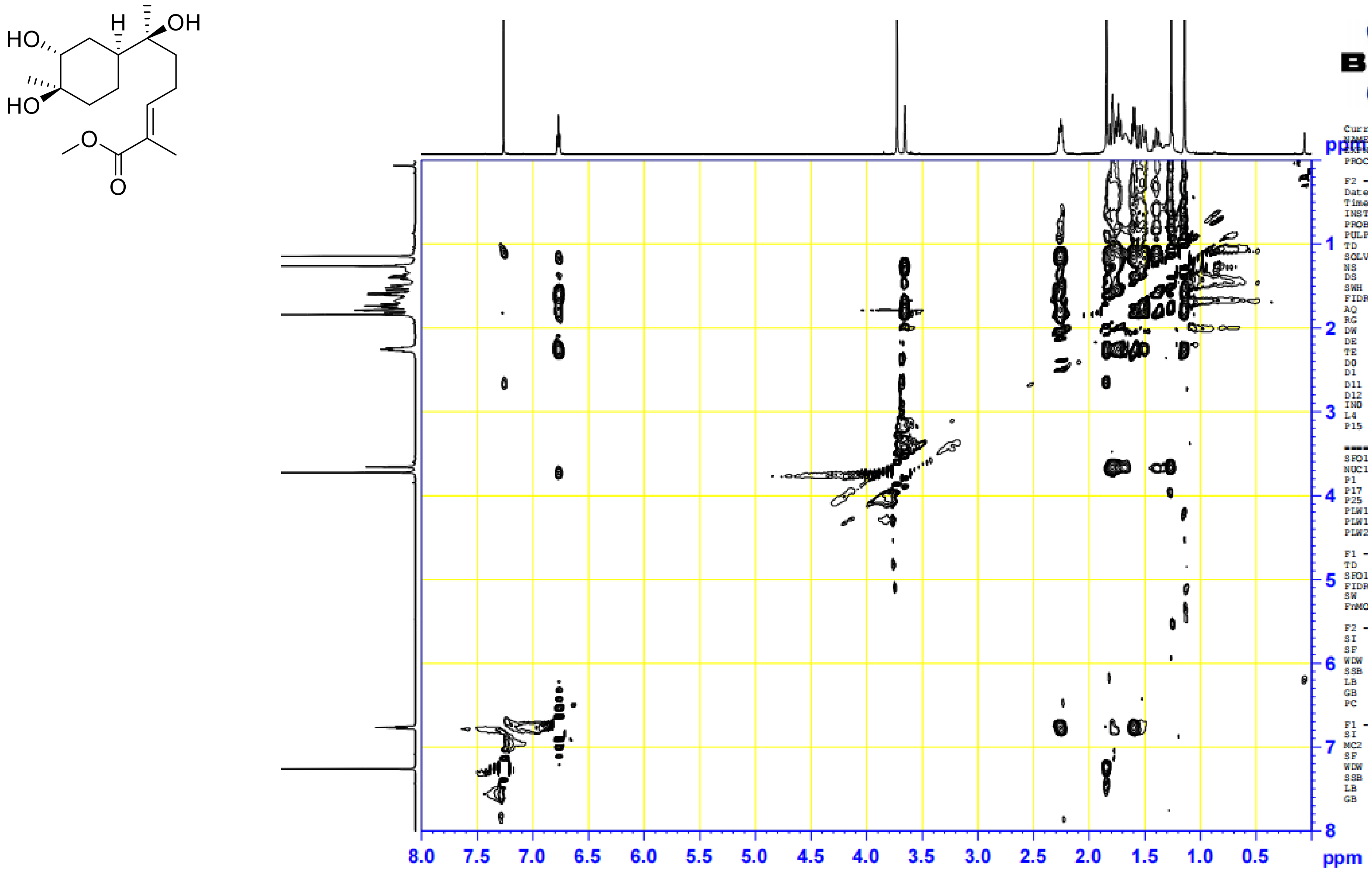

Fig. S59 ROESY Spectrum of **4** in chloroform-*d* (600 MHz) (expanded).

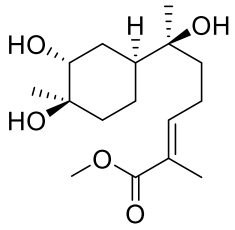

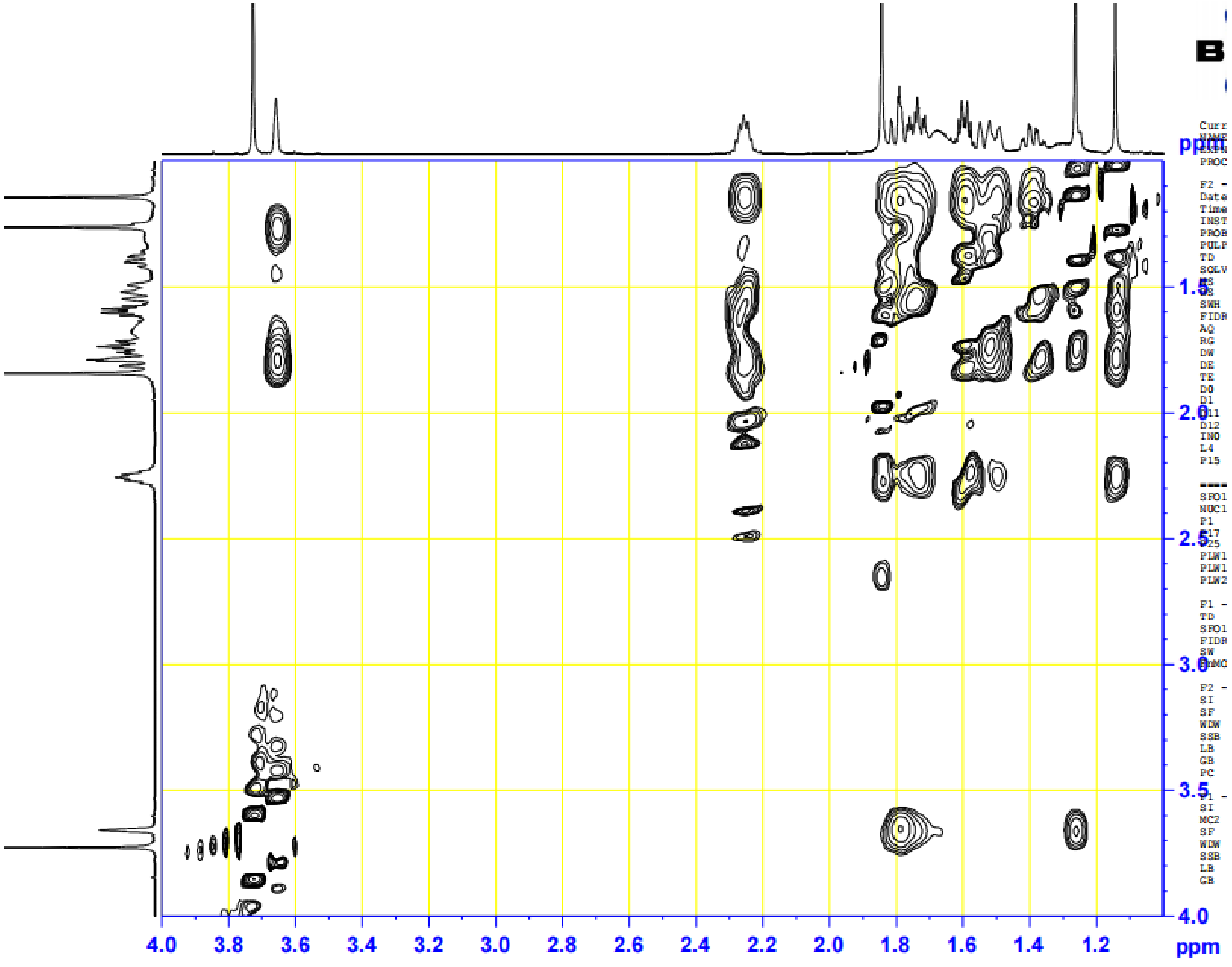

Fig. S60 ROESY Spectrum of **4** in chloroform-*d* (600 MHz) (expanded).

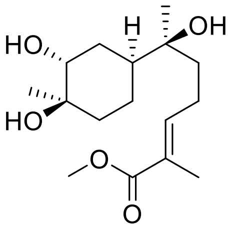

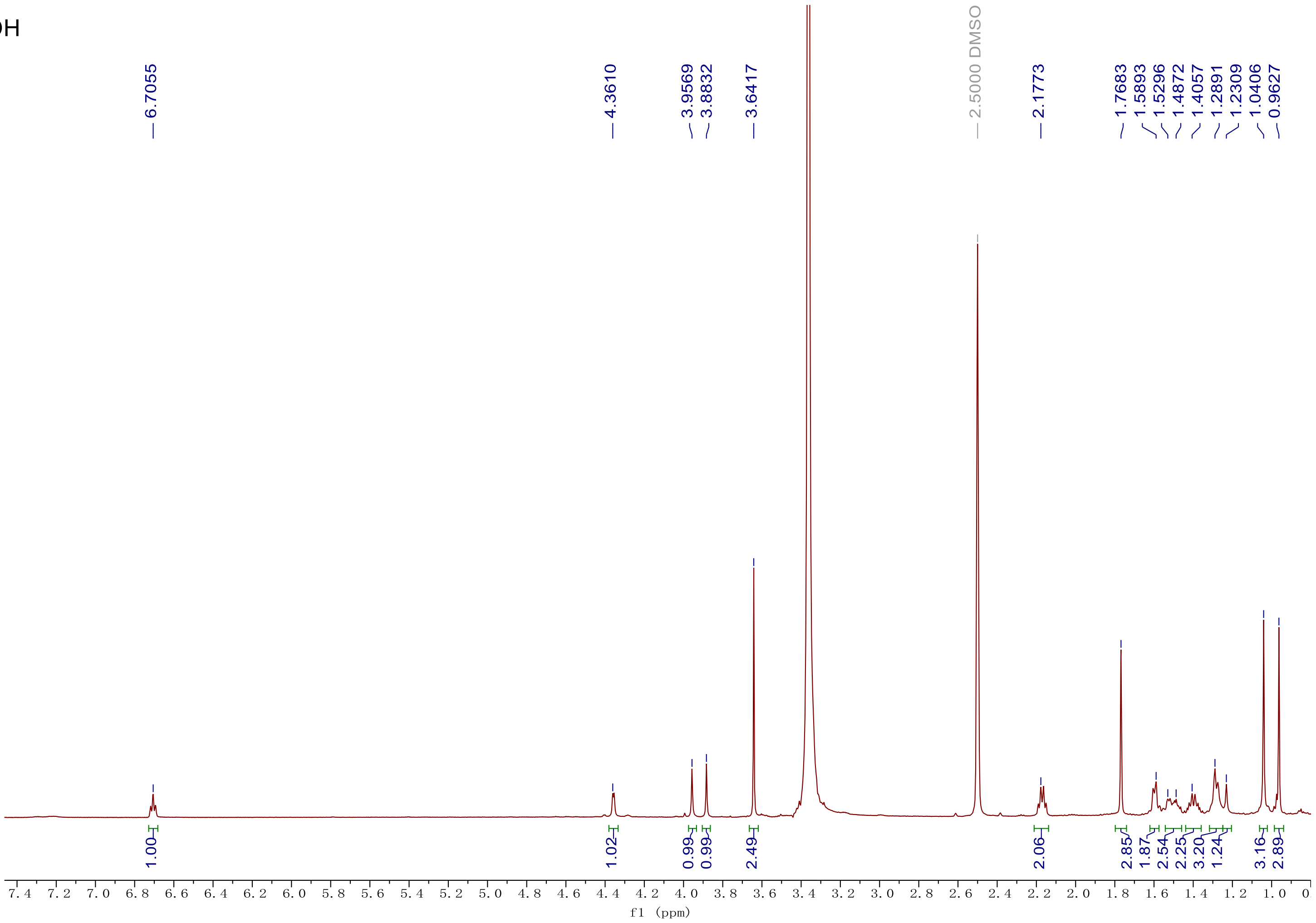

Fig. S61 ^{1}H NMR Spectrum of **4** in DMSO-d_6 (600 MHz).

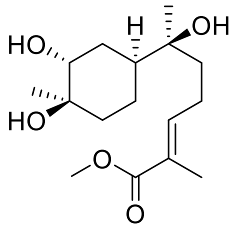

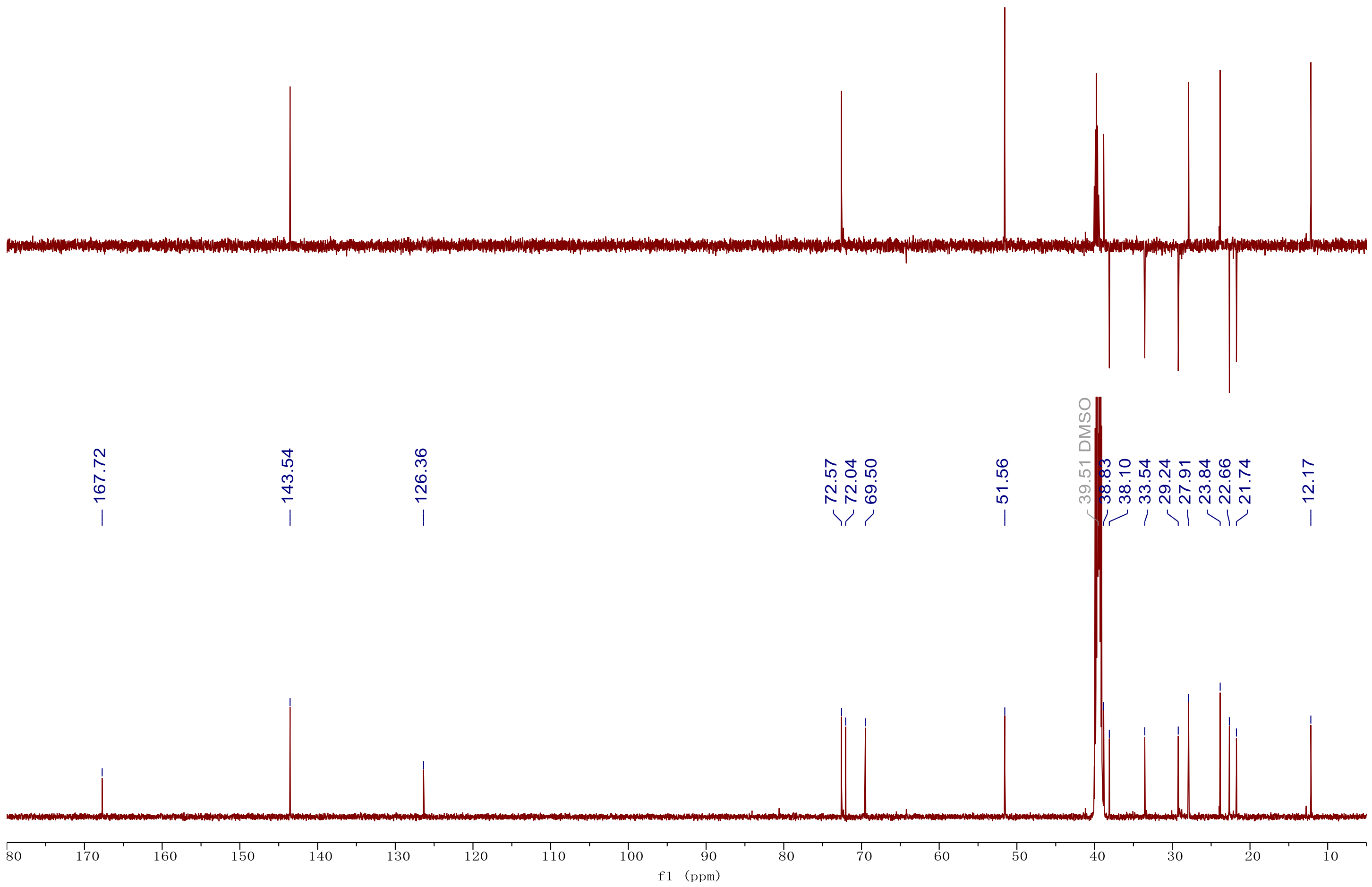

Fig. S62 ^{13}C NMR Spectrum of **4** in DMSO-d_6 (150 MHz).

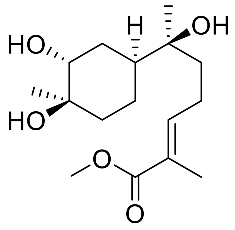

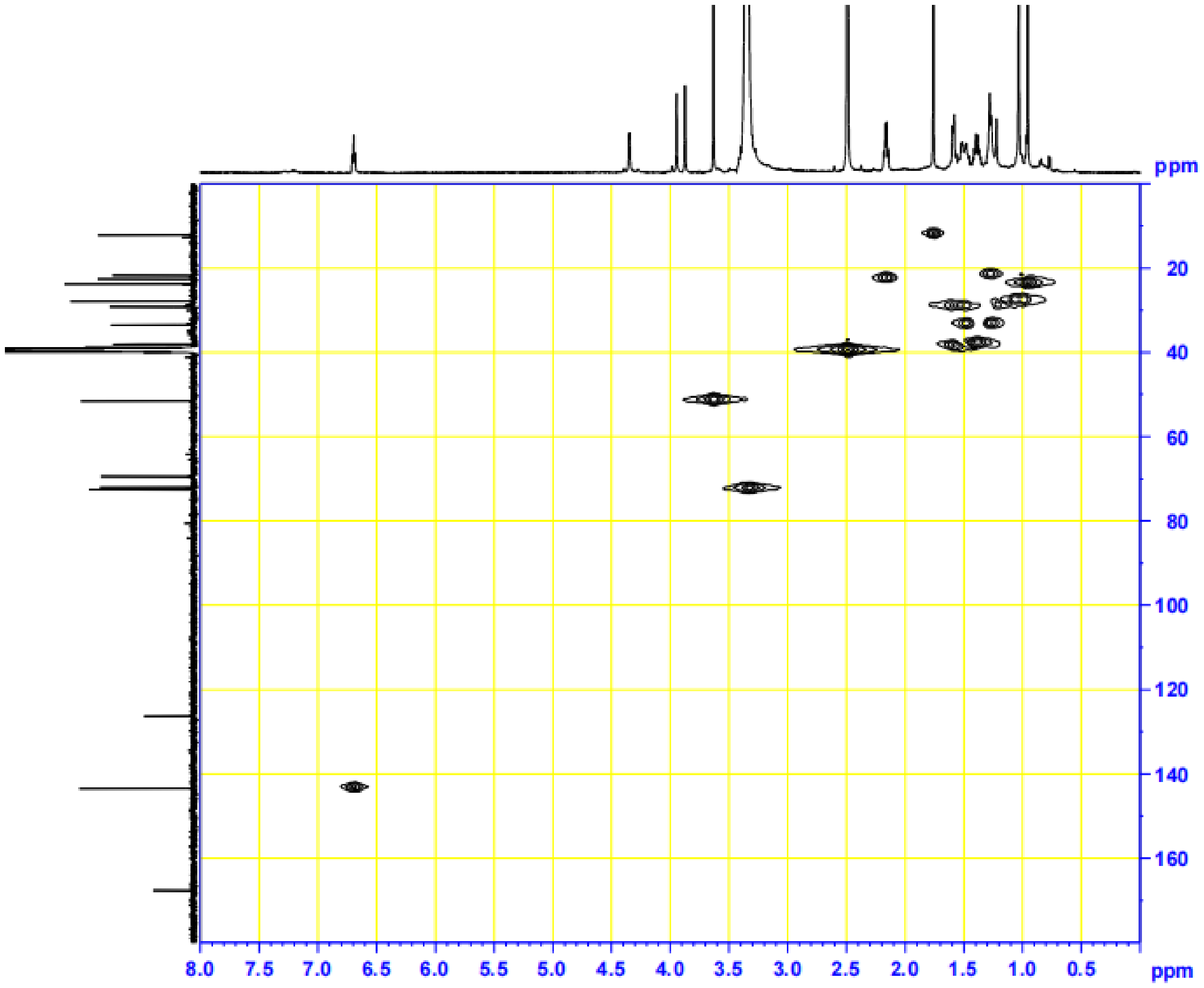

Fig. S63 HSQC Spectrum of **4** in DMSO-d_6 (600 MHz).

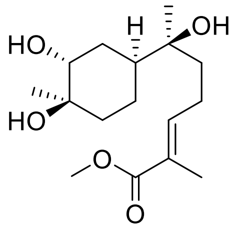

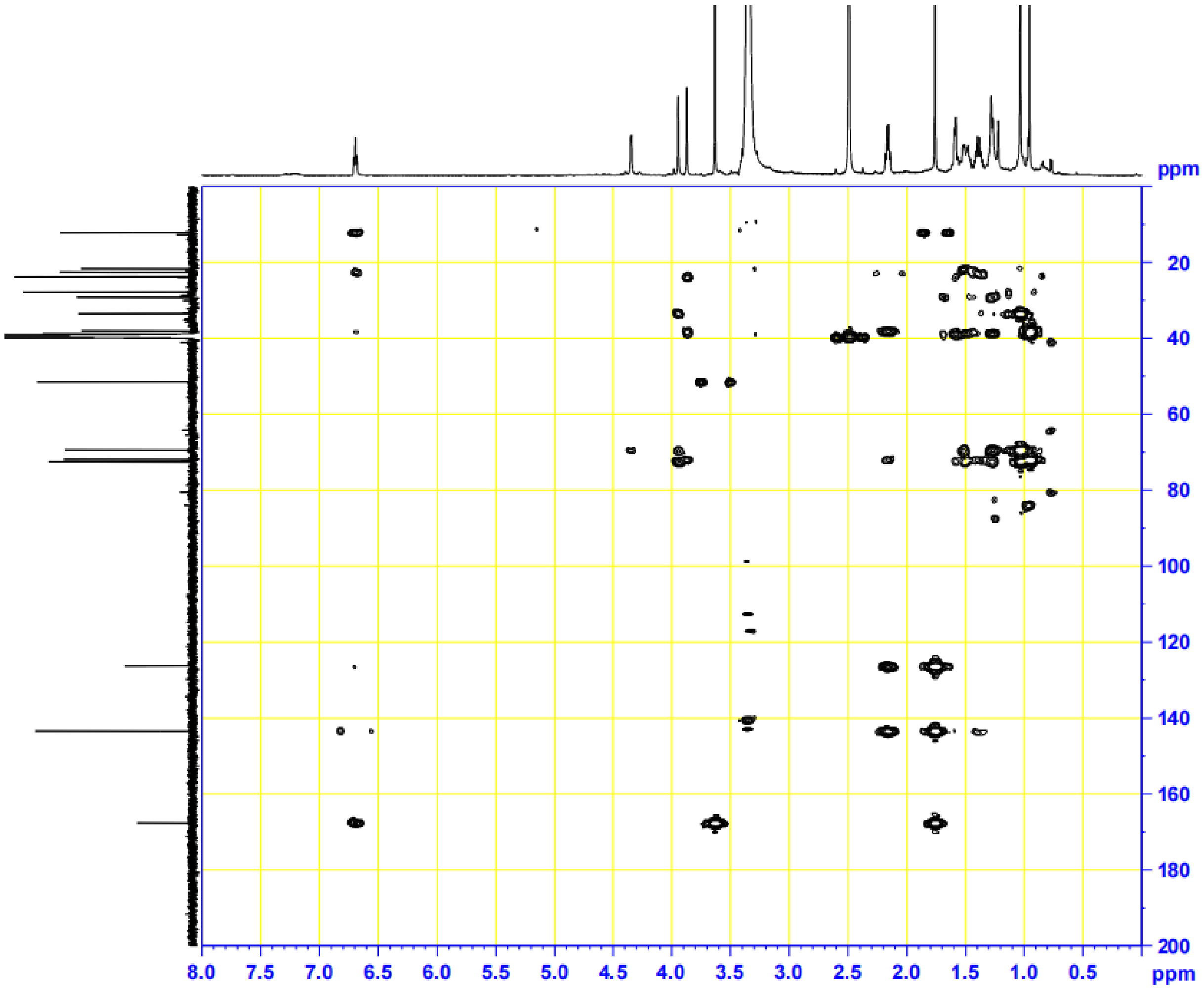

Fig. S64 HMBC Spectrum of **4** in DMSO-d_6 (600 MHz)

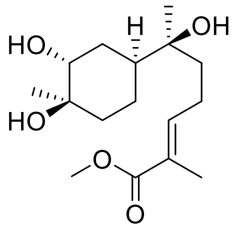

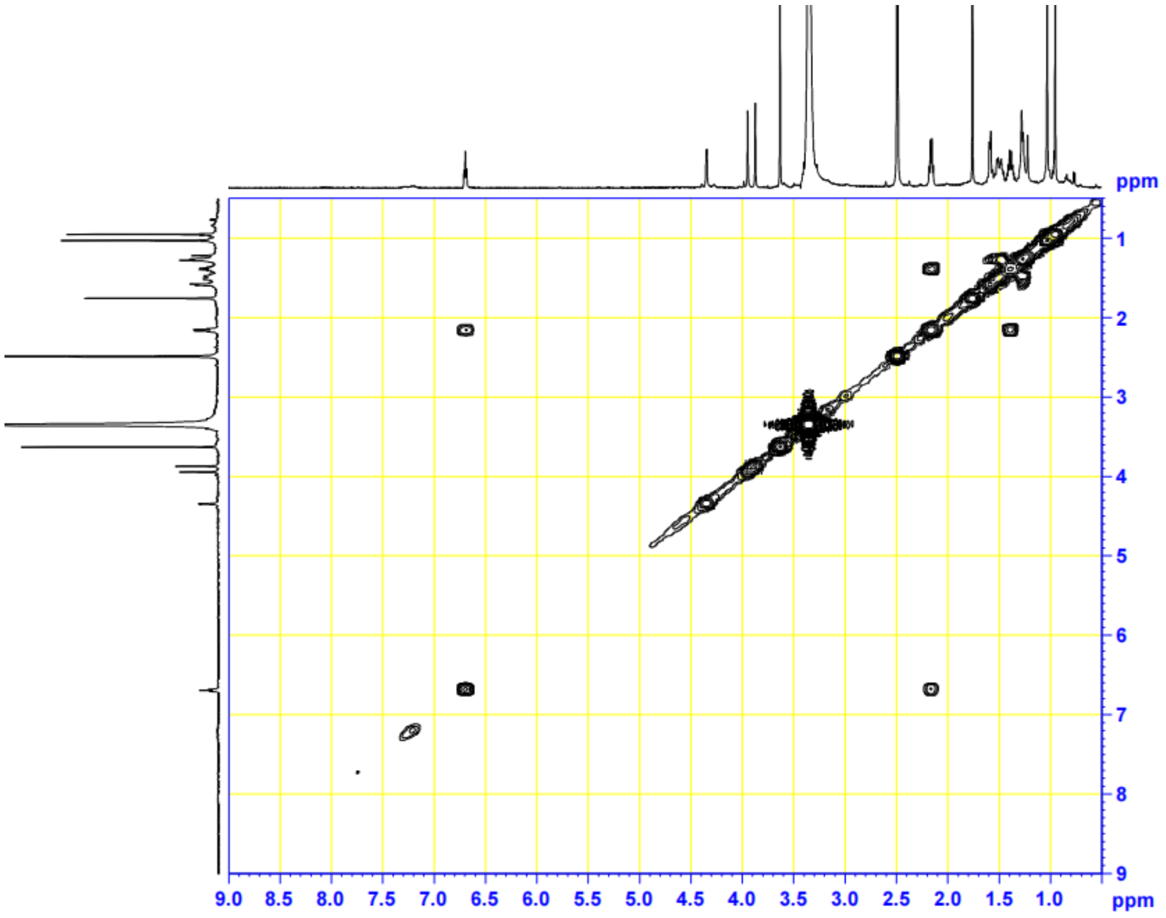

Fig. S65 ^{1}H–^{1}H COSY Spectrum of **4** in DMSO-d_6 (600 MHz).

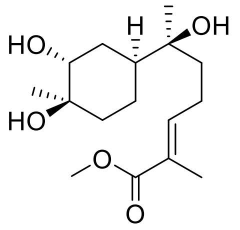

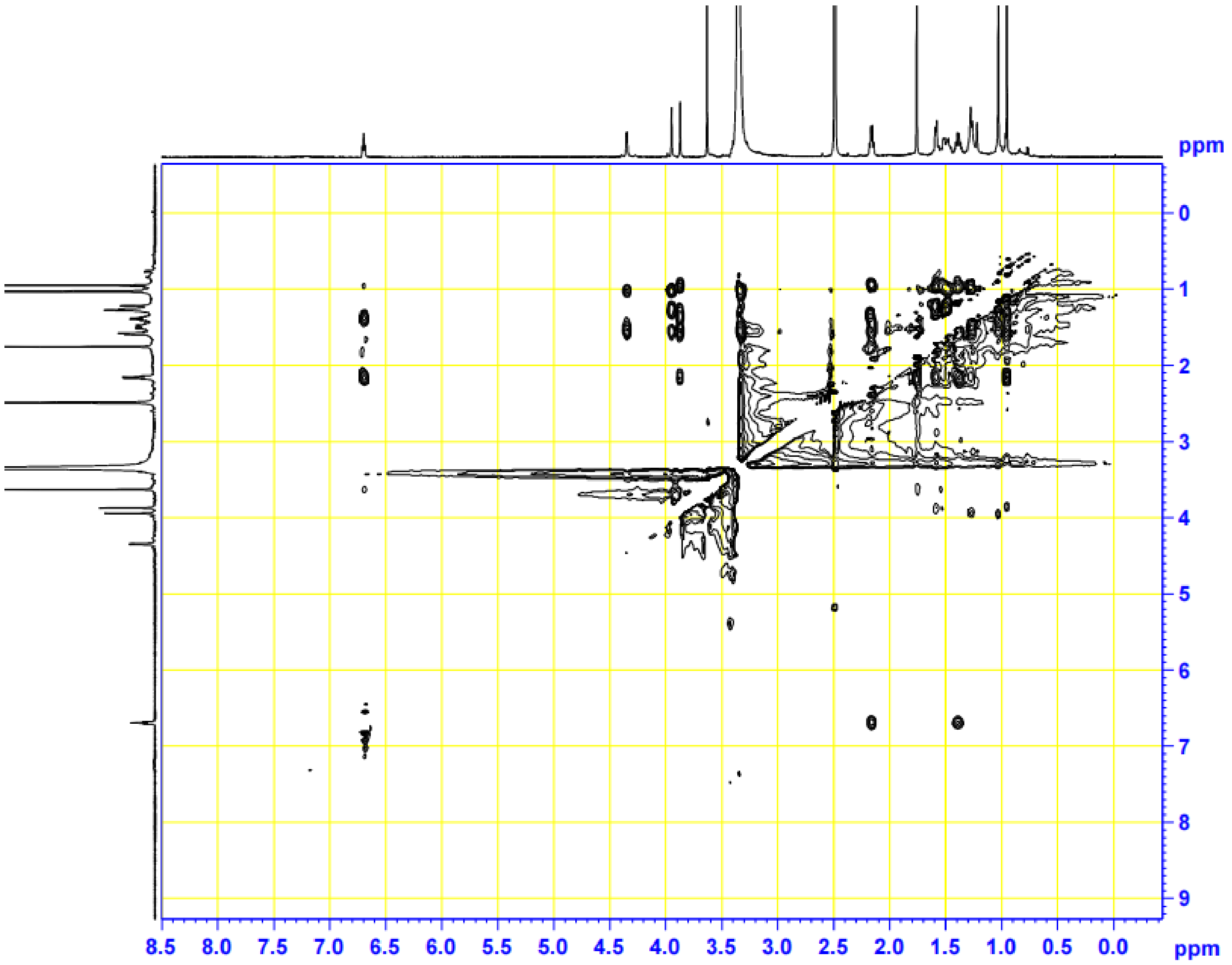

Fig. S66 ROESY Spectrum of **4** in DMSO-d_6 (600 MHz).

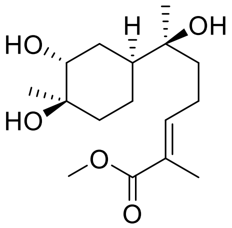

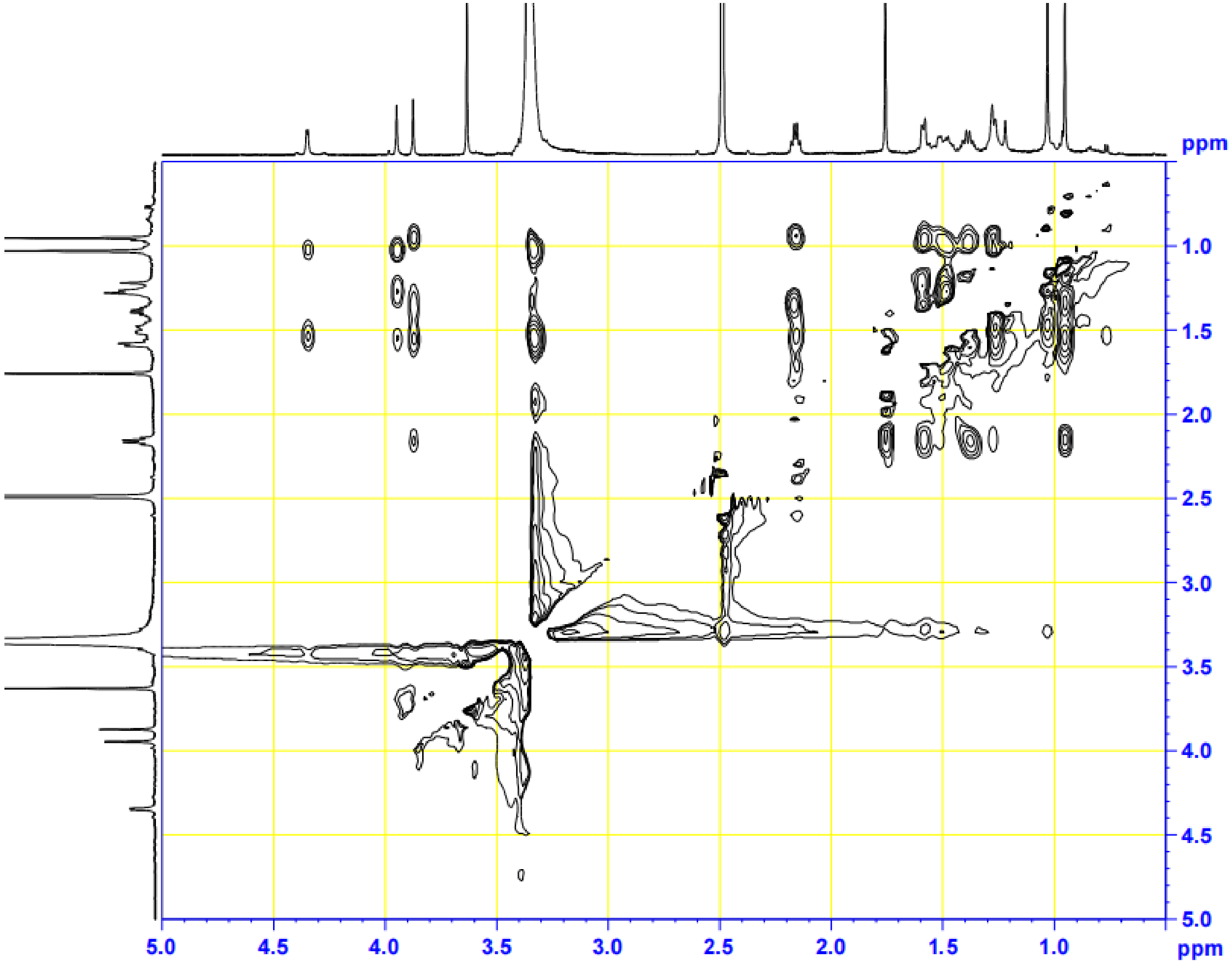

Fig. S67 ROESY Spectrum of **4** in DMSO-d_6 (600 MHz) (expanded).

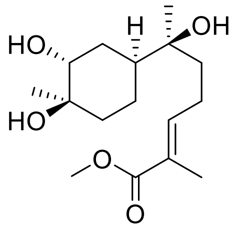

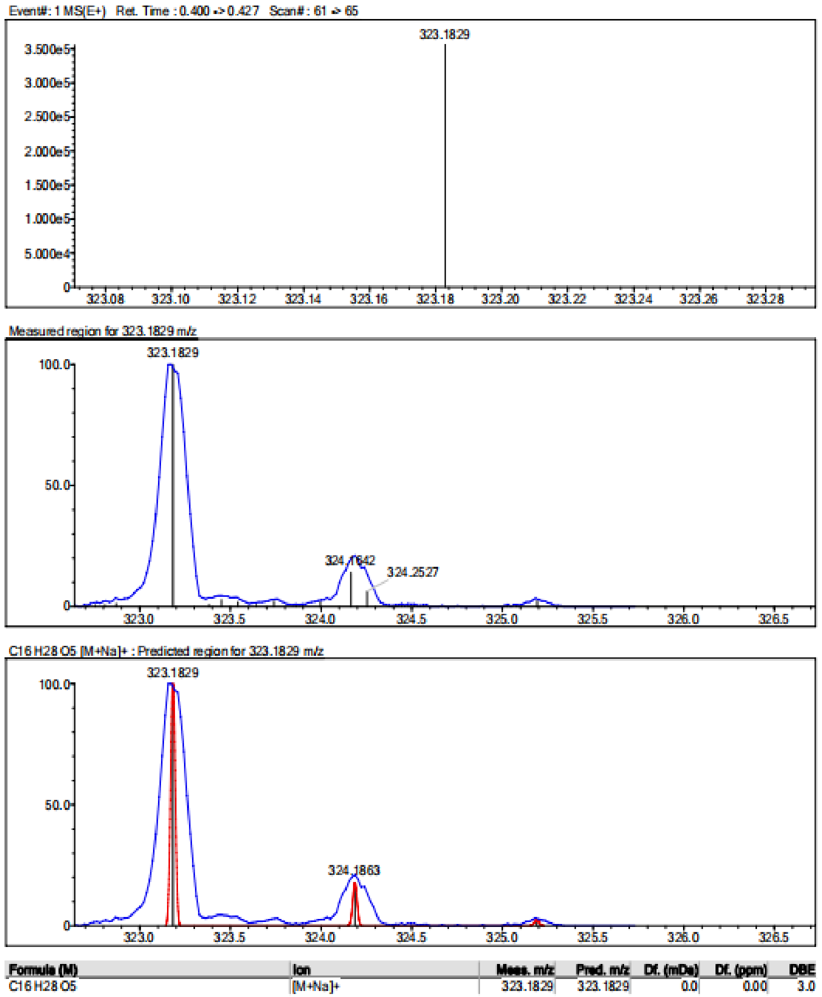

Fig. S68 HRESIMS Spectrum of **4**.

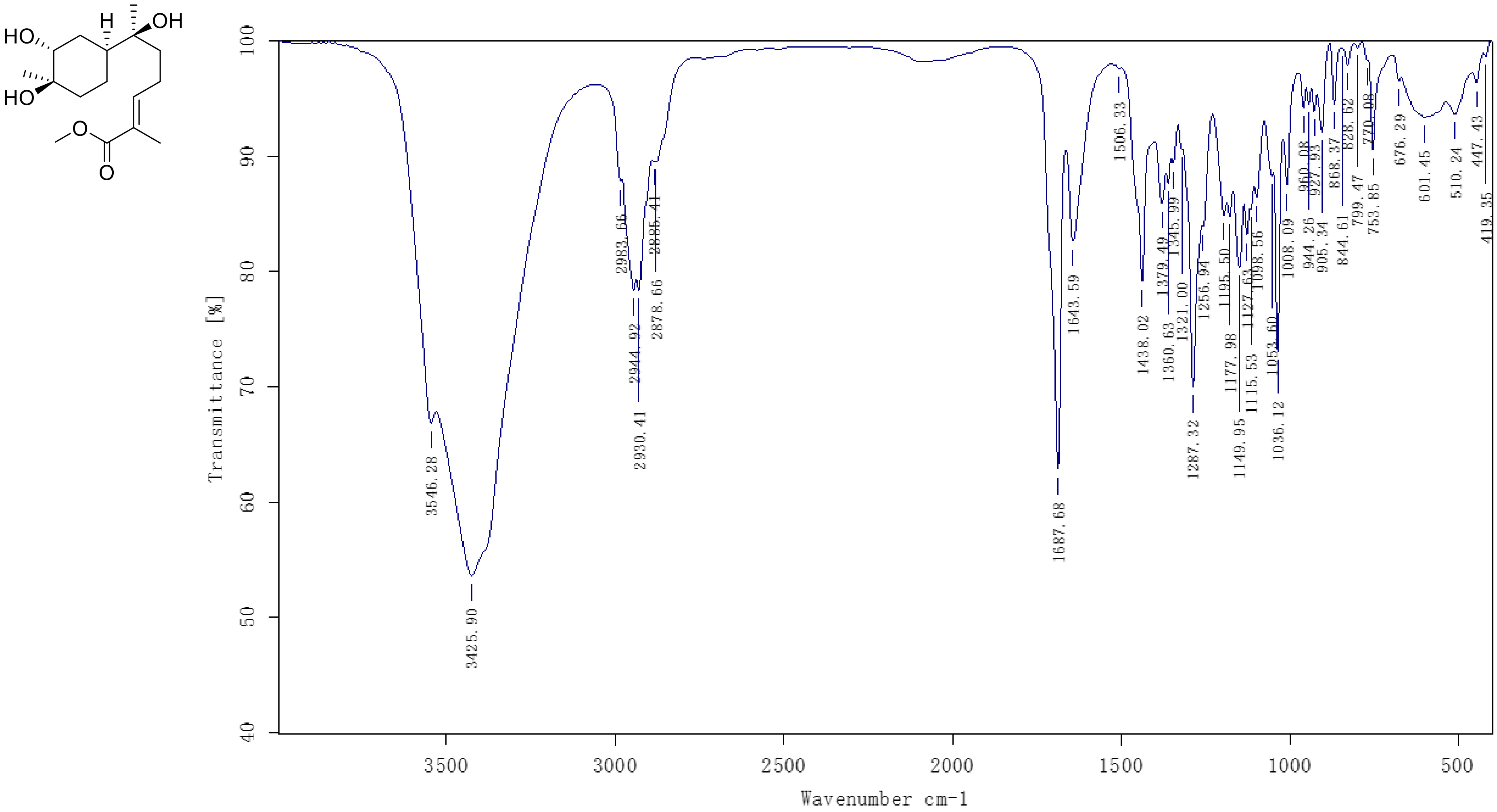

Fig. S69 IR Spectrum of **4**.

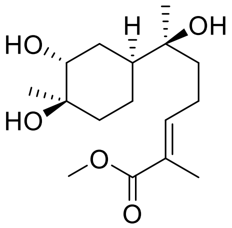

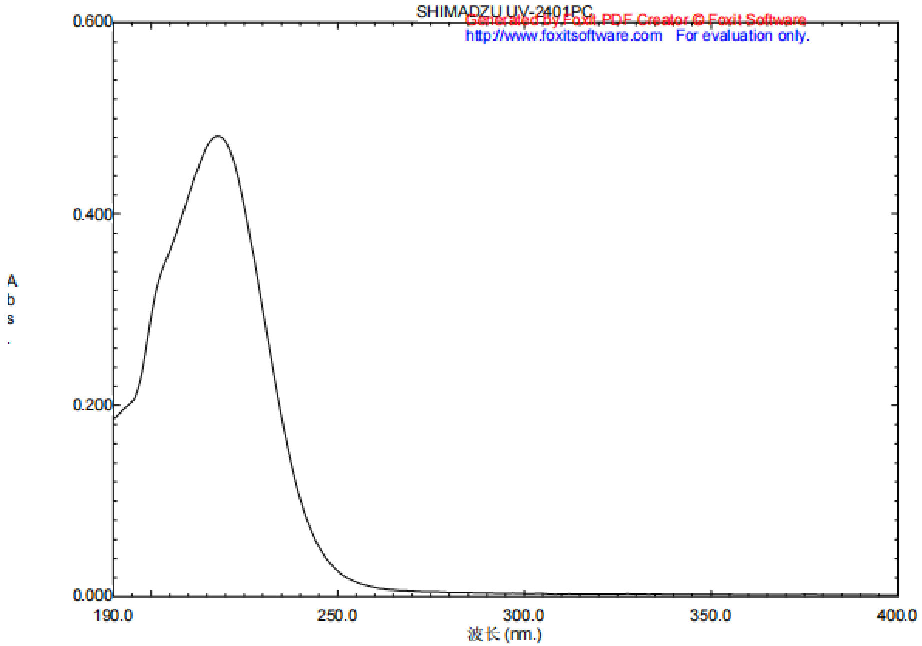

Fig. S70 UV Spectrum of **4**.

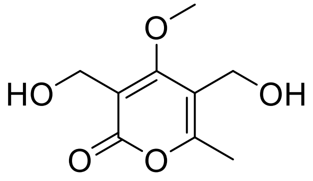

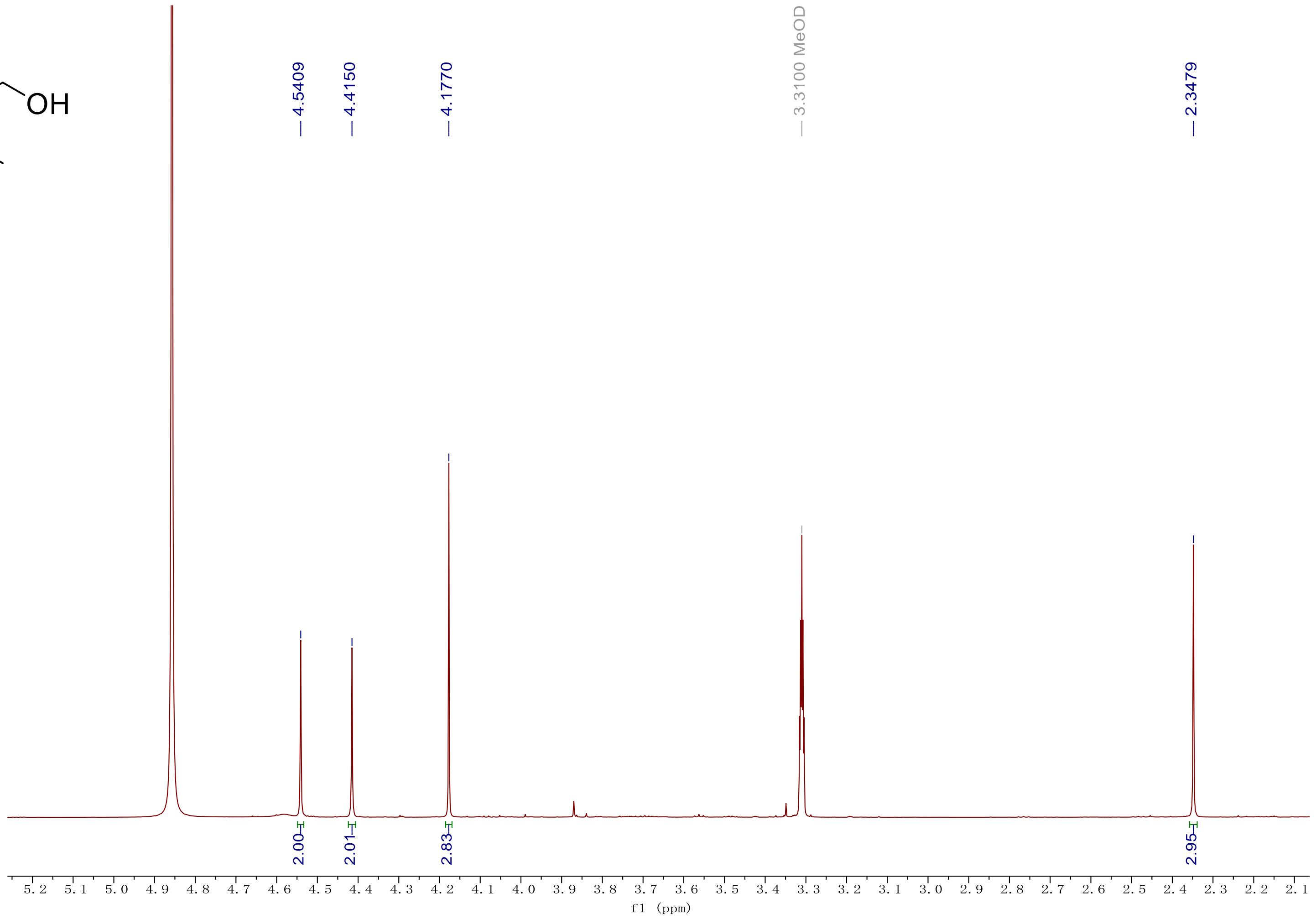

Fig. S71 ^{1}H NMR Spectrum of **5** in methanol-d_4 (600 MHz).

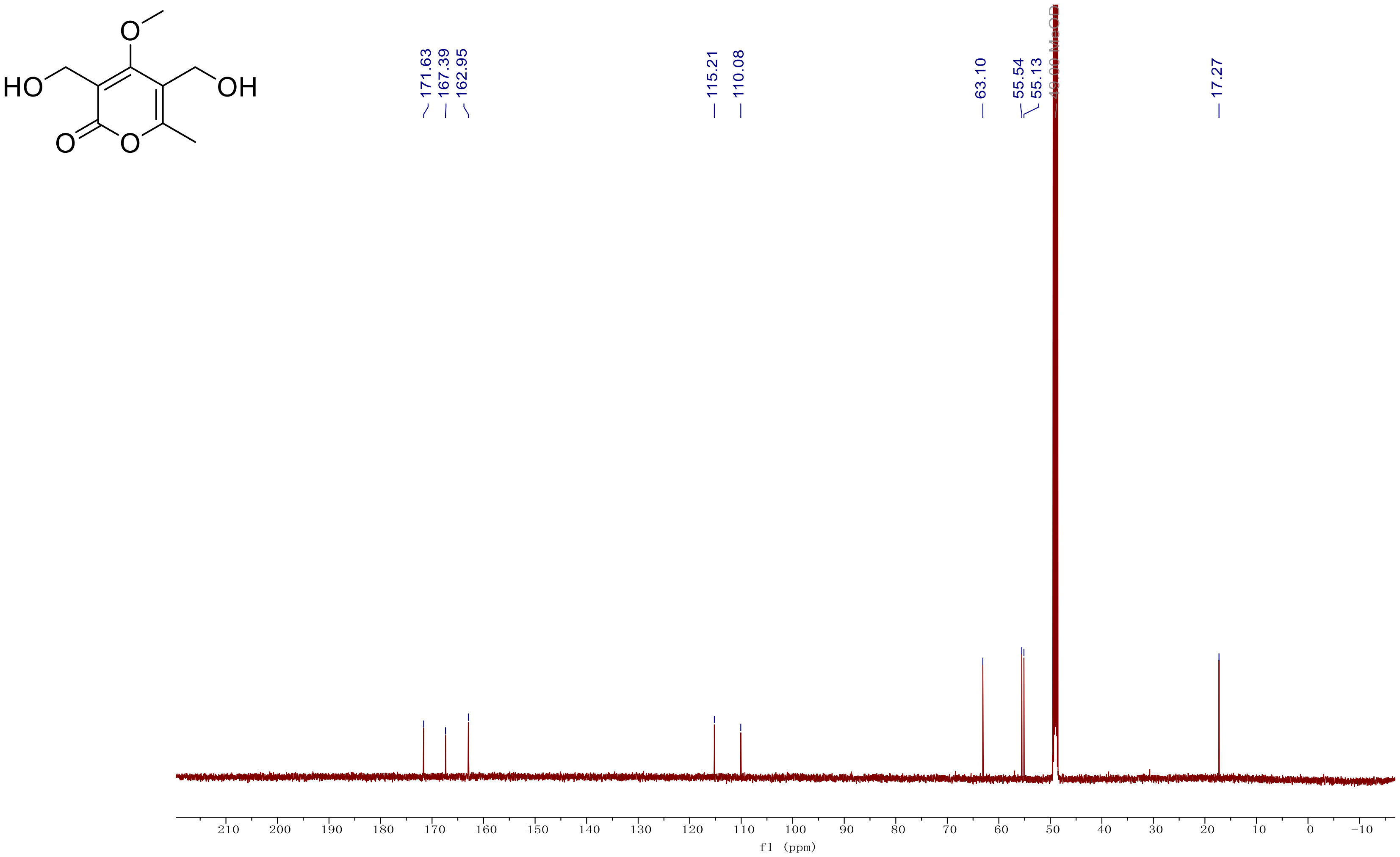

Fig. S72 ^{13}C NMR Spectrum of **5** in methanol-d_4 (150 MHz).

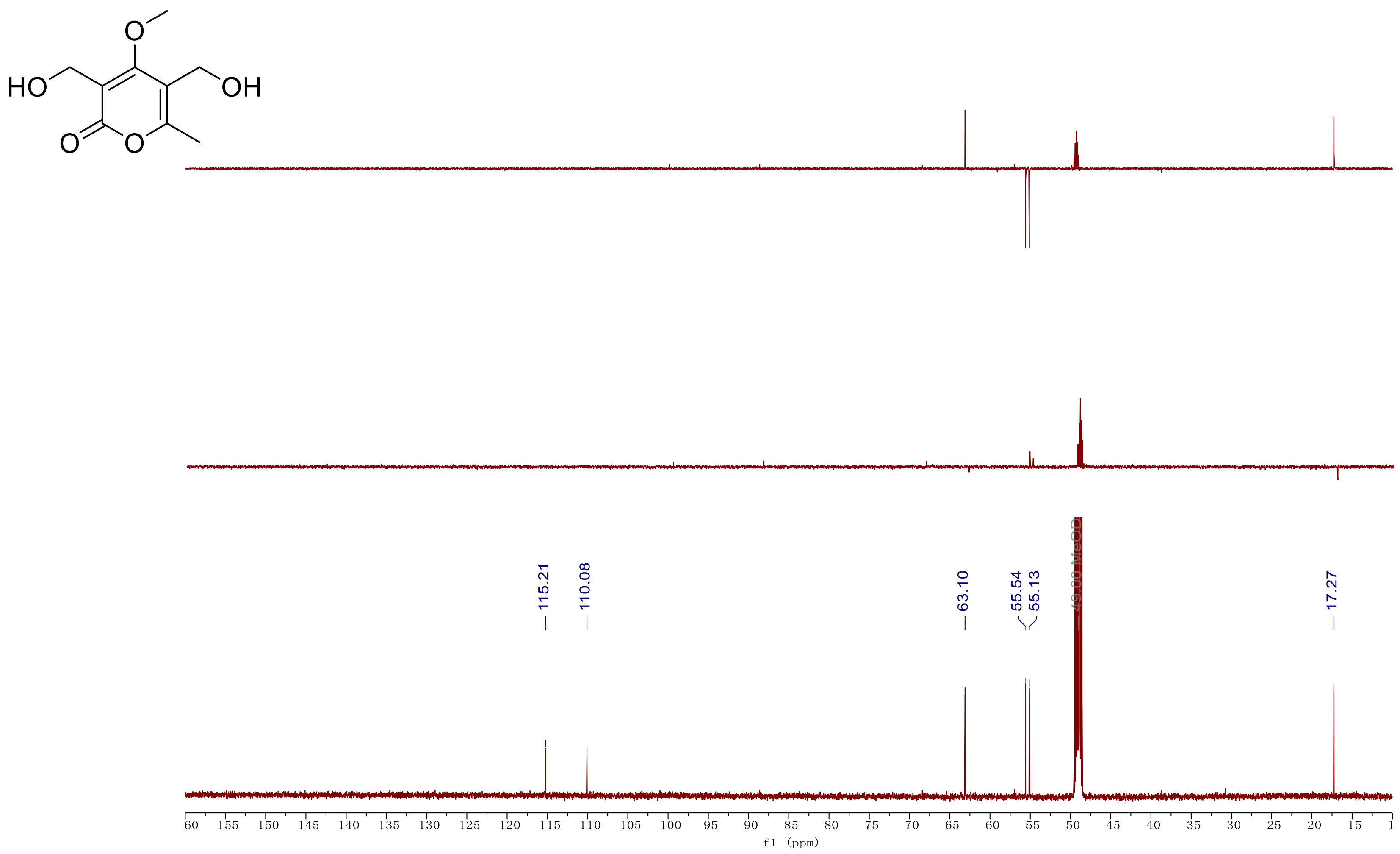

Fig. S73 The DEPT Spectrum of **5** in methanol-d_4 (150 MHz).

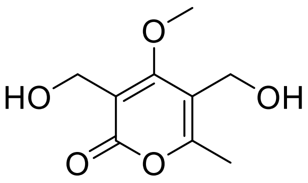

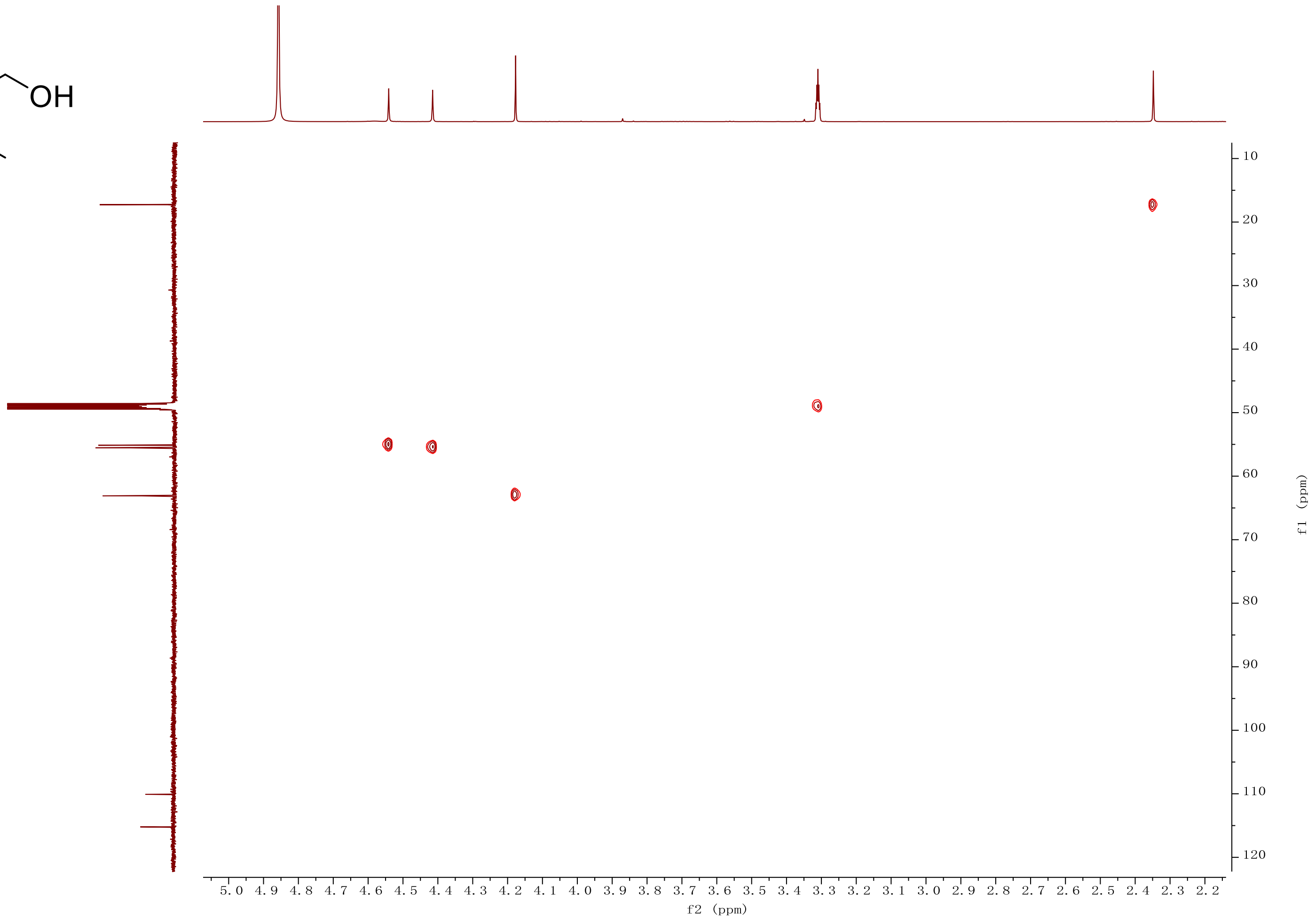

Fig. S74 HSQC Spectrum of **5** in methanol-d_4 (600 MHz).

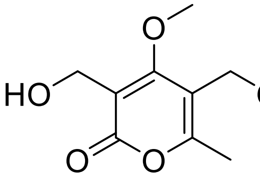

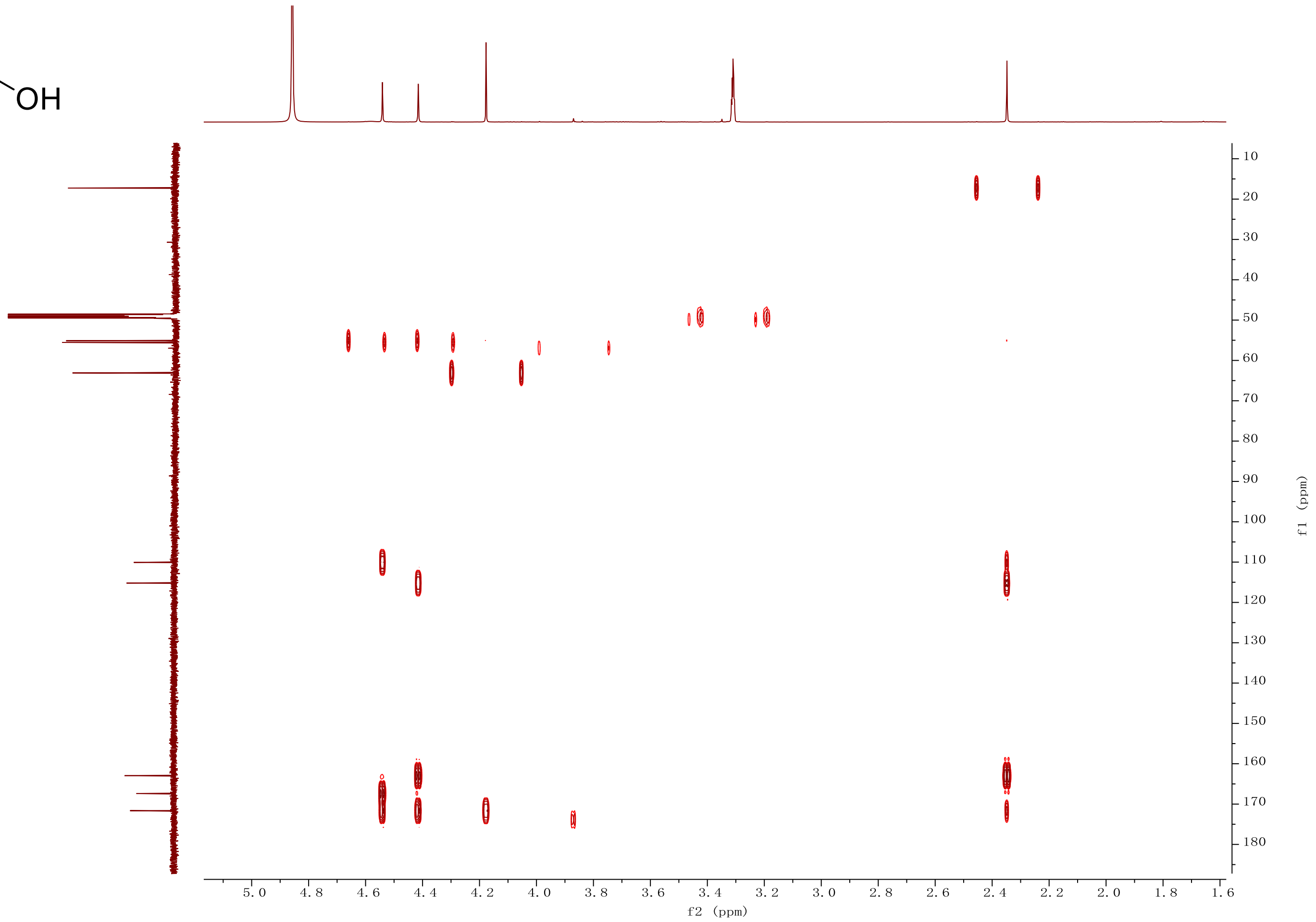

Fig. S75 HMBC Spectrum of **5** in methanol-d_4 (600 MHz).

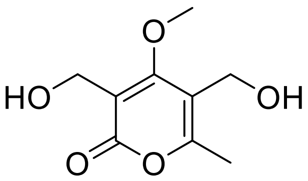

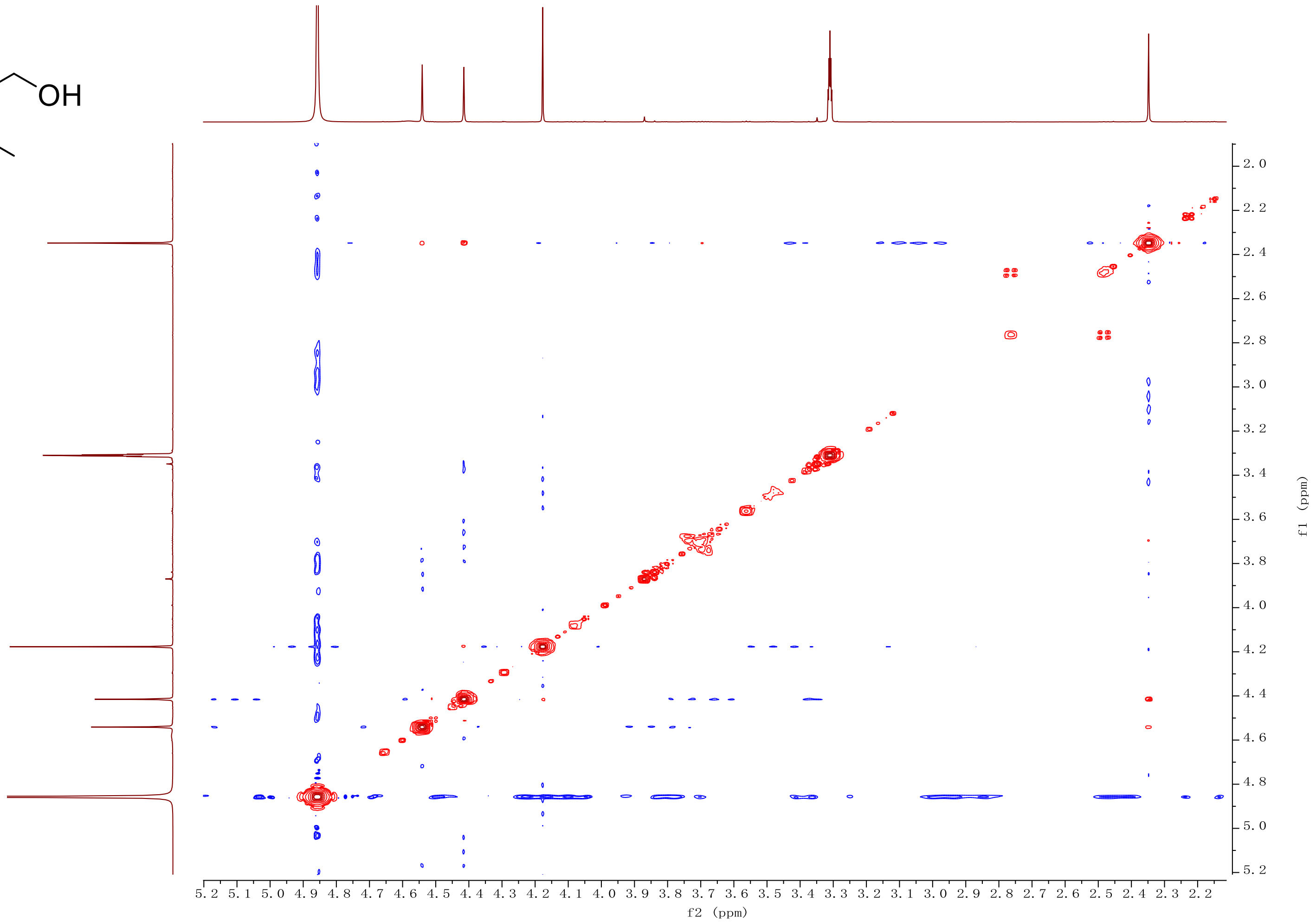

Fig. S76 ¹H–¹H COSY Spectrum of **5** in methanol-d_4 (600 MHz).

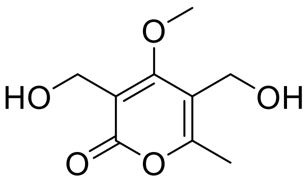

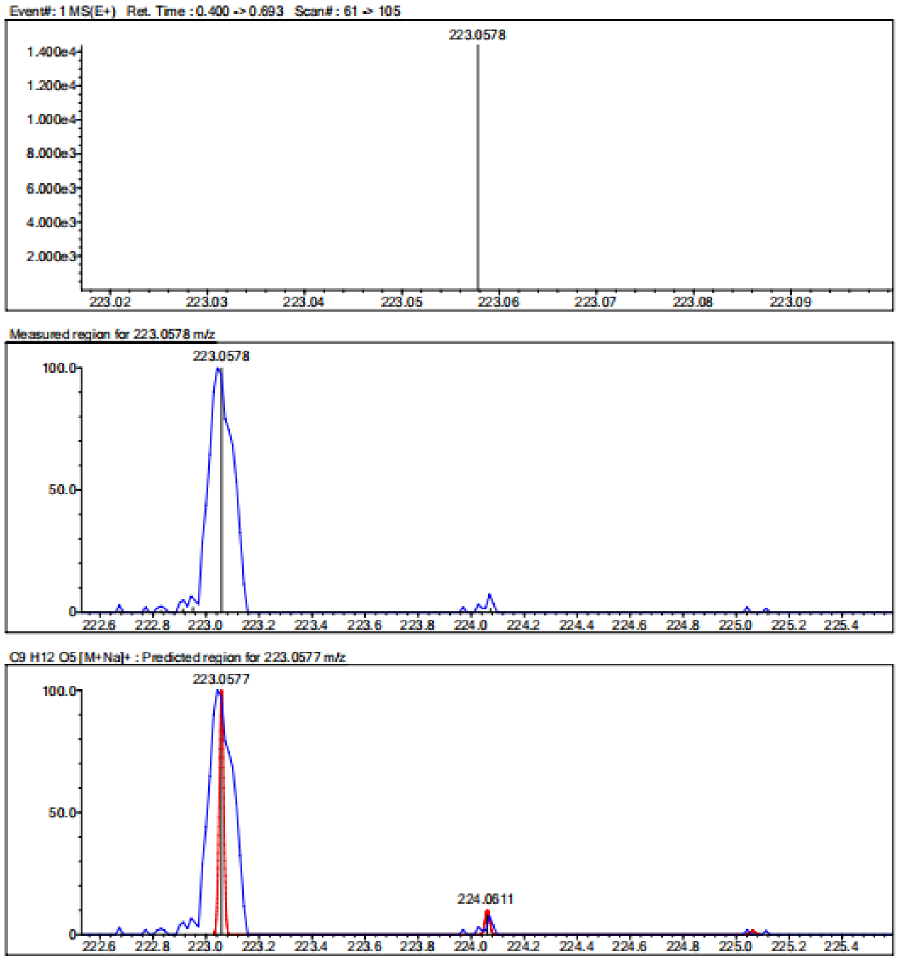

Formula (M)	Ion	Meas. m/z	Pred. m/z	Df. (mDa)	Df. (ppm)	DBE
C9 H12 O5	[M+Na]+	223.0578	223.0577	0.1	0.45	4.0

Fig. S77 HRESIMS Spectrum of **5**.

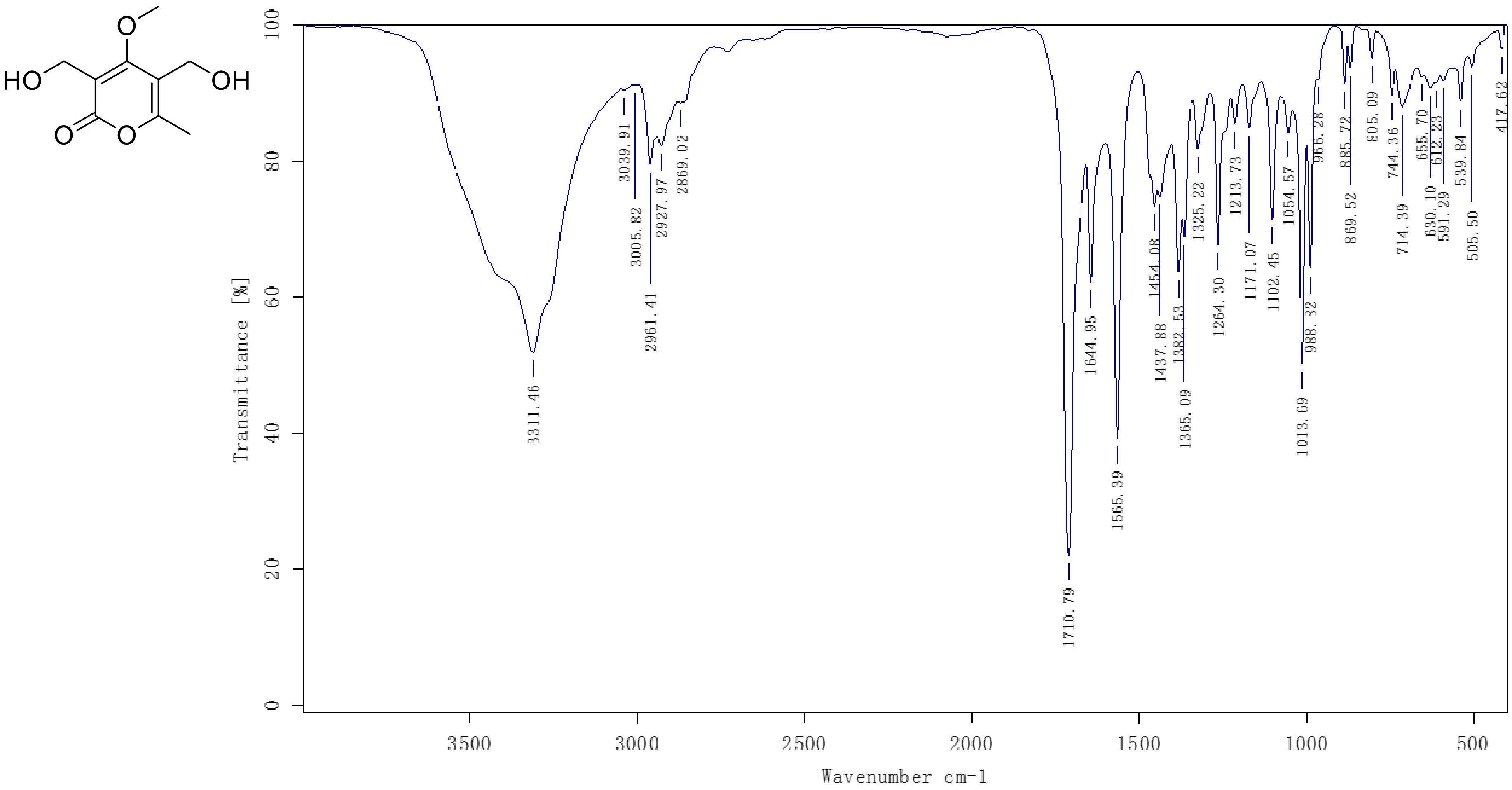

Fig. S78 IR Spectrum of **5**.

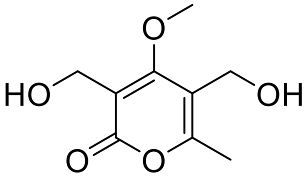

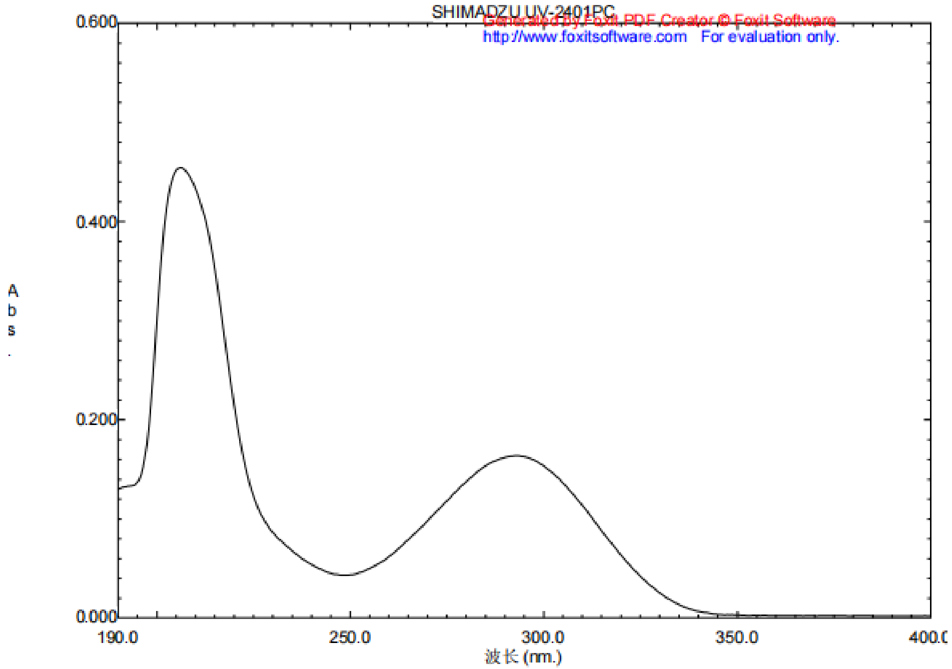

Fig. S79 UV Spectrum of **5**.

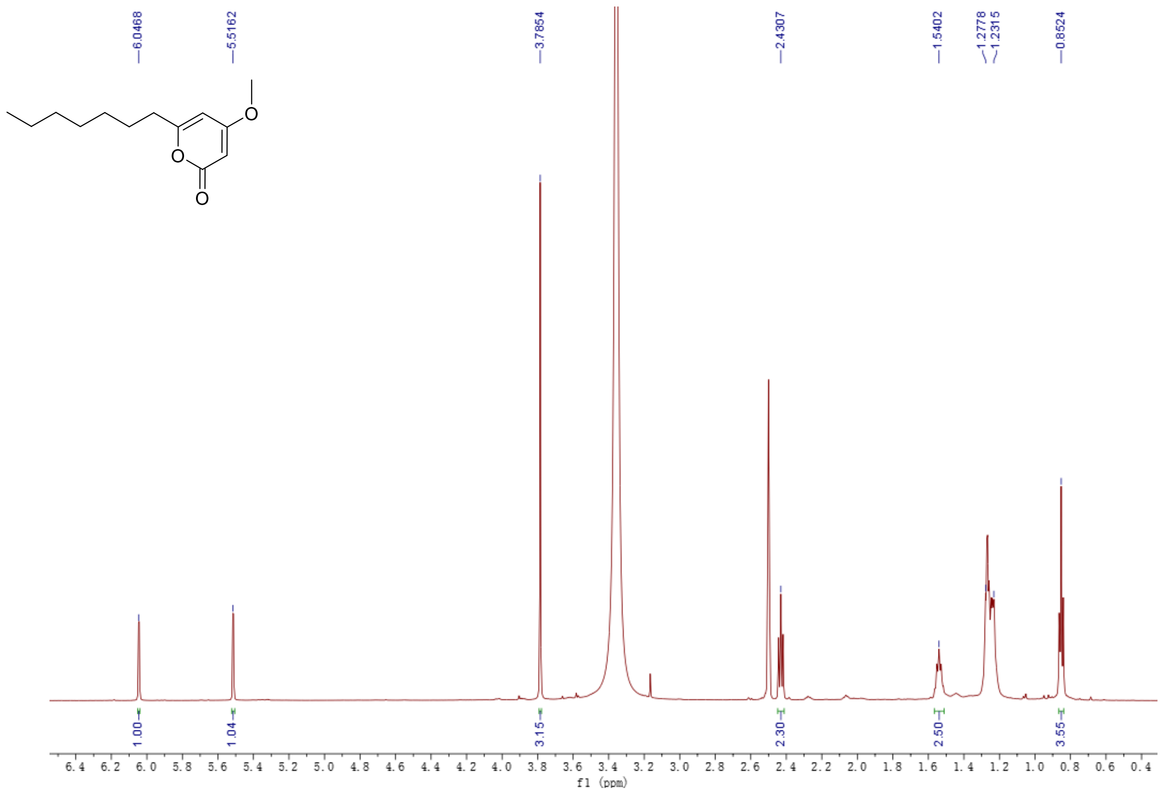

Fig. S80 ^{1}H NMR spectrum of **6** in DMSO-d_6 (600 MHz).

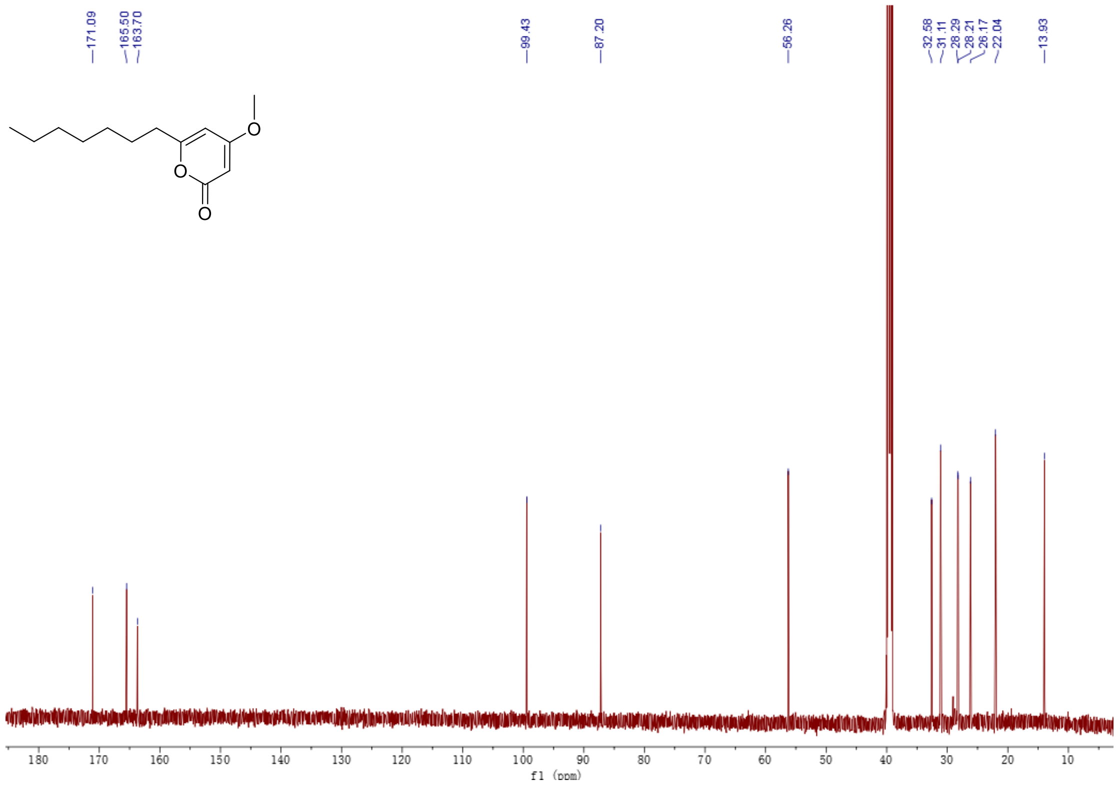

Fig. S81 ^{13}C NMR spectrum of **6** in DMSO-d_6 (150 MHz).

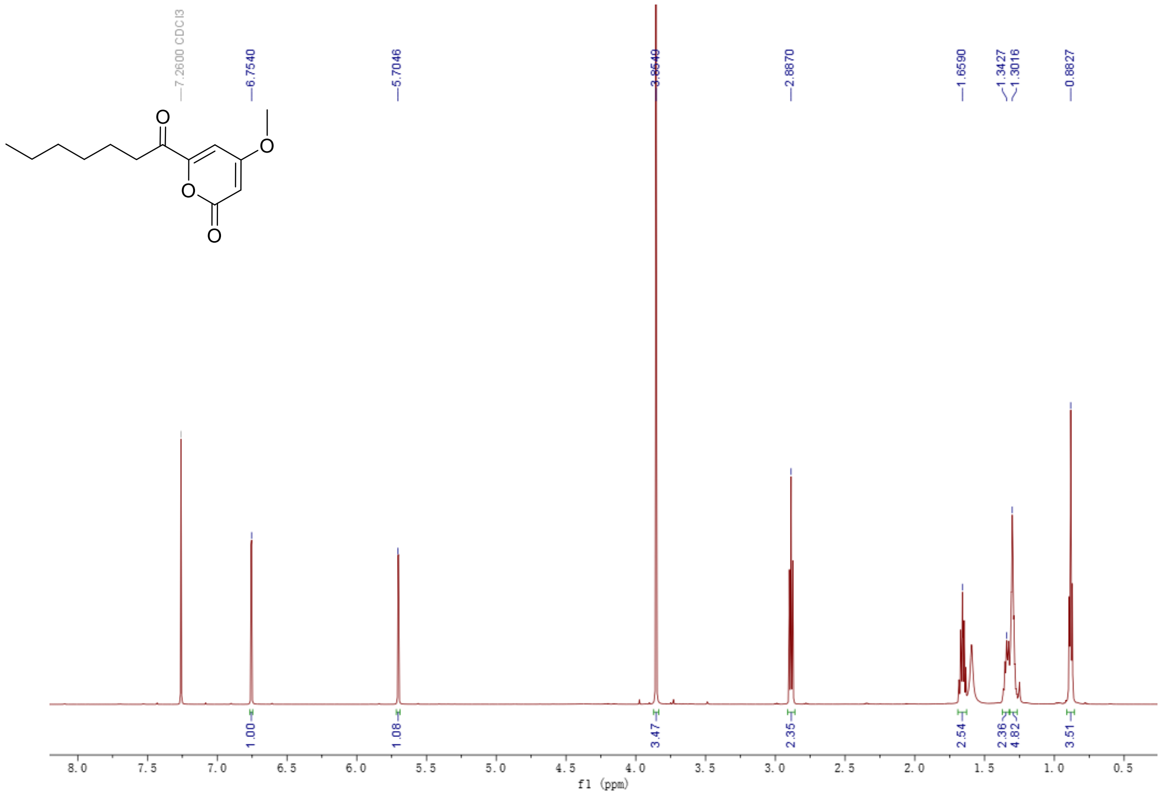

Fig. S82 ^{1}H NMR spectrum of **7** in chloroform-*d* (600 MHz).

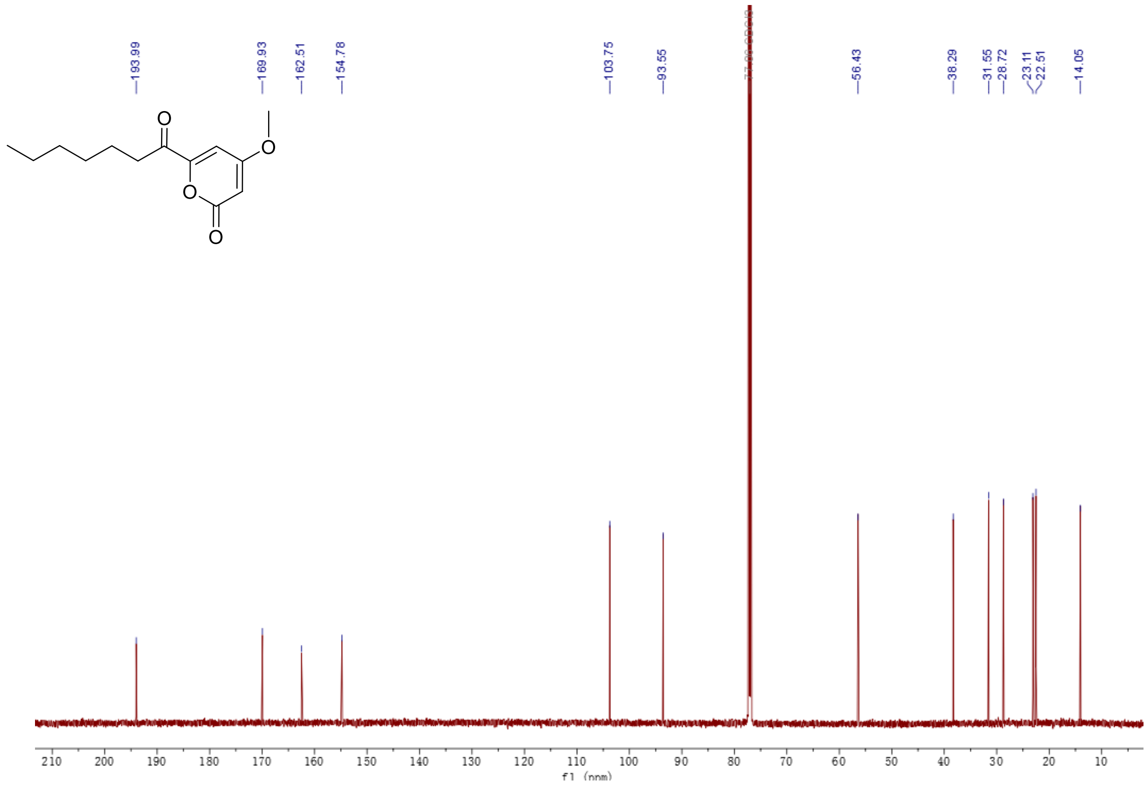

Fig. S83 ^{13}C NMR spectrum of **7** in chloroform-*d* (150 MHz).

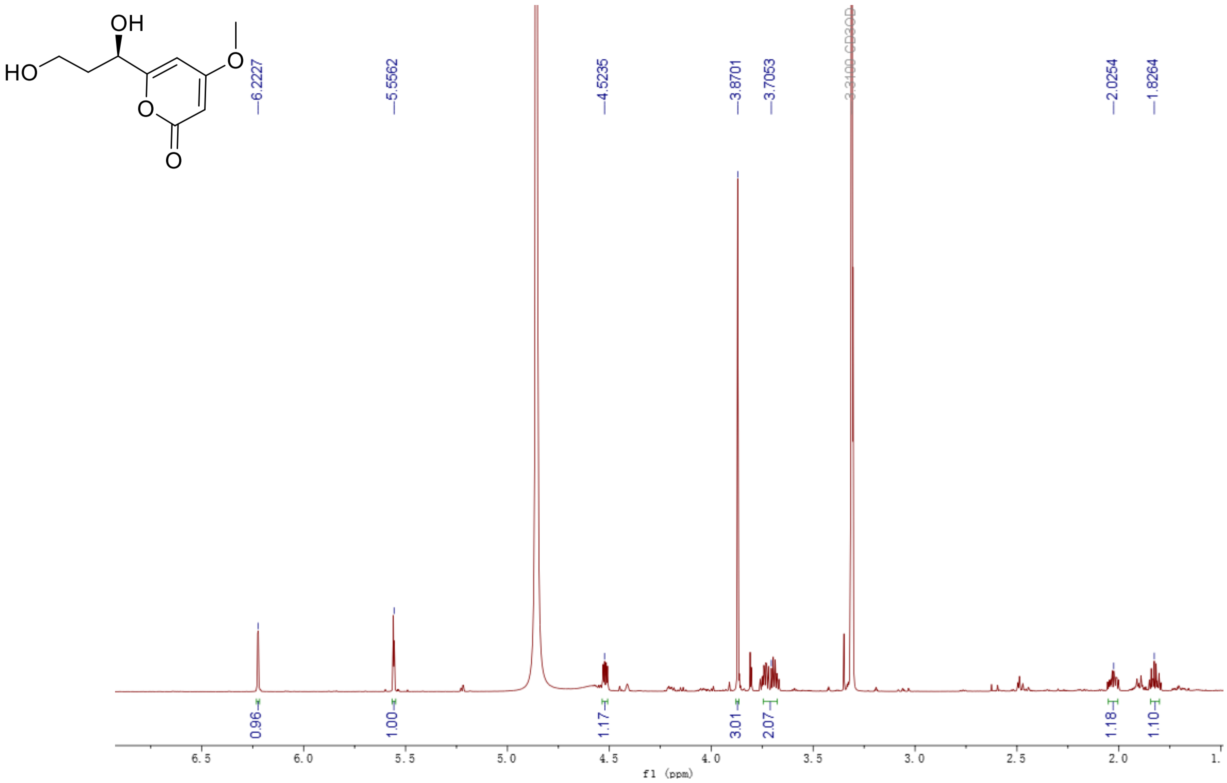

Fig. S84 ^{1}H NMR spectrum of **8** in methanol-d_4 (600 MHz).

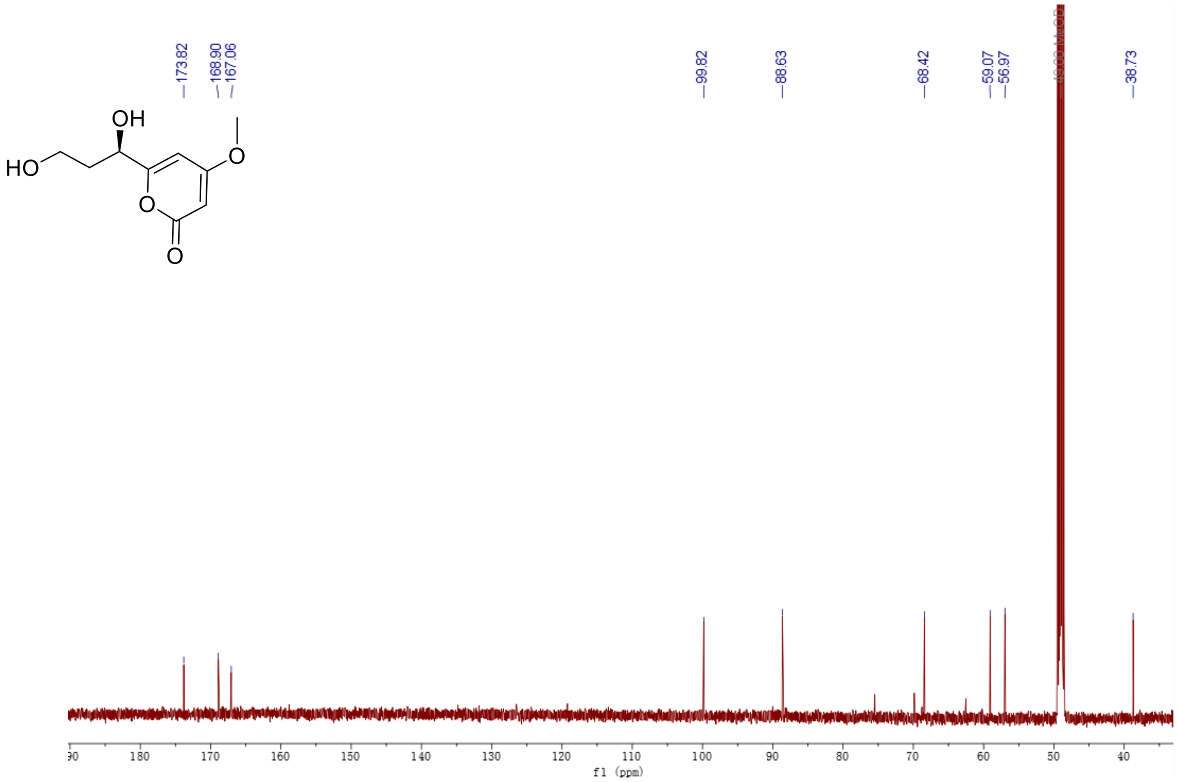

Fig. S85 ^{13}C NMR spectrum of **8** in methanol-d_4 (150 MHz).

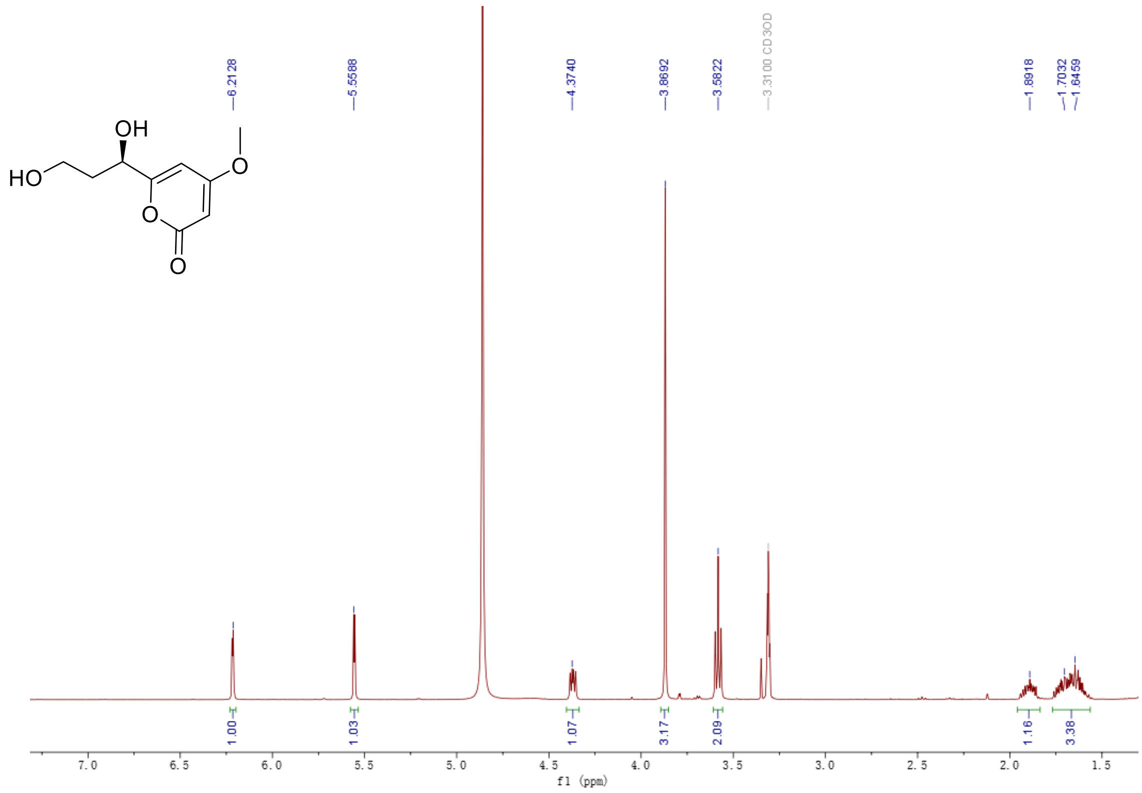

Fig. S86 ^{1}H NMR spectrum of **9** in methanol-d_4 (600 MHz).

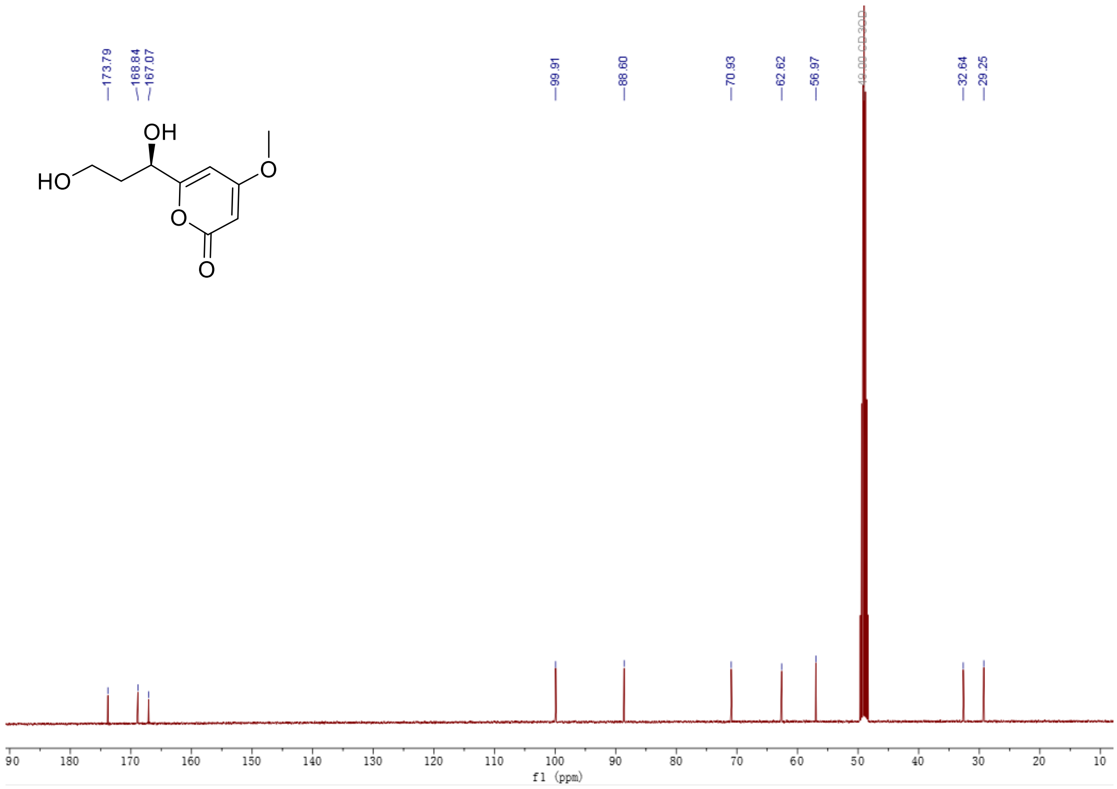

Fig. S87 ^{13}C NMR spectrum of **9** in methanol-d_4 (150 MHz).

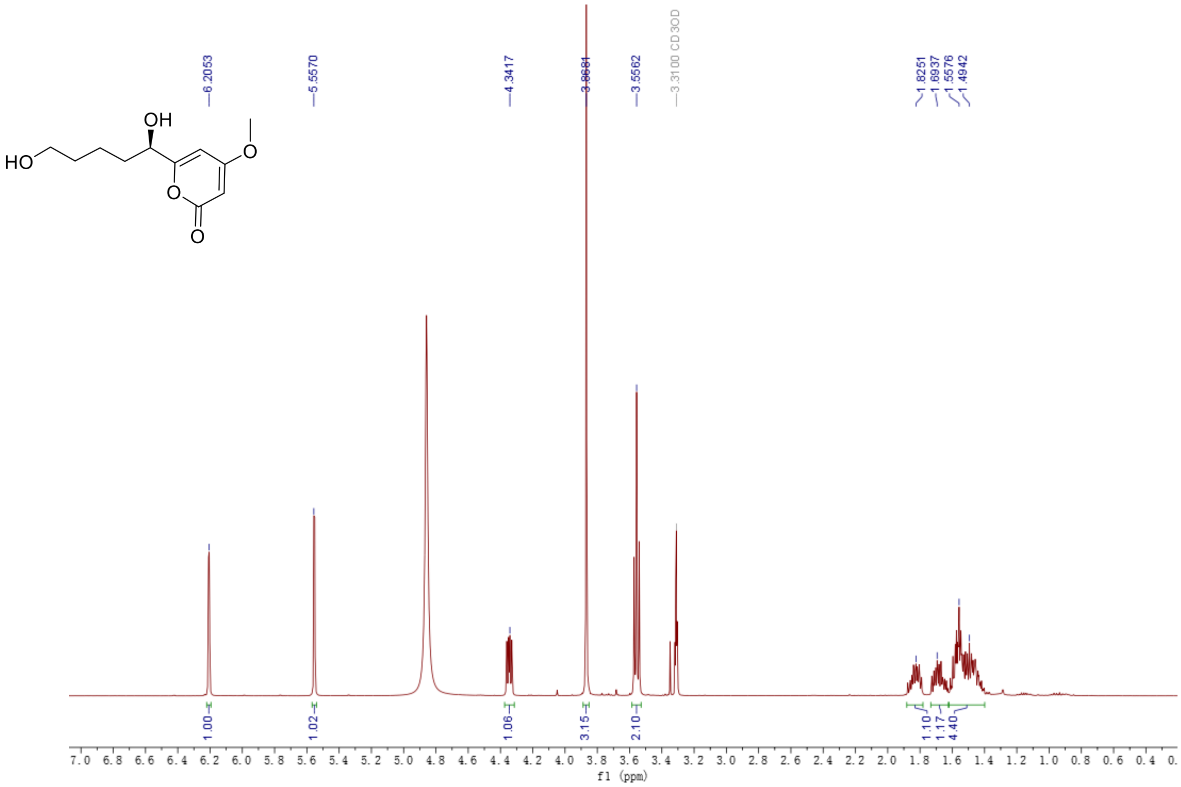

Fig. S88 ^{1}H NMR spectrum of **10** in methanol-d_4 (400 MHz).

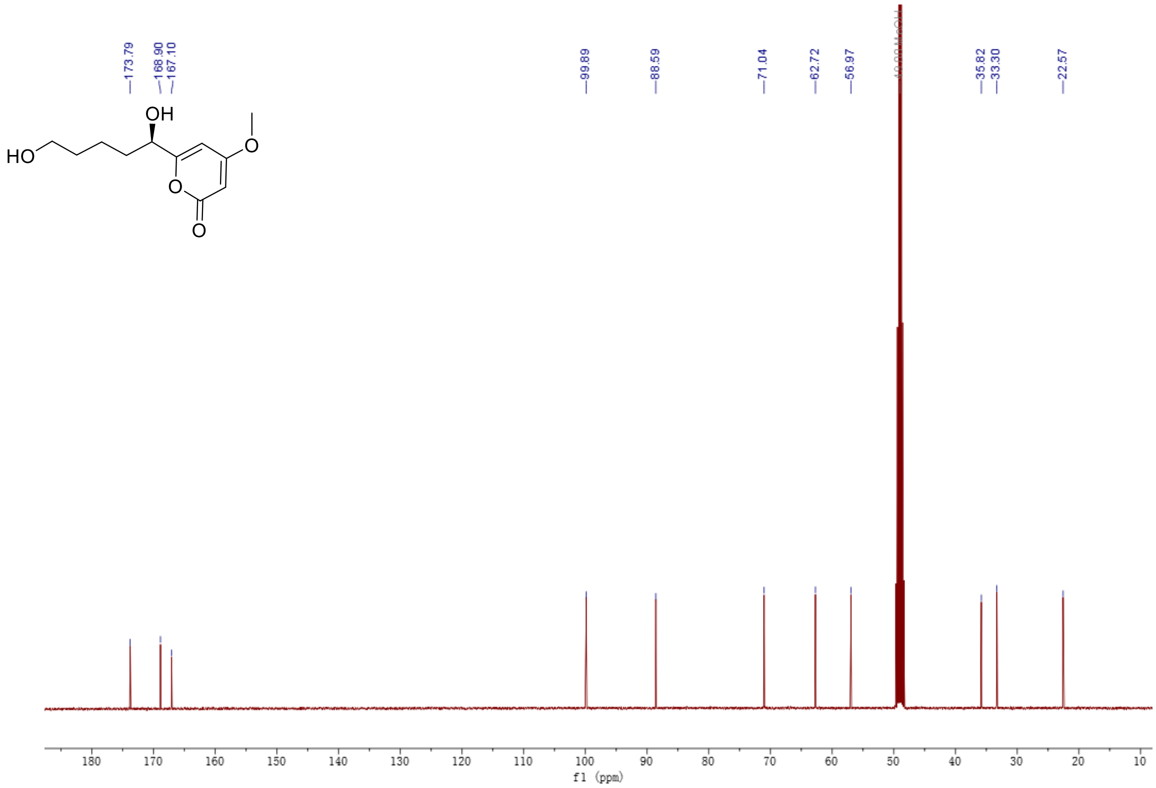

Fig. S89 ^{13}C NMR spectrum of **10** in methanol-d_4 (100 MHz).

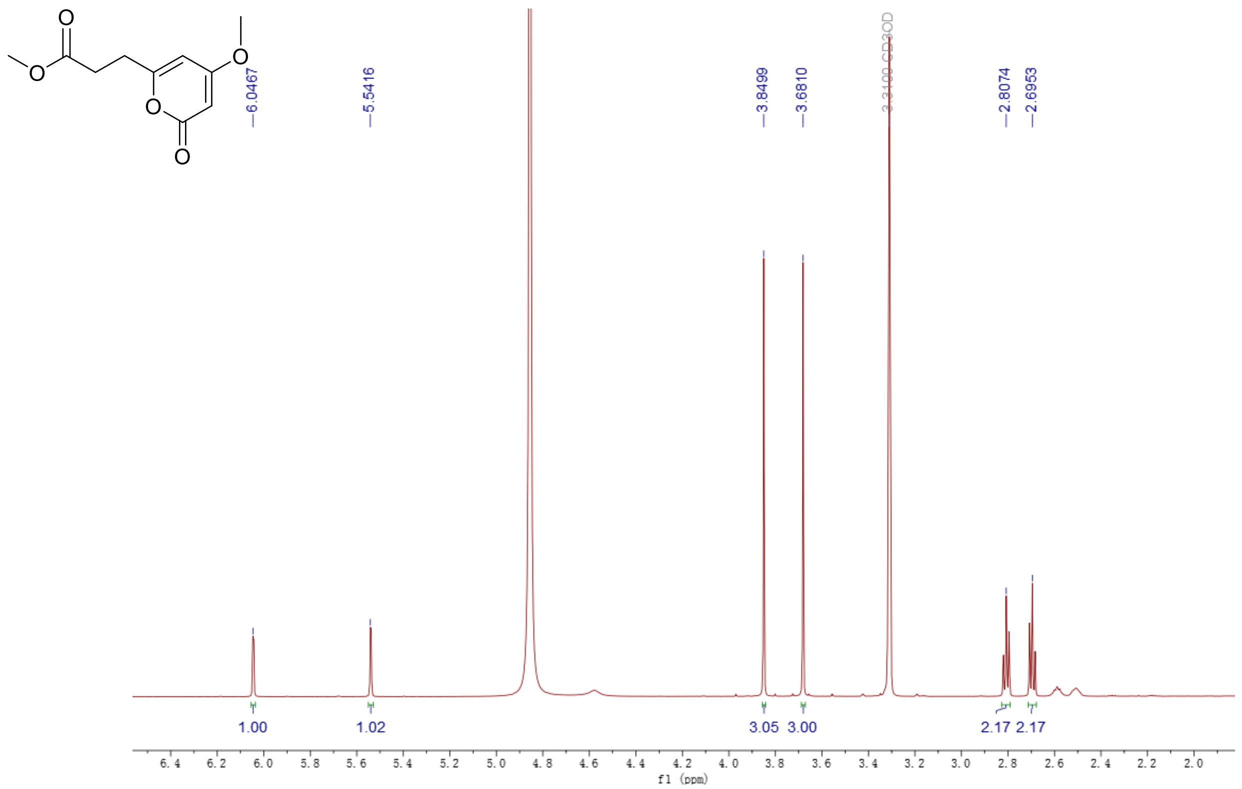

Fig. S90 ^{1}H NMR spectrum of **11** in methanol-d_4 (600 MHz).

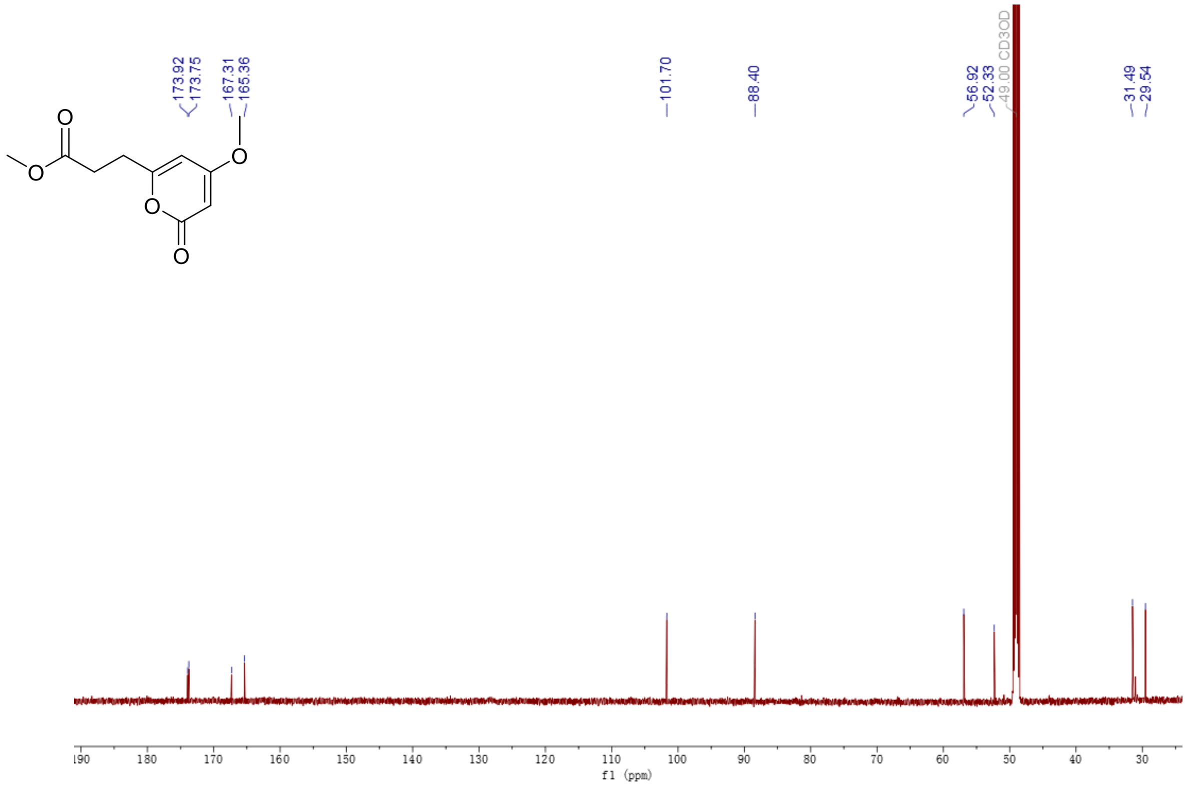

Fig. S91 ^{13}C NMR spectrum of **11** in methanol-d_4 (150 MHz).

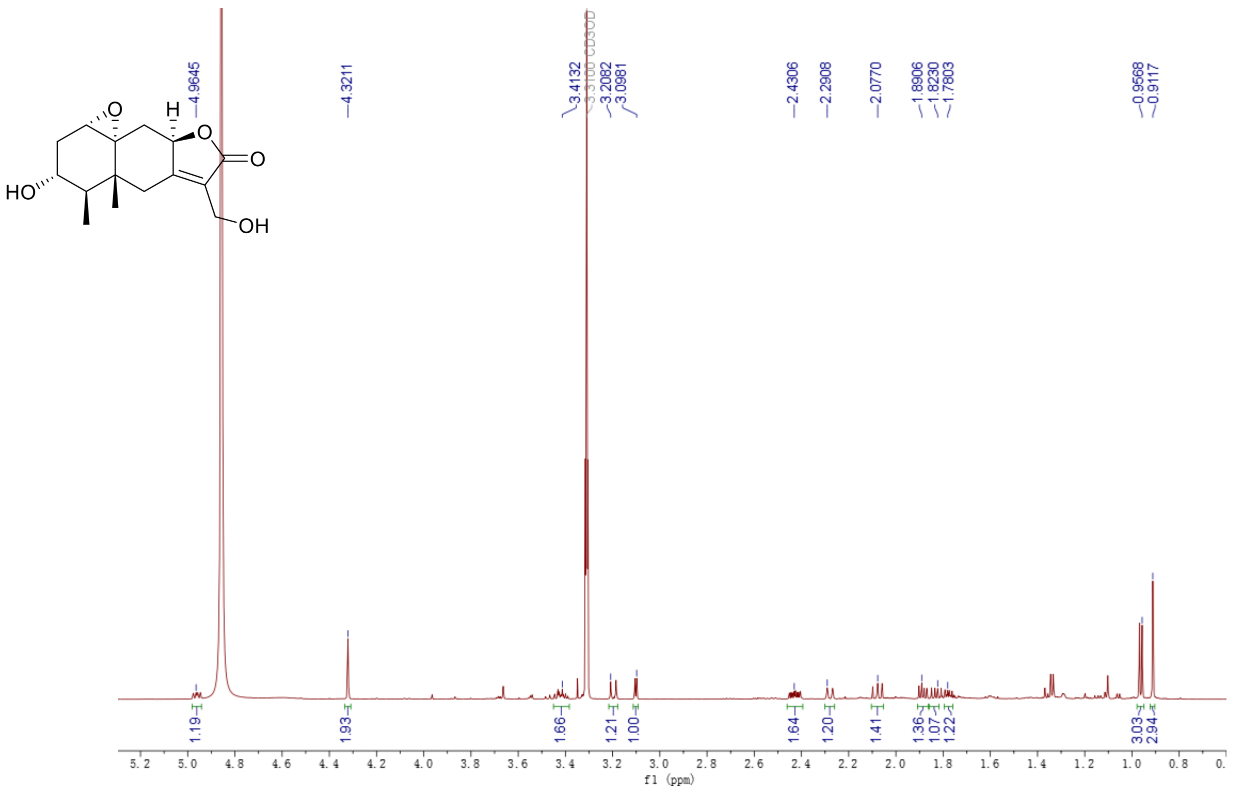

Fig. S92 ^{1}H NMR spectrum of **12** in methanol-d_4 (600 MHz).

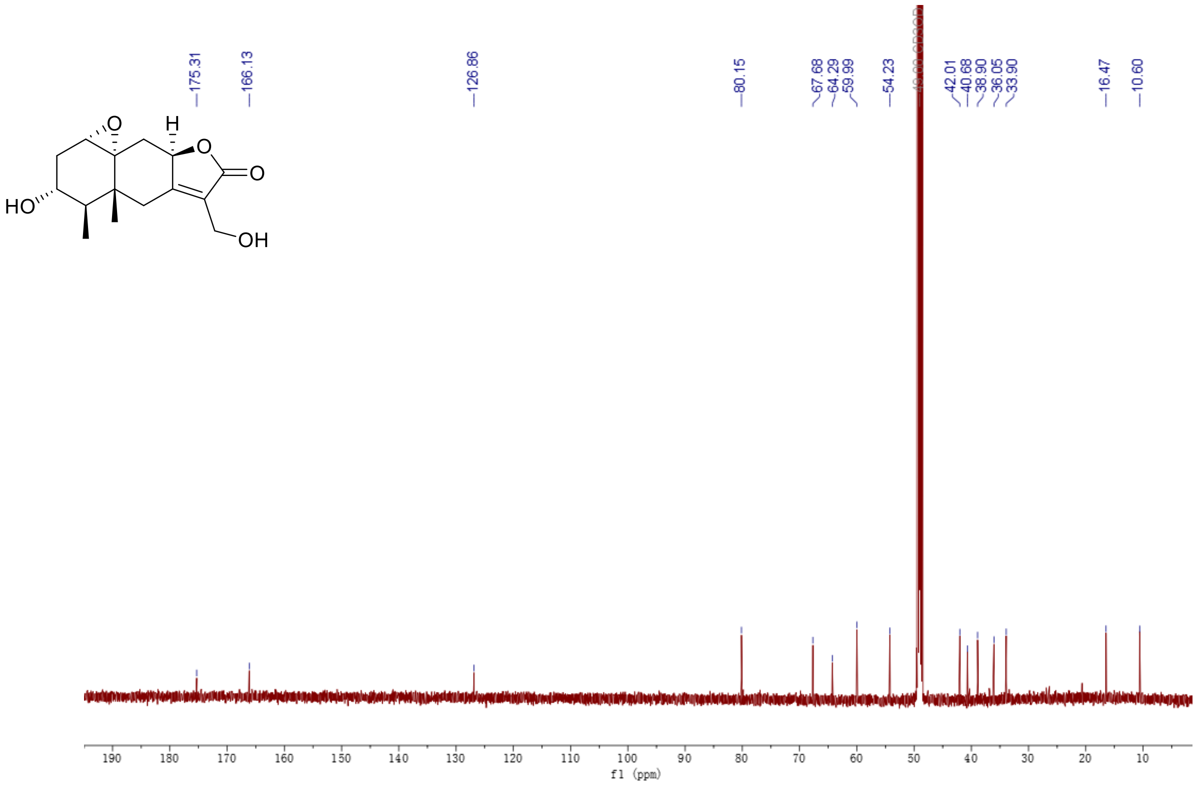

Fig. S93 ^{13}C NMR spectrum of **12** in methanol-d_4 (150 MHz).

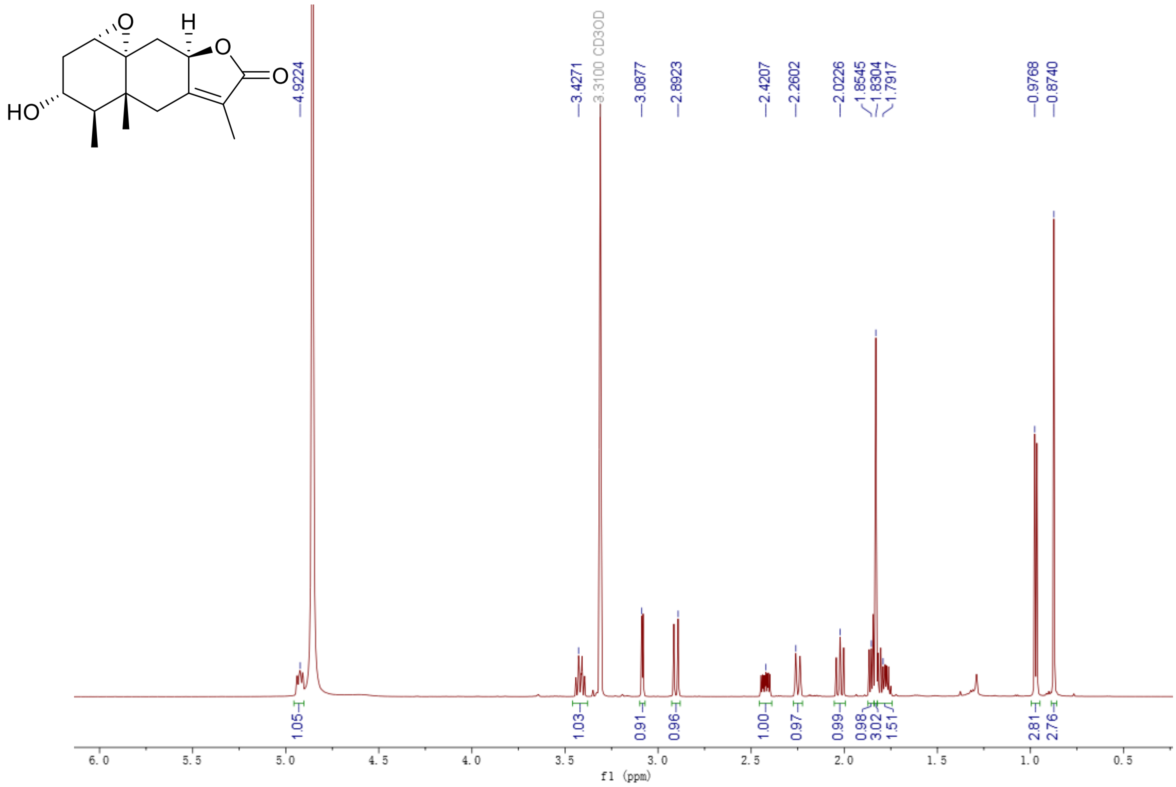

Fig. S94 ^{1}H NMR spectrum of **13** in methanol-d_4 (600 MHz).

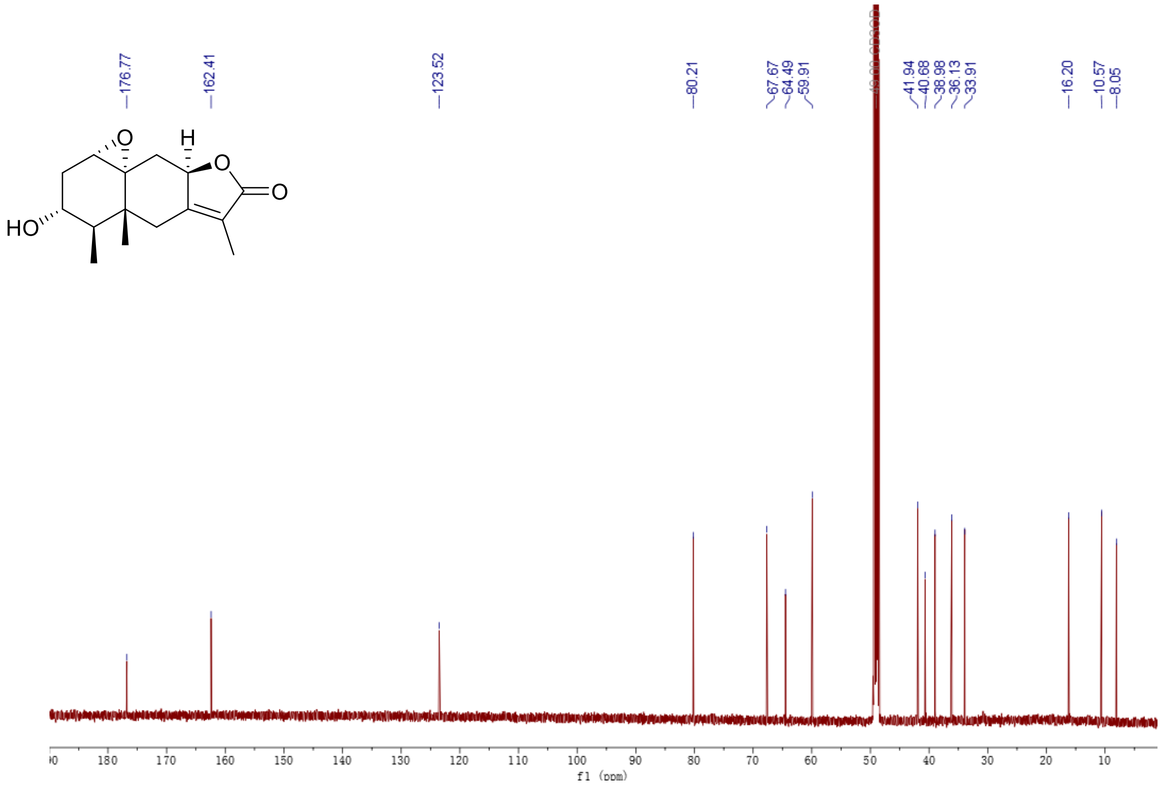

Fig. S95 ^{13}C NMR spectrum of **13** in methanol-d_4 (150 MHz).

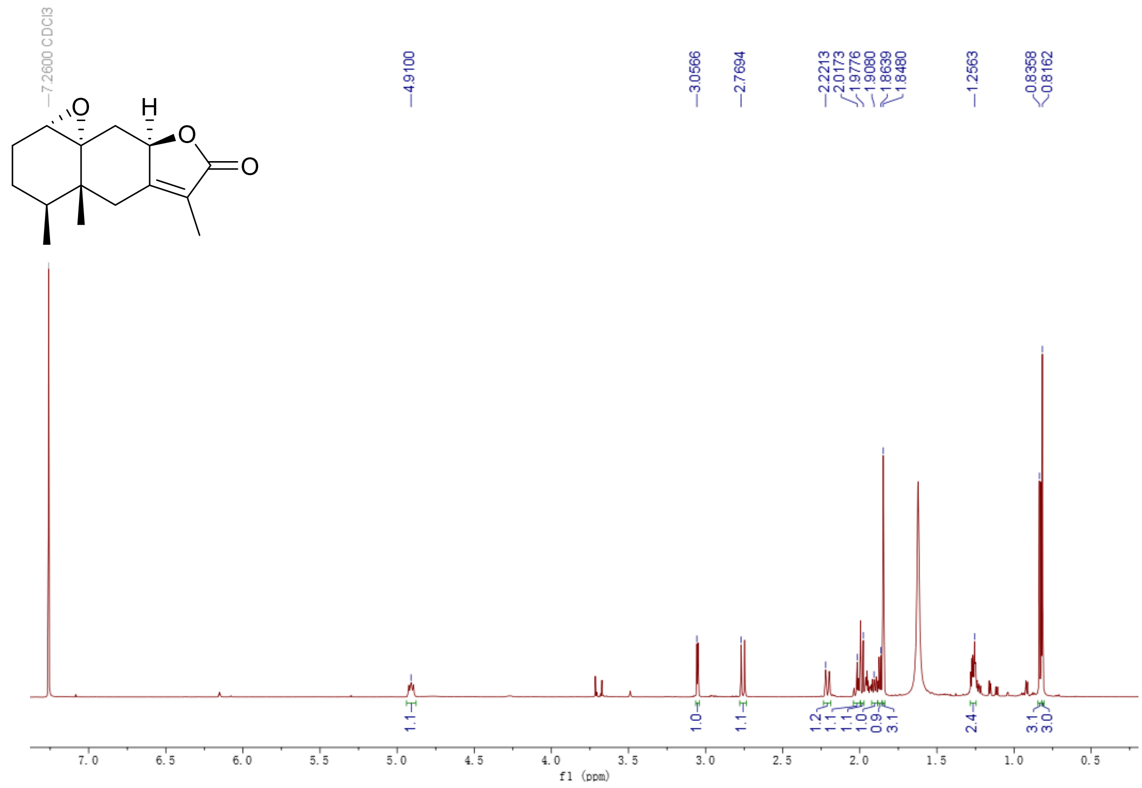

Fig. S96 ^{1}H NMR spectrum of **14** in chloroform-*d* (600 MHz).

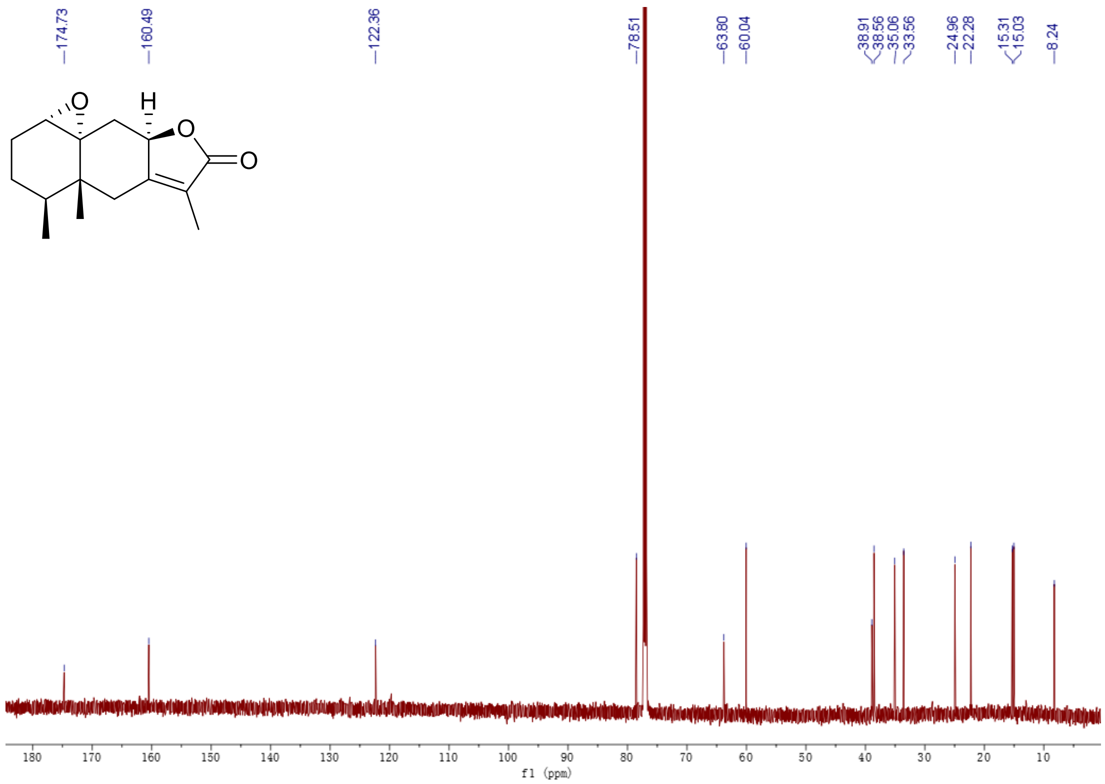

Fig. S97 ^{13}C NMR spectrum of **14** in chloroform-*d* (150 MHz).

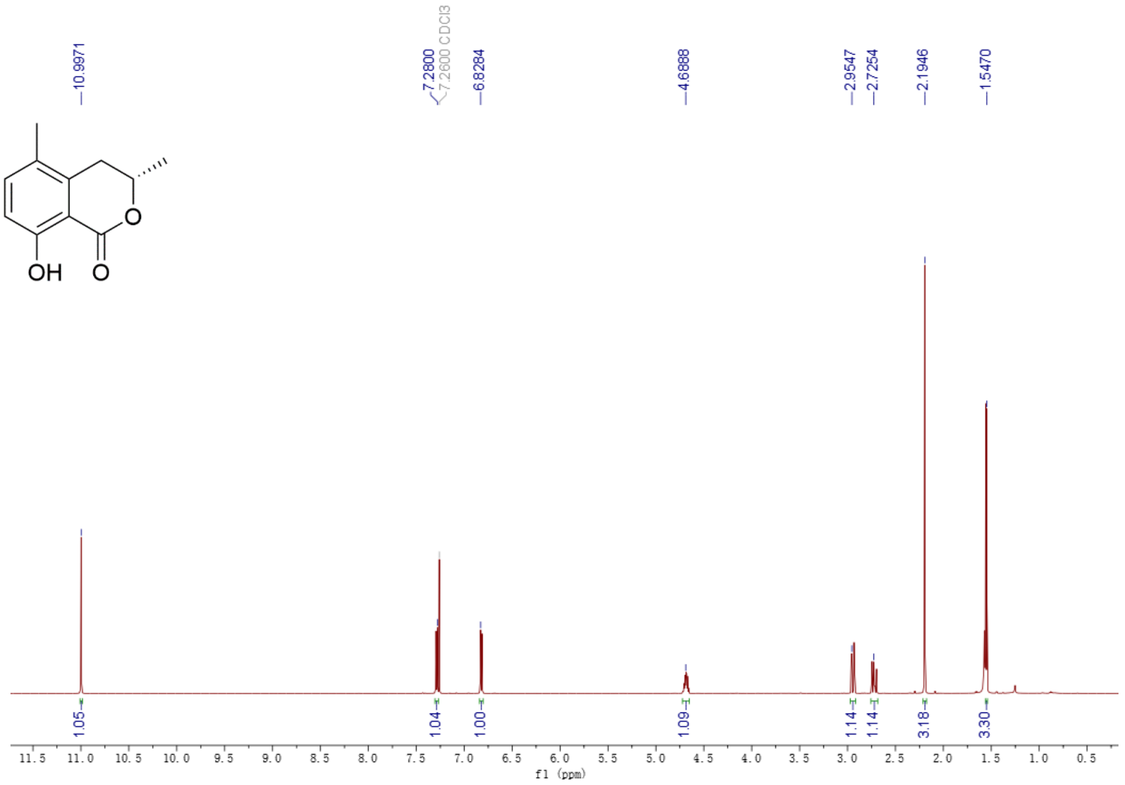

Fig. S98 ^{1}H NMR spectrum of **15** in chloroform-*d* (600 MHz).

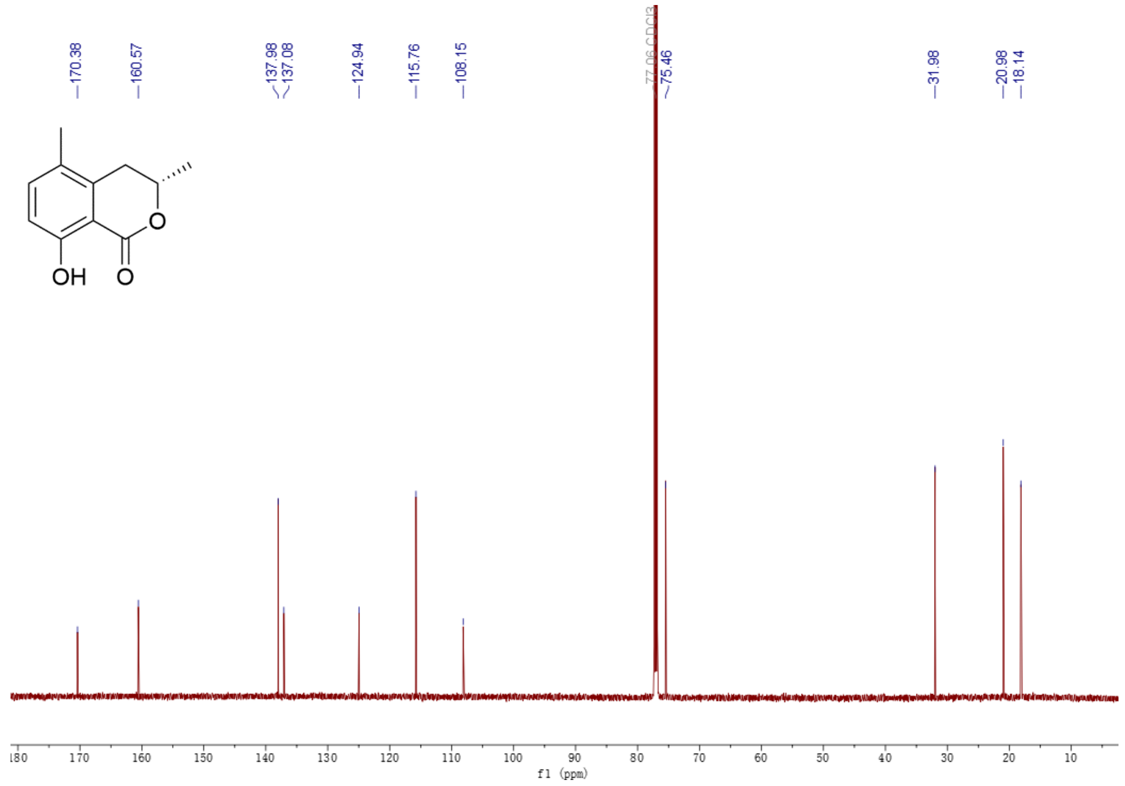

Fig. S99 ^{13}C NMR spectrum of **15** in chloroform-*d* (150 MHz).

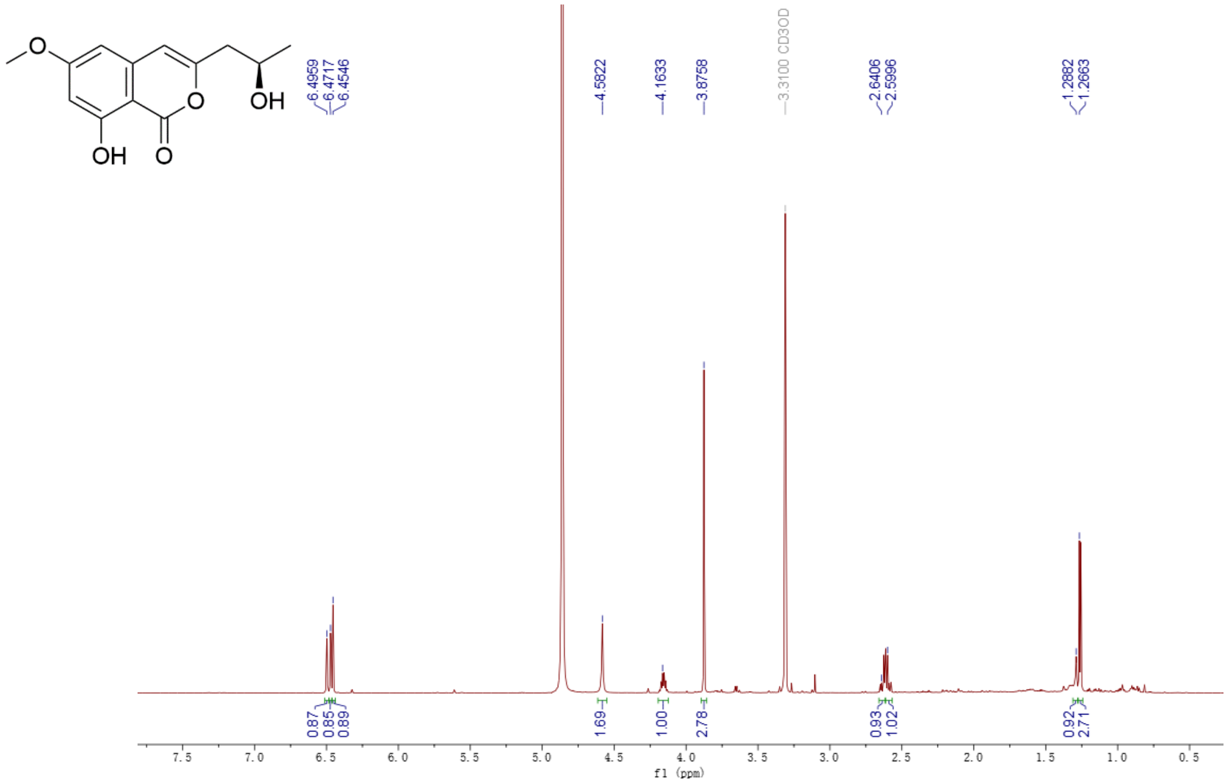

Fig. S100 ^{1}H NMR spectrum of **16** in methanol-d_4 (600 MHz).

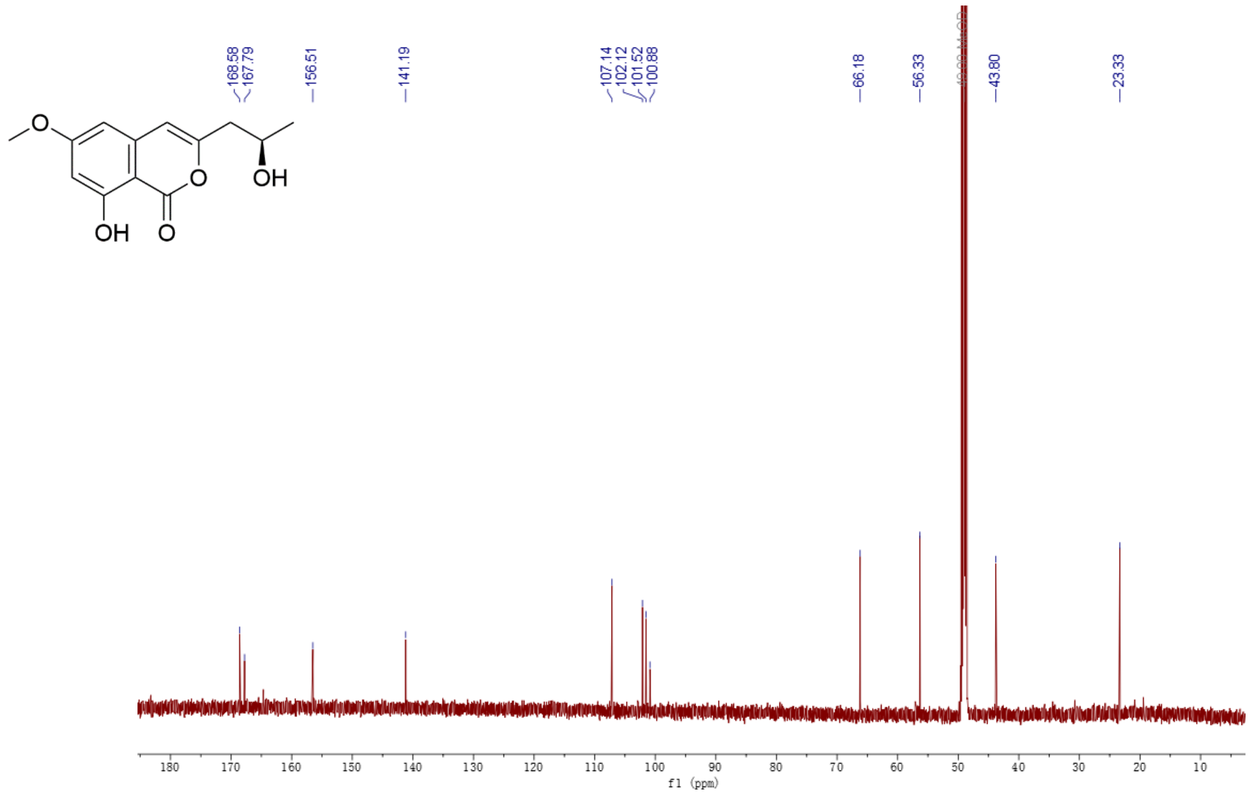

Fig. S101 ^{13}C NMR spectrum of **16** in methanol-d_4 (150 MHz).

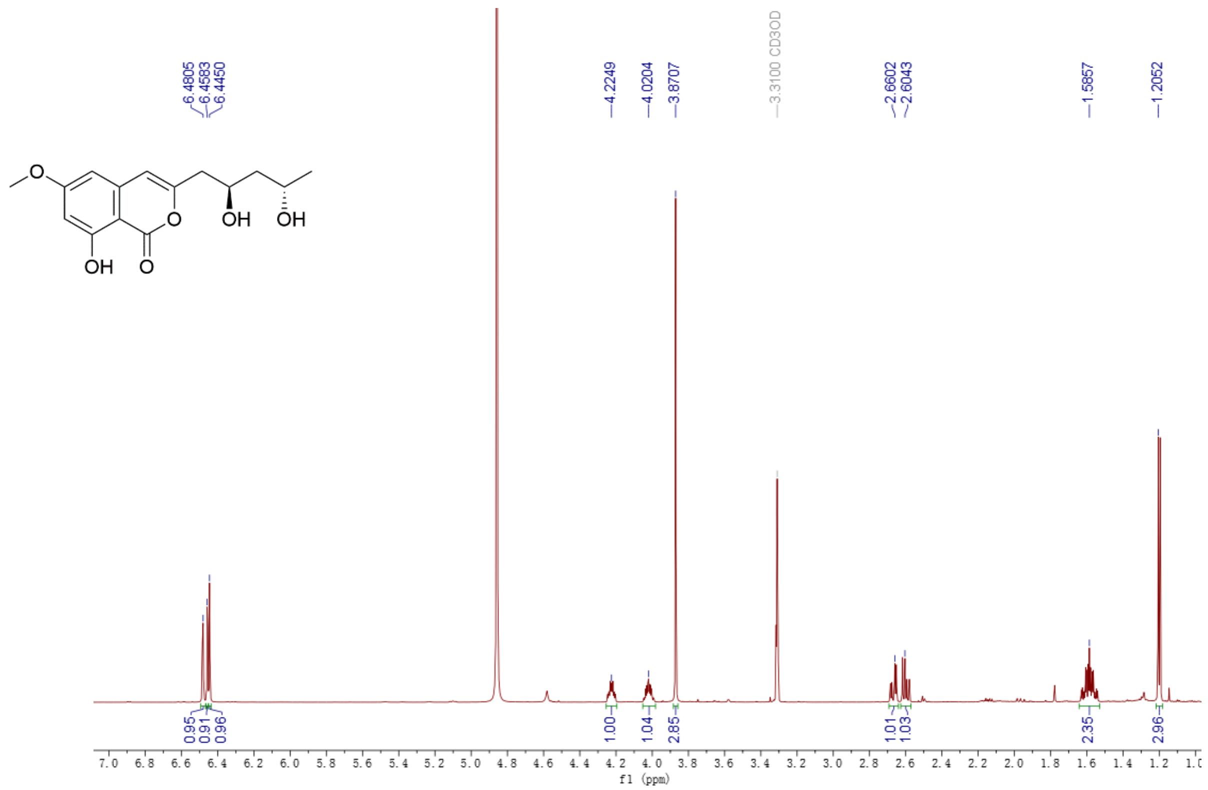

Fig. S102 ^{1}H NMR spectrum of **17** in methanol-d_4 (600 MHz).

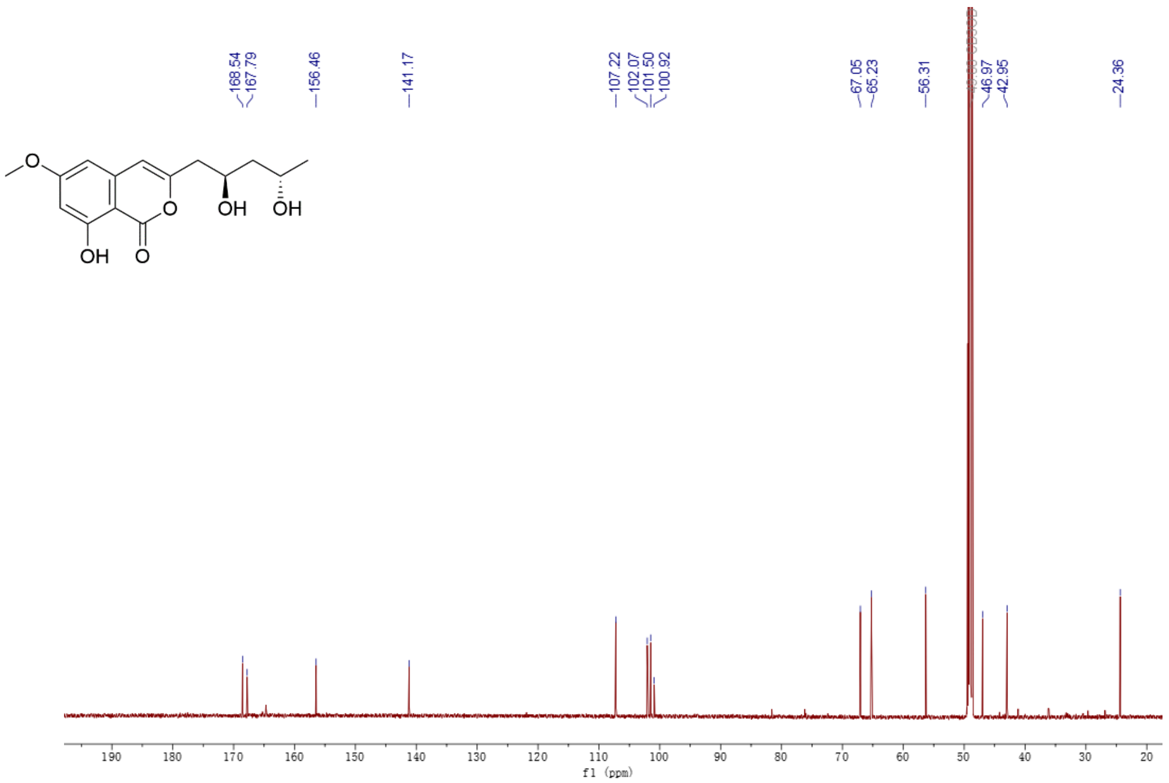

Fig. S103 ^{13}C NMR spectrum of **17** in methanol-d_4 (150 MHz).

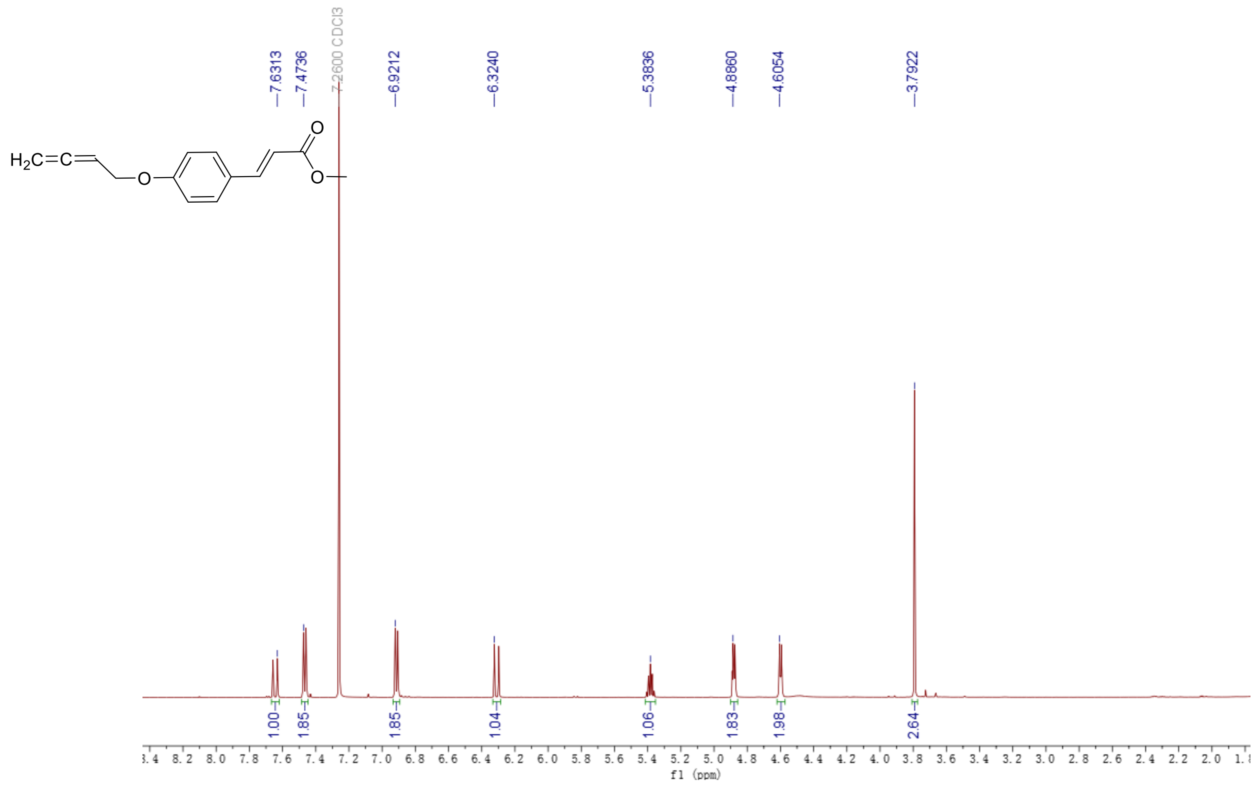

Fig. S104 ^{1}H NMR spectrum of **18** in chloroform-*d* (600 MHz).

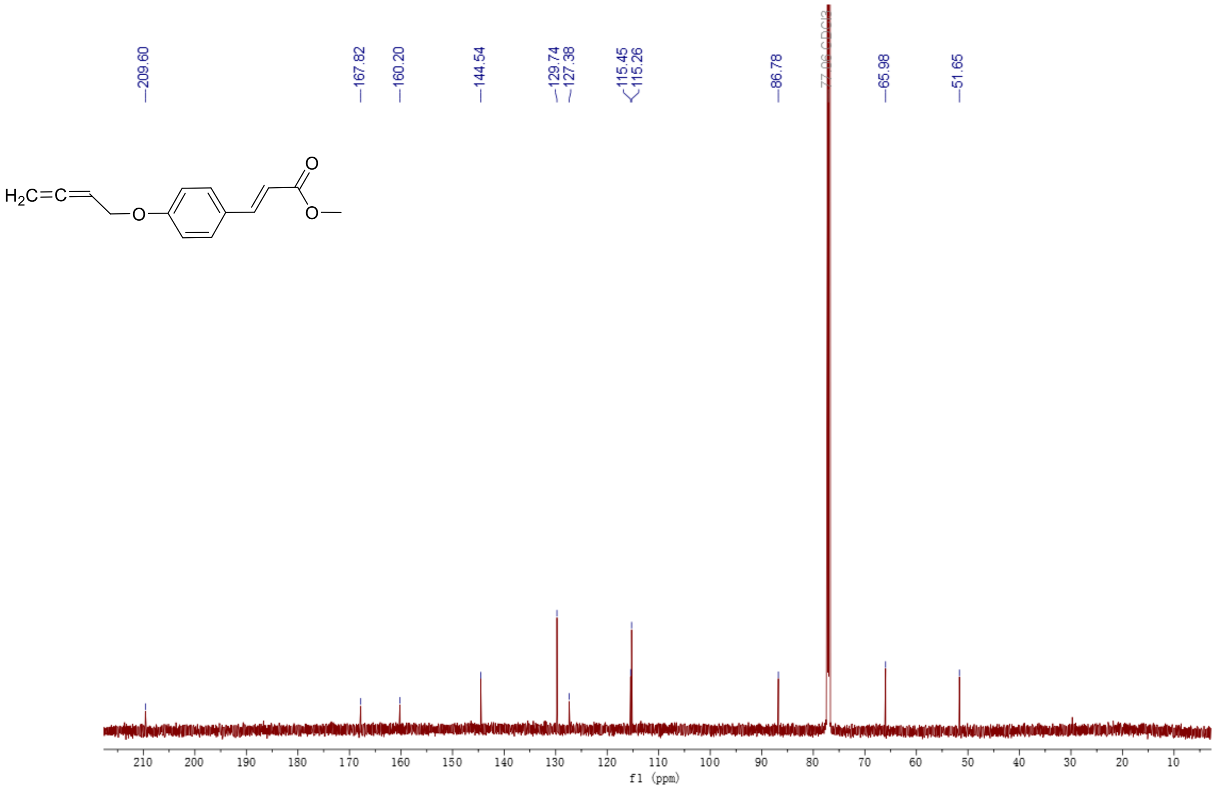

Fig. S105 ^{13}C NMR spectrum of **18** in chloroform-*d* (150 MHz).

HPLC system: Waters, Waters e2695 pump, Waters 2996 detector.

-Column: XB-C18, 5 μm, 4.6 × 250 mm.

-Solvent system.

Time (min), Flow rate (1 mL/min)	MeOH (%)	Water (%)
-	10	90
40	100	0
50	100	0

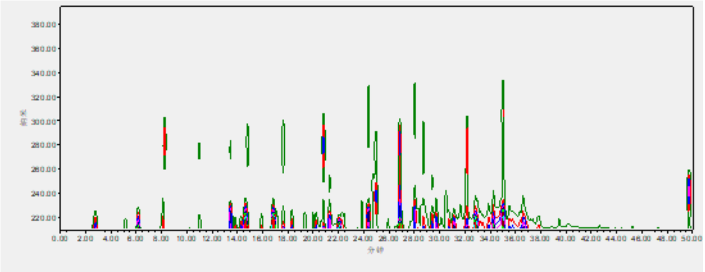

(a) HPLC analysis

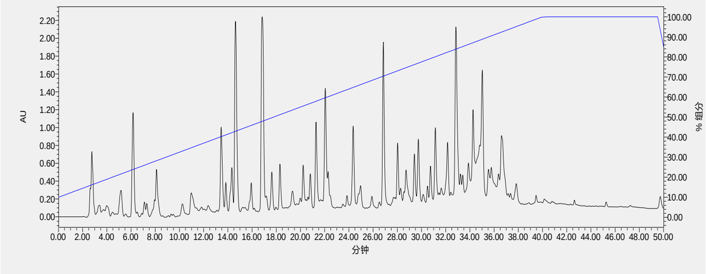

Wavelength at 215 nm.

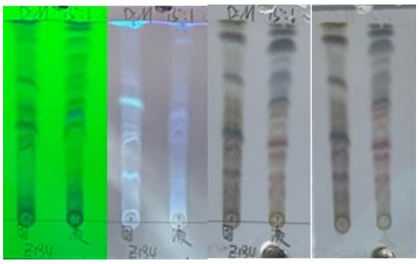

(b) TLC analysis (dichloromethane: methanol = 15:1) of crude extract.

Fig. S106 The TLC and HPLC profiles of the crude extract of *Xylaria* sp. Z184.
